# Photocatalytic Microenvironment Proteomics of Thiol-Mediated Uptake

**DOI:** 10.1021/jacsau.5c00432

**Published:** 2025-07-01

**Authors:** Saidbakhrom Saidjalolov, Yibo Wu, Giacomo Renno, Nicholas Rose, Jelena Gajić, Bertrand Pologne, Nicolas Winssinger, Vincent Mercier, Dimitri Moreau, Naomi Sakai, Stefan Matile

**Affiliations:** † Department of Organic Chemistry, 27212 University of Geneva, 1211 Geneva, Switzerland; ‡ National Centre of Competence in Research (NCCR) Molecular Systems Engineering, BPR 1095, 4002 Basel, Switzerland; § ChemBioMS Proteomics Platform, University of Geneva, 1211 Geneva, Switzerland; ∥ ACCESS Platform, University of Geneva, 1211 Geneva, Switzerland

**Keywords:** thiol-mediated uptake, dynamic covalent chemistry, photocatalytic microenvironment proteomics, photocatalytic proximity labeling, relay networks, off-equilibrium proteomics, genetic knockdown, cascade exchange, dynamic covalent inhibition

## Abstract

Although facilitated cellular entry of substrates with thiol-reactive motifs has been observed for decades, this so-called thiol-mediated uptake (TMU) remains poorly understood. We have proposed a mechanism of entry involving cellular proteins that form reversible dynamic covalent bonds with thiol-reactive cascade exchangers (CAXs), which is challenging to prove because the substrate–protein bond is transient and constantly shifting. Thus, with conventional proteomics analysis of TMU, continuing exchange during processing should result in labeling of the inert binders rather than the best exchangers, that is, repressors and intracellular targets, instead of the enablers of TMU. Any static covalent bonding to a binding site will also perturb the molecular relay network of interest. The emerging photocatalytic microenvironment mapping (μMap) proteomics, however, promises to catch snapshots of off-equilibrium relay networks without disturbing their flow. Exchange partners that are temporarily within <4 nm radius of photocatalyst–CAX conjugates should be irreversibly biotinylated without systematically interfering with TMU. μMap proteomics of this elusive flow of TMU was explored for three different photocatalyst–CAX conjugates. They were measured against CAX-free photocatalyst controls and dynamic covalent TMU inhibitors. Validated by genetic knockdown, solute carriers (MFSD5, SLC29A2), flippases (ATP11C), and tetraspanins (TSPAN8) are identified as primary exchange partners. This is rewarding because their canonical functions already involve local membrane reorganization. The result is a new understanding of the nature of TMU, which will be helpful to guide future progress toward control over cell penetration for drug delivery and drug discovery. It also highlights the unique potential of photocatalytic proximity labeling proteomics to elucidate off-equilibrium molecular relay networks without disturbing their flow.

## Graphical abstract

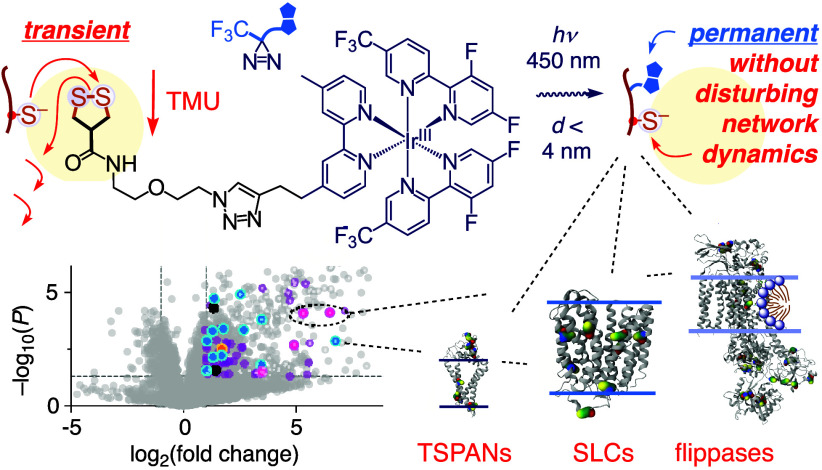

## Introduction

Cellular entry of exogenous chemicals, including drugs, proteins, and oligonucleotides, represents one of the main hurdles to achieving their intended functions. It is generally considered to occur through endocytosis, fusion, or direct translocation across the plasma membrane,^1^ but given the challenges and limitations of the first two approaches, the discovery of new ways to penetrate cells by direct translocation is of high importance.^2–9^ Thiol-mediated uptake (TMU)^10,11^ has been occasionally observed for decades^12,13^ but only recently began to gain substantial attention^2–5,10,11,14–19^ as an intriguing yet elusive alternative to well-established cell-penetrating peptides,^4,6,7,20,21^ which is also applicable to nanoparticles, including coacervates and liposomes.^3,15,22^ TMU refers to the cell-penetrating ability that arises in a substrate upon attachment of thiol-reactive motifs (Figure 1A,B) and is inhibited by thiol-reactive agents (Figure 1C).^10,11^ We found earlier that dynamic covalent cascade exchangers (CAXs), capable of undergoing multiple exchange reactions with thiolates/disulfides, are particularly powerful in enabling the uptake, suggesting the involvement of exchange cascades with several cysteine-containing cellular proteins (**P**) in TMU. The selectivities of TMU inhibition imply that several cascades coexist to form a dynamic covalent network of high complexity.^11^ In their simplest form, CAXs are cyclic disulfides. Oligomers and polymers qualify as well, as does a rich collection of other chalcogens,^23^ pnictogens^24^ and tetrels as exchange centers.^25^

**Figure 1:**
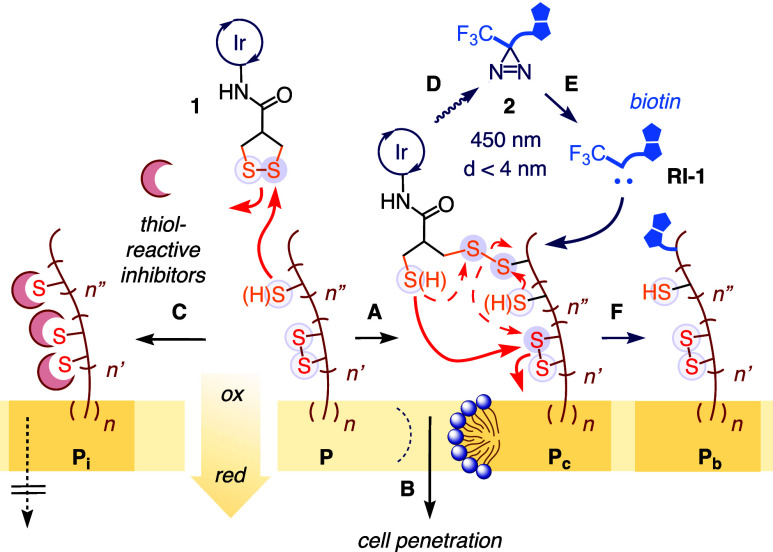
Systems design. Thiol-mediated uptake (A, B) occurs through successive dynamic covalent exchanges between CAX and cellular thiols/disulfides and, thus, can be inhibited with thiol-reactive agents (C). Exchange partners **P** are to be identified using CAX **1**, equipped with an Ir photocatalyst (pc) that (D) activates diazirine **2** within a radius of <4 nm to (E) produce carbenes that (F) stably label exchange partner **P**_**b**_ for proteomics analysis. **P**_**i**_: inhibited partner; **P**_**c**_: partner-CAX conjugate.

Only a few cellular exchange partners are known today (transferrin receptor TfR, integrins, PDIs). They have been found by intuition, pattern recognition,^11^ or conventional chemoproteomics^26^ and validated by genetic (knockdown) or chemical suppression (inhibitors).^11,26^ However, conventional chemoproteomics, which relies on permanent covalent bond formation between the probe and proteins, is not well-suited for TMU because it identifies inert protein-probe complexes with low off-rates rather than labile ones in dynamic exchange.^14,26^ Recent advances in proximity labeling methods have provided an alternative for studying transient protein interactions.^27–29^ These methods, which include genetically encoded enzymes like BioID, BioID2,^30^ TurboID, RapID,^31^ APEX, APEX2, or others,^32–35^ allow for precise labeling within a defined radius, offering a new toolkit for studying dynamic processes including TMU. When genetically encoded to a substrate of interest, these enzymes label vicinal proteins in a spatially and temporally controlled manner, capturing even weak and transient interactions. While the effectiveness of these methods has been widely validated by the scientific community,^27–29^ we opted for genetic engineering free photocatalytic microenvironment mapping (μMap) proteomics,^20,36–41^ one of the latest developments in the proximity labeling method,^21,42–47^ to possibly identify exchange partners contributing to TMU. In the currently leading approach, Ir photocatalysts (pcs) are attached to the object of interest, here a CAX as in **1** (Figure 1). Upon irradiation with visible light (blue light, 450 nm), Dexter energy transfer (Figure 1D) from pc* converts biotinylated diazirines **2** in a defined radius into reactive carbenes **RI-1** (Figure 1E). These carbene intermediates **RI-1** then covalently biotinylate nearby proteins **P**_**c**_ for pull-down and proteomics analysis of the covalent conjugates (**P**_**b**_) (Figure 1F). The permanent nature of protein labeling and the short effective distance (<4 nm)^20,36–41^ of energy transfer in the μMap approach promise to unravel transient cellular exchange partners accounting for TMU.

This design addresses the fundamental challenge to understand TMU on the molecular level and highlights a unique characteristic of photocatalytic proximity labeling proteomics. The proteins of interest are not inert binders but labile ones. Such proteins could serve as temporary relay posts for CAXs to transit to another protein via dynamic covalent exchange in a largely directional manner along a redox gradient, ultimately reaching intracellular targets with a much higher affinity that are, however, irrelevant for the understanding of TMU. In conventional proteomics of such relay networks, continuing dynamic covalent exchange of CAX during processing^26^ should favor labeling of the strong but inert binders rather than the highly reactive but labile exchangers, that is, repressors and intracellular targets, rather than the enablers of TMU. In addition, proteomics methods that are based on irreversible bonding to the binding sites, will render relay posts inaccessible and perturb the network of interest. Compared to such established methods, photocatalytic proximity labeling appears unique in permanently and selectively labeling participants of off-equilibrium relay networks, that is exchange cascades, without systematically perturbing the flow of interest. Measured against pertinent controls, photocatalytic proximity labeling thus offers unique promise to elucidate the off-equilibrium dynamic covalent networks accounting for TMU.

## Results

### Methods Development

Asparagusic acid derivatives (AspA) as in **1** (Figures 1 and 2) were tested first as the classical, original small-molecule CAX.^48^ The standard Ir photocatalyst was attached to yield pc-CAX dyad **1** in a few unproblematic steps (Schemes S2–S3). Its specific accumulation in the Golgi through dynamic covalent double palmitoylation as described,^49^ and inhibition by established CAXs (below) supported that pc-AspA **1** enters cells by TMU (Figures S11 and S16). The biotinylated diazirine **2** was prepared following reported procedures (Scheme S6).^38^ In the CAX-free pc-control **3**, the cyclic disulfide moiety of the pc-AspA conjugate **1** was replaced by a phenyl group. This CAX-free pc-control **3** enters cells but neither accumulates in the Golgi (Figure S11) nor is impeded by TMU inhibitors (Figure S15). These opposite characteristics demonstrated that TMU of pc-AspA **1** (and other TMU-positive pc-CAX conjugates used in this study) is not affected by the Ir catalyst.^41^ Photocatalytic activities of new pcs **1** and **3** were confirmed to be comparable in vitro (Figures S20–S26).

**Figure 2:**
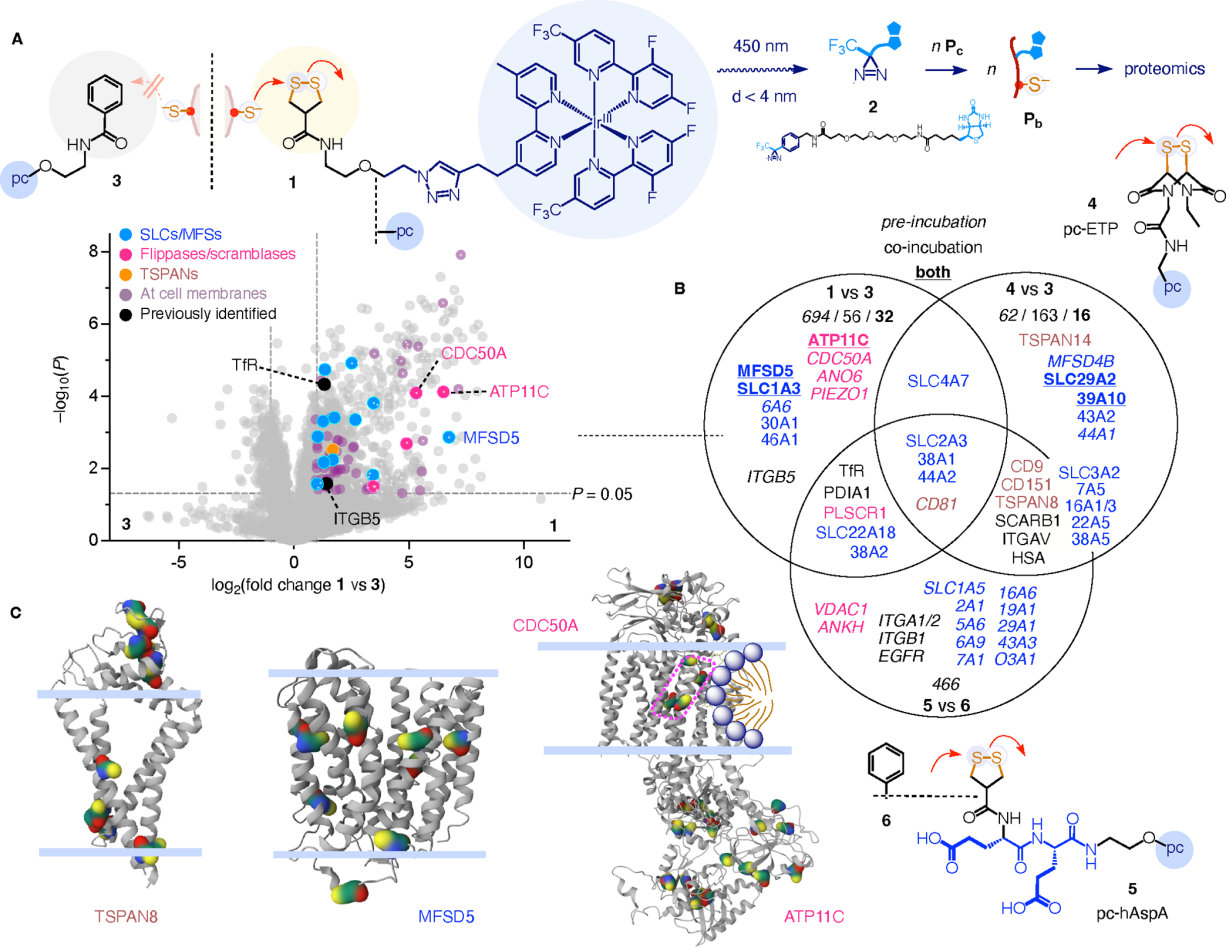
Microenvironment mapping of thiol-mediated uptake. (A) Representative proteomics volcano plot for pc-AspA **1** (right) compared to disulfide-free control **3** (left), obtained after preincubation of HK cells with **1** or **3** (20 min) followed by washing, addition of **2** and irradiation at 450 nm for 15 min. Purple: proteins at cell membranes, blue: SLCs/MFSs, magenta: flippases/scramblases, orange: tetraspanins, black: previously identified targets, white dots: heavily imputed, thus unreliable data points, mainly due to the undetectability with control probes. (**B**) Notable enriched membrane proteins with pc-AspA **1** (left), pc-ETP **4** (right), and pc-hAspA **5** (below) compared to controls **3** or **6** observed for coincubation, preincubation (italic), or both (bold); color codes as in A. (C) SWISS-MODELs^53^ of TSPAN8 and MFSD5 and the crystal structure of ATP11C/CDC50A heterodimer (PDB 6lkn) with highlighted cysteines (Gaussian surface) and membrane interfaces (pale blue).

Two general procedures were employed for μMap proteomics. Under preincubation conditions, cells were first incubated with pc-AspA **1** or CAX-free control **3**, then rinsed to remove excess photocatalysts before the addition of diazirine **2** and irradiation at 450 nm. Under coincubation conditions, unbound photocatalysts were present during irradiation. Both methods have their advantages and limitations. Preincubation detects only stably bound CAXs, while coincubation risks to produce more noise and false positives. A homemade photoreactor, based on the design of the Wisconsin photoreactor platform,^50^ was crucial for success (Figure S1). After 15 min of irradiation, the biotinylated proteins were pulled down and subjected to quantitative proteomics analysis.

The ratios of each protein photolabeled in the presence of pc-AspA **1** compared to CAX-free pc-control **3** were calculated following standard procedures^51^ and summarized in volcano plots (Figures 2A, 3A, S29–S32). The obtained protein fold change on the *x*-axis shows the enrichment of protein labeling by pc-AspA **1** over control **3** on the right and the contrary on the left. The *y*-axis of the volcano plot reports the calculated *P*-value,^51^ that is, the probability of significance of each observation. In other words, the volcano plot shows proteins selectively labeled by pc-AspA **1** on the right side, with significance increasing from the bottom center to the top right, while those labeled by the CAX-free pc-control **3** are on the left side (Figure 2A). Proteins in the middle are equally labeled by both pc-AspA **1** and pc-control **3**. Since the only structural difference between pc-AspA **1** and control **3** is the presence or absence of AspA, this comparison ensures that the selectivity of protein labeling stems from contact with AspA, and neither from the photocatalyst^41^ or from other motifs.

**Figure 3:**
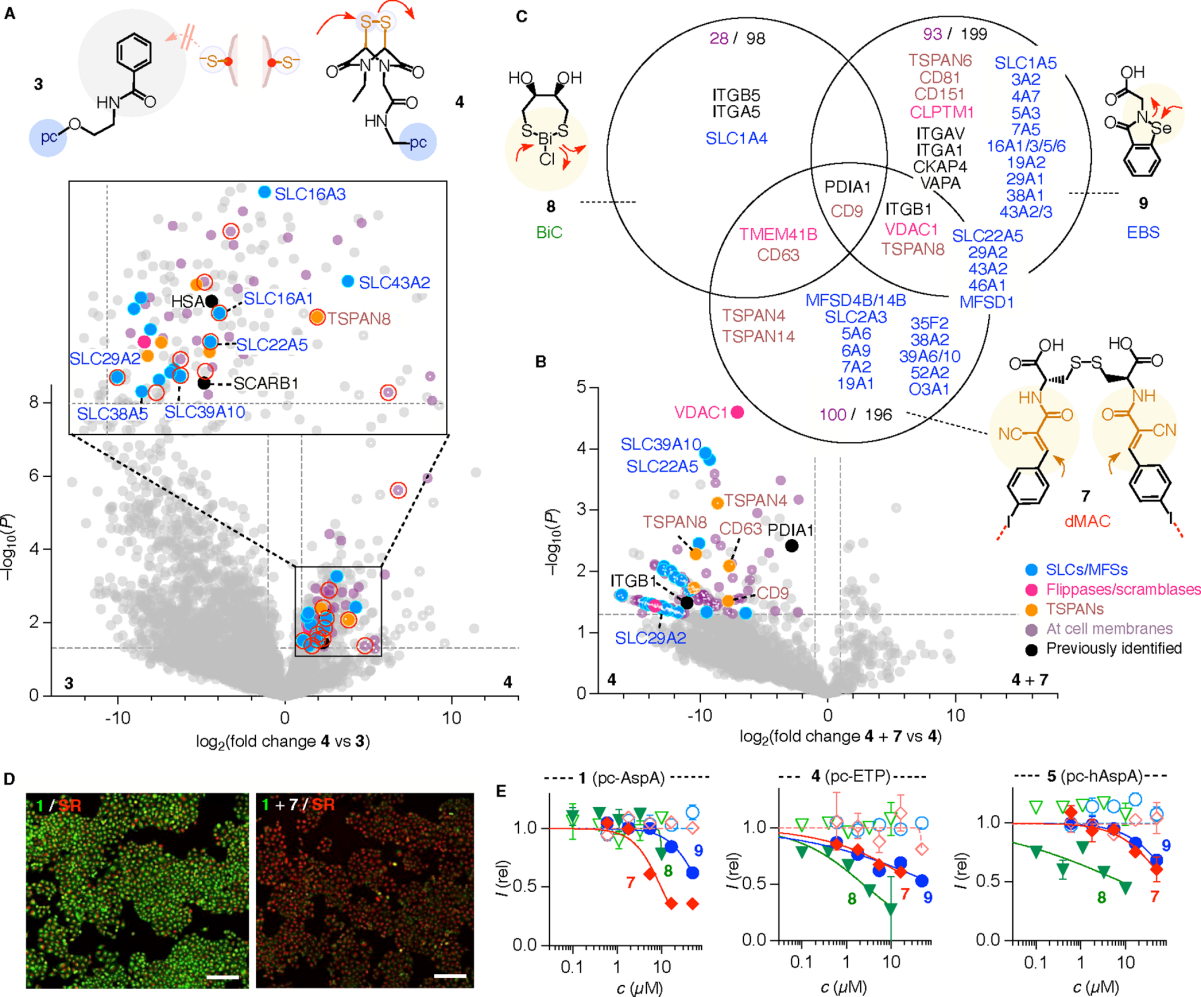
TMU inhibitors in microenvironment mapping proteomics. (A) Zoomed (top) and complete μMap volcano plot (bottom) for pc-ETP **4** (right) compared to CAX-free control **3** (left) obtained after coincubation of HK cells with **2** (15 min) plus **4**/**3** (20 min) followed by irradiation at 450 nm for 10 min; purple: at cell membranes, blue: SLCs/MFSs, magenta: flippases/scramblases, orange: tetraspanins, black: previously identified targets, white dots: heavily imputed data points, red circles: significantly inhibited proteins (compare C). (B) Representative μMap proteomics volcano plot for pc-ETP **4** with inhibitor **7** (right) compared to **4** alone (left) obtained after coincubation of HK cells with/without **7** (1 h) plus pc-ETP **4** (20 min) plus **2** (15 min) and irradiation at 450 nm for 10 min. (C) Summary of μMap results for the inhibition of pc-ETP **4** by dMAC **7** (bottom), BiC **8** (left), and EBS **9** (right) with numbers of significant proteins (purple: on plasma membranes, black: all); other colors as in A and B. (D) Imaging of photocatalyst uptake (wide field) in HK cells incubated with pc-AspA **1** (10 μM, green) without (left) and with dMAC **7** (50 μM, right; red: SYTO deep red for nuclei; scale bars, 200 μm). (E) Representative dose–response curves for uptake (filled symbols) of pc-AspA **1** (left), pc-ETP **4** (middle), and pc-hAspA **5** (right; all 10 μM) with varying concentrations of dMAC **7** (red diamonds), BiC **8** (green triangles), and EBS **9** (blue circles). Empty symbols: cell viability.

For pc-AspA **1**, the total number of significantly enriched proteins (log_2_(fold change) ≥1, −log_10_(*P*) ≥ 1.3) detected under preincubation conditions exceeded those by coincubation by far (Figures 2B and S31). This finding was consistent with the relatively slow detachment kinetics of AspA, evidenced by its strong retention on thiol-exchange columns.^52^ Particularly noteworthy among the enriched plasma membrane proteins (Figure 2A, purple) was the high proportion of detected MFS-SLCs (blue^53^) and flippases/scramblases (e.g., ATP11C, magenta). The previously validated transferrin receptor (TfR, black)^26^ was also among the top hits, supporting the validity of the current proteomics results. Other nonvalidated hits from conventional proteomics^26^ were not significantly enriched.

### CAX Variations

Epidithiodiketopiperazines (ETPs) are bioinspired highly strained cyclic disulfides and one of the most powerful CAXs for TMU.^54^ Conjugated to Ir photocatalyst (Scheme S4), pc-ETP **4** was found to enter nuclei after a short incubation time (Figure S10). This intracellular localization was as for the corresponding fluorescein (Fl) conjugate,^54^ again confirming negligible interference from the Ir photocatalyst to TMU of pc-ETP **4**. Operational TMU was supported by uptake inhibition with established CAXs (below, Figure S18). Consistent with poor retention on thiol-affinity columns^52^ and contrary to AspA, microenvironment mapping of photocatalyst-ETP dyads **4** afforded more labeled proteins under co- rather than preincubation conditions (Figures 2B, 3A, S29 and S32). Outstanding with pc-ETP **4**, particularly under coincubation conditions, was the high number of SLCs and TSPANs. The difference in proteins enriched by pc-AspA **1** and pc-ETP **4** is consistent with our earlier findings by inhibitor screenings, indicating that ETP and AspA enter cells through different pathways.

Considering the emergence of SLCs as potential primary partners with pc-ETP **4**, we wondered whether their less prominent role with pc-AspA **1** might be due to its hydrophobicity, which could accelerate the uptake beyond detectability by proximity labeling photoproteomics, particularly under the more revealing preincubation conditions (Figure 2A,B). To slow down TMU flow for more effective photocatalytic microenvironment mapping, the more hydrophilic pc-hAspA **5** with two glutamates between CAX and the photocatalyst was designed, synthesized, and evaluated (Figure 2B and Scheme S5). Preserved TMU of pc-hAspA **5** was demonstrated by its cell penetration activity that could be hindered by established TMU inhibitors (Figure S17). With this hydrophilic pc-hAspA **5** μmapped against the corresponding CAX-free pc-control **6** to subtract contributions of the Ir photocatalyst and the hydrophilic linker, a rich collection of SLCs also became detectable with AspA under preincubation conditions (Figures 2B, bottom, S30). Again, with pc-hAspA **5**, TfR was enriched, along with a variety of established candidates (ITGs, PDI, SCARB1,^26^ etc.).

### TMU Inhibitors

Inhibition of uptake with thiol-reactive agents is a hallmark of TMU (Figure 1C).^10,11^ Patterns generated by inhibitor screenings have been instrumental in decoding the exchange networks accounting for TMU and identifying drug discovery motifs, antivirals and beyond.^11,23^ The same inhibitor screens were applied to all new pc-CAXs to demonstrate their cellular entry by TMU (Figures 3, S15–S18, Tables S1–S4). For instance, uptake of pc-AspA **1** was efficiently inhibited by dMAC **7**, an excellent TMU inhibitor that operates with dynamic covalent Michael additions in combination with a halogen-bonding switch (Figure 3D).^25^ Against pc-AspA **1**, dMAC **7** inhibited better than the pnictogen-centered BiC **8** ^24^ or EBS **9** (Figures 3E and S16). In contrast, the inhibition of pc-ETP **4** and pc-hAspA **5** was the best with BiC **8**, better than dMAC **7** or EBS **9** (Figures 3E, and S17–S18). The selectivities between different inhibitors were comparable for pc-CAXs and Fl-CAXs (Figures S15–S18 and Tables S1–S4).^11^

For μMap proteomics, TMU inhibitors were considered as rational tools to refine the interpretation of volcano plots of pc-CAXs. The volcano plot comparing the proteins enriched by pc-ETP **4** in the presence of a generally strong inhibitor dMAC **7** (Figure 3B, right) compared to pc-ETP **4** without inhibitor (left) revealed a massive reduction of labeling in the presence of this inhibitor. This global change was consistent with competitive dynamic covalent inhibition of many exchange partners of pc-ETP **4** by dMAC **7**. The analogous volcano plots were recorded for pnictogen- and chalcogen-centered inhibitors **8** and **9** (Figures S33–S34). Different inhibitors were found to interfere with some of the same but mostly different proteins, which is unsurprising given the insights from inhibitor screening studies (Figure 3C). Cross-comparison of these volcano plots with inhibitors (Figures 3B, S33–S34) and the original volcano plot of pc-ETP **4** measured against the CAX-free control **3** (Figure 3A) revealed that only a few proteins appear significant in both plots (red circles, Figure 3A). Among these targets were several of the previously noted SLCs and TSPAN8.

### Partner Validation

Genetic knockdown utilizing siRNA technology was employed to validate the participation of several notable proteins in TMU.^11^ Immunofluorescence tests using the matching primary antibodies confirmed the reduction of the respective proteins, although to varying degrees (Figures S36–S41). Then, TMU of previously reported fluorescently labeled CAXs into the engineered cells was assessed. Three Fl-CAXs, Fl-AspA **10**, Fl-ETP **11**, and Fl-MAC **12** were chosen because our earlier studies established their orthogonal TMU pathways, thus the proteins identified by proteomics analyses of the first two CAXs are expected to contribute differently for the uptake of three probes.^11^ Fl-MAC **12** is an analog of dMAC inhibitor **7** and operates by tetrel-centered exchange cascades, as described above.^25^ TMU of probes into unmodified HK cells showed the established intracellular targeting (Figure 4A). For instance, Fl-AspA **10** labeled the Golgi,^49^ while Fl-ETP **11** accumulated in the nuclei.^54^ TMU of Fl-AspA **10** and Fl-ETP **11** into HK cells with reduced MFSD5 expression was strongly diminished, while that of Fl-MAC **12** was barely affected (Figure 4A). This difference in sensitivity is consistent with orthogonal TMU networks used by these probes.

**Figure 4:**
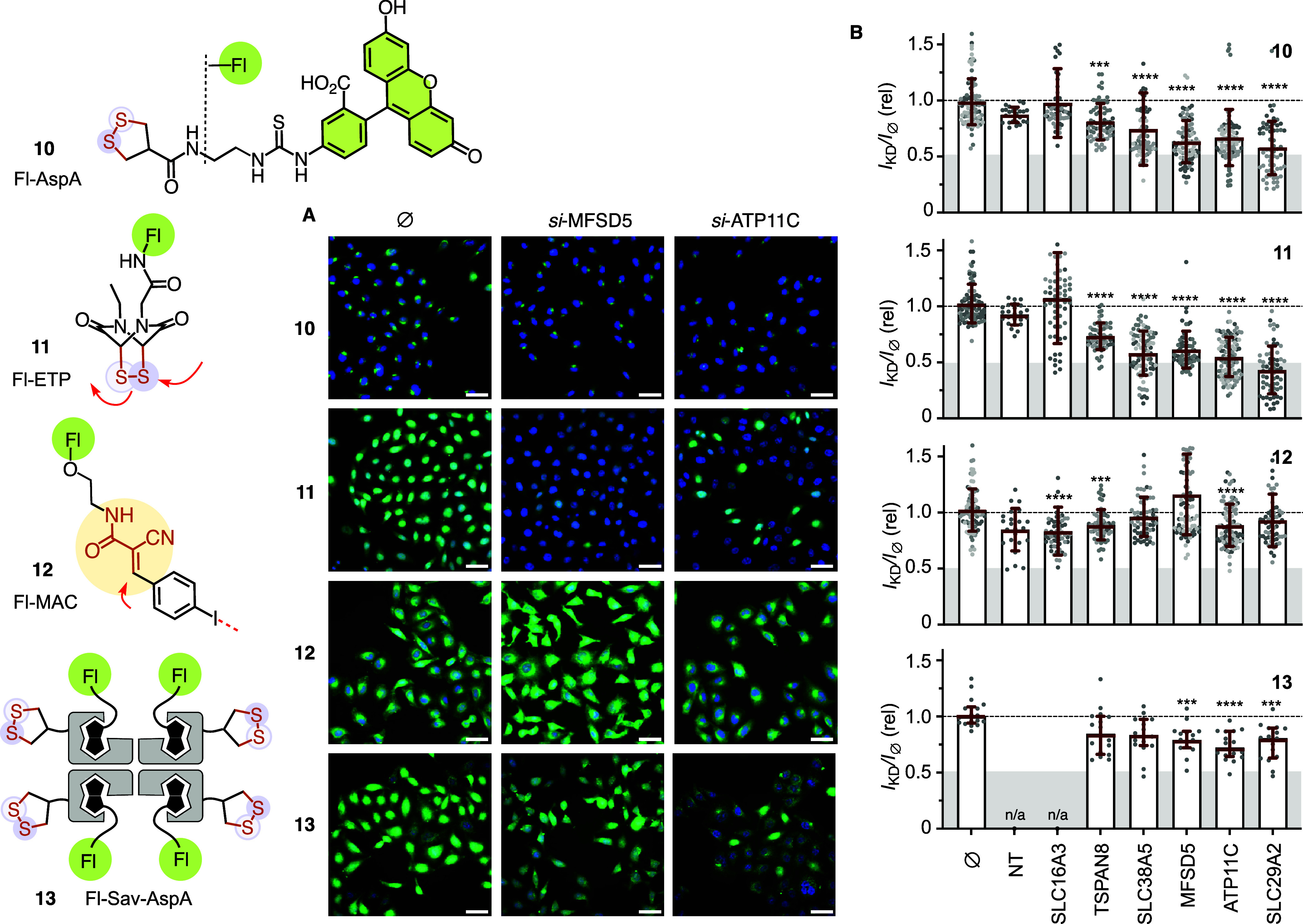
Partner validation. (A) Representative SDCM images of HK cells without (⌀, left) and with MFSD5 (middle) or ATP11C (right) siRNA treatment, incubated with Fl-AspA **10 (**10 μM, 0.5 h), Fl-ETP **11 (**5 μM, 0.5 h), Fl-MAC **12 (**10 μM, 0.5 h) and Fl-Sav-AspA **13** (10 μM, 2 h, all in Leibovitz’s L15 medium without serum,^11^ top to bottom, green), scale bar 50 μm, Hoechst 33342 (blue). (B) Average fluorescence (green) intensity in cell mask of knockdown cells relative to nontreated cells (gray filled circles, one per image) with **10**, **11**, **12**, and **13** (top to bottom; outlier data points >1.5 not shown). Global medians (red horizontal lines) ± interquartile range of experimental replicates (different shades of gray) with the results of nonparametric one-way ANOVA tests compared to data in nontreated (⌀) cells (*P* < 0.0001: ****, 0.0002: ***). NT: nontargeted.

Quantitative analysis of the images revealed that the knockdown of MFSD5 reduced the uptake of Fl-AspA **10** by about 40% (median 0.61, Figure 4B). This modest decrease is reasonable considering the involvement of multiple proteins in TMU. Thus, knocking down a single target cannot lead to complete inhibition. A similar effect of MFSD5 was observed for Fl-ETP **11** (0.55), even though this protein was not among the top hits in the μMap analysis of pc-ETP **4**. Fl-AspA **10** (0.64) and Fl-ETP **11** (0.60) are also highly dependent on ATP11C, which only appeared in the volcano plot with AspA **1** (Figures 4A,B, and 2E). Conversely, SLC29A2 was only enriched by ETP **4** and was more important for Fl-ETP **11** (0.43) than for Fl-AspA **10** (0.56), although widely scattered data (interquartile range ≈ 0.15) make its significance questionable. The other two proteins, SLC38A5 and TSPAN8, were found with ETP **4** and hAspA **5**, and were again slightly more important for the uptake of Fl-ETP **11** (0.57 and 0.73, respectively) than Fl-AspA **10** (0.63 and 0.78). It is worth noting that the apparent importance of TSPAN8 may be underestimated, as its knockdown was poorly effective according to the immunofluorescence test (Figure S40). Finally, the absence of SLC16A3 had only a marginal effect on the uptake of Fl-MAC **12** (0.80), but not **11** (1.1) or **10** (0.89). Given the independence from all other proteins (>0.86), Fl-MAC **12** uptake appears orthogonal to those of Fl-AspA **10** and Fl-ETP **11**, while the latter two seem to share some partners. Overall, partners detected only for AspA also affected ETP, while those detected only for ETP were less involved with AspA, a trend that agreed with ETP being less retained on thiol-exchange columns.^52^ Similar effects of knockdown on uptake were also found in retinal pigment epithelial-1 (RPE-1) and epidermoid carcinoma A431 cells (Figures S54–S56).

The involvement of these proteins in TMU of relatively large substrates was verified using a previously reported cell-penetrating streptavidin (Sav) **13** equipped with multiple AspAs and a fluorophore Fl (Scheme S1, Figures S12–S13).^55^ Orthogonal CLSM images reconstituted from z-stacks evinced that the evenly distributed fluorescence originates from cytosolic localizations (Figure S12), FLIM images confirmed that the fluorescence arises from intact protein conjugates (Figure S13), and uptake inhibition by the usual TMU probes supported that also the large AspA-protein conjugates enter cells by TMU (Figure S19). Consistent with the findings with small-molecule transporters above, TMU of Fl-Sav-AspA **13** was less efficient in siRNA-treated HK cells compared to the WT cells (Figures 4A,4B, S45, S49, S53). Whereas the effects were weaker compared to those on Fl-AspA **10** or Fl-ETP **11**, these results supported that the same set of proteins participate in the transmembrane transport of large substrates as well as the small ones.

## Discussion

The SLC superfamily contains at least 70 families with more than 446 proteins.^56–63^ This is one of the most prominent membrane protein families in the human genome. MFSs are a subfamily of SLCs, and MFSD5 belongs to SLC61, i.e., molybdate transporters.^62,61^ Beyond SLC61, observed were members of SLC1–7, 16, 19, 22, 29, 30, 35, 38, 39, 43, 44, 46, 52, and O3,^56^ which are known to transport amino acids (1, 3, 6, 7, 38, 43), other monocarboxylates (4, 16) and organic ions (22, O3), nucleosides (29, 35), glucose (2, 5), folate/thiamine/choline (19, 44, 46), zinc and other metal ions (30, 39), and riboflavin (52) into cells. While many SLCs are orphaned without known substrates, they are also found to transport various cytotoxic drugs.^59^ SLCs are comparably small transmembrane helix bundles with 0–35 but mostly 6–16 cysteines (Figure 2C).^60^ Many of these cysteines are located within the hydrophobic part of the membrane, which is unusual for membrane proteins and interesting for TMU (Figure 2C). Reactivities of these cysteines to “warheads” were previously demonstrated by chemoproteomics analyses.^64^

With regard to TMU, it is intriguing that significant conformational changes of SLCs are necessary for substrate transport, whether by so-called rocker-switch, rocking-bundle, or elevator mechanisms (Figure 5).^62^ Local structural reorganization during transport, including protein elevators and membrane deformations like toroidal elastics^4,49^ or endolipoplexing,^7^ is generally considered important for TMU because, otherwise, compatibility with larger substrates is hardly conceivable. Information on larger substrates for SLCs is rare, but their involvement in the viral entry has been confirmed,^36,65^ and MFSD5 has been reported to account for the uptake of lipoprotein­(a), but not of LDL.^66^ Lp­(a) is a nanoparticle of 25 nm diameter composed of proteins, lipids and “bad” cholesterol. The identification of this MFSD5 as one of the important exchange partners in TMU was thus very meaningful.

**Figure 5:**
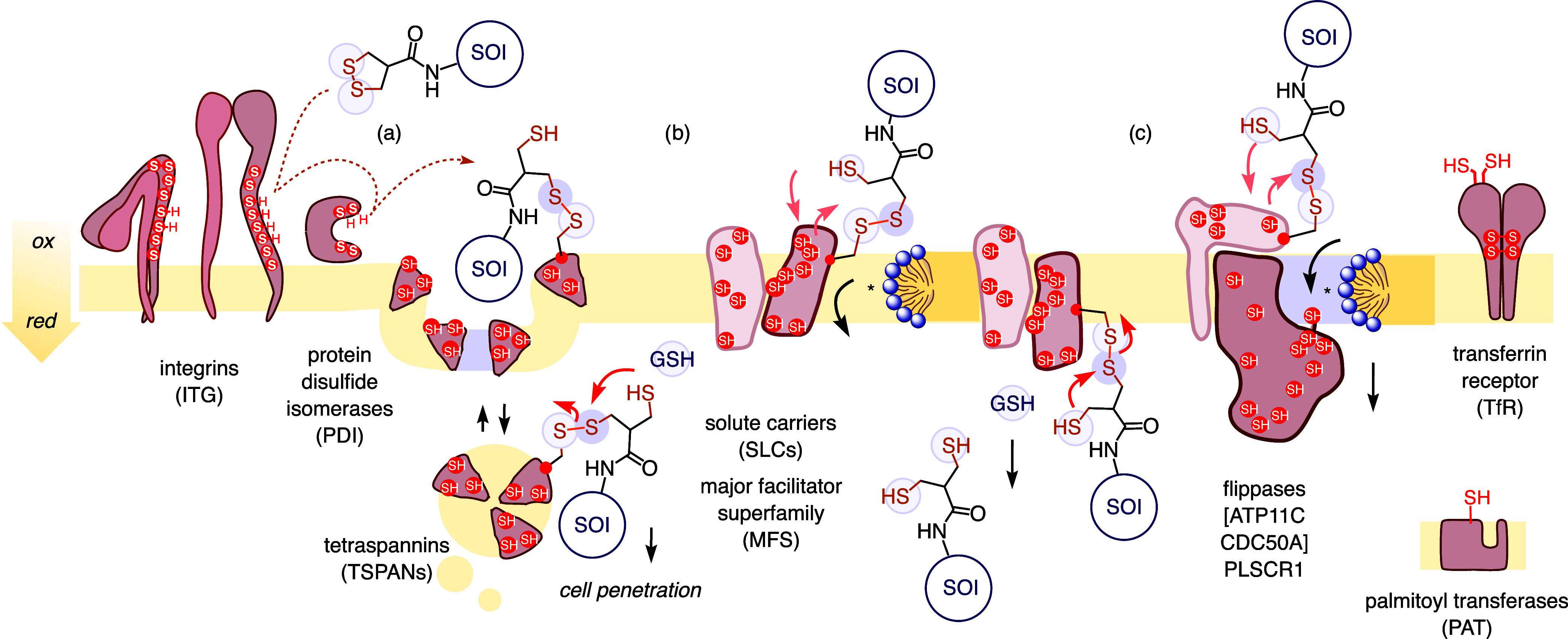
Nature of thiol-mediated uptake. μMap proteomics and KD validation identify SLC-MFS, flippases, and TSPANs as primary cascade exchange partners, possibly enabling TMU by combining (b) elevator mechanisms with toroidal elastics (*), (c) intrinsic membrane flip-flop activity and (a) endolipoplexing. The previously identified TfR (AspA) is confirmed as an exchange partner, candidates like integrins or PDIs are supported, and PATs, as confirmed final internal exchange partners of AspA, are added for completeness. Solid black arrows: Direction of TMU; solid red arrows: Selected disulfide exchange; dotted arrows: Representative multiprotein exchange cascade. SOI = substrate of interest.

Among the many SLCs detected, SLC38A5 and SLC29A2 were corroborated as partners of ETP by knockdown experiments, while SLC16A3 did not seem to be involved (Figure 4B). These results suggested that not all dynamic exchanges between proteins and CAXs are productive in promoting the uptake. SLC16A3 might be locked in an inactive conformation upon reacting with CAXs, similar to the action of a cysteine-reactive organomercury agent pCMBS.^67^

Considering the apparent need for local membrane deformations like toroidal elastics^4,49^ or endolipoplexing^7^ to deliver large substrates by TMU, the emergence of flippases/scramblases was noteworthy. ATP11C forms a heterodimer with CDC50A (aka TMEM30A, Figure 2C).^68^ Both are rich in cysteines. Particularly interesting for TMU are C306/883/895/914 of ATP11C near the hydrophilic groove of the lipid passageway (Figure 2C, magenta dashed square). Other detected scramblases, ANO6 (anoctamine-6, aka TMEM16F)^69–71^ and phospholipid scramblase 1 (PLSCR1),^72,73^ are also promising potential TMU partners, known to cause membrane deformation, and equipped with cysteine residues in their putative transmembrane regions.

Tetraspanins (TSPANs) are another family of proteins that is attractive as potential exchange partners in TMU.^74^ TSPAN8 consists of 237 amino acids, featuring four putative transmembrane helices and 12 cysteines, many located near the interfaces but also within the transmembrane region (Figure 2C). Their primary function again involves membrane reorganization, especially membrane deformation, related to their inverted cone shape.^74^ They can assemble into TSPAN-enriched microdomains (TEMs or TERMs), increase membrane curvature, promote fusion, participate in clathrin-independent endocytosis and viral entry, appear in exosomes, facilitate membrane damage repair, and more.^74^ In the context of TMU, the properties of TSPANs could thus convincingly explain how the exchange with thiols at the cell surface could also trigger the delivery of large substrates into the cytosol (Figure 5).

Most of the previously identified partner proteins were confirmed by this study. Namely, transferrin receptor, identified by classical chemoproteomics study for AspA and validated by knockdown,^26^ was confirmed as one of the exchange partners of both AspA **1** and **5** (Figure 2). Integrins were earlier proven to be involved in the uptake of ETP by knockdown (Figure 4).^11^ Some of the more than 20 existing ITGs appeared among the top hits in the volcano plots, not only with pc-ETP **4** but also with pc-AspA **1** and **5**. Other exchange candidates from previous studies were supported as well by μMap proteomics, like PDIs,^11^ SCARB1,^26^ HSA^49^ or EGFR^75^ (Figures 2B, and S29–S34). Many other detected candidates, e.g., IFITMs, recognized for their roles in uptake elsewhere,^76^ were not further considered because their structure made direct involvement in TMU unlikely. STRING analysis^77^ of the identified TMU exchangers revealed that only a few networked proteins were enriched by μMap proximity labeling, such as partners of flippases (ATP11C/TMEM30A) and a few SLCs. These results corroborated the very short effective energy transfer distance and, thus, the selective labeling of the target proteins by the photocatalytic μMap strategy (Figure S35).

Palmitoyl transferases (PATs) in the Golgi as confirmed final partners, were not among the top hits for AspA, suggesting that the contact time of AspA with PATs for double palmitoylation is too short, while the permanent residence of AspA in the Golgi is unrelated to proximity with PATs.^49^

The undetectability of this enzyme in the AspA proteomics exemplified the intrinsic challenge of identifying TMU exchange partners outlined in the introduction. Analogous to the coupled fast off-equilibrium exchange cascades underlying the molecular relay in TMU, efficient catalysis requires strong transition-state interactions but only weak ground-state interactions and high off-rates to ensure turnover. However, proteomics, in general, more efficiently capture the ground-state binders with low off-rates, such as receptors or repressors, rather than the labile binders that account for function. Although photocatalytic proximity labeling proteomics was expected to improve the detectability of the latter, the obtained results confirm the obvious that (i) not all exchange partners will be detected under any conditions and (ii) most of the detected proteins will not participate in TMU, which calls for validation of every meaningful candidate by knockdown. Considering this challenge to elucidate the molecular relays underlying TMU, the results of μMap proteomics analyses are remarkably consistent with our previous findings and proposed TMU mechanism (Figure 5). Namely, SOI-CAX conjugates bind to cysteine-rich cell surface proteins (ITG, EGFR, etc.) through dynamic covalent bonds and continue exchanging with other cysteine-containing nearby proteins until they reach toroidal elastics or related deformations,^4,7,49^ probably made with SLCs, flippases or TSPANs, to cross the plasma membranes and enter the cytosol.

## Conclusions

Thiol-mediated uptake (TMU) refers to the appearance of cell-penetrating activity in the presence of a motif capable of reversible multiple dynamic covalent exchange with cellular thiols and disulfides. Although TMU is slowly emerging as an important strategy for delivering substrates of interest into cells, it remains poorly understood and, therefore, underused. This poor mechanistic understanding originates not from a lack of interest but from the complexity of the dynamic covalent cascade exchange networks, presumably underlying TMU. The challenge is to identify not the strong and inert binders but cellular protein partners in coupled dynamic covalent exchange processes without perturbing the off-equilibrium relay network they are part of. This study elaborates on the expectation that recently developed photocatalytic proximity labeling methods could be ideal for this task.

Photocatalytic microenvironment mapping proteomics confirms TMU as a process of exceptional complexity that operates with dynamic covalent networks with varied selectivities. Solute carriers (SLCs), flippases/scramblases and tetraspanins (TSPANs) emerge as key exchange partners. Validation by genetic knockdown particularly emphasizes ATP11C and MFSD5, as well as SLC38A5. Their identification is rewarding because their canonical mode of action already involves local membrane and protein reorganization, which is necessary for TMU to transport large substrates of interest across the plasma membrane (Figure 5). Other exchange partners are confirmed (TfR,^26^ ITG,^11^ PDI,^11^ HSA^49^) or suspected to contribute.

The intrinsic challenge of elucidating cascade exchange networks without disturbing their flow implies that not all exchange partners will be detected under any conditions and that many of the detected proteins will not participate in TMU. While this demands the knockdown of every meaningful candidate to validate, single protein knockdown will reduce but never completely shut down complex relay networks. Continuing studies with different cascade exchangers^11,78^ and possibly improved photocatalysts^21^ will thus be of high interest to expand partner identification and understanding of TMU. TMU networks will naturally change with proteins available in different cells: TMU has been reported to occur also in plants^17^ or bacteria.^79^

Beyond the identification of individual cellular exchange partners involved, this study provides a new understanding of the nature of TMU. This insight, with all its complexity, could well reflect the truth as close as possible today, could therefore last, apply more generally, also to the entry of certain pathogens, and inspire new research directions to attain control over cell penetration, also in practice.

## Supplementary Material



## Data Availability

The data that support the findings of this study are openly available in Zenodo at https://doi.org/10.5281/zenodo.15387120.

## Notes

Detailed experimental procedures; materials and methods; compound synthesis and characterization; AHCHT procedures for cellular uptake and inhibition; photoproteomics procedures; photoproteomics data analysis; siRNA knockdown procedures (PDF)
