# Supplementary material for: Photocatalytic Microenvironment Proteomics of Thiol-Mediated Uptake
Source: JACS Au. 2025 Jul 1;5(7):3288–98. doi: 10.1021/jacsau.5c00432 (PMC12308380; doi:10.1021/jacsau.5c00432)
Supplement: Supplementary file 1 [file au5c00432_si_001.pdf]

Supporting Information

Photocatalytic Microenvironment Mapping of Thiol-Mediated Uptake

Saidbakhrom Saidjalolov,[§,‡] Yibo Wu,[¶] Giacomo Renno,[§,‡] Nicholas Rose,[§,‡] Jelena Gajić,[§] Bertrand Pologne,[§] Nicolas Winssinger,[§] Vincent Mercier,[†] Dimitri Moreau,[†] Naomi Sakai*,[§,‡] and Stefan Matile*,[§,‡]

[§]Department of Organic Chemistry, University of Geneva, 1211 Geneva, Switzerland

[‡]National Centre of Competence in Research (NCCR) Molecular Systems Engineering, BPR 1095, 4002, Basel, Switzerland

[¶]ChemBioMS Proteomics Platform, University of Geneva, 1211 Geneva, Switzerland

[†]ACCESS Platform, University of Geneva, 1211 Geneva, Switzerland

*E-mail: naomi.sakai@unige.ch, stefan.matile@unige.ch

Table of Contents

1.	Materials and Methods	S4
2.	Photoreactor	S7
3.	Synthesis	S8
	3.1. TMU Compounds	S8
	3.2. Preparation of Fl-Sav-AspA	S9
	3.3. Synthesis of Labeling Probes	S12
4.	TNBSA Assay	S29
5.	Stability in Aqueous Medium	S30
6.	Spectroscopic Properties	S31
7.	Cell Culture	S32
8.	Lysate Preparation	S33
9.	Western Blot Analyses	S33
10.	Cellular Uptake of PCs	S34
	10.1. General Experimental Procedure	S34
	10.2. Data Analysis	S35
	10.3. Optimization of Uptake	S36
	10.3.1. Cellular Uptake Pattern	S36
	10.3.2. Results for Uptake of pc-CAXs in HK Cells	S38
	10.4. Cellular Uptake of Fl-Sav-AspA	S39
	10.5. Cytotoxicity	S41
11.	AHCHT TMU Inhibitor Screening	S41
	11.1. General Experimental Procedure	S41
	11.2. Data Analysis	S42
	11.3. Results for Inhibitors Screening in HK Cells	S43

11.4. Inhibition of Fl-SAV-AspA with TMU Inhibitors S51

12. Photocatalysts Reactivity Assessment S52

12.1. In Organic Solvent S52

12.2. Protein Labeling S56

12.3. In Cells S57

13. Proteomics S60

13.1. General Procedure S60

13.2. Data Acquisition S62

13.3. Data Analysis S63

14. Protein Knockdown S71

14.1. Level of Expression S71

14.2. Knockdown Procedure S72

14.3. Quantification of Protein Knockdown by Immunofluorescence S72

14.4. Cellular Uptake in Knocked-Down Cells S76

15. Supporting References S92

16. NMR Spectra S95

1. Materials and Methods

As described in S1, reagents for synthesis were purchased from Merck, Sigma-Aldrich, TCI, Broadpharm, and Fluorochem. High-quality salts from Fluka or Sigma-Aldrich were used without further purification. Complex **24** was purchased from Sigma-Aldrich (747793). The following media and solutions were obtained from Thermo Fisher Scientific: phosphate-buffered saline (PBS, pH 7.4), Dulbecco's PBS (calcium/magnesium-free, DPBS, 14190094), DMEM (11965092), FluoroBrite DMEM (GlutaMAX, 4.5 g/L D-glucose, pyruvate, without phenol red, A1896701), Opti-MEM (11058021), and Leibovitz's L15 (without phenol red, 21083027). Other reagents and consumables from the same supplier included penicillin-streptomycin, fetal calf serum, TrypLE Express enzyme, V96-MicroWell plates, Nunclon™ Sphera™ U-bottom 96-well sterile plates, SYTO™ deep red (S34900), Hoechst 33342 (HOE, H3570), propidium iodide (PI, P3566), streptavidin Alexa Fluor® 680 conjugate (S21378), Dynabeads M-280 streptavidin (11206D), and Lipofectamine RNAiMAX (13778075). siRNA solutions for protein knockdown were purchased from siTOOLs Biotech. µ-Plate 96-Well Black plates were obtained from Ibidi. Primary antibodies against SLC38A5 (ab317685), SLC29A2 (ab181192), SLC16A3 (ab308528), ATP11C (ab262923) and TSPAN8 (ab70007) were purchased from Abcam. Primary antibody against MFSD5 (HPA039773) was purchased from Sigma-Aldrich. Fluorescent conjugate secondary antibody (Alexa Fluor® 647 (711-605-152), AffiniPure Donkey Anti-Rabbit IgG (H+L)) was purchased from Jackson ImmunoResearch. Analytical thin-layer chromatography (TLC) was conducted on silica gel 60 F254 (Merck, 0.2 mm), visualized under UV light at 254 nm. Column chromatography was performed on silica gel 60 (SilicaFlash® P60, SILICYCLE, 230-400 mesh), and flash chromatography was done using a Biotage Isolera^{TM} Spektra or Selekt system with pre-packed Scorpius cartridges (BGB). Melting points (Mp) were recorded using a Melting Point M-565 (BUCHI). Alpha-D values were measured with a Polarimeter P-1030 (Jasco). IR spectra were obtained using a Perkin Elmer Spectrum Two™ FT-IR spectrometer (ATR, Golden Gate), reported as wavenumbers (v) in cm^{-1} with intensities described as broad (br), strong

(s), medium (m), or weak (w). ^{1}H, ^{13}C, and ^{19}F NMR spectra were recorded on Bruker 300, 400, or 500 MHz spectrometers at 25°C, with chemical shifts (δ) in parts per million (ppm) referenced to residual solvent peaks (DMSO-d_6: 2.50/39.5 ppm; CD_3OD: 3.31/49.0 ppm; $CDCl_3$: 7.26/77.2 ppm). Spin multiplicities were reported as singlet (s), doublet (d), triplet (t), or multiplet (m), with coupling constants (J) in Hz. ^{1}H and ^{13}C assignments were supported by 1D and 2D NMR spectra (^{1}H-^{1}H COSY, DEPT 135, HSQC, HMBC). ESI-HRMS was performed on a Xevo G2-S TOF (Waters). LC-MS (low resolution) analysis was conducted on an Advion Avant® UHPLC system with a Thermo C18 Hypersil GOLD column (50 x 2.1 mm, 1.9 µm, 0.75 mL/min, gradient elution of H_2O + 0.01% TFA / CH_3CN + 0.01% TFA from 3:7 to 0:1 in 4.0 min) and an Advion Expression® CMS in ESI mode. All mass data are presented as mass-to-charge ratios (*m/z*). UV-Vis spectra were recorded on a JASCO V-650 spectrophotometer with a stirrer and temperature controller (20 °C), with maximal absorption wavelengths (λ) in nm and extinction coefficients (ε) in M^{-1} cm^{-1}. Fluorescence spectra were collected on a FluoroMax-4 (Horiba Scientific), with correction factors applied. Proteome analysis was performed on an Easy Nano LC - Orbitrap Fusion System equipped with a nanospray flex™ ion source (Thermo Fisher Scientific, USA). Confocal and widefield cell imaging were performed using an IXM-C automated microscope (ImageXpress), equipped with a Lumencor Aura III light source and bandpass filters, with 5 objectives (4x to 60x). Washing steps were carried out using a Biotek EL406 plate washer. Confocal laser scanning and fluorescence lifetime imaging was performed on Leica Stellaris FALCON equipped with 60x oil immersion objective.

Abbreviations: AHCHT: Automated high-content high-throughput; AsC: 2-(4-Aminophenyl)-1,3,2-dithiarsinane-5-carboxylic acid; AspA: Asparagusic acid; BBTA: 2-(4-((Bis((1-(tert-butyl)-1H-1,2,3-triazol-4-yl)methyl)amino)methyl)-1H-1,2,3-triazol-1-yl)acetic acid; BCA: Bicinchoninic acid; BiC: 2-Chloro-1,3,2-dithiabismepane-5,6-diol; BSA: Bovine serum albumin; CAX: Covalent exchangers; CLSM: Confocal laser scanning microscopy; CPS: Cell-penetrating streptavidin; DET: Dexter energy transfer; DIPEA: *N*,*N*-Diisopropylethylamine; dMAC: Double Michael acceptor;

DMEM: Dulbecco's modified eagle medium; DMF: *N*,*N*-dimethylformamide; DMSO: Dimethyl sulfoxide; DPBS: Dulbecco's phosphate-buffered saline; DTNB: (5,5′-Dithiobis-(2-nitrobenzoic acid); DTT: Dithiothreitol; EBS: Ebselen analogue; EBX: Ethynylbenziodoxolone; EDC: *N*-(3-Dimethylaminopropyl)-*N*′-ethylcarbodiimide; EtOAc: Ethyl acetate; ETP: Epidithiodiketopiperazine; FA: Formic acid; FCS: Fetal calf serum; FDMEM: FluoroBrite DMEM; FITC: Fluorescein isothiocyanate; Fl-: Fluorescein; FLIM: Fluorescent lifetime imaging; HATU: Hexafluorophosphate azabenzotriazole tetramethyl uronium; HeLa: Henrietta Lacks; HK: HeLa Kyoto; HPLC: High-Performance Liquid Chromatography; HRMS: High resolution mass spectra; IC_{50}: Half maximal inhibitory concentration; KD: Knockdown; LRMS: Low resolution mass spectra; MIC: Minimum inhibitory concentration; NHS: *N*-Hydroxysuccinimide; NT: Non-treated or non-target; PBS: Phosphate-buffered saline; pc: Photocatalyst; PE: Pentane; PFA: Paraformaldehyde; PI: Propidium iodide; PS: Penicillin-streptomycin; RIPA buffer: Radioimmunoprecipitation assay buffer; RP: reverse-phase; RT: Room temperature; RV: Relative viability; Sav: Streptavidin; SAV680: Streptavidin-AlexaFluor680; SD: Standard deviation; SDCM: Spinning disk confocal microscopy; SEM: Standard error of mean; siRNA: Small interfering RNA; TBTA: Tris(benzyltriazolylmethyl)-amine; TCEP: Tris(2-carboxyethyl)phosphine; TEA: Triethylamine; TFA: Trifluoroacetic acid; THF: Tetrahydrofuran; THPP: Tris(hydroxypropyl)phosphine; THTPA: Tris(hydroxypropyltriazolyl-methyl)amine; TLC: Thin layer chromatography; TMU: Thiol-mediated uptake; TNBSA: 2,4,6-Trinitrobenzenesulfonic acid; TRIS buffer: Tris-(hydroxymethyl)aminoethane buffer; WB: Western blot; WI: Water immersion; WT: Wild type.

2. Photoreactor

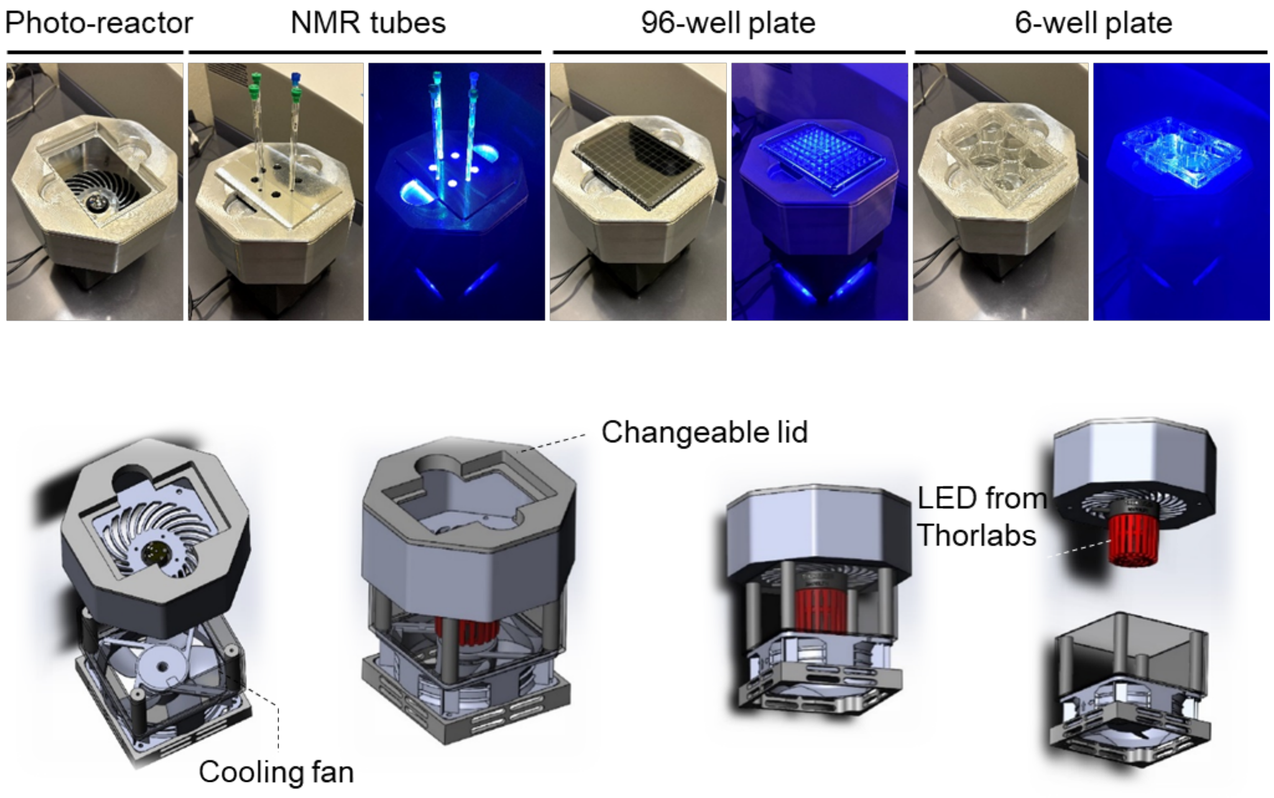

Figure S1. Photographs of a homemade photoreactor, inspired by the Wisconsin photoreactor platform (WPP) design,[S2] featuring a customizable light source, an adaptable lid, and an air ventilation system. The light source uses LEDs purchased from Thorlabs, specifically the Royal Blue M450LP2 (450 nm). These LEDs have a maximum power output of approximately 3 W at the maximum current of 2000 mA. The maximum irradiance is 34.2 μW/mm² when the LEDs are operating at 700 mA, with a distance of 200 mm from the light source, as provided by the supplier.

3. Synthesis

3.1. TMU Compounds

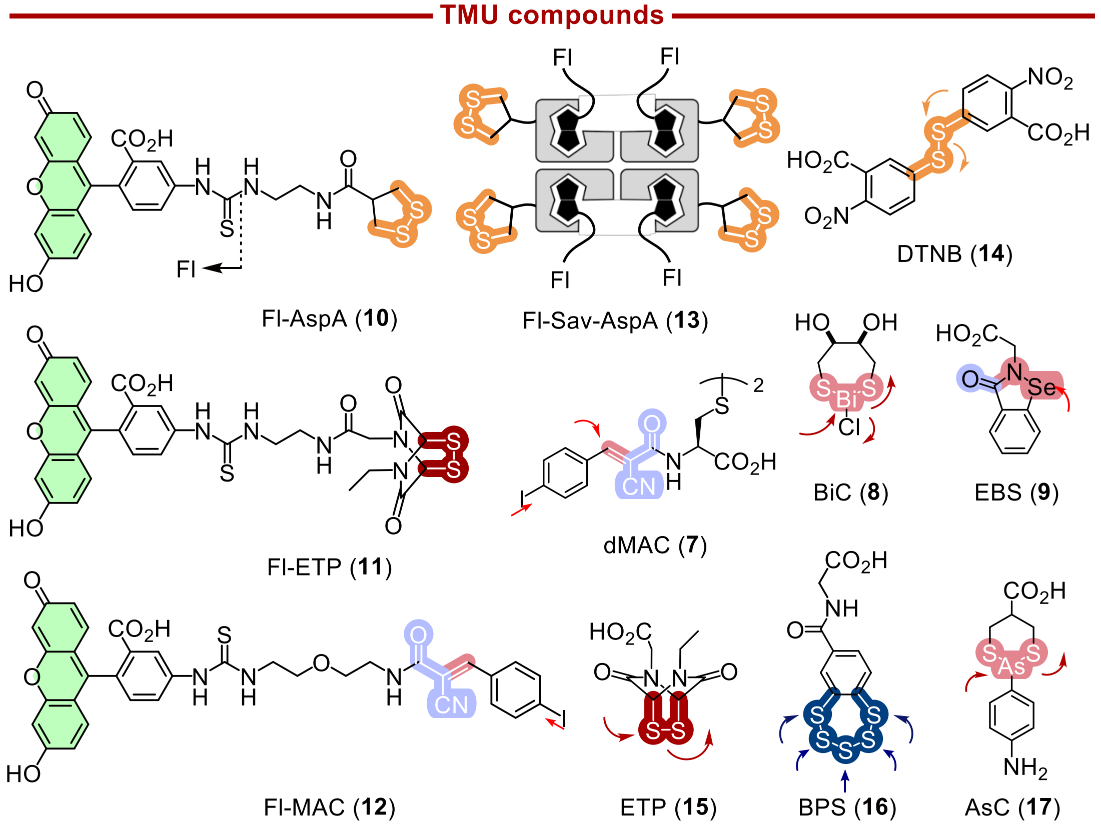

Figure S2. Structure of synthesized TMU compounds used in this study.

7 and **12** were synthesized according to procedures described in reference S3.

8 and **17** were synthesized according to procedures described in reference S4.

9 was synthesized according to procedures described in references S5.

10 was synthesized according to procedures described in reference S6.

11 and **15** were synthesized according to procedures described in reference S7.

16 was synthesized according to procedure described in reference S8.

3.2. Preparation of Fl-Sav-AspA

The Cell-Penetrating Sav (CPS) was prepared following a slightly modified procedure described in reference S9.

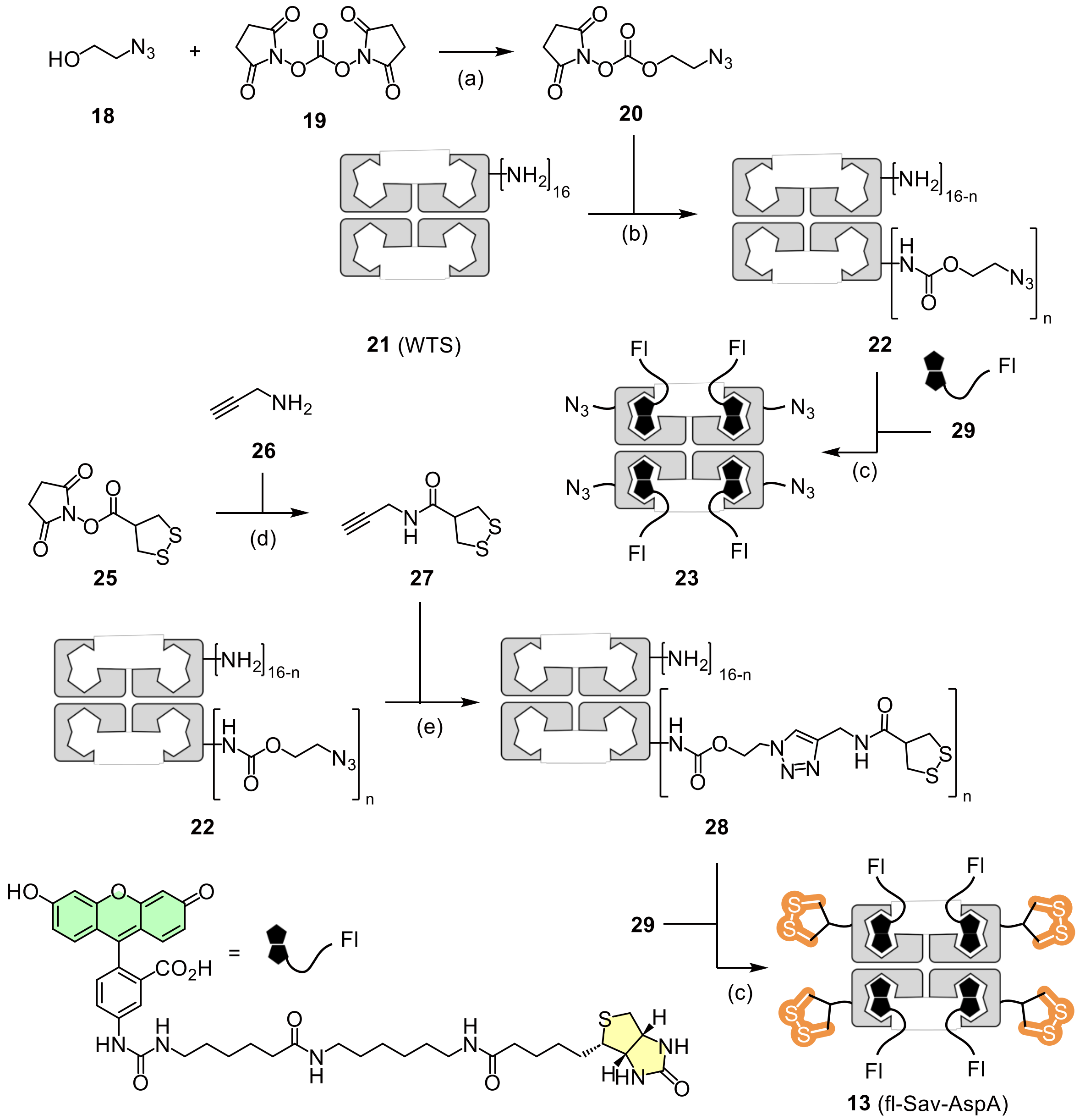

Scheme S1. (a) Pyridine, CH_2Cl_2, RT, 4 h, 61%; (b) **20**, PBS, 25 °C, 3 h; (c) **29**, bidistilled H_2O, 25 °C, 10 min; (d) **26**, DIPEA, CH_2Cl_2, RT, 2 h, 79%; (e) **27**, $CuSO_4$, aminoguanidine hydrochloride, sodium ascorbate, BTTAA, PBS, 25 °C, 1 h.

Compound 20. Pyridine (557 µL, 6.89 mmol) was added to a stirred solution of **18** (200 mg, 2.30 mmol) and **19** (480 µL, 2.30 mmol) in CH_2Cl_2 (20 mL), which was stirred at RT for 4 h. The

reaction mixture was diluted with further CH_2Cl_2 (30 mL) and washed with 0.2 M HCl (20 mL) and brine (20 mL). The organic layer was then dried over Na_2SO_4, filtered and concentrated *in vacuo* to give a yellow oil. The crude residue was subjected to purification by flash chromatography (Biotage, Sfar cartridge, 12 g, pentane/EtOAc 9:1) to afford **20** (318 mg, 61%) as a yellow oil. R_f (pentane/EtOAc 9:1): 0.3; IR (neat): 2951 (w, C-H), 2103 (m, N_3), 1787 (m), 1732 (s, C=O), 1355 (w), 1194 (s, C-O-C), 1090 (m), 716 (w), 642 (m); ^{1}H NMR (400 MHz, $CDCl_3$): 4.47 – 4.42 (m, 2H), 3.64 – 3.58 (m, 2H), 2.84 (s, 4H); ^{13}C NMR (101 MHz, $CDCl_3$): 168.5 (2C), 151.5 (C), 69.3 (CH_2), 49.4 (CH_2), 25.6 ($2CH_2$).

Compound 22. As in reference S9, to a solution of **21** (Sav, WT) in PBS (365 µL, 20 µM), **20** was added (34 µL of a 35 mM stock solution in DMSO, final concentration 3.0 mM). The mixture was shaken at 25 °C for 2 h, then filtered through Amicon® Ultra 0.5 mL centrifugal filters (cut-off: 3 kDa, 14.5 krpm, 10 min) and washed twice with a bicarbonate buffer solution (0.1 M, pH 8.5, 2 × 0.4 mL) and filtered again (cut-off: 3 kDa, 14.5 krpm, 10 min). The material was recovered from the centrifugal filter, and the concentration of **22** (61 µM) was determined by UV spectroscopy (Figure S4a). TNBSA assay was used according to the procedure in refenrece S9 to determine the degree of functionalization, $n = 13.0 \pm 0.3$, out of 16 lysine residues per Sav tetramer (Figure S4b).

Preparation of complex 23. To a solution of **22** in H_2O (14.5 µM, 200 µL), **29** (0.30 µL of a 48 mM stock solution in DMSO) was added, and the mixture was incubated at 25 °C for 10 min. Afterward, the mixture was filtered through Amicon® Ultra 0.5 mL centrifugal filters (cut-off: 3 kDa, 14.5 krpm, 10 min). The stock solution, whose concentration was calculated assuming full recovery of the protein, was then diluted with the medium to give the desired concentration for the uptake experiments.

Compound 28. To a solution of **22** in PBS buffer (12.5 µM, 290 µL), **27** (5.5 µL of a 20 mM stock solution in DMSO) was added. In a separate Eppendorf tube, a click-reagents solution was prepared by mixing $CuSO_4$ (7.4 µL of a 10 mM stock solution in H_2O), Na ascorbate (7.4 µL of a

150 mM stock solution in H_2O), guanidine HCl (7.4 μL of a 75 mM stock solution in H_2O), BTTAA (7.4 μL of a 150 mM stock solution in H_2O) and H_2O (40 μL). The resulting mixture was added to the solution of **22** and **27** and stirred at 25 °C for 1 h. During this time, the same quantities of click-reagent mixture were freshly prepared and added at $t = 20$ min and $t = 40$ min. Afterward, the mixture was transferred and filtered through Amicon® Ultra 0.5 mL centrifugal filter (cut-off: 3 kDa, 14.5 krpm, 10 min), and washed with PBS using the same cut-off filter (5 × 0.4 mL, cut-off: 3 kDa, 14.5 krpm, 10 min). Based on several experiments and on data reported in reference S9, a loss of 20% of the material during the washing cycles was considered when calculating the final concentration of **28**.

Preparation of complex 13. To a solution of **28** in H_2O (14.5 μM, 200 μL), **29** (0.30 μL of a 48 mM stock solution in DMSO) was added, and the mixture was incubated at 25 °C for 10 min. Afterward, the mixture was filtered through Amicon® Ultra 0.5 mL centrifugal filters (cut-off: 3 kDa, 14.5 krpm, 10 min). The stock solution, whose concentration was calculated assuming full recovery of the protein, was then diluted with the medium to give the desired concentration for the uptake experiments.

3.3. Synthesis of Labeling Probes

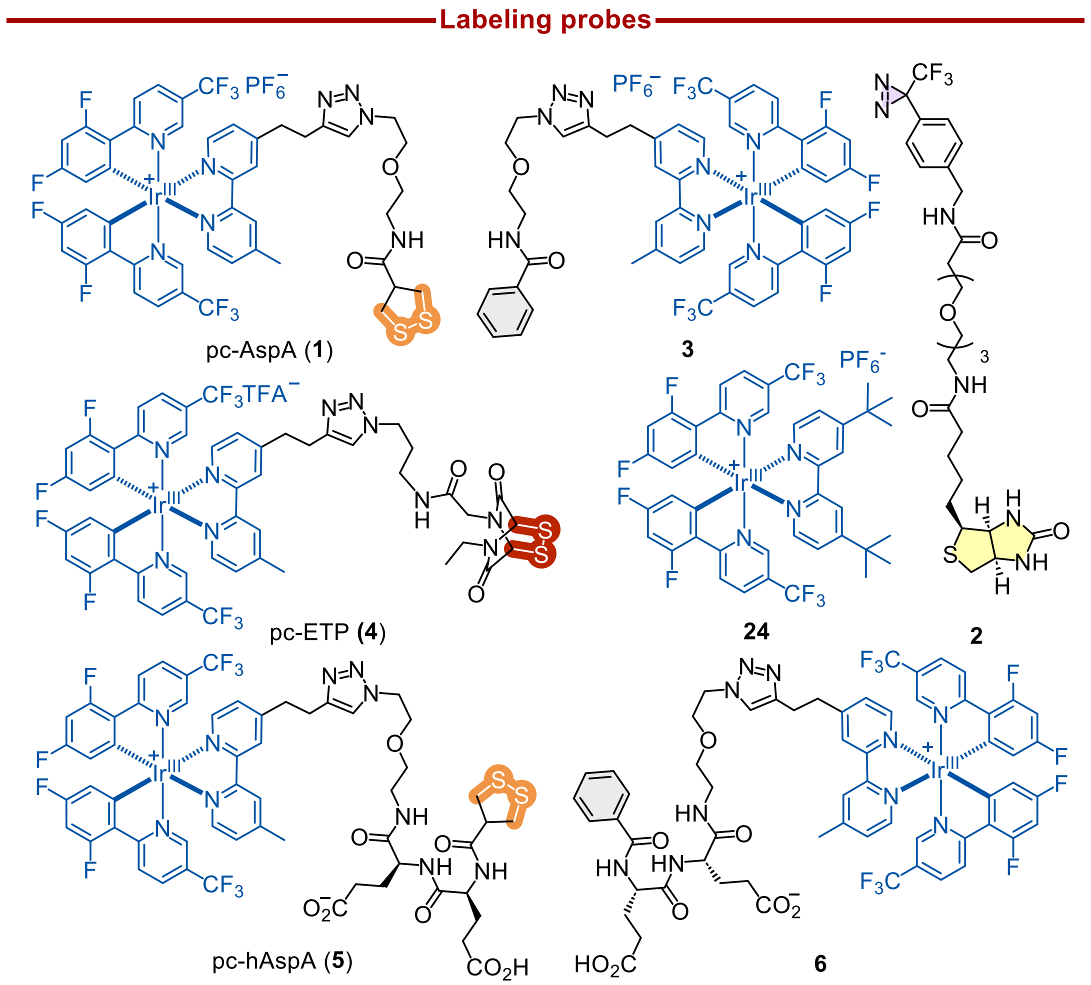

Figure S3. Structure of photocatalysts (PCs) and biotinylated diazirine used in this study.

General procedure for CuAAC coupling. To a mixture of alkyne (1.0 equiv.) and azide (1.2 equiv.) in dry THF under N_2 atmosphere was added a pre-mixed solution of CuI (2.0 equiv.) and TBTA (0.5 equiv.) in THF (final concentration of alkyne 5 mM). The reaction mixture was stirred at RT and monitored by LC-MS. After completion of reaction (6 – 96 hours), the crude mixture was filtered to remove the excess of CuI and then the filtrate was concentrated under reduced pressure. The crude solid was subjected to purification by flash chromatography to afford the desired triazole product.

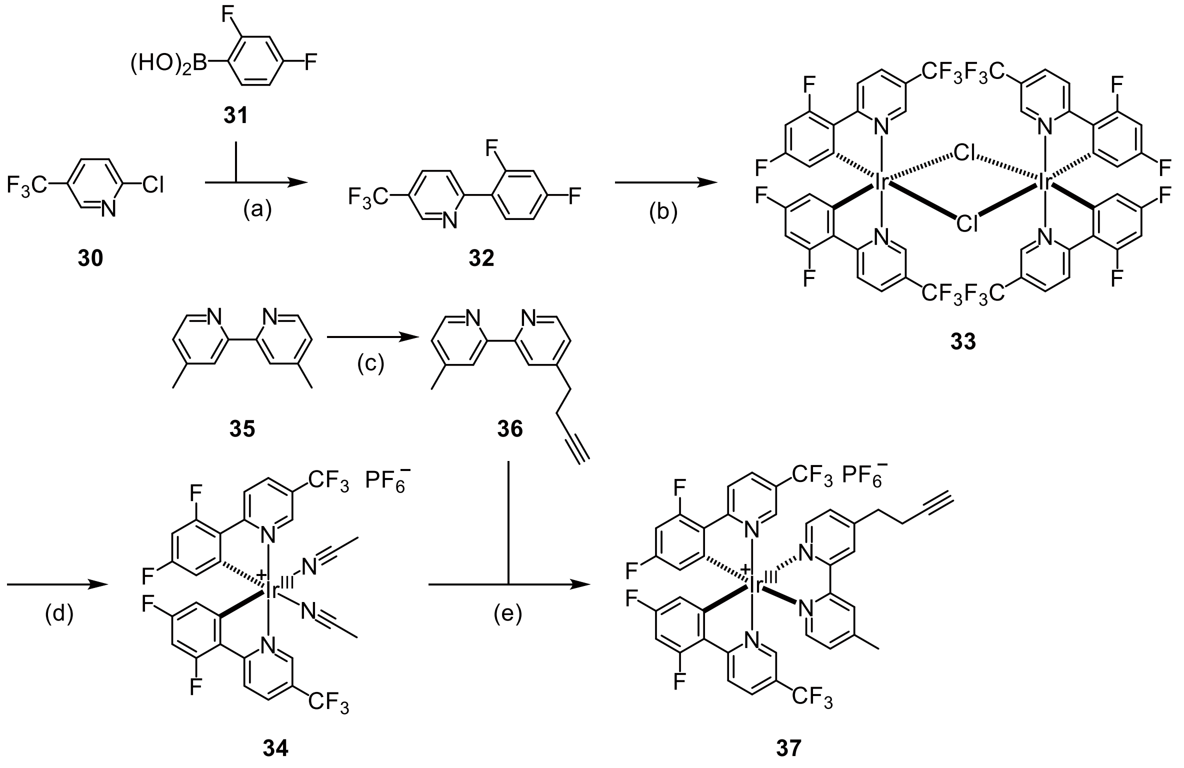

Scheme S2. (a) XPhos Pd G3, K_3PO_4, THF/H_2O 1:2, 40 °C, 4 h, 95%; (b) $IrCl_3.H_2O$, 2-methoxyethanol/H_2O 2:1, 120 °C, 18 h, 78%; (c) LDA, propargyl bromide, THF, 0 °C, 1.5 h, 90%; (d) $AgPF_6$, CH_2Cl_2/CH_3CN 5:1, 40 °C, 20 h, 98%; (e) CH_2Cl_2/EtOH 3:1, 30 °C, 16 h, 75%.

Compounds 32-34 were synthesized following the procedure described in reference S10.

Compound 36. To a solution of **35** (250 mg, 1.40 mmol) in dry THF (10 mL) at -78 °C was added dropwise a solution of LDA (2.0 M in THF, 880 µL, 1.80 mmol). The reaction mixture was stirred for 30 min at 0 °C. A solution of propargyl bromide (80% in THF, 220 µL, 2.00 mmol) was added rapidly to the reaction mixture which resulted in a change of color. The reaction mixture was allowed to stir for additional 1 h followed by quenching with water (30 mL). The crude product was extracted with Et_2O (3 x 20 mL). The combined organic layers were washed with brine, dried over Na_2SO_4, and concentrated *in vacuo* to afford a brown oil. The crude oil was subjected to purification by flash chromatography (Scorpius silica, 40 g, CH_2Cl_2/MeOH + 10% NH_4OH 97:3) to afford **36** (272 mg, 90%) as brown crystals. The spectroscopic data were in accordance with the ones reported in reference S11.

Compound 37. To a round-bottomed flask containing **36** (54 mg, 240 μmol) and compound **34** (150 mg, 160 μmol) was added CH_2Cl_2/EtOH 3:1 (10 mL). The reaction mixture was purged with N_2 for 10 min and left stirring at 30 °C for 16 h in the dark. After completion of the reaction (monitored by TLC), the resulting mixture was concentrated *in vacuo* and the crude solid was subjected to purification by flash chromatography (Scorpius silica, 40 g, pentane/EtOAc gradient from 1:0 to 0:1) to afford **37** (129 mg, 75%) as a yellow solid. R_f (EtOAc/pentane 8:2): 0.6; Mp: decomposed > 160 °C; IR (neat): 3293 (w), 3086 (w, C-H), 1600 (s, C=C), 1573 (m, C=C), 1328 (s, C-N), 1298 (m), 1136 (m), 1107 (s, C-F), 1089 (m), 827 (s, P-F), 720 (m); ^{1}H NMR (500 MHz, DMSO-d_6): 8.84 (d, $^4J_{H-H}$ = 1.8 Hz, 1H), 8.81 (d, $^4J_{H-H}$ = 1.8 Hz, 1H), 8.49 – 8.42 (m, 4H), 7.86 (d, $^3J_{H-H}$ = 5.7 Hz, 1H), 7.81 (d, $^3J_{H-H}$ = 5.7 Hz, 1H), 7.68 – 7.64 (m, 2H), 7.58 (dd, $^3J_{H-H}$ = 5.7, $^4J_{H-H}$ = 1.8 Hz, 1H), 7.52 (s, 1H), 7.07 (ddd, $^3J_{H-F}$ = 12.2, $^3J_{H-F}$ = 9.4, $^4J_{H-H}$ = 2.3 Hz, 2H), 5.78 (dt, $^3J_{H-F}$ = 8.3, $^4J_{H-H}$ = 2.3 Hz, 2H), 3.02 (t, $^3J_{H-H}$ = 7.1 Hz, 2H), 2.77 (t, $^4J_{H-H}$ = 2.6 Hz, 1H), 2.66 (td, $^3J_{H-H}$ = 7.1, $^4J_{H-H}$ = 2.6 Hz, 2H), 2.58 (s, 3H); ^{13}C NMR (126 MHz, DMSO-d_6): 166.8 (2C), 163.9 (dd, $^1J_{C-F}$ = 259.7, $^3J_{C-F}$ = 12.6 Hz, 2C), 161.7 (dd, $^1J_{C-F}$ = 259.7, $^3J_{C-F}$ = 12.6 Hz, 2C), 155.3 (d, $^2J_{C-F}$ = 7.6 Hz, C), 155.2 (d, $^2J_{C-F}$ = 7.6 Hz, C), 155.1 (C), 154.9 (C), 154.6 (C), 152.6 (C), 150.2 (CH), 150.0 (CH), 145.5 (q, $^3J_{C-F}$ = 5.0 Hz, CH), 145.1 (q, $^3J_{C-F}$ = 5.0 Hz, CH), 137.6 (2CH), 129.7 (CH), 129.1 (CH), 126.4 (2C), 125.9 (CH), 125.3 (CH), 124.6 (q, $^2J_{C-F}$ = 34.0 Hz, 2C), 123.8 (CH), 123.6 (CH), 121.9 (q, $^1J_{C-F}$ = 272.2 Hz, 2C), 114.2 (d, $^2J_{C-F}$ = 17.6 Hz, CH), 114.0 (d, $^2J_{C-F}$ = 17.6 Hz, CH), 99.6 (t, $^2J_{C-F}$ = 26.5 Hz, 2CH), 82.5 (C), 72.3 (CH), 33.2 (CH_2), 21.0 (CH_3), 18.1 (CH_2); ^{19}F NMR (282 MHz, DMSO-d_6): -61.6 (CF_3), -61.7 (CF_3), -70.1 (d, $^1J_{F-P}$ = 71.3 Hz, PF_6), -103.1 (td, J = 14.1, 5.8 Hz, 2F), -106.4 (dd, J = 12.0, 5.8 Hz, 2F); HRMS (ESI, +ve) calcd for $C_{39}H_{24}F_{16}IrN_4P$ $[M-PF_6]^+$: 929.1442, found: 929.1481.

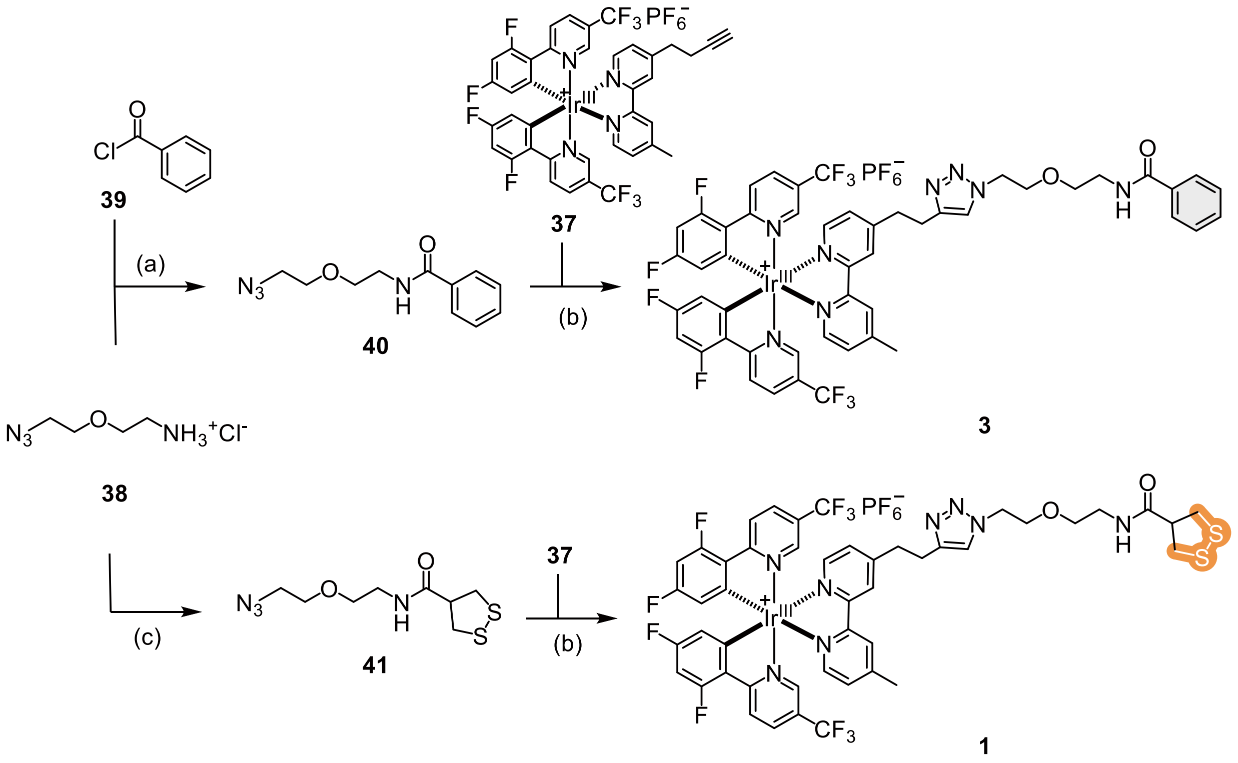

Scheme S3. (a) **39**, DIPEA, CH_2Cl_2, RT, 2 h, 80%; (b) **37**, CuI, TBTA, THF, RT, overnight, 88% for **3** and 89% for **1**; (c) DIPEA, CH_2Cl_2, RT, overnight, 97%.

Compound 41 was synthetized following the procedure described in reference S12.

Compound 40. Benzoyl chloride **39** (110 µL, 945 µmol) was added dropwise to a solution of **37** (189 mg, 1.10 mmol) and DIPEA (1.7 mL, 9.4 mmol) in dry CH_2Cl_2 (10 mL) at 0 °C. The reaction mixture was stirred for 2 h at RT under N_2 atmosphere. After completion of the reaction, the crude mixture was diluted in EtOAc, washed with 10% citric acid (x3) and brine, dried over Na_2SO_4, and concentrated *in vacuo*. The crude oil was subjected to purification by flash chromatography (Scorpius silica, 25 g, pentane/EtOAc 6:4) to afford **40** (178 mg, 80%) as a colorless oil. R_f (pentane/EtOAc 6:4) = 0.3; IR (neat): 3319 (br, N-H), 2925 (w, C-H), 2867 (w, C-H), 2099 (s, N_3), 1637 (s, C=O), 1602 (m, C=C), 1578 (m, C=C), 1533 (s, C=C), 1487 (s, C=C), 1289 (s, C-N), 1118 (s, C-O-C), 1026 (m), 929 (w), 865 (w), 802 (w), 711 (s), 693 (s), 669 (m), 556 (m); ^{1}H NMR (500 MHz, $CDCl_3$): 7.82 – 7.77 (m, 2H), 7.51 – 7.46 (m, 1H), 7.45 – 7.39 (m, 2H), 6.65 (bs, 1H), 3.70 – 3.65 (m, 6H), 3.40 (t, $^3J_{H\text{-}H}$ = 5.0 Hz, 2H); ^{13}C NMR (126 MHz, $CDCl_3$): 167.6 (C), 134.5 (C), 131.6 (CH), 128.6 (2CH),

127.1 (2CH), 70.3 (CH_2), 70.1 (CH_2), 50.8 (CH_2), 39.8 (CH_2); LRMS (ESI): 235 ($C_{11}H_{14}N_4O_2$, $[M+H]^+$).

Compound 3. Following the general procedure for CuAAC coupling, **37** (50 mg, 47 µmol) and **40** (15 mg, 56 µmol) were mixed in presence of CuI (18 mg, 93 µmol) and TBTA (12 mg, 23 µmol). After 20 hours of reaction time, purification by flash chromatography (Scorpius silica, 12 g, CH_2Cl_2/MeOH 98:2) afforded **3** (55 mg, 88%) as a yellow solid. R_f (CH_2Cl_2/MeOH 95:5): 0.4; Mp: > 120 °C decomposed; IR (neat): 2926 (w, C-H), 1600 (s, C=O), 1574 (m, C=C), 1488 (w, C=C), 1385 (w), 1328 (s, C-N), 1296 (s), 1251 (w), 1167 (m), 1107 (s, C-O-C), 1089 (s, C-F), 1050 (w), 991 (m, C-H), 828 (s, P-F), 720 (s, P-F), 713 (s), 693 (w), 677 (w), 614 (w), 556 (s), 527 (m); ^{1}H NMR (500 MHz, DMSO-d_6): 8.84 (d, $^4J_{H-H}$ = 1.7 Hz, 1H), 8.83 (d, $^4J_{H-H}$ = 1.7 Hz, 1H), 8.50 – 8.41 (m, 5H), 7.84 – 7.79 (m, 5H), 7.66 (d, $^4J_{H-H}$ = 1.7 Hz, 1H), 7.60 – 7.57 (m, 2H), 7.53 (d, $^4J_{H-H}$ = 1.7 Hz, 1H), 7.40 – 7.37 (m, 3H), 7.10 – 7.04 (m, 2H), 5.79 – 5.76 (m, 2H), 4.47 (t, $^3J_{H-H}$ = 5.3 Hz, 2H), 3.78 (t, $^3J_{H-H}$ = 5.3 Hz, 2H), 3.51 (t, $^3J_{H-H}$ = 5.3 Hz, 2H), 3.39 (t, $^3J_{H-H}$ = 5.3 Hz, 2H), 3.13 – 3.10 (m, 2H), 2.98 – 2.94 (m, 2H), 2.57 (s, 3H); ^{13}C NMR (126 MHz, DMSO-d_6): 166.8 (2C), 166.2 (C), 163.8 (dd, $^1J_{C-F}$ = 258.3, $^3J_{C-F}$ = 12.6 Hz, 2C), 161.7 (dd, $^1J_{C-F}$ = 258.3, $^3J_{C-F}$ = 12.6 Hz, 2C), 155.6 (C), 155.3 (d, $^2J_{C-F}$ = 6.3 Hz, 2C), 155.2 (C), 154.9 (C), 152.6 (C), 150.2 (CH), 145.0 (CH), 145.4 (q, $^3J_{C-F}$ = 5.0 Hz, CH), 145.1 (q, $^3J_{C-F}$ = 7.6 Hz, CH), 145.1 (C), 137.6 (2CH), 134.3 (C), 131.0 (CH), 129.7 (CH), 128.9 (CH), 128.2 (2CH), 127.1 (2CH), 126.5 (2C), 126.0 (CH), 125.1 (CH), 124.5 (q, $^2J_{C-F}$ = 34.0 Hz, 2C), 123.8 (CH), 123.6 (CH), 122.4 (CH), 121.9 (q, $^1J_{C-F}$ = 272.2 Hz, 2C), 114.2 (d, $^2J_{C-F}$ = 16.4 Hz, C), 114.0 (d, $^2J_{C-F}$ = 17.6 Hz, C), 99.6 (t, $^2J_{C-F}$ = 27.7 Hz, 2C), 68.6 (CH_2), 68.5 (CH_2), 49.3 (CH_2), 38.9 (CH_2), 34.0 (CH_2), 25.0 (CH_2), 20.9 (CH_3); ^{19}F NMR (282 MHz, DMSO-d_6): -61.5 (CF_3), -61.6 (CF_3), -70.1 (d, $^1J_{F-P}$ = 71.3 Hz, PF_6), -103.3 (dd, J = 11.8, 5.5 Hz, 2F), -106.6 – -106.7 (m, 2F); HRMS (ESI, +ve) calcd for $C_{50}H_{38}F_{16}IrN_8O_2P$ $[M-PF_6]^+$: 1163.2558, found: 1163.2582.

Compound 1. Following the general procedure for CuAAC coupling, **37** (50.0 mg, 46.5 µmol) and **41** (13.1 mg, 55.8 µmol) were mixed with CuI (17.7 mg, 93.0 µmol) and TBTA

(12.3 mg, 23.2 μmol). After 20 hours of reaction time, flash chromatography (Scorpius silica, 12 g, CH_2Cl_2/MeOH 98:2) afforded **1** (48 mg, 89%) as a yellow solid. R_f (CH_2Cl_2/MeOH 95:5): 0.2; Mp: > 125 °C decomposed; IR (neat): 3076 (w, N-H), 2163 (w), 1980 (w), 1667 (m), 1601 (s, C=O), 1574 (m, C=C), 1539 (m, C=C), 1494 (w), 1425 (w), 1385 (w), 1328 (s, C-N), 1298 (s, C-N), 1167 (s), 1129 (s, C-O-C), 1107 (s, C-F), 1089 (s, C-F), 1051 (m), 991 (s), 918 (w), 831 (s, P-F), 807 (s), 720 (s, P-F), 704 (s), 614 (w), 527 (w); ^{1}H NMR (500 MHz, DMSO-d_6): 8.89 (d, $^4J_{H-H}$ = 1.8 Hz, 1H), 8.84 (d, $^4J_{H-H}$ = 1.8 Hz, 1H), 8.50 – 8.41 (m, 4H), 8.13 (t, $^3J_{H-H}$ = 5.6 Hz, 1H), 7.88 (s, 1H), 7.84 (d, $^3J_{H-H}$ = 5.6 Hz, 1H), 7.81 (d, $^3J_{H-H}$ = 5.6 Hz, 1H), 7.68 – 7.62 (m, 2H), 7.58 (dd, $^3J_{H-H}$ = 5.6, $^4J_{H-H}$ = 1.8 Hz, 1H), 7.52 (s, 1H), 7.07 (ddd, $^3J_{H-F}$ = 12.1, $^3J_{H-F}$ = 9.4, $^4J_{H-H}$ = 1.8 Hz, 2H), 5.79 – 5.76 (m, 2H), 4.46 (t, $^3J_{H-H}$ = 5.6 Hz, 2H), 3.76 (t, $^3J_{H-H}$ = 5.6 Hz, 2H), 3.40 (t, $^3J_{H-H}$ = 5.6 Hz, 2H), 3.36 – 3.33 (m, 2H), 3.23 – 3.17 (m, 4H), 3.16 – 3.11 (m, 3H), 3.10 – 3.06 (m, 2H), 2.58 (s, 3H); ^{13}C NMR (126 MHz, DMSO-d_6): 170.5 (C), 166.8 (2C), 163.8 (dd, $^1J_{C-F}$ = 258.3, $^3J_{C-F}$ = 12.6 Hz, 2C), 161.7 (dd, $^1J_{C-F}$ = 258.3, $^3J_{C-F}$ = 12.6 Hz, 2C), 155.6 (C), 155.3 (d, $^2J_{C-F}$ = 7.6 Hz, 2C), 155.2 (C), 154.9 (C), 152.6 (C), 150.3 (CH), 150.0 (CH), 145.4 (q, $^3J_{C-F}$ = 5.0 Hz, CH), 145.2 (C), 145.1 (q, $^3J_{C-F}$ = 5.0 Hz, CH), 137.6 (2CH), 129.7 (CH), 129.0 (CH), 126.4 (2C), 126.0 (CH), 125.2 (CH), 124.5 (q, $^2J_{C-F}$ = 26.5 Hz, 2C), 123.8 (CH), 123.6 (CH), 122.4 (CH), 121.9 (q, $^1J_{C-F}$ = 272.2 Hz, 2C), 114.1 (d, $^2J_{C-F}$ = 18.9 Hz, C), 114.0 (d, $^2J_{C-F}$ = 17.6 Hz, C), 99.6 (t, $^2J_{C-F}$ = 27.7 Hz, 2C), 68.7 (CH_2), 68.5 (CH_2), 51.4 (CH), 49.2 (CH_2), 42.0 (2CH_2), 38.7 (CH_2), 34.1 (CH_2), 25.2 (CH_2), 21.0 (CH_3); ^{19}F NMR (282 MHz, DMSO-d_6): -61.5 (CF_3), -61.6 (CF_3), -70.2 (d, $^1J_{F-P}$ = 71.6 Hz, PF_6), -103.3 – -103.4 (m, 2F), -106.8 (dd, J = 12.0, 5.8 Hz, 2F); HRMS (ESI, +ve) calcd for $C_{47}H_{38}F_{16}IrN_8O_2PS_2$ [M-PF_6]$^+$: 1191.2001, found: 1191.2001.

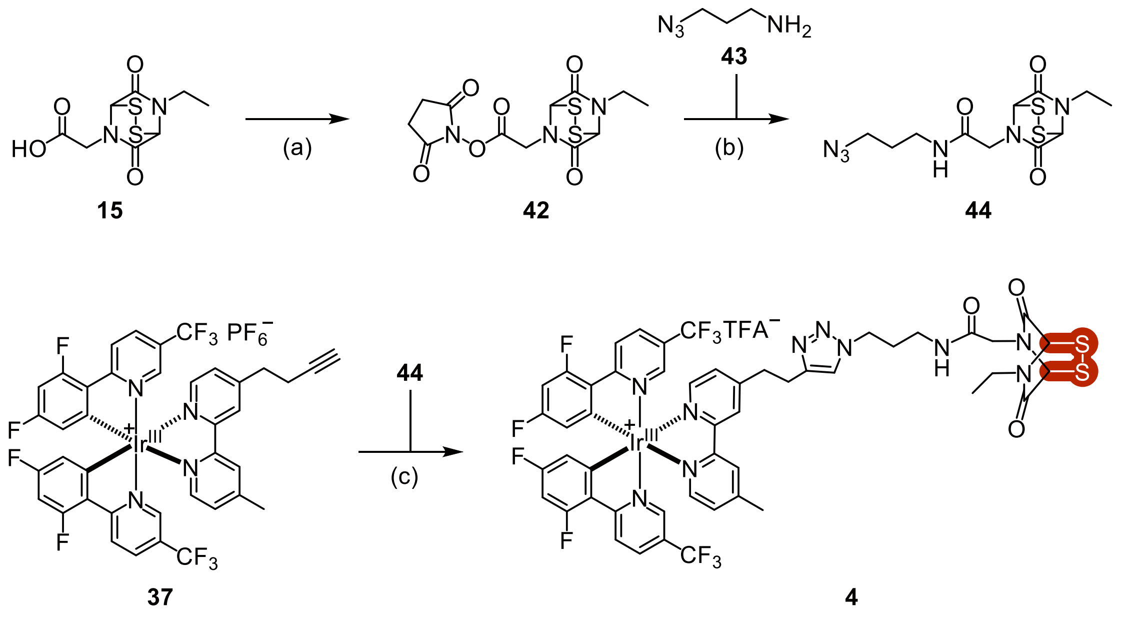

Scheme S4. (a) DCC, NHS, THF, RT, 24 h; (b) **43**, THF, 0 to RT, 2 h, 66% over two steps; (c) **44**, CuI, TBTA, THF, RT, 20 h, 47%.

Compound 42. To a solution of **15** (47 mg, 180 µmol) in dry THF (5 mL) was added DCC (45 mg, 220 µmol) at RT. The reaction mixture was stirred for 5 min to activate the ester. After this, *N*-hydroxysuccinimide (25 mg, 220 µmol) was added in one portion and the reaction mixture was stirred for 24 h at RT until the complete consumption of starting material (evidenced by LC-MS). The crude mixture was filtered through a PTFE syringe filter (0.2 µm) to remove DCU formed during the reaction. The clear filtrate was concentrated *in vacuo*, and the obtained yellow oil was engaged in the next step without any further purification.

Compound 44. The crude oil **42** was dissolved in dry THF (5 mL). To this solution was added dropwise over 10 min a solution of **43** (14 mg, 140 µmol) in dry THF (2 mL) at RT. The reaction mixture was stirred for 2 h at RT. Once the starting material was quasi-consumed (<90% based on LC-MS), THF was removed *in vacuo* and the crude solid was subjected to purification by flash chromatography (Scorpius silica, 12 g, CH_2Cl_2/MeOH 95:5) followed by RP flash chromatography (Scorpius C18, 33 g, H_2O + 0.1% TFA / CH_3CN + 0.1% TFA, 0 to 100% of CH_3CN + 0.1% TFA) to afford **44** (41 mg, 66%) as a pale yellow solid. R_f (CH_2Cl_2/MeOH 95:5): 0.3; Mp: 205.5 – 206.3 °C;

IR (neat): 3385 (m, N-H), 2100 (m, N_3), 1740 (s, C=O), 1672 (s, C=O), 1553 (m), 1430 (m, C-H), 1381 (m), 1260 (w), 1202 (s, C-N), 1131 (s, C-N), 1049 (m), 1025 (s), 1002 (s), 827 (m), 800 (m), 721 (s), 619 (m), 562 (m), 518 (m); ^{1}H NMR (500 MHz, DMSO-d_6): 8.18 (t, $^3J_{H-H}$ = 5.7 Hz, 1H), 6.12 (s, 1H), 5.95 (s, 1H), 4.26 (d, $^2J_{H-H}$ = 16.5 Hz, 1H), 3.82 (d, $^2J_{H-H}$ = 16.5 Hz, 1H), 3.50 – 3.42 (m, 2H), 3.36 (t, $^3J_{H-H}$ = 6.8 Hz, 2H), 3.15 – 3.11 (m, 2H), 1.66 (p, $^3J_{H-H}$ = 7.0 Hz, 2H), 1.16 (t, $^3J_{H-H}$ = 7.0 Hz, 3H); ^{13}C NMR (126 MHz, DMSO-d_6): 166.5 (C), 164.0 (C), 163.6 (C), 65.7 (CH), 64.2 (CH), 48.3 (CH_2), 45.0 (CH_2), 39.3 (CH_2), 36.0 (CH_2), 28.3 (CH_2), 12.7 (CH_3); LRMS (ESI): 345 ($C_{11}H_{16}N_6O_3S_2$, $[M+H]^+$).

Compound 4. Following the general procedure A, **37** (28 mg, 26 μmol) and **44** (15 mg, 44 μmol) were suspended in dry THF (5 mL) with CuI (10 mg, 52 μmol) and TBTA (7.0 mg, 13 μmol). The reaction mixture was stirred at RT for 20 h (completion evidenced by LC-MS). Flash chromatography (Scorpius silica, 25 g, CH_2Cl_2/MeOH 98:2) followed by RP-chromatography (Scorpius C18, 33 g, H_2O + 0.1% TFA / CH_3CN + 0.1% TFA, 1:1) afforded **4** (17 mg, 47%) as a yellow solid. R_f (CH_2Cl_2/MeOH 95:5): 0.23; Mp: > 120 °C decomposed; IR (neat): 2942 (w, N-H), 1682 (m, C=C), 1600 (s, C=O), 1574 (m, C=C), 1492 (w), 1423 (w), 1385 (w), 1328 (s, C-N), 1297 (s, C-N), 1166 (s, C-N), 1131 (s, C-N), 1107 (s, C-F), 1089 (s, C-F), 1050 (m), 990 (s, C-H), 845 (s), 830 (s), 720 (s), 705 (s), 613 (w), 557 (w), 527 (m); ^{1}H NMR (500 MHz, DMSO-d_6): 8.89 (d, $^4J_{H-H}$ = 1.8 Hz, 1H), 8.85 (d, $^4J_{H-H}$ = 1.7 Hz, 1H), 8.50 – 8.41 (m, 4H), 8.26 (t, $^3J_{H-H}$ = 5.6 Hz, 1H), 7.91 (s, 1H), 7.82 (d, $^3J_{H-H}$ = 5.9 Hz, 1H), 7.80 (d, $^3J_{H-H}$ = 5.9 Hz, 1H), 7.68 – 7.62 (m, 2H), 7.58 (dd, $^3J_{H-H}$ = 5.9 Hz, $^4J_{H-H}$ = 2.1 Hz, 1H), 7.52 (d, $^4J_{H-H}$ = 2.1 Hz, 1H), 7.09 – 7.04 (m, 2H), 6.12 (s, 1H), 5.95 (d, $^4J_{H-H}$ = 2.1 Hz, 1H), 5.79 – 5.77 (m, 2H), 4.34 – 4.27 (m, 3H), 3.84 (d, $^2J_{H-H}$ = 16.4 Hz, 1H), 3.50 – 3.40 (m, 2H), 3.20 (t, $^3J_{H-H}$ = 7.1 Hz, 2H), 3.10 – 3.00 (m, 4H), 2.57 (s, 3H), 1.93 (p, $^3J_{H-H}$ = 7.1 Hz, 2H), 1.14 (t, $^3J_{H-H}$ = 7.1 Hz, 3H); ^{13}C NMR (126 MHz, DMSO-d_6): 166.8 (d, $^2J_{C-F}$ = 6.3 Hz, 2C), 166.6 (C), 164.0 (C), 164.3 (dd, $^1J_{C-F}$ = 245.4, $^3J_{C-F}$ = 12.7 Hz, 2C), 163.6 (C), 162.3 (dd, $^1J_{C-F}$ = 245.4, $^3J_{C-F}$ = 12.7 Hz, 2C), 158.0 (q, $^2J_{C-F}$ = 34.0 Hz, TFA), 155.6 (C), 155.3 (d, $^2J_{C-F}$ = 7.6 Hz, 2C),

155.2 (C), 155.0 (C), 152.6 (C), 150.3 (CH), 149.9 (CH), 145.4 (q, $^{3}J_{C-F}$ = 5.8 Hz, 2CH), 145.1 (C), 137.6 (2CH), 129.7 (CH), 129.0 (CH), 126.5 (2C), 126.0 (CH), 125.2 (CH), 124.6 (q, $^{2}J_{C-F}$ = 32.8 Hz, 2C), 123.8 (CH), 123.6 (CH), 122.2 (CH), 121.9 (q, $^{1}J_{C-F}$ = 272.9 Hz, 2C), 114.1 (d, $^{2}J_{C-F}$ = 10.1 Hz, CH), 114.0 (d, $^{2}J_{C-F}$ = 10.1 Hz, CH), 99.6 (t, $^{2}J_{C-F}$ = 27.7 Hz, 2CH), 65.6 (CH), 64.1 (CH), 46.9 (CH_2), 45.0 (CH_2), 39.1 (CH_2 under DMSO), 35.8 (CH_2), 34.1 (CH_2), 29.7 (CH_2), 25.3 (CH_2), 20.9 (CH_3), 12.6 (CH_3); ^{19}F NMR (282 MHz, DMSO-d_6): -61.5 (CF_3), -61.7 (CF_3), -74.2 (TFA), -103.4 (t, J = 12.2 Hz, 2F), -106.8 (dd, J = 11.9, 5.5 Hz, 2F); HRMS (ESI, +ve) calcd for $C_{52}H_{40}F_{13}IrN_{10}O_4S_2$ $[M\text{-}TFA]^+$: 1275.2196, found: 1275.2169.

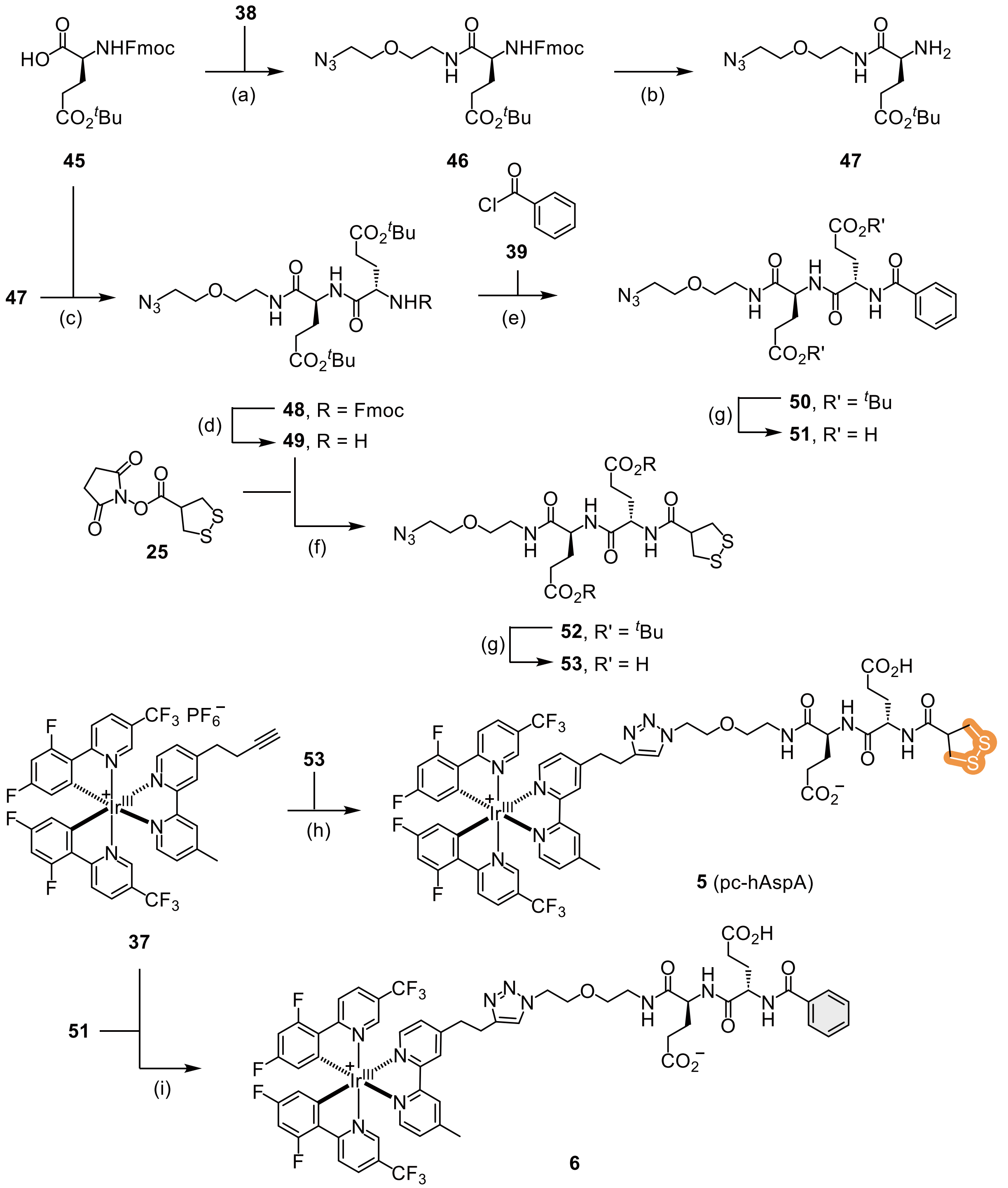

Scheme S5. (a) **38**, HATU, DIPEA, CH_2Cl_2, 45 min, 0 to RT, 72%; (b) $NHMe_2$ 2 M THF, RT, 1 h, 99%; (c) **45**, HATU, DIPEA, CH_2Cl_2, 1.25 h, 0 to RT, 85%; (d) $NHMe_2$ 2 M THF, RT, 1 h, quant; (e) **39**, DIPEA, CH_2Cl_2, 2 h, RT, 96%; (f) **25**, DIPEA, CH_2Cl_2, 2 h, RT, 84%; (g) TFA 50% in CH_2Cl_2, RT, 1 h; (h) **53**, CuI, TBTA, THF, RT, 6 h, 62% over two steps; (i) **51**, CuI, TBTA, THF, RT, 6 h, 57% over two steps.

Compound 46. Fmoc-L-Glu(O^{t}Bu)-OH **45** (889 mg, 2.09 mmol) in dry CH_2Cl_2 (10 mL) was premixed with HATU (794 mg, 2.09 mmol) and DIPEA (616 µL, 3.48 mmol) under N_2 atmosphere. The reaction mixture was stirred at RT for 15 min. The activated ester was then added dropwise to a solution of **38** and DIPEA (616 µL, 3.48 mmol) in dry CH_2Cl_2 (10 mL) at 0 °C. The reaction mixture was stirred for 30 min at RT. After completion of the reaction, a sat. solution of $NaHCO_3$ (50 mL) was added to quench the reaction. The crude product was extracted with EtOAc (3 x 50 mL). The combined organic layers were washed with brine, dried over Na_2SO_4 and concentrated *in vacuo*. The crude oil was subjected to purification by flash chromatography (Scorpius silica, 40 g, pentane/EtOAc 1:1) to afford **46** (677 mg, 72%) as a colorless foam. R_f (pentane/EtOAc 1:1) = 0.4; $[\alpha]_D^{20}$ -3.1 (*c* 1.0, $CHCl_3$); Mp: 101.2 – 101.5 °C; IR (neat): 3292 (m, C-H), 2977 (w), 2933 (w), 2101 (m, N_3), 1722 (s, C=O), 1688 (s, C=O), 1644 (s, C=O), 1533 (s, C=C), 1448 (m, C=C), 1366 (m), 1248 (s, C-N), 1151 (s, C-O-C), 1084 (m), 1043 (m), 848 (m), 756 (m), 737 (s), 644 (m), 621 (w), 590 (w), 542 (w); ^{1}H NMR (500 MHz, $CDCl_3$): 7.76 (d, $^3J_{H-H}$ = 7.0 Hz, 2H), 7.60 (d, $^3J_{H-H}$ = 7.0 Hz, 2H), 7.40 (t, $^3J_{H-H}$ = 7.0 Hz, 2H), 7.32 (t, $^3J_{H-H}$ = 7.0 Hz, 2H), 6.54 (bs, 1H), 5.71 (d, $^3J_{H-H}$ = 7.8 Hz, 1H), 4.38 (d, $^3J_{H-H}$ = 7.0 Hz, 2H), 4.23 – 4.18 (m, 2H), 3.65 (t, $^3J_{H-H}$ = 4.9 Hz, 2H), 3.61 – 3.54 (m, 2H), 3.50 (q, $^3J_{H-H}$ = 5.2 Hz, 2H), 3.35 (t, $^3J_{H-H}$ = 5.2 Hz, 2H), 2.42 (dt, $^2J_{H-H}$ = 15.0, $^3J_{H-H}$ = 7.0 Hz, 1H), 2.31 (dt, $^2J_{H-H}$ = 15.0, $^3J_{H-H}$ = 7.0 Hz, 1H), 2.10 (dq, $^2J_{H-H}$ = 15.0, $^3J_{H-H}$ = 7.0 Hz, 1H), 1.94 (dq, $^3J_{H-H}$ = 15.0, $^2J_{H-H}$ = 7.0 Hz, 1H), 1.46 (s, 9H); ^{13}C NMR (126 MHz, $CDCl_3$): 172.9 (C), 171.4 (C), 156.3 (C), 144.0 (2C), 141.4 (2C), 127.9 (2CH), 127.2 (2CH), 125.3 (2CH), 120.1 (2CH), 81.2 (C), 70.2 (CH_2), 69.8 (CH_2), 67.3 (CH_2), 54.6 (CH), 50.7 (CH_2), 47.3 (CH), 39.4 (CH_2), 31.9 (CH_2), 28.4 (CH_2), 28.2 ($3CH_3$); LRMS (ESI): 538 ($C_{28}H_{35}N_5O_6$, $[M+H]^+$).

Compound 47. To **46** (550 mg, 1.02 mmol) in dry THF (5 mL) under a stream of N_2 was added dimethylamine (5.1 mL, 10 mmol) (2.0 M in THF) at RT. The reaction mixture was stirred at RT for 1 h. After completion of the reaction (monitored by TLC), THF was removed *in vacuo* and the obtained crude oil was subjected to purification by flash chromatography (Scorpius silica, 12 g,

CH_2Cl_2/MeOH + 10% NH_4OH 95:5) to afford the deprotected compound **47** (320 mg, 99%) as a colorless oil. R_f (CH_2Cl_2/MeOH + 10% NH_4OH 95:5) = 0.4; $[\alpha]_D^{20}$ -4.5 (*c* 1.0, CH_3OH); IR (neat): 3300 (m, N-H), 2973 (w, C-H), 2931 (w, C-H), 2102 (s, N_3), 1719 (s, C=O), 1654 (s, C=O), 1527 (s, N-H), 1447 (m), 1366 (m, C-N), 1253 (s, C-N), 1150 (s, C-O-C), 1123 (s, C-O), 917 (w), 846 (m), 751 (w), 664 (w), 557 (w); ^1H NMR (500 MHz, $CDCl_3$): 7.45 (t, $^3J_{H-H}$ = 6.1 Hz, 1H), 3.67 – 3.61 (m, 2H), 3.58 – 3.52 (m, 2H), 3.48 – 3.41 (m, 2H), 3.39 – 3.32 (m, 3H), 2.39 – 2.25 (m, 2H), 2.10 – 2.03 (m, 1H), 1.82 – 1.75 (m, 1H), 1.41 (s, 9H); ^{13}C NMR (126 MHz, $CDCl_3$): 174.7 (C), 172.8 (C), 80.6 (C), 70.0 (CH_2), 69.9 (CH_2), 54.7 (CH), 50.7 (CH_2), 38.9 (CH_2), 32.1 (CH_2), 30.4 (CH_2), 28.2 ($3CH_3$); LRMS (ESI): 316 ($C_{13}H_{25}N_5O_4$, $[M+H]^+$).

Compound 48. To a solution of Fmoc-L-Glu(O^{t}Bu)-OH **45** (337 mg, 793 µmol) and DIPEA (280 µL, 1.59 mmol) in CH_2Cl_2 (10 mL) was added HATU (332 mg, 872 µmol) in one portion at 0 °C. The reaction mixture was stirred for 15 min at this temperature to form an activated ester. A solution of **47** (300 mg, 951 µmol) and DIPEA (280 µL, 1.59 mmol) in CH_2Cl_2 (10 mL) was added to the solution of activated ester and the reaction mixture was stirred at RT for 1 h (completion monitored by TLC). The reaction mixture was dissolved in EtOAc (100 mL) and washed with 10% citric acid followed by brine, dried over Na_2SO_4 and concentrated under reduced pressure. The obtained crude solid was subjected to purification by flash chromatography (Scorpius Silica, 40 g, pentane/EtOAc 1:1) to afford **48** (487 mg, 85%) as a colorless solid. R_f (pentane/EtOAc 1:1) = 0.4; $[\alpha]_D^{20}$ -11 (*c* 1.0 $CHCl_3$); Mp: 75.3 – 76.7 °C; IR (neat): 3289 (m, N-H), 2977 (w, C-H), 2932 (w, C-H), 2102 (m, N_3), 1723 (s, C=O), 1693 (s, C=O), 1642 (s, C=O), 1531 (s, C=C), 1448 (m, C=C), 1366 (m), 1252 (s, C-N), 1150 (s, C-O-C), 1084 (m, C-O), 1043 (m), 940 (w), 848 (m), 757 (m), 738 (s), 665 (m), 621 (m), 544 (w); ^1H NMR (500 MHz, $CDCl_3$): 7.76 (d, $^3J_{H-H}$ = 7.5 Hz, 2H), 7.60 (d, $^3J_{H-H}$ = 7.5 Hz, 2H), 7.40 (t, $^3J_{H-H}$ = 7.5 Hz, 2H), 7.31 (t, $^3J_{H-H}$ = 7.5 Hz, 2H), 7.22 (d, $^3J_{H-H}$ = 7.5 Hz, 1H), 6.79 (t, $^3J_{H-H}$ = 5.8 Hz, 1H), 5.96 (d, $^3J_{H-H}$ = 6.8 Hz, 1H), 4.46 – 4.33 (m, 3H), 4.21 (t, $^3J_{H-H}$ = 7.5 Hz, 1H), 4.20 – 4.16 (m, 1H), 3.62 (t, $^3J_{H-H}$ = 5.8 Hz, 2H), 3.55 (t, $^3J_{H-H}$ = 5.8 Hz, 2H), 3.51 – 3.40 (m,

2H), 3.33 (t, $^3J_{H-H}$ = 5.8 Hz, 2H), 2.49 – 2.25 (m, 4H), 2.17 – 2.06 (m, 2H), 2.01 – 1.90 (m, 2H), 1.46 (s, 9H), 1.43 (s, 9H); ^{13}C NMR (126 MHz, $CDCl_3$): 173.1 (2C), 171.5 (C), 171.0 (C), 156.6 (C), 143.9 (2C), 141.4 (2C), 127.9 (2CH), 127.2 (2CH), 125.3 (2CH), 120.1 (2CH), 81.4 (C), 81.2 (C), 70.0 (CH_2), 69.6 (CH_2), 67.4 (CH_2), 55.2 (CH), 53.0 (CH), 50.7 (CH_2), 47.2 (CH), 39.4 (CH_2), 32.0 (CH_2), 31.9 (CH_2), 28.2 ($6CH_3$), 27.9 (CH_2), 27.6 (CH_2); LRMS (ESI): 773 ($C_{37}H_{50}N_6O_9$, $[M+H]^+$).

Compound 49. To **48** (450 mg, 623 μmol) under a N_2 stream was added dropwise a solution of dimethylamine (5.0 mL, 10 mmol) (2.0 M in THF). The reaction mixture was stirred at RT for 1 h. After completion of the reaction (monitored by TLC), THF was removed *in vacuo*. The crude oil was subjected to purification by flash chromatography (Scorpius Silica, 12 g, CH_2Cl_2/MeOH + 10% NH_4OH 95:5) to afford **49** (312 mg, quantitative) as a colorless oil. R_f (CH_2Cl_2/MeOH + 10% NH_4OH 95:5) = 0.3; $[\alpha]_D^{20}$ -10 (*c* 1.0, CH_3OH); IR (neat): 3283 (m, N-H), 2975 (w, C-H), 2931 (w, C-H), 2104 (m, N_3), 1724 (s, C=O), 1631 (s, C=O), 1531 (s, N-H), 1452 (m), 1366 (s, C-N), 1273 (s, C-N), 1253 (s, C-N), 1150 (s, C-O-C), 1126 (s, C-O), 847 (m), 759 (w), 696 (w), 664 (w), 555 (w); ^{1}H NMR (400 MHz, $CDCl_3$): 7.79 (d, $^3J_{H-H}$ = 8.0 Hz, 1H), 6.66 (t, $^3J_{H-H}$ = 5.6 Hz, 1H), 4.38 (td, $^3J_{H-H}$ = 8.0, $^3J_{H-H}$ = 5.6 Hz, 1H), 3.63 (t, $^3J_{H-H}$ = 4.8 Hz, 2H), 3.55 (t, $^3J_{H-H}$ = 4.8 Hz, 2H), 3.48 – 3.43 (m, 2H), 3.40 – 3.34 (m, 3H), 2.40 – 2.22 (m, 4H), 2.16 – 2.01 (m, 2H), 1.97 – 1.86 (m, 1H), 1.84 – 1.74 (m, 1H), 1.43 (s, 18H); ^{13}C NMR (101 MHz, $CDCl_3$): 175.0 (C), 172.8 (C), 172.6 (C), 171.3 (C), 81.0 (C), 80.7 (C), 70.1 (CH_2), 69.8 (CH_2), 54.8 (CH), 52.4 (CH), 50.8 (CH_2), 39.4 (CH_2), 32.1 (CH_2), 31.9 (CH_2), 30.4 (CH_2), 28.2 ($6CH_3$), 28.0 (CH_2); LRMS (ESI): 501 ($C_{22}H_{40}N_6O_7$, $[M+H]^+$).

Compound 50. To a solution of **49** (83 mg, 160 μmol) and DIPEA (145 μL, 819 μmol) in dry CH_2Cl_2 (15 mL) was added dropwise **39** (38.0 μL, 328 μmol) at RT. The reaction mixture was stirred for 2 h at RT (completion evidenced by TLC). The reaction mixture was diluted in EtOAc (50 mL) and was washed with 1 M HCl followed by brine. The organic phase was dried over Na_2SO_4 and concentrated *in vacuo*. The obtained crude solid was subjected to purification by flash chromatography (Scorpius Silica, 12 g, CH_2Cl_2/MeOH 99:1 to 95:5) to afford **50** (95 mg, 96%) as a

colorless solid. R_f (CH_2Cl_2/MeOH 99:1) = 0.2; $[\alpha]_D^{20}$ -13 (*c* 1.0, CH_3Cl); Mp: 135.2 – 135.5 °C; IR (neat): 3281 (w, N-H), 2973 (w, C-H), 2103 (s, N_3), 1724 (s, C=O), 1683 (s, C=O), 1623 (s, C=O), 1575 (s, C=C), 1530 (m, C=C), 1489 (m), 1450 (m), 1393 (m, C-N), 1308 (s, C-N), 1142 (s, C-O-C), 1074 (s, C-O), 940 (s), 849 (s), 802 (s), 758 (s), 538 (s); ^{1}H NMR (500 MHz, $CDCl_3$): 7.90 (d, $^3J_{H-H}$ = 7.2 Hz, 3H), 7.57 (d, $^3J_{H-H}$ = 7.2 Hz, 1H), 7.52 (t, $^3J_{H-H}$ = 7.2 Hz, 1H), 7.44 (t, $^3J_{H-H}$ = 7.2 Hz, 2H), 6.99 (t, $^3J_{H-H}$ = 5.7 Hz, 1H), 4.48 – 4.44 (m, 2H), 3.64 (t, $^3J_{H-H}$ = 5.7 Hz, 2H), 3.60 (t, $^3J_{H-H}$ = 5.7 Hz, 2H), 3.57 – 3.50 (m, 1H), 3.48 – 3.41 (m, 1H), 3.33 (t, $^3J_{H-H}$ = 5.7 Hz, 2H), 2.62 – 2.56 (m, 1H), 2.48 – 2.34 (m, 2H), 2.32 – 2.26 (m, 1H), 2.25 – 2.13 (m, 2H), 2.13 – 2.03 (m, 1H), 2.04 – 1.94 (m, 1H), 1.44 (s, 9H), 1.33 (s, 9H); ^{13}C NMR (126 MHz, $CDCl_3$): 174.5 (C), 173.5 (C), 171.4 (C), 171.1 (C), 168.2 (C), 133.4 (C), 132.1 (CH), 128.7 (2CH), 127.5 (2CH), 81.8 (C), 81.1 (C), 70.0 (CH_2), 69.6 (CH_2), 55.5 (CH), 53.1 (CH), 50.7 (CH_2), 39.5 (CH_2), 32.5 (CH_2), 31.9 (CH_2), 28.2 ($3CH_3$), 28.1 ($3CH_3$), 27.0 (CH_2), 26.5 (CH_2); LRMS (ESI): 605 ($C_{29}H_{44}N_6O_8$, $[M+H]^+$).

Compound 51. To a solution of **50** (50 mg, 83 µmol) in dry CH_2Cl_2 (5 mL) was added dropwise trifluoroacetic acid (5.00 mL, 65.3 mmol) at RT. The reaction mixture was stirred for 60 min at RT until completion of the reaction (monitored by TLC). Once **50** was fully consumed, the crude mixture was dried with a stream of N_2 to remove the excess of TFA. The crude solid was co-evaporated with toluene to remove traces of TFA, and triturated twice in diethylether. The crude solid was engaged in the next step without further purification.

Compound 52. To a solution of AspA-NHS **25** (30 mg, 120 µmol) in dry CH_2Cl_2 (15 mL) was added dropwise a solution of **49** (50 mg, 100 µmol) and DIPEA (27 µL, 150 µmol) in dry CH_2Cl_2 (5 mL) at RT. The reaction mixture was allowed to stir at RT until full completion was observed (monitored by TLC, 2 h). The reaction mixture was diluted in EtOAc (50 mL) and washed with 1 M HCl followed by brine. The organic phase was dried over Na_2SO_4 and concentrated *in vacuo*. The obtained crude solid was subjected to purification by flash chromatography (Scorpius Silica, 12 g, CH_2Cl_2/MeOH 99:1 to 95:5) to afford **52** (53 mg, 84%) as a colorless solid. R_f (CH_2Cl_2/MeOH 98:2)

= 0.3; $[\alpha]_D^{20}$ -11 (*c* 1.0, CH_3Cl); Mp: 134.5 – 135.5 °C; IR (neat): 3268 (m, NH), 2974 (w, C-H), 2928 (w, C-H), 2108 (m, N_3), 1724 (s, C=O), 1628 (s, C=O), 1531 (s), 1453 (m), 1367 (m), 1272 (s, C-N), 1149 (s, C-O-C), 1124 (s, C-O), 989 (w), 938 (w), 849 (m), 760 (m), 709 (m), 665 (m); ^1H NMR (400 MHz, $CDCl_3$): 7.55 (d, $^3J_{H-H}$ = 5.9 Hz, 2H), 7.00 (t, $^3J_{H-H}$ = 5.9 Hz, 1H), 4.54 – 4.46 (m, 2H), 3.64 (t, $^3J_{H-H}$ = 5.0 Hz, 2H), 3.57 (t, $^3J_{H-H}$ = 5.0 Hz, 2H), 3.50 – 3.41 (m, 3H), 3.42 – 3.33 (m, 7H), 2.45 – 2.21 (m, 4H), 2.10 – 2.02 (m, 2H), 1.99 – 1.88 (m, 2H), 1.43 (s, 9H), 1.42 (s, 9H); ^{13}C NMR (101 MHz, $CDCl_3$): 173.3 (C), 172.8 (C), 171.9 (2C), 171.1 (C), 81.4 (C), 81.1 (C), 69.9 (CH_2), 69.6 (CH_2), 53.6 (CH), 52.9 (CH), 52.4 (CH), 50.7 (CH_2), 42.4 (CH_2), 42.2 (CH_2), 39.5 (CH_2), 31.9 (CH_2), 31.7 (CH_2), 28.2 ($6CH_3$), 28.0 (CH_2), 27.7 (CH_2); LRMS (ESI): 634 ($C_{26}H_{44}N_6O_8S_2$, $[M+H]^+$).

Compound 53. To a solution of **52** (50 mg, 83 µmol) in dry CH_2Cl_2 (5 mL) was added dropwise trifluoroacetic acid (5.00 mL, 65.3 mmol) at RT. The reaction mixture was stirred at RT until completion of the reaction (monitored by TLC). Once the SM was fully consumed, the crude mixture was dried with a stream of N_2 to remove the excess of TFA. The crude solid was co-evaporated with toluene to remove traces of TFA, and triturated twice in diethylether. The crude solid was engaged in the next step without further purification.

Compound 5. Following the general procedure for CuAAC coupling, **37** (17 mg, 16 µmol) and **53** (10 mg, 19 µmol) were mixed with TBTA (4.3 mg, 8.0 µmol) and CuI (6.1 mg, 32 µmol). After 6 hours of reaction time, RP chromatography (Scorpius C_{18}, 33 g, linear gradient of ACN + 0.1% TFA 0 to 100% in H_2O + 0.1% TFA) afforded **5** (13.2 mg, 57% over 2 steps) as a yellowish solid. Mp: > 113 °C, decomposed; IR (neat): 2930 (w, C-H), 2162 (w), 1601 (s, C=O), 1575 (m, C=C), 1491 (m, C=C), 1424 (m), 1396 (m), 1328 (s, C-N), 1297 (s, C-N), 1251 (m), 1167 (m), 1133 (s, C-O-C), 1107 (s, C-O), 1089 (s, C-F), 1051 (m), 991 (m), 830 (s), 720 (s), 614 (w), 556 (m), 527 (w); ^1H NMR (500 MHz, DMSO-d_6): 8.89 (s, 1H), 8.84 (d, $^4J_{H-H}$ = 1.7 Hz, 1H), 8.50 – 8.41 (m, 4H), 8.33 (d, $^3J_{H-H}$ = 7.5 Hz, 1H), 7.97 (d, $^3J_{H-H}$ = 9.9 Hz, 1H), 7.89 – 7.86 (m, 2H), 7.83 (d, $^3J_{H-H}$ = 5.7 Hz, 1H), 7.81 (d, $^3J_{H-H}$ = 5.7 Hz, 1H), 7.66 – 7.64 (m, 2H), 7.58 (d, $^3J_{H-H}$ = 5.7 Hz, 1H), 7.51 (s, 1H),

7.10 – 7.04 (m, 2H), 5.79 – 5.75 (m, 2H), 4.45 (t, $^3J_{H\text{-}H}$ = 5.3 Hz, 2H), 4.26 – 4.17 (m, 2H), 3.75 (t, $^3J_{H\text{-}H}$ = 5.4 Hz, 2H), 3.42 – 3.33 (m, 3H), 3.28 – 3.11 (m, 6H), 3.08 (t, $^3J_{H\text{-}H}$ = 8.0 Hz, 2H), 2.57 (s, 3H), 2.25 (t, $^3J_{H\text{-}H}$ = 7.9 Hz, 2H), 2.22 – 2.17 (m, 2H), 1.93 – 1.84 (m, 2H), 1.78 – 1.69 (m, 2H); ^{13}C NMR (126 MHz, DMSO-d_6): 173.9 (2C), 171.0 (C), 170.9 (2C), 166.7 (2C), 163.9 (dd, $^1J_{C\text{-}F}$ = 256.6, $^3J_{C\text{-}F}$ = 12.7 Hz, 2C), 161.7 (dd, $^1J_{C\text{-}F}$ = 256.6, $^3J_{C\text{-}F}$ = 12.7 Hz, 2C), 155.6 (C), 155.3 (d, $^2J_{C\text{-}F}$ = 6.3 Hz, 2C), 155.2 (C), 154.9 (C), 152.6 (C), 150.2 (CH), 149.9 (CH), 145.4 (C), 145.1 (2CH), 137.6 (2CH), 129.6 (CH), 128.9 (CH), 126.4 (2C), 126.0 (CH), 125.2 (CH), 124.5 (q, $^2J_{C\text{-}F}$ = 32.8 Hz, 2C), 123.7 (CH), 123.6 (CH), 122.5 (CH), 121.9 (q, $^1J_{C\text{-}F}$ = 272.2 Hz, 2C), 114.0 (d $^2J_{C\text{-}F}$ = 15.1 Hz, 2CH), 99.6 (t, $^2J_{C\text{-}F}$ = 27.7 Hz, 2CH), 68.6 (CH_2), 68.5 (CH_2), 52.4 (CH), 51.9 (CH), 51.0 (CH), 49.2(CH_2), 42.1 (CH_2), 42.0 (CH_2), 38.3 (CH_2), 34.1 (CH_2), 30.1 (CH_2), 30.0 (CH_2), 27.2 (CH_2), 27.0 (CH_2), 25.2 (CH_2), 20.9 (CH_3); ^{19}F NMR (282 MHz, DMSO-d_6): -61.5 (CF_3), -61.6 (CF_3), -103.3 (dd, J = 11.8, 8.7 Hz, 2F), -106.8 (dd, J = 12.0, 5.3 Hz, 2F); HRMS (ESI, +ve) calcd for $C_{57}H_{52}F_{10}IrN_{10}O_8S_2$ $[M+H]^+$: 1451.2875, found: 1451.2897.

Compound 6. Following the general procedure for CuAAC coupling, **37** (20 mg, 19 μmol) and **51** (37 mg, 74 μmol) were mixed in presence of TBTA (5 mg, 9 μmol) and CuI (7 mg, 37 μmol). After 6 hours of reaction time, RP chromatography (Scorpius C18, 33 g, linear gradient of ACN + 0.1% TFA 0 to 100% in H_2O + 0.1% TFA) afforded **6** (13 mg, 57% over 2 steps) as a yellow solid. Mp: > 120 °C, decomposed; IR (neat): 3072 (w, N-H), 1980 (w), 1659 (m, C=C), 1600 (s, C=O), 1574 (m, C=C), 1491 (m, C=C), 1423 (w), 1385 (m), 1328 (s, C-N), 1297 (m, C-N), 1251 (w), 1167 (s, C-N), 1130 (s, C-O-C), 1107 (s, C-O), 1089 (s, C-F), 1020 (w), 991 (m), 919 (w), 846 (m), 831 (m), 798 (m), 720 (s), 614 (w), 527 (w); ^{1}H NMR (500 MHz, DMSO-d_6): 8.88 (d, $^4J_{H\text{-}H}$ = 1.9 Hz, 1H), 8.83 (s, 1H), 8.52 (d, $^3J_{H\text{-}H}$ = 7.4 Hz, 1H), 8.50 – 8.39 (m, 4H), 8.01 (d, $^3J_{H\text{-}H}$ = 7.4 Hz, 1H), 7.92 – 7.84 (m, 4H), 7.83 (d, $^3J_{H\text{-}H}$ = 5.7 Hz, 1H), 7.81 (d, $^3J_{H\text{-}H}$ = 5.7 Hz, 1H), 7.67 – 7.62 (m, 2H), 7.57 (dd, $^3J_{H\text{-}H}$ = 5.7, $^4J_{H\text{-}H}$ = 1.9 Hz, 1H), 7.55 – 7.50 (m, 2H), 7.45 (t, $^3J_{H\text{-}H}$ = 7.4 Hz, 2H), 7.09 – 7.03 (m, 2H), 5.82 – 5.57 (m, 3H), 4.45 (t, $^3J_{H\text{-}H}$ = 5.7 Hz, 2H), 4.42 – 4.38 (m, 1H), 4.24 (td, $^3J_{H\text{-}H}$ = 8.2

Hz, $^3J_{H\text{-}H}$ = 5.3 Hz, 1H), 3.74 (t, $^3J_{H\text{-}H}$ = 5.3 Hz, 2H), 3.38 (t, $^3J_{H\text{-}H}$ = 5.8 Hz, 2H), 3.24 – 3.09 (m, 4H), 3.07 (t, $^3J_{H\text{-}H}$ = 8.0 Hz, 2H), 2.57 (s, 3H), 2.34 – 2.31 (m, 2H), 2.27 – 2.15 (m, 2H), 2.06 – 1.95 (m, 1H), 1.95 – 1.83 (m, 2H), 1.77 – 1.70 (m, 1H); ^{13}C NMR (126 MHz, DMSO-d_6): 174.1 (C), 173.9 (C), 171.3 (C), 171.0 (C), 166.8 (2C), 163.8 (dd, $^1J_{C\text{-}F}$ = 257.4, $^3J_{C\text{-}F}$ = 12.7 Hz, 2C), 161.7 (dd, $^1J_{C\text{-}F}$ = 257.4, $^3J_{C\text{-}F}$ = 12.7 Hz, 2C), 155.6 (C), 155.3 (d, $^2J_{C\text{-}F}$ = 6.3 Hz, 2C), 155.2 (C), 155.0 (C), 152.6 (C), 150.2 (CH), 149.9 (CH), 145.4 (2CH), 145.1 (C), 137.6 (2CH), 133.9 (C), 131.4 (CH), 129.6 (CH), 128.9 (CH), 128.2 (2CH), 127.5 (2CH), 126.4 (2C), 126.0 (CH), 125.2 (CH), 124.5 (q, $^2J_{C\text{-}F}$ = 35.4 Hz, 2C), 123.7 (CH), 123.6 (CH), 122.6 (CH), 121.9 (q, $^1J_{C\text{-}F}$ = 272.2 Hz, 2C), 114.0 (t, $^2J_{C\text{-}F}$ = 15.1 Hz, 2C), 99.6 (t, $^2J_{C\text{-}F}$ = 27.7 Hz, 2C), 68.6 (CH_2), 68.5 (CH_2), 53.2 (CH), 51.8 (CH), 49.2 (CH_2), 38.4 (CH_2), 34.1 (CH_2), 30.5 (CH_2), 30.0 (CH_2), 27.3 (CH_2), 26.7 (CH_2), 25.2 (CH_2), 20.9 (CH_3); ^{19}F NMR (282 MHz, DMSO-d_6): -61.5 (CF_3), -61.7 (CF_3), -103.3 (dd, J = 11.8, 8.7 Hz, 2F), -106.8 (dd, J = 12.0, 5.3 Hz, 2F); HRMS (ESI, +ve) calcd for $C_{60}H_{52}F_{10}IrN_{10}O_8$ $[M]^+$: 1423.3444, found: 1423.3413.

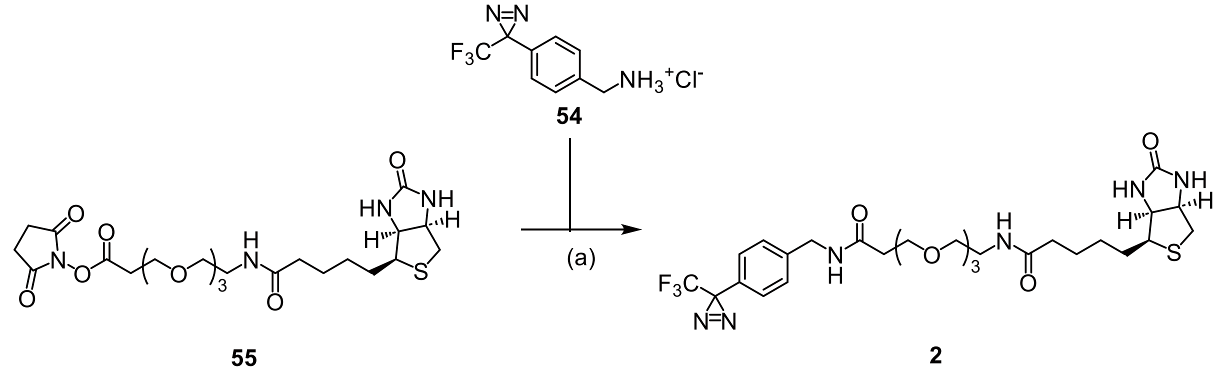

Scheme S6. (a) DIPEA, DMF (0.2 M), RT, 18 h, 92%.

Compound 2. To a solution of **54** (100 mg, 0.4 mmol) in dry DMF (2 mL) was added in one portion **55** (216 mg, 0.4 mmol) and DIPEA (138 µL, 0.8 mmol) in a 4 mL vial. The mixture was purged with N_2 for 15 minutes and left stirring at RT for 18 h (completion evidenced by LC-MS). The reaction mixture was concentrated under high vacuum to a volume of ≈500 µL of DMF. The solution was subjected to purification by RP chromatography (Scorpius C18, 33g, H_2O + 0.1% TFA

/ CH_3CN + 0.1% TFA, linear gradient 10 to 100% of CH_3CN + 0.1% TFA) afforded **2** (235 mg, 92%) as a colorless solid. The spectroscopic data were in accordance with the ones reported reference S14.

4. TNBSA Assay

Calibration. In different Eppendorf tubes, 100 µL of Sav (WT) at different concentration (0.15 – 3.0 µM in bicarbonate buffer) were prepared. To each solution, TNBSA was added (50 µL of a 0.01% solution in 0.1 M bicarbonate buffer – pH 8.5) and the mixture was shaken at 37 °C for 2 h. Then, SDS (50 µL of a 10% solution in bidistilled water) and HCl (25 µL of a 1 M solution in bidistilled water) were added. The UV-Vis spectrum of the mixture was directly recorded and the absorbance at 335 nm was plotted against the concentration of free lysines in solution ($c_K = 16 \times c_{WT}$), affording calibration line of Figure S4b (circles).

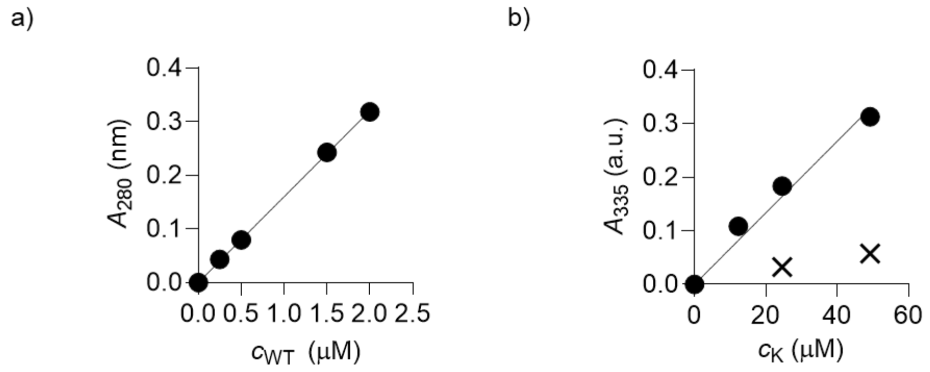

Figure S4. a) Absorbance at λ = 280 nm of **21**, at increasing concentrations of WT. b) Absorbance at λ = 335 nm of **21** (filled circles) or **22** (crosses) depending on the concentration of lysines residues, upon TNBSA treatment. All the UV spectra were recorded in a quartz cuvette with optical path l = 0.3 cm.

5. Stability in Aqueous Medium

Stock solutions of 2 – 5 mM of photocatalysts were prepared in DMSO and stored at -20 °C for short period of storage (< 3 months) or at -80 °C for longer period of storage (< 12 months). The concentration was each time validated by UV/vis using **24**'s extinction coefficient ε = 5300 $M^{-1}cm^{-1}$ at ~ 450 nm, and, if necessary, re-adjusted. **pc-CAX** (5 mM, DMSO) was diluted in water (1 mL) to give a final concentration of 100 µM. The solution was stored at RT and analyzed by HPLC at 15, 30, 60, 120 min and 1 day (using LC-MS conditions as described in section 1).

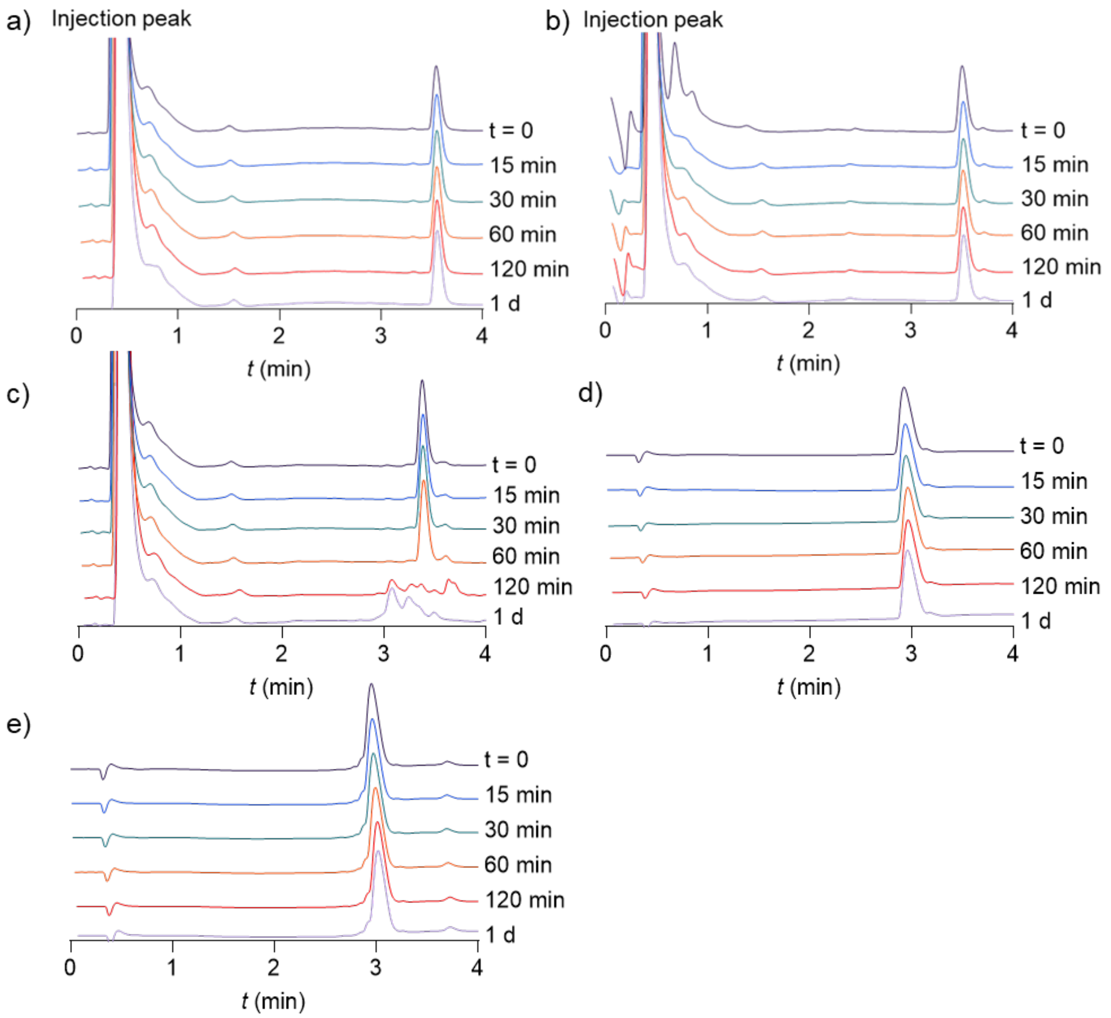

Figure S5. LC-MS traces over time of 100 µM of a) **1**, b) **3**, c) **4**, d) **5** and e) **6** in water. Note: the samples were left at RT in the dark. Most of them remain stable even after 1 day, except for **4** which precipitated after 1 h. Detection at 220 nm for **1**, **3** and **4**; detection at 280 nm for **5** and **6**.

6. Spectroscopic Properties

In H_2O. Stock solutions of **1**, **3**, **4** – **6** and **24** (5 mM in DMSO) were diluted to give a 10 µM solution in H_2O, and the absorption and emission spectra (excitation at 410 nm, slits 1.5; *l* = 1.0 cm) were then recorded and reported in Figure S6.

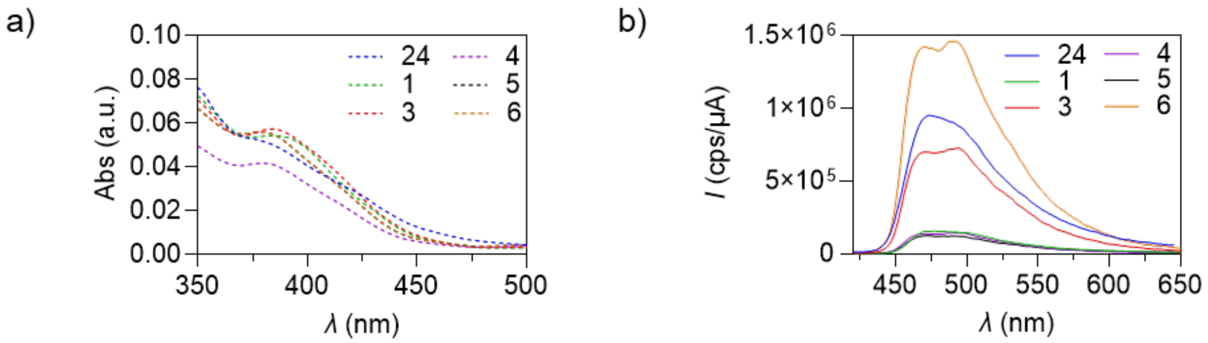

Figure S6. a) Absorption (dashed) and b) emission (solid) spectra of 10 µM of **24** and **pc-CAX**s (**1**, **3**, **4-6**) in water upon excitation at 410 nm. Note: presence of cyclic disulfide quenches the luminescence.

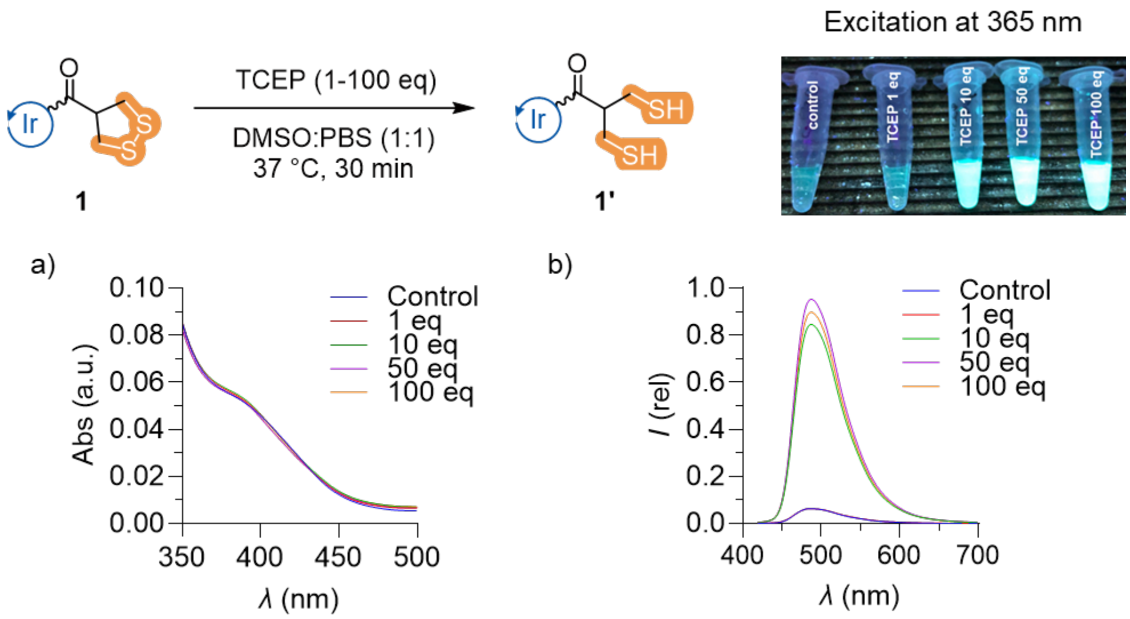

Figure S7. a) the absorption spectra of **1** with increasing concentration of TCEP.HCl (1–100 eq.) in DMSO:PBS 1:1 and b) the corresponding emission spectra upon irradiation at 410 nm (slits 1.5; *l* = 1.0 cm).

7. Cell Culture

Human cervical cancer-derived HeLa Kyoto, human epidermoid carcinoma (A431) and human retinal pigment epithelial-1 (RPE-1) cells were cultured in complete DMEM (GlutaMAX, 4.5 g/L D-glucose), which contains 10% fetal calf serum (FCS) and 1% Penicillin/Streptomycin (PS). The cells were grown under 5% CO_2 humidified atmosphere at 37 °C on a 75 cm^3 tissue culture flask (TPD Corporation). Cells were harvested by treatment with 3 mL of phenol-red free TrypLE Express, followed by the addition of 10 mL of complete FDMEM (GlutaMAX, 4.5 g/L D-glucose) containing 10% fetal calf serum (FCS) and 1% Penicillin/Streptomycin (PS) at 37 °C. The cells were spun down at 1500 g for 3 min, re-suspended in complete FDMEM (GlutaMAX, 4.5 g/L D-glucose) medium, and plated according to the concentration needed. For uptake or inhibition experiments, the cells were seeded in a μ-Plate 96-well Black ibiTreat sterile at 12 000 cells/well in complete FDMEM and left incubating under 5% CO_2 humidified atmosphere at 37 °C overnight.

8. Lysate Preparation

Cell lysates were prepared by treating the cells (10^6/well) in a 6 well-plate for 15 min at 4 °C with a RIPA lysis buffer (1X, Millipore, 20-188) supplemented with cOmplete™ EDTA-free protease inhibitor cocktail (Sigma-Aldrich 4693159001) and PMSF (1 mM, Sigma-Aldrich 52332). The lysate was collected by scraping the surface of the well. After centrifugation at 14.5 x 1000 g for 15 min at 4 °C, the supernatant was collected. The protein concentration was determined using the BCA assay (Thermo Scientifics 23225) and adjusted with a solution of RIPA 1X to a final concentration of either 500 µg/mL or 1 mg/mL. The protein lysate was either used immediately, frozen at -20 °C for experiments within the next few days, or stored at -80 °C for experiments in the following weeks.

9. Western Blot Analyses

The protein lysate (50 µL, 1 mg/mL) was pre-mixed with a reducing loading buffer (Laemmli buffer 4x, Bio-Rad 1610747) for 10 min at 95 °C. The lysate samples (20–30 µg per well) were loaded onto a precast PAGE gel (4-20% gradient, Bio-Rad 4561096), and the proteins were separated by electrophoresis at 200 V for 1 h. The proteins were transfered to a PVDF membrane using the iBlot 3 Western Blot Transfer System (Thermo Fisher Scientific). The proteins were first revealed using a ponceau S solution for 15 min at RT. Then, the membrane was blocked by incubating with a solution of 1% BSA in TBST for 20 min at RT. Streptavidin Alexa Fluor 670 conjugate (10 mL, 4 µg/mL) in TBST was incubated with the membrane at 4 °C overnight. The membrane was washed 3 times with TBST buffer (5 min each time) and then fluorescence was visualized with a Fusion Solo (Vilber Lourmat) using the Cy-5 channel.

10. Cellular Uptake of PCs

10.1. General Experimental Procedure

The procedure described in reference S1 for the uptake of **fl-CAXs** was adapted for **pc-CAXs**. Cells were prepared in a 96 well plate as described in section 7, then medium was removed, and cells were washed with PBS (3 × 3 mL/well) followed by fresh FDMEM serum-free medium (4 × 100 µL/well) using a plate washer (Biotek EL406®), and kept in a 100 µL of the latter medium. The solution of **pc-CAX** (10 mM, DMSO) was diluted in FDMEM to give a solution at 3x final concentration (1 – 50 µM), of which 50 µL was added to the well resulting in a final volume of 150 µL per well. The cells were incubated under 5% CO_2 humidified atmosphere at 37 °C for the indicated time (5 – 240 minutes). Afterward, to remove the excess of reporter, the cells were washed with PBS and the medium was exchanged with FDMEM keeping a final volume of 100 µL/well, and a solution of SYTO deep red (2 mM in DMSO, working concentration of 1 µM) in PBS (50 µL/well) was added. After 30 min of incubation under 5% CO_2 humidified atmosphere at 37 °C, cells were washed with PBS (3 × 3 mL/well) and kept in FDMEM (100 µL/well) for live cell imaging. The distribution of fluorescent signals was captured on a IXM-C automated microscope with two channels, blue/green for **pc-CAX** (377/50 nm excitation filter; 536/40 nm emission filter) and far red for SYTO deep red (620/50 nm; emission filter: 690/50 nm). The rest of the parameters were adjusted according to the nature of the experiment. The same experiments were performed in Leibovitz L15 or DMEM/Hepes. Technical duplicates were performed for each condition.

10.2. Data Analysis

Images from experiments with **pc-CAXs** were automatically analyzed and quantified using the slightly modified protocol described in reference S1. Briefly, the nuclei and cell bodies were segmented using the red channel image (SYTO deep red). Dividing and dying cells were detected based on their shape factor (round shape, > 0.8) and filtered out. Bright aggregates resulting from precipitation were segmented and filtered out based on the maximum signal intensity and their size. The resulting objects were then grown, and all cells in the vicinity were removed from the analysis. All the cells touching the border of the image were removed to prevent inaccurate quantification in the final mask (Figure S8). Finally, the luminescence of the blue/green channel was quantified as the average luminescence intensity per pixels I_{cell} in live cells (yellow mask) minus the average intensity I_{bg} of the background (blue mask) to give average luminescence intensity in cells $I_{\text{pc-CAX}}$.

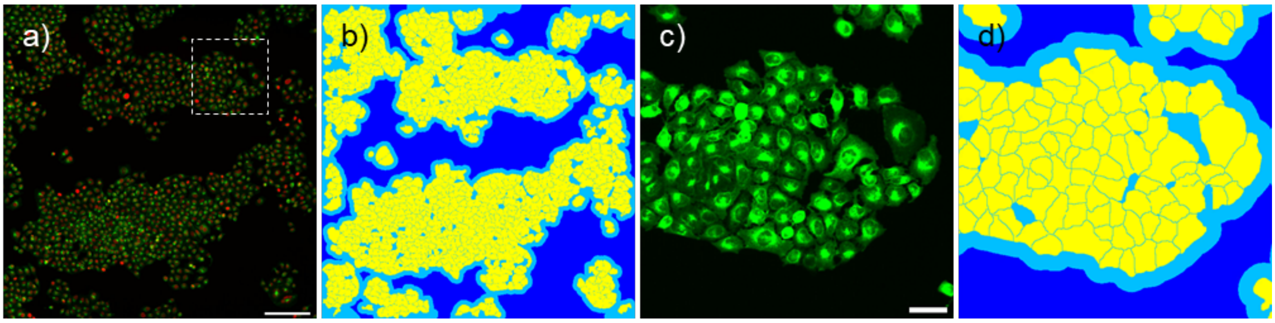

Figure S8. a) Widefield images (10X) of HK cells treated with pc-AspA **1** (10 µM, green) and b) the respective final mask: yellow – cells used for quantification; cyan –small inter-cellular space; dark blue – background. c) The zoomed region from (a) with increased brightness and d) the respective mask. (red: SYTO deep red, nuclei; scale bar 200 µm for a) and 50 µm for c)).

10.3. Optimization of Uptake

10.3.1. Cellular Uptake Pattern

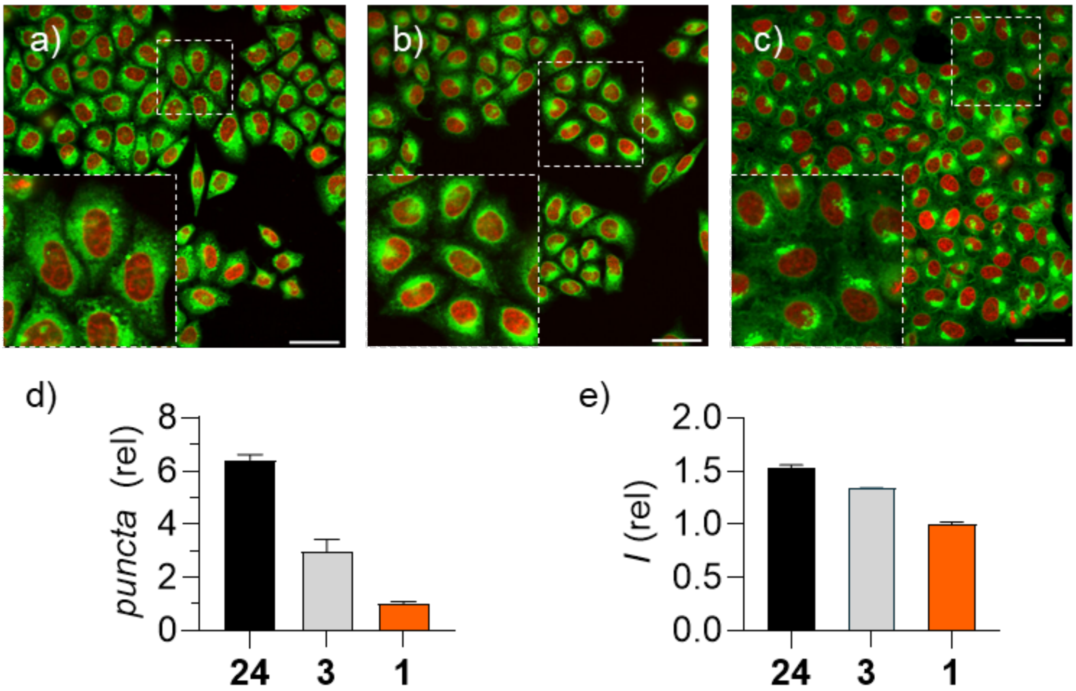

Figure S9. SDCM images (40X WI, confocal) of HK cells after incubation for 30 min with a) **24** (5 µM, green), b) **3** (5 µM, green) or pc-AspA **1** (5 µM, green) (inset; zoom; Red: SYTO deep red, nuclei; scale bar 50 µm). d) Relative punctum number ± SEM and e) relative fluorescence intensity *I* (rel) ± SEM of **24**, **3** and pc-AspA **1** in HK cells.

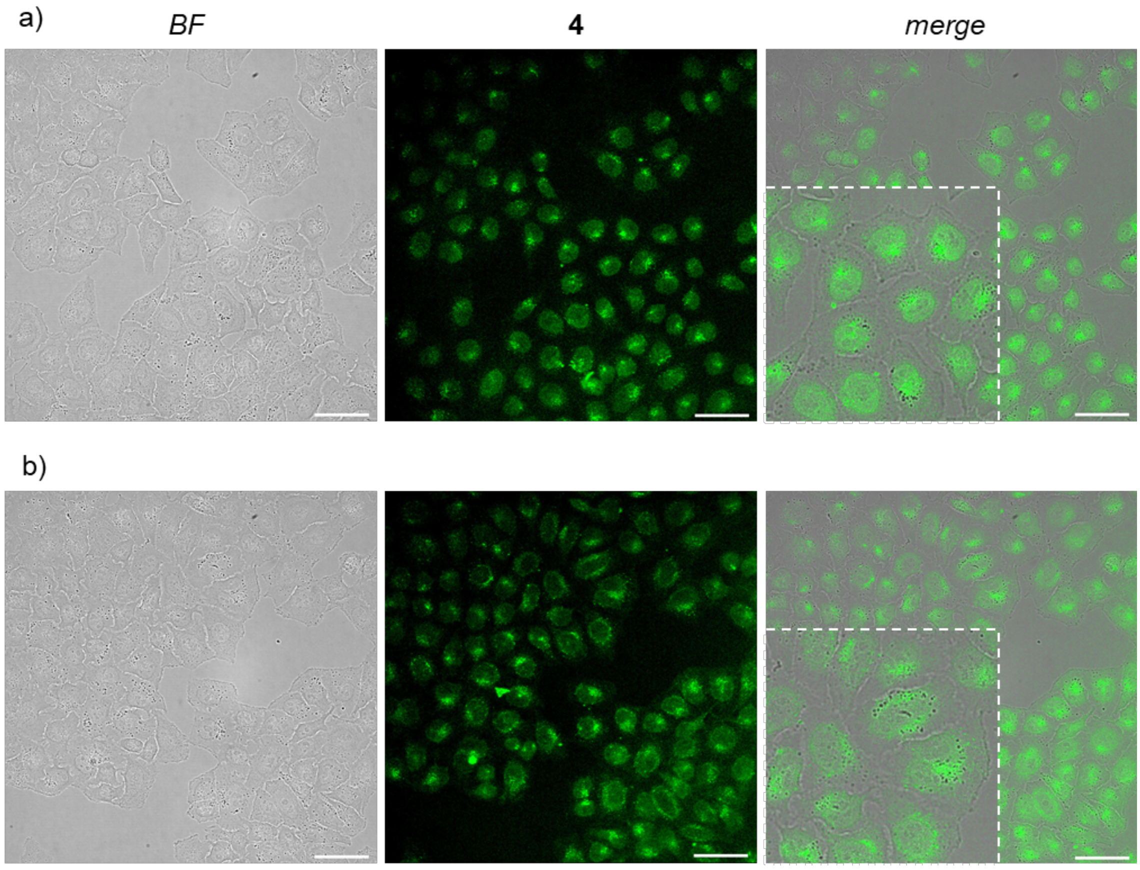

Figure S10. SDCM images (40X WI, confocal) of HK cells after incubation for a) 10 min or b) 30 min with **4** (1 μM, green) (inset; zoom; scale bar 50 μm). At initial time points, pc-ETP **4** labels the nucleus, however, after prolonged incubation time, the pc spreads in the cytosol. In accordance with the results reported with Fl-ETP **11**.[S7]

10.3.2. Results for Uptake of pc-CAXs in HK Cells

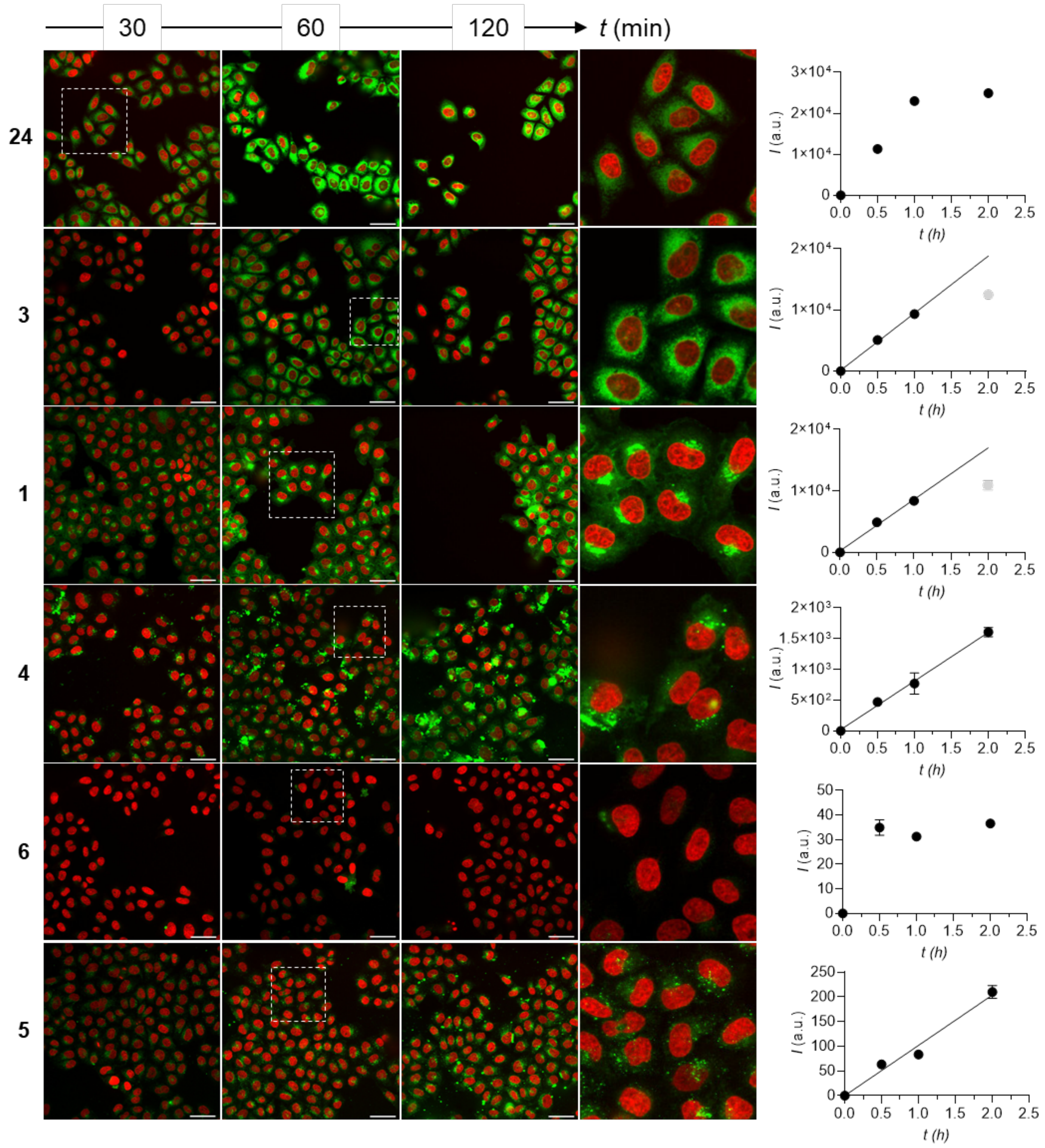

Figure S11. a) SDCM images (40X WI, confocal) of HK cells after 30, 60, or 120 min incubation and a zoomed region (left image) with 10 µM of (from top to bottom) **24**, **3**, **1**, **4**, **6**, **5** and their respective fluorescence intensity $I \pm$ SD. Note: the fluorescence coming from the precipitates in **4** were removed using the mask described in section 10.2 (red: SYTO deep red, nuclei; scale bar 50 µm).

10.4. Cellular Uptake of Fl-Sav-AspA

Cells preparation. For the uptake of the complexes **13** and **23**, HK cells were seeded in a 35 mm glass bottom round dishes (MatTek dishes) at 80 000 cells/mL in complete FDMEM and left incubating under 5% CO_2 humidified atmosphere at 37 °C overnight. On the day of the experiment, the medium was removed, cells were washed with Leibovitz L15 medium (3 × 1 mL) and treated with a solution of the corresponding complex (**13** or **23**, 5 μM) in L15 for 6 h at 37 °C under 5% of CO_2. Afterward, the medium was removed, and cells were rinsed with L15 (3 × 1 mL) and kept in fresh L15 (1 mL) for live imaging.

Confocal laser scanning microscopy (CLSM). Imaging was performed without fixation using a Leica Stellaris 8 FALCON confocal laser scanning microscope at 20 MHz, with an excitation wavelength of 488 nm (white light laser). The fluorescence was measured between 499 and 600 nm (laser power 13%). During the imaging, the samples were kept at 37 °C. Brightness and contrast were adjusted equally in all images using ImageJ software.

Fluorescence lifetime imaging microscopy (FLIM). Samples were prepared as described for CLSM experiment and imaged with a Leica Stellaris 8 FALCON, at 20 MHz, with λ_{ex} = 488 nm (white light laser). For the analysis, Leica LAS X FLIM/FCS software (4.5.0) was used, and lifetimes were estimated by manually selecting the center of photon clouds in phasor plots. Obtained lifetime of **13** in HK cells was τ = 2.20 ± 0.01 ns, almost identical to that of **13** in L15 medium (2.12 ± 0.01 ns) and different from **29** (3.30 ± 0.01 ns), demonstrating that the fluorescence in HK cells arises from the intact **13** and not from the degradation product.

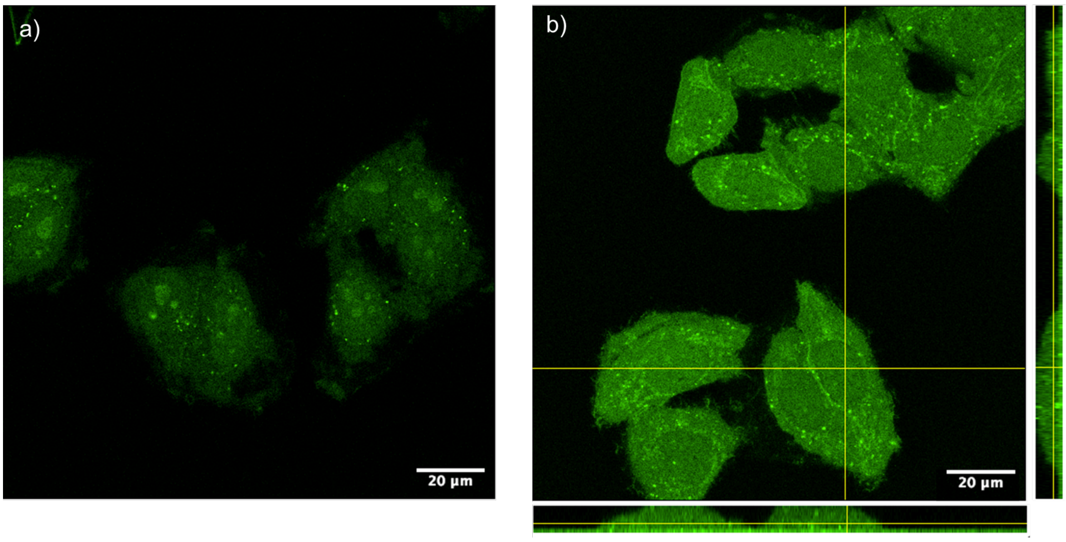

Figure S12. CLSM images of HK cells after incubation with a) **23** (5 µM) and b) **13** (5 µM) for 6 h in L15 medium. Orthogonal views are reported for **13** as additional proof of CPS internalization. Brightness and contrast were equally adjusted by using ImageJ software.

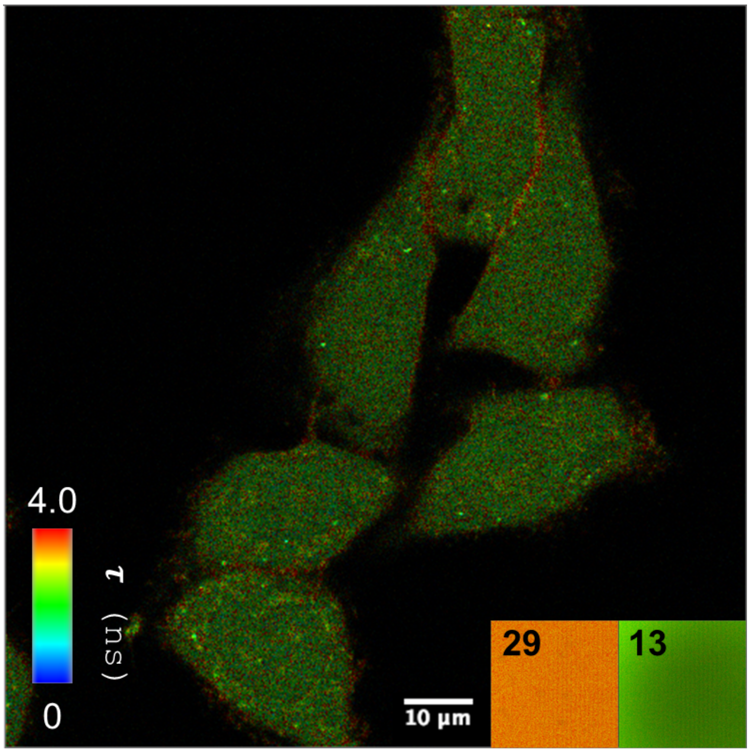

Figure S13. FLIM images of HK cells after incubation with **13** (5 µM, $\tau = (2.20 \pm 0.01)$ ns). Inset: FLIM images of **29** (5 µM, $\tau = (3.30 \pm 0.01)$ ns) and **13** (5 µM, $\tau = (2.12 \pm 0.01)$ ns) in L15 medium. Lifetimes are given as average ± SD.

10.5. Cytotoxicity

The relative cell viability (*RV*) of different **pc-CAXs** (10 µM) in HK cells was measured by comparing the number of live cells, based on their shapes and PI staining, after treatment with each probe to the same with DMSO (control).

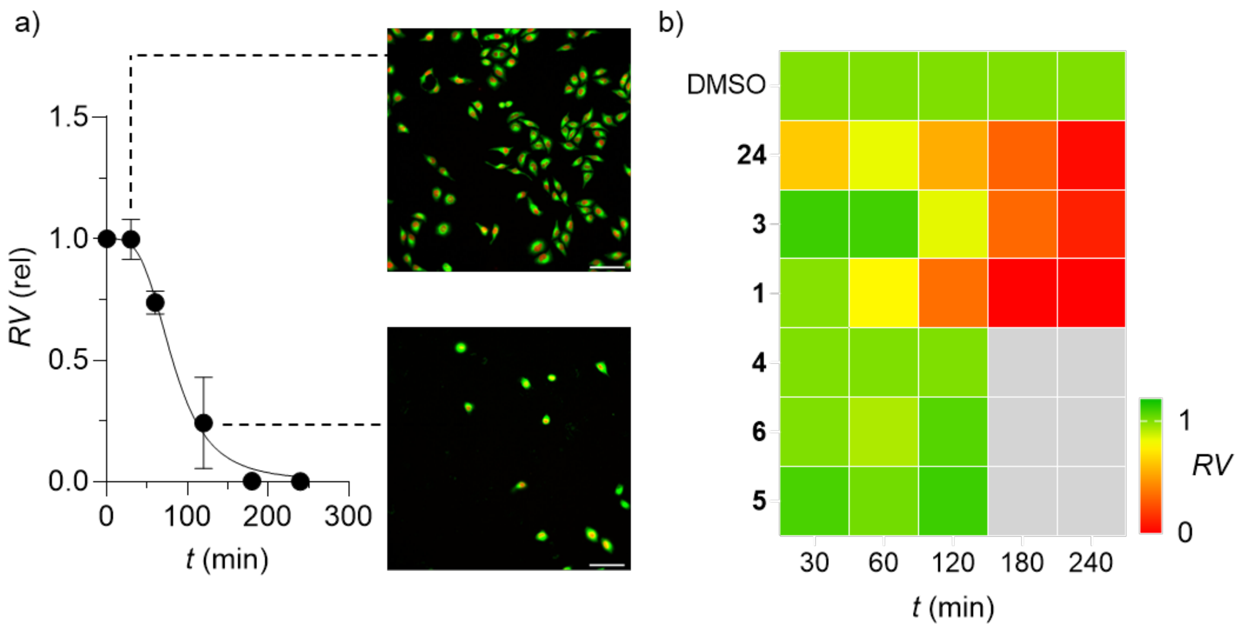

Figure S14. a) Relative cell viability *RV* ± SEM of **1** (10 µM) as a function of the time in HK cells with the corresponding SDCM images (20X WI, green: pc-AspA **1**, red: SYTO deep red, scale bar 100 µm) at 30 min (top) and 120 min (bottom). b) Heatmap of relative cell viability of different **pc-CAXs** (10 µM). Grey: not measured.

11. AHCHT TMU Inhibitor Screening

11.1. General Experimental Procedure

Following the procedure described in reference S1 with some modifications: the cells were prepared in a 96-well plate as described in section 7, then medium was removed, and cells were washed with PBS (3 × 3 mL/well) followed by fresh FDMEM (4 × 100 µL/well) using a plate washer (Biotek EL406®), and kept in 100 µL/well of the latter medium. Stock solutions of the inhibitors (3×

final concentration), transporters (10× in FDMEM), and SYTO deep red (3 μM in FDMEM) were prepared freshly in a 96-well V-bottom plate before adding to the cells. The inhibitor solutions from the V-bottom plate were added using an electronic multichannel pipette to the cells (50 μL/well) keeping a final volume of 150 μL/well and cells were incubated for 1 h under 5% CO_2 humidified atmosphere at 37 °C. Then, the corresponding transporter from the V-bottom plate was added (15 μL/well) to the cells, giving a final volume of 165 μL/well and a final transporter concentration of 5-10 μM. The cells were incubated for an additional 60 min with **pc-CAX** under 5% CO_2 humidified atmosphere at 37 °C. After that, the cells were washed with PBS (3 × 3 mL/well) and FDMEM (4 × 100 μL/well) using the plate washer. A solution of SYTO deep red (1 μM) was added (50 μL/well) to the cells and left incubating for 30 min under 5% CO_2 humidified atmosphere at 37 °C. After indicated time, the cells were washed with PBS (9 × 3 mL/well) and kept in FDMEM for imaging. During live cell imaging, samples were kept at under 5% CO_2 atmosphere at 37 °C. The distribution of fluorescent signals was captured on a IXM-C automated wide-field fluorescence microscope acquiring 4 images per well using a 10× or 20× objective lens with 2 channels, *i.e.* blue/green for PC (377/50 nm excitation filter, 536/40 nm emission filter) and far red for SYTO deep red (excitation filter: 620/50 nm; emission filter: 690/50 nm). Technical duplicates were performed for each condition.

11.2. Data Analysis

Resulting images were automatically analyzed and quantified using the protocol described in reference S1.

11.3. Results for Inhibitors Screening in HK Cells

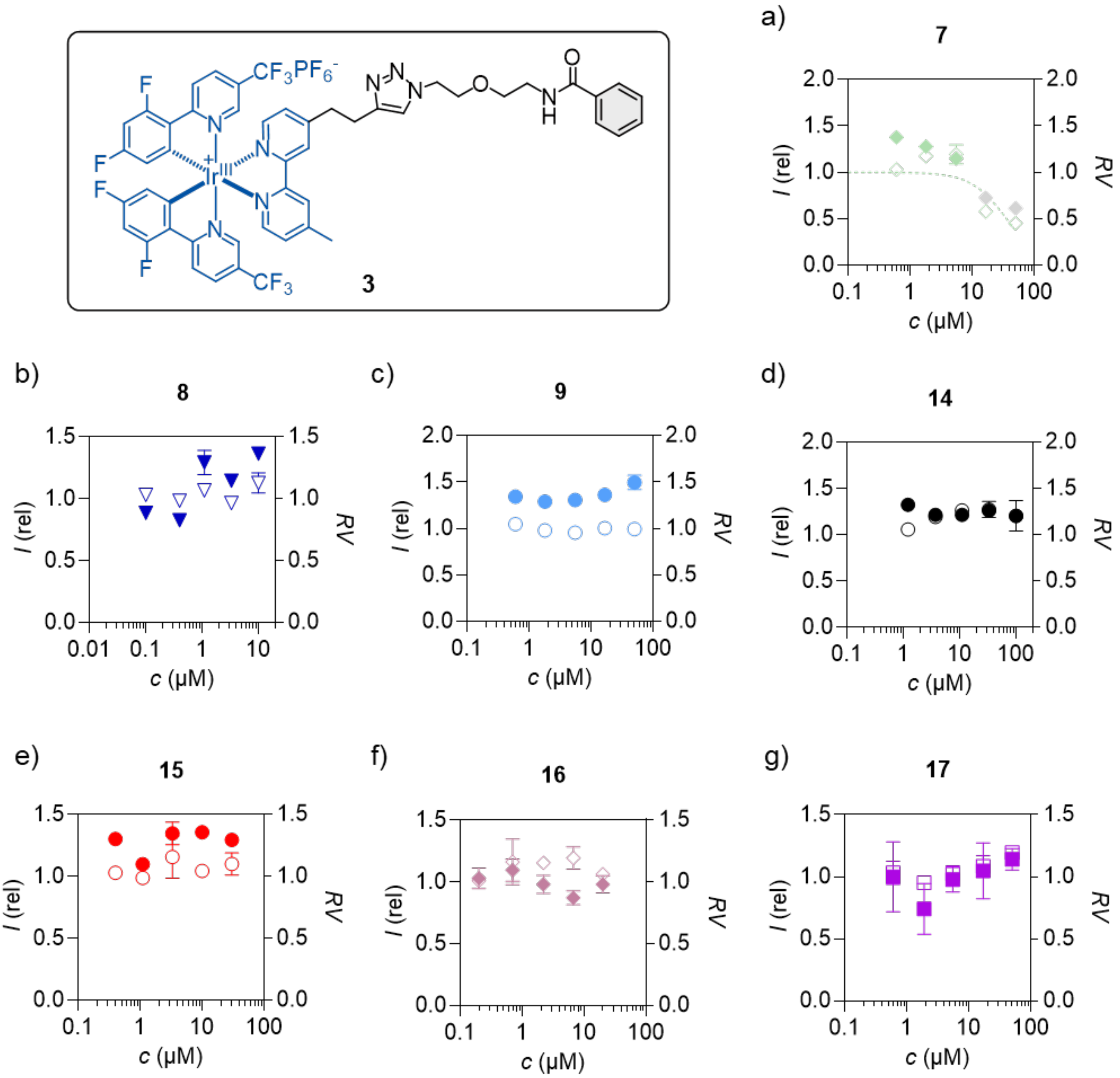

Figure S15. Relative luminescence *I* (rel) ± SEM (filled symbols) of **3** (10 μM) in HK cells and relative viability *RV* ± SEM (empty symbols) as a function of the concentration of a) **7**, b) **8**, c) **9**, d) **14**, e) **15**, f) **16** and g) **17**.

Table S1. Dependence of cellular uptake of **3** and the respective cell viability in HK cells on the concentration of CAX inhibitors.[a]

Entry	T[b]	I[c]	MIC (μM)[d]	IC_{50} (μM)[e]	*n* (IC_{50})[f]	RV_{50} (μM)[g]	*n* (RV_{50})[h]
1	**3**	**7**	-	>>10	-	35 ± 10	1.5 ± 0.6
2	**3**	**8**	-	>>10	-	>10	-
3	**3**	**9**	-	>>50	-	>50	-
4	**3**	**14**	-	>>100	-	>100	-
5	**3**	**15**	-	>>30	-	>30	-
6	**3**	**16**	-	>>30	-	>30	-
7	**3**	**17**	-	>>50	-	>50	-

[a]Results from dose-response curves in Figure S15. [b]Transporter. [c]Inhibitor. [d]Concentration needed to reach 15% inhibition. [e]Concentration needed to reach 50% inhibition. [f]Hill coefficient for inhibition of cellular uptake. [g]Concentration needed to lower relative viability (*RV*) by 50%. [h]Hill coefficient for cell viability.

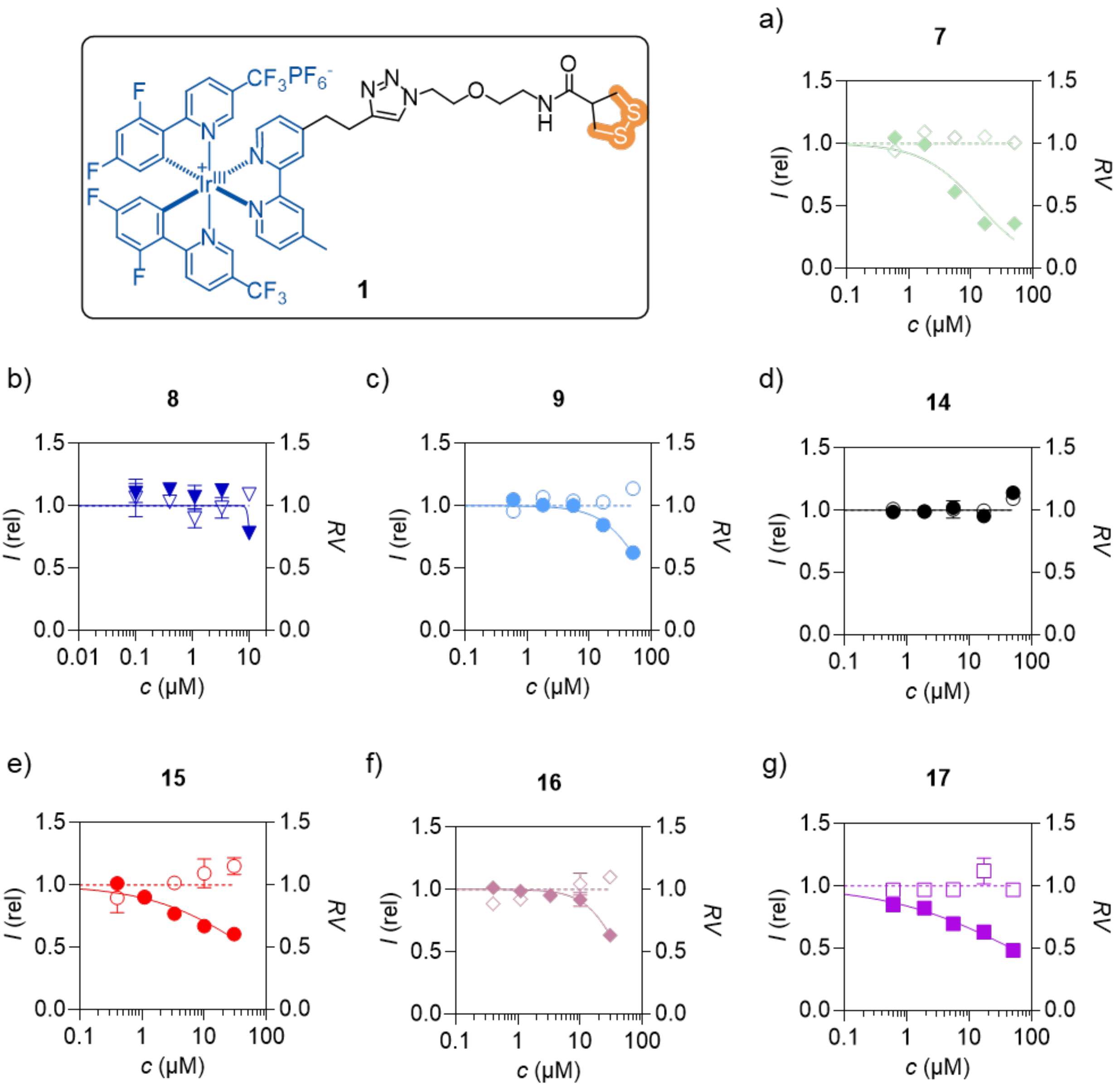

Figure S16. Relative fluorescence intensity *I* (rel) ± SEM (filled symbols) of **1** (10 µM) in HK cells and relative viability *RV* ± SEM (empty symbols) as a function of the concentration of a) **7**, b) **8**, c) **9**, d) **14**, e) **15**, f) **16** and g) **17**.

Table S2. Dependence of cellular uptake of **1** (pc-AspA) and the respective cell viability in HK cells on the concentration of CAX inhibitors.[a]

Entry	T[b]	I[c]	MIC (μM)[d]	IC_{50} (μM)[e]	*n* (IC_{50})[f]	RV_{50} (μM)[g]	*n* (RV_{50})[h]
1	**1**	**7**	2	14 ± 3	0.9 ± 0.2	>50	-
2	**1**	**8**	9	>10	-	>10	-
3	**1**	**9**	19	(70) ± 10	1.3 ± 0.2	>50	-
4	**1**	**14**	-	>>100	-	>100	-
5	**1**	**15**	2	(50) ± 15	0.5 ± 0.1	>30	-
6	**1**	**16**	14	(43) ± 4	1.6 ± 0.3	>30	-
7	**1**	**17**	1	(50) ± 25	0.4 ± 0.3	>50	-

[a]Results from dose-response curves in Figure S16. [b]Transporter. [c]Inhibitor. [d]Concentration needed to reach 15% inhibition. [e]Concentration needed to reach 50% inhibition. Values in parentheses were extrapolated. [f]Hill coefficient for inhibition of cellular uptake. [g]Concentration needed to lower relative viability (*RV*) by 50%. [h]Hill coefficient for cell viability.

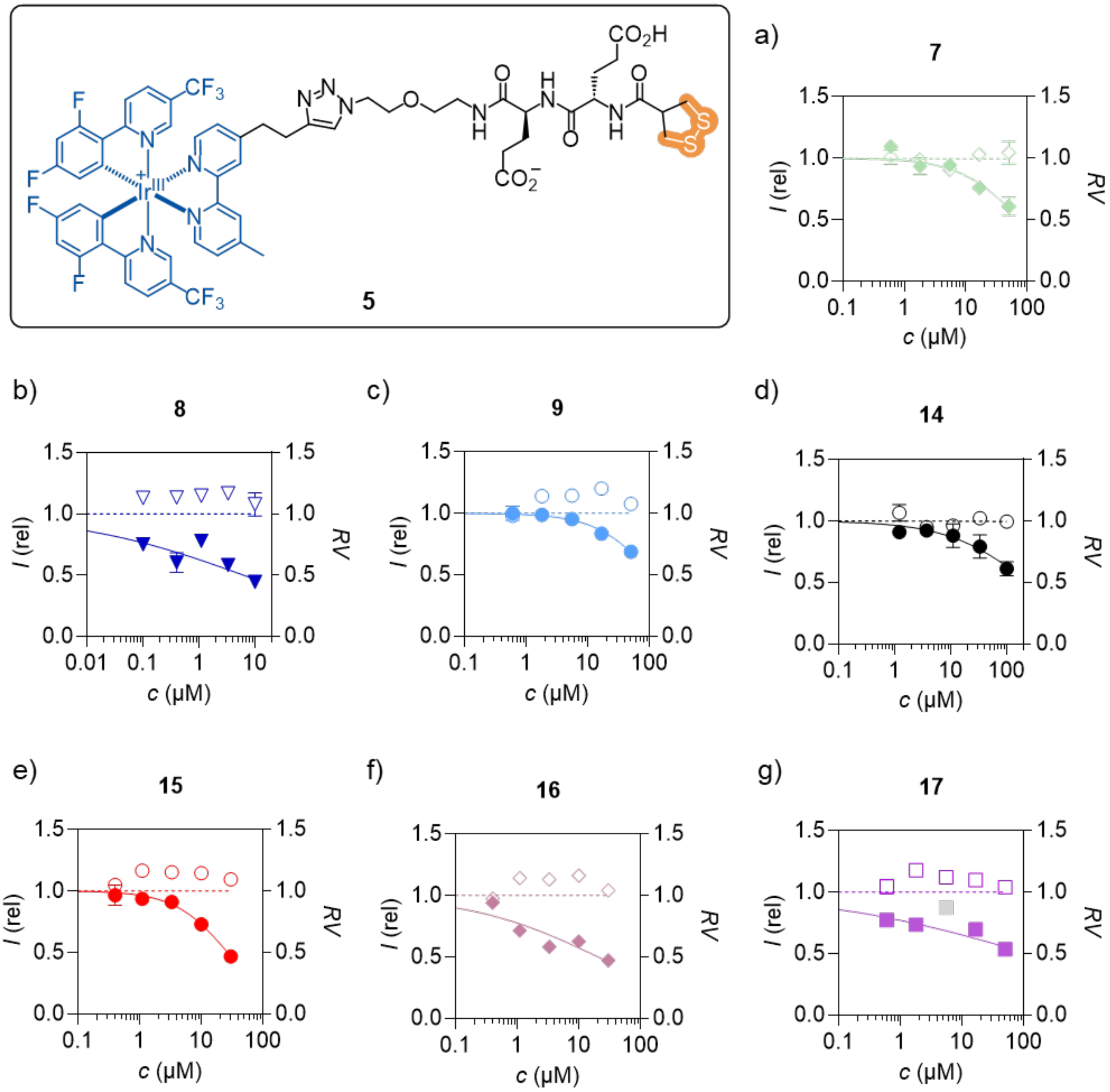

Figure S17. Relative fluorescence intensity *I* (rel) ± SEM (filled symbols) of **5** (10 µM) in HK cells and relative viability *RV* ± SEM (empty symbols) as a function of the concentration of a) **7**, b) **8**, c) **9**, d) **14**, e) **15**, f) **16** and g) **17**.

Table S3. Dependence of cellular uptake of **5** and the respective cell viability in HK cells on the concentration of CAX inhibitors.[a]

Entry	T[b]	I[c]	MIC (μM)[d]	IC_{50} (μM)[e]	n (IC_{50})[f]	RV_{50} (μM)[g]	n (RV_{50})[h]
1	**5**	**7**	11	(75) ± 25	0.9 ± 0.3	>50	-
2	**5**	**8**	<0.1	6 ± 4	0.3 ± 0.1	>10	-
3	**5**	**9**	18	(110) ± 30	0.9 ± 0.2	>50	-
4	**5**	**14**	15	(240) ± 140	0.6 ± 0.2	>100	-
5	**5**	**15**	5	27 ± 4	1.0 ± 0.1	>30	-
6	**5**	**16**	<0.4	21 ± 8	0.4 ± 0.1	>30	-
7	**5**	**17**	<0.6	(120) ± 60	0.3 ± 0.1	>50	-

[a]Results from dose-response curves in Figure S17. [b]Transporter. [c]Inhibitor. [d]Concentration needed to reach 15% inhibition. [e]Concentration needed to reach 50% inhibition. Values in parentheses were extrapolated. [f]Hill coefficient for inhibition of cellular uptake. [g]Concentration needed to lower relative viability (*RV*) by 50%. [h]Hill coefficient for cell viability.

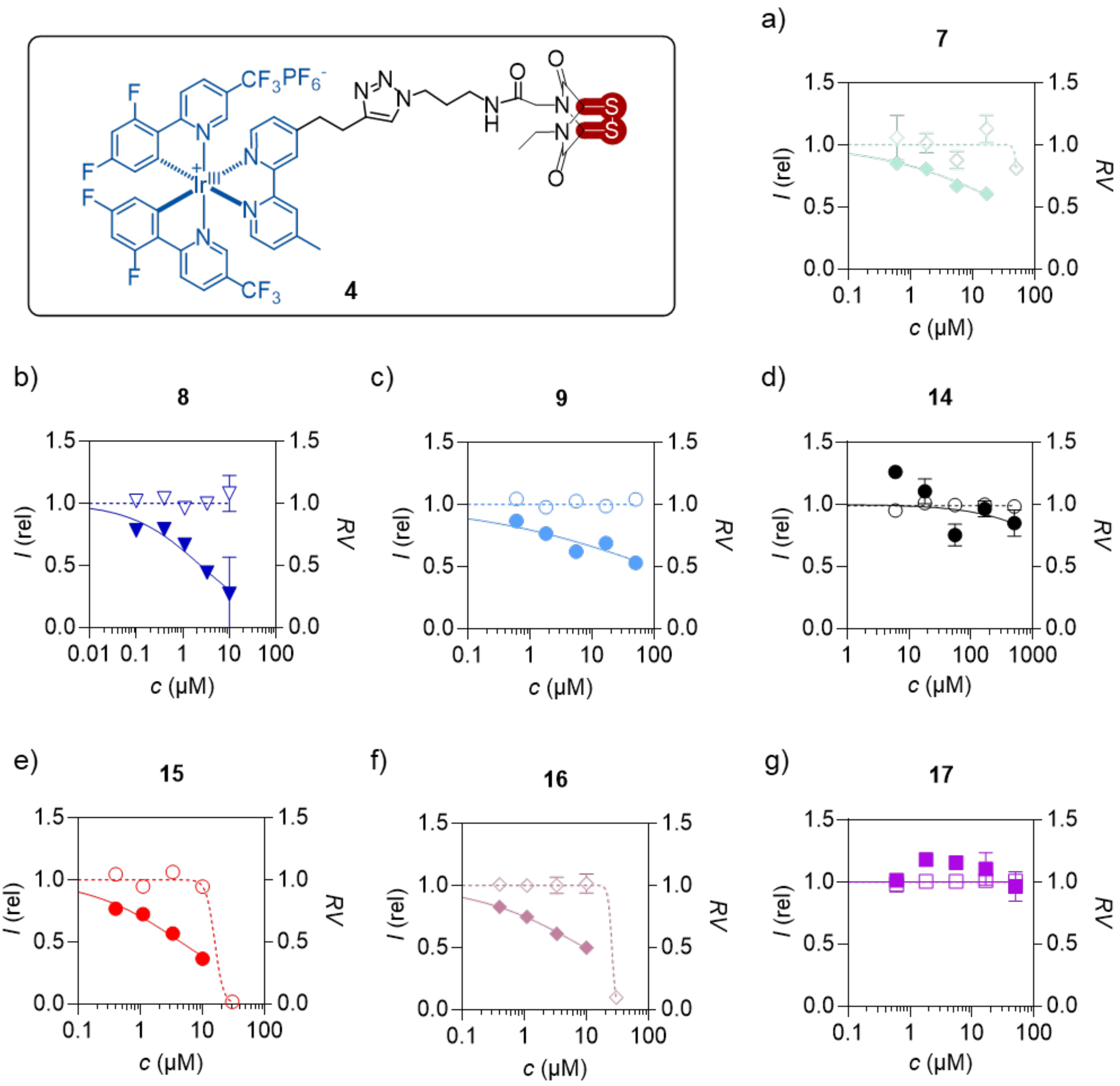

Figure S18. Relative fluorescence intensity *I* (rel) ± SEM (filled symbols) of **4** (10 μM) in HK cells and relative viability *RV* ± SEM (empty symbols) as a function of the concentration of a) **7**, b) **8**, c) **9**, d) **14**, e) **15**, f) **16** and g) **17**.

Table S4. Dependence of cellular uptake of **4** (pc-ETP) and the respective cell viability in HK cells on the concentration of CAX inhibitors.[a]

Entry	T[b]	I[c]	MIC (μM)[d]	IC_{50} (μM)[e]	*n* (IC_{50})[f]	RV_{50} (μM)[g]	*n* (RV_{50})[h]
1	**4**	**7**	<0.6	(40) ± 10	0.4 ± 0.1	~55	-
2	**4**	**8**	0.1	2.4 ± 1.2	0.6 ± 0.2	>10	-
3	**4**	**9**	<0.6	(100) ± 50	0.3 ± 0.1	>50	-
4	**4**	**14**	-	>500	-	>500	-
5	**4**	**15**	<0.4	4.5 ± 0.5	0.6 ± 0.1	16 ± 3	6 ± 2
6	**4**	**16**	<0.4	9.4 ± 1.6	0.5 ± 0.1	~26	-
7	**4**	**17**	-	>>50	-	>50	-

[a]Results from dose-response curves in Figure S18. [b]Transporter. [c]Inhibitor. [d]Concentration needed to reach 15% inhibition. [e]Concentration needed to reach 50% inhibition. Values in parentheses were extrapolated. [f]Hill coefficient for inhibition of cellular uptake. [g]Concentration needed to lower relative viability (*RV*) by 50%. [h]Hill coefficient for cell viability.

11.4. Inhibition of Fl-SAV-AspA with TMU Inhibitors

HK cells were seeded at 2.4 × 10^4 cells/well on an 8-well plate and cultured overnight. After removing the medium, cells were rinsed with L15 medium (3 × 300 µL) and treated with 200 µL of inhibitor solutions (3 µM of BiC **8**, 100 µM of EBS **9**, both in L15 medium). The cells were incubated at 37 °C under 5% CO_2 atmosphere for 1 h. Afterward, Fl-Sav-AspA **13** was added (36 µL of a 33 µM stock solution in bidistilled H_2O, final concentration 5 µM), and cells were incubated for 4 h at 37 °C under 5% CO_2 atmosphere. Lastly, the medium was removed by aspiration, and cells were rinsed with fresh L15 (3 × 300 µL) and kept in L15 for imaging. The distribution of fluorescent compounds was analyzed without fixing by CLSM (Leica Stellaris 8), equipped with a 63x oil immersion objective lens. White light laser was used as a light source, with λ_{ex} = 488 nm, and emission range spanning from 499 to 600 nm. During CLSM measurement, samples were kept at 37 °C under 5% CO_2 atmosphere. Resulting images (Figure S19a) were analyzed by ImageJ Software and the result of the quantification is reported in Figure S19b.

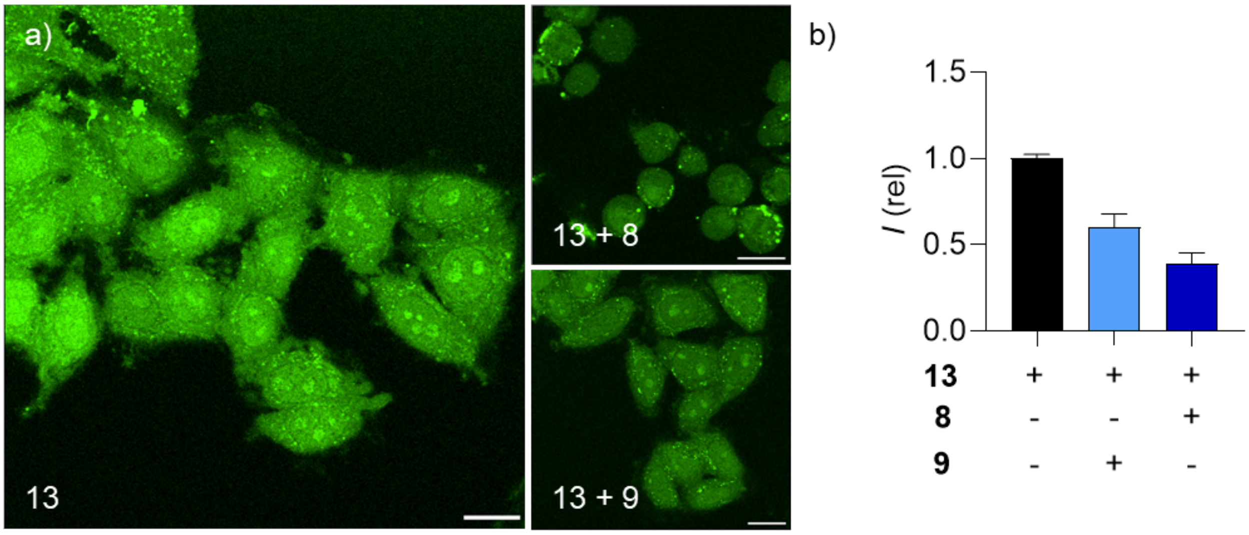

Figure S19. a) CLSM images of HK cells after incubation with CPS **13** (5 µM) in presence of **8** (3 µM) or **9** (100 µM) in L15 medium, under co-incubation condition. b) the corresponding intensity quantification (scale bar = 20 µm. Error bars represent SEM).

12. Photocatalysts Reactivity Assessment

12.1. In Organic Solvent

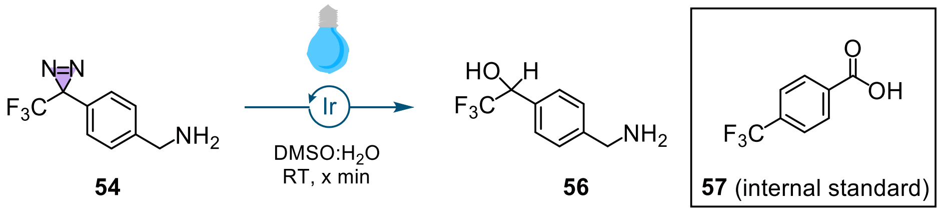

Scheme S7. Conversion of **54** into **56** in presence of **pc-CAX** upon irradiation at 450 nm.

Following the procedure described in reference S14, a solution containing diazirine **54** (100 μM), 4-(trifluoromethyl)benzoic acid (**57**) (100 μM), and photocatalyst **1**, **3-6**, **24** (10 μM) was prepared in a mixture of DMSO-d_6:D_2O (1:1, 500 μL) in an NMR tube. After purging with N_2 for 15 min, the mixture was irradiated with blue light (450 nm) for indicated period of time. Following irradiation, the samples were analyzed using ^{19}F-NMR spectroscopy (Burker 300 MHz). Conversion of diazirine **54** (δ -64.61 (3F, singlet)) was measured against internal standard (δ -61.35 (3F, singlet)) after different irradiation time. The data are reported and summarized in Figure S25. The catalytic activities of **1**, **3-6** were comparable but slightly lower than that of **24**.

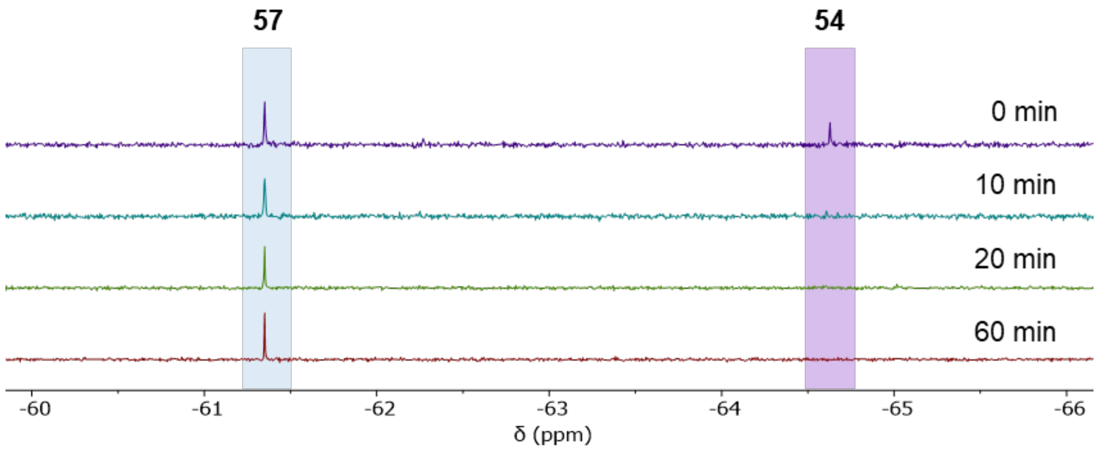

Figure S20. Quantitative ^{19}F NMR spectra of the conversion of **54** (100 μM) catalyzed by **24** (10 μM) in a mixture of DMSO-d_6:D_2O (1:1, 500 μL) after irradiation with blue light (450 nm) at different time points (top to bottom). Internal standard: 4-(trifluoromethyl)benzoic acid.

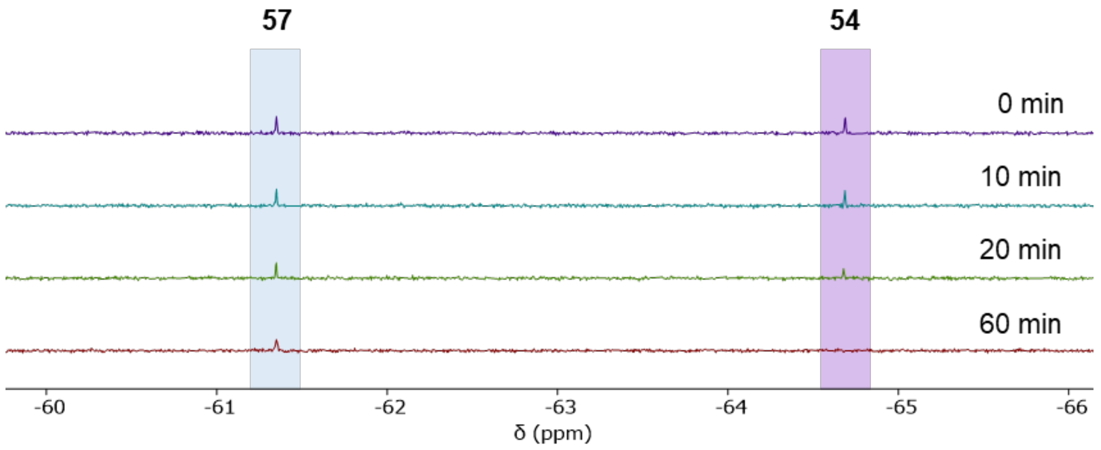

Figure S21. Quantitative ^{19}F NMR spectra of the conversion of **54** (100 µM) catalyzed by **3** (10 µM) in a mixture of DMSO-d_6:D_2O (1:1, 500 µL) after irradiation with blue light (450 nm) at different time points (top to bottom). Internal standard: 4-(trifluoromethyl)benzoic acid.

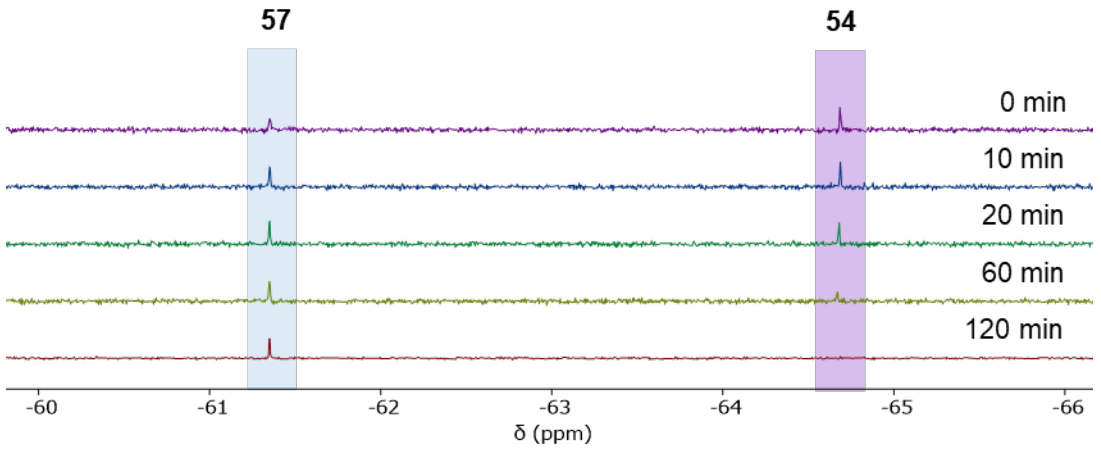

Figure S22. Quantitative ^{19}F NMR spectra of the conversion of **54** (100 µM) catalyzed by **1** (10 µM) in a mixture of DMSO-d_6:D_2O (1:1, 500 µL) after irradiation with blue light (450 nm) at different time points (top to bottom). Internal standard: 4-(trifluoromethyl)benzoic acid.

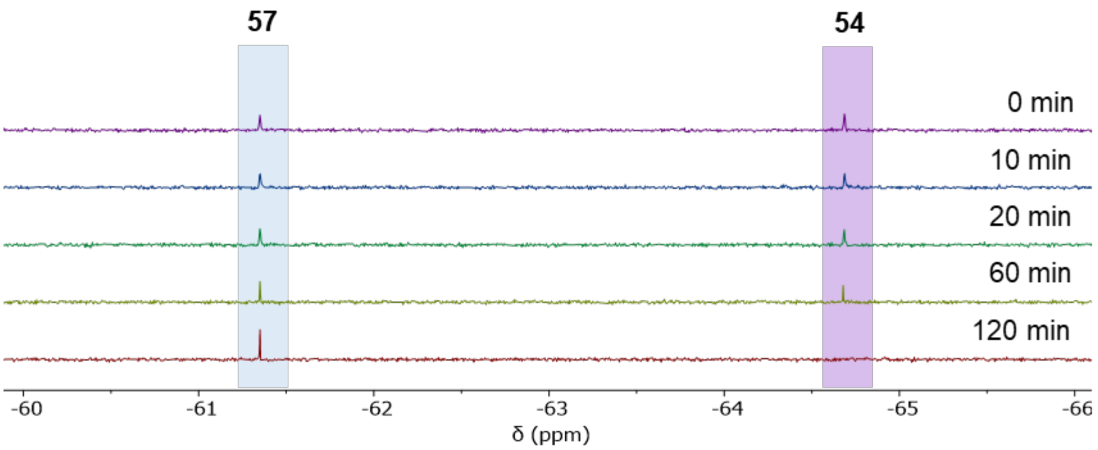

Figure S23. Quantitative ^{19}F NMR spectra of the conversion of **54** (100 µM) catalyzed by **4** (10 µM) in a mixture of DMSO-d_6:D_2O (1:1, 500 µL) after irradiation with blue light (450 nm) at different time points (top to bottom). Internal standard: 4-(trifluoromethyl)benzoic acid.

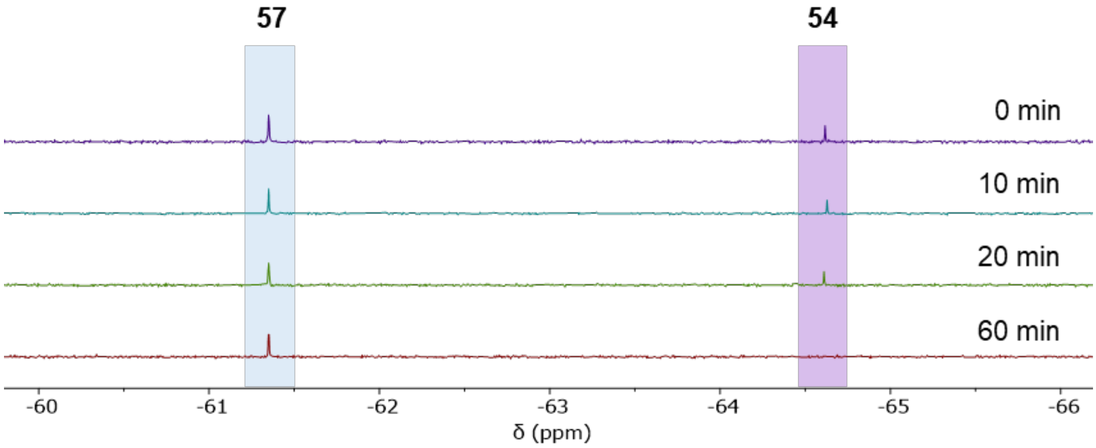

Figure S24. Quantitative ^{19}F NMR spectra of the conversion of **54** (100 µM) catalyzed by **5** (10 µM) in a mixture of DMSO-d_6:D_2O (1:1, 500 µL) after irradiation with blue light (450 nm) at different time points (top to bottom). Internal standard: 4-(trifluoromethyl)benzoic acid.

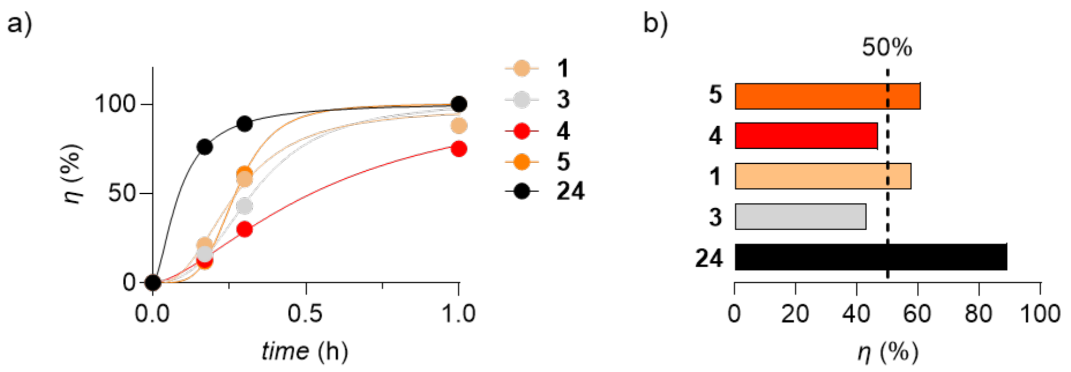

Figure S25. Summary of the conversion of **54** with a catalytic amount of pc (10 mol%, **24**, **1**, **3**, **4**, **5**) under 450 nm constant irradiation: a) conversion curves for different pcs. b) conversion after 20 min of irradiation, simulating cellular conditions.

12.2. Protein Labeling

Labeling efficiency was assessed by a slightly modified procedure from reference S14. Photocatalyst **24** or **pc-CAX** (10 μM) was combined with diazirine **2** (100 μM) and bovine serum albumin (BSA, 10 μM) in DPBS (100 μL). These samples were then either placed in the dark or irradiated with blue light (450 nm) for 15 min at 25 °C. The samples were then subjected to WB analysis as described in section 9, and the results are reported in Figure S26b. Labeling efficiencies of **pc-CAX** ($I_{rel} \approx 1.4$) were comparable to the unmodified **24** ($I_{rel} = 1.0$).

Labeling *vs* different irradiation time. Following the procedure described in reference S14. Photocatalyst **1** (10 μM, DMSO) was added to **2** (100 μM) and BSA (10 μM) in DPBS (1 mL). Aliquots (20 μL) were removed every 5 minutes. The solution was kept in the dark for 15 minutes, followed by irradiation for the same duration. The cycle was repeated up to 30 minutes of irradiation (2 x 15 minutes). The aliquots were subjected to WB analysis as described in section 9 and the results are reported in Figure S26c.

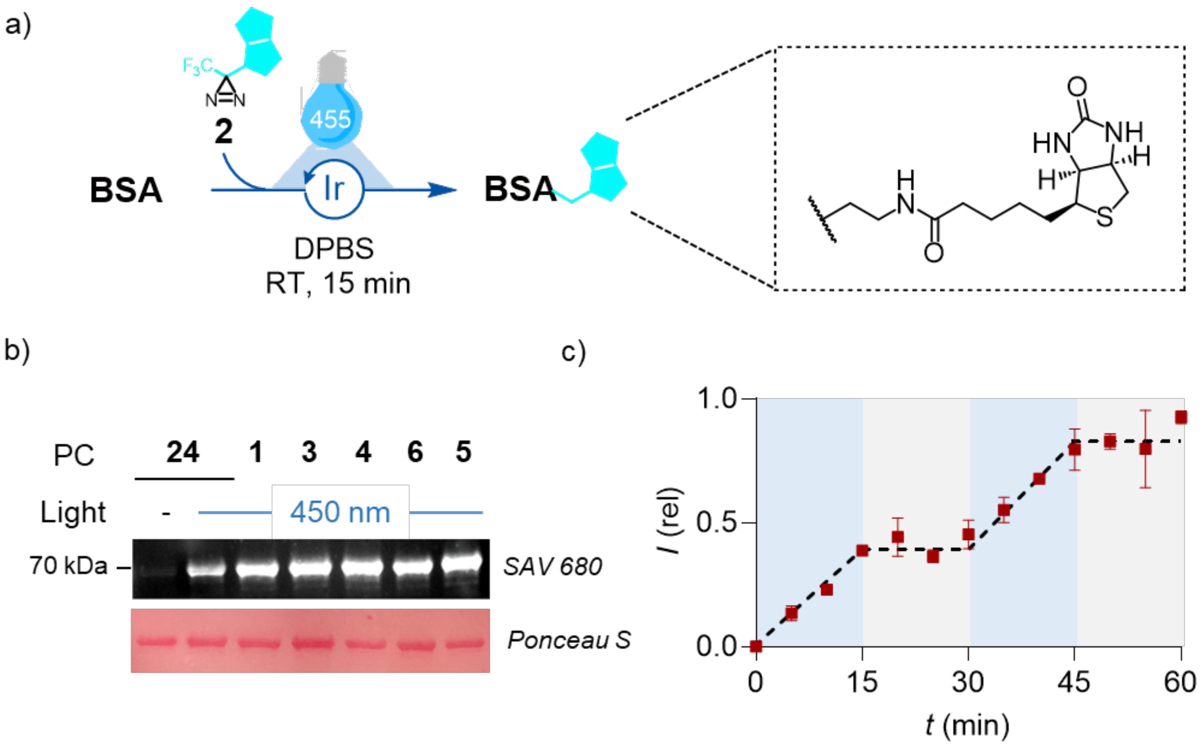

Figure S26. a) Labeling of a generic protein (100 µM, here BSA) with **2** (100 µM) in presence of catalytic amount of pc (10 µM, **1**, **3**, **4-6**, **24**) in DPBS upon irradiation with blue light (450 nm). b) Representative Western Blot of labeling process detected by SAV680. c) Photonically controlled BSA labeling. SAV680: Streptavidin-AlexaFluor 680 conjugate.

12.3. In Cells

The HK cells were prepared in a 96-well plate as described in section 7, then medium was removed, and cells were washed with PBS (3 × 3 mL/well) followed by fresh FDMEM (4 × 100 µL/well) using a plate washer (Biotek EL406®), and kept in 100 µL/well of the latter medium. The solution of **pc-CAX** (10 mM, DMSO) was diluted in FDMEM to give a solution at 3x final concentration (5 µM), of which 50 µL was added to the well resulting in a final volume of 150 µL per well. The cells were incubated under 5% CO_2 humidified atmosphere at 37 °C for 30 min. Afterward, the cells were washed with PBS, and the medium was exchanged with FDMEM, keeping a final volume of 100 µL/well. The cells were treated with a solution of **2** (100 mM, DMSO) pre-

diluted in FDMEM to give a solution at 3x final concentration (100 µM), of which 50 µL was added to the well, resulting in a final volume of 150 µL per well. After incubating for 30 min under 5% CO_2 humidified atmosphere at 37 °C, cells were continuously irradiated in the photoreactor at 450 nm for 15 min at RT. The cells were washed with PBS (3 × 3 mL/well) and fixed with a solution of 3% PFA (70 µL/well) for 15 min at RT. Afterward, the cells were washed with PBS (3 × 3 mL/well) and treated with a solution of SAV680 (5 µg/mL, 80 µL/well) in PBS complemented with 0.05% saponin and 1% BSA for 1 h at RT in the dark. The cells were washed with PBS (9 × 3 mL/well) and kept in the same buffer (100 µL/well) for live cell imaging. The distribution of fluorescent signals was captured on a IXM-C automated microscope with two channels, blue/green for **pc-CAX** (377/50 nm excitation filter; 536/40 nm emission filter) and Cy-5 for SAV680 (620/50 nm; emission filter: 690/50 nm). Technical duplicates were performed for each condition.

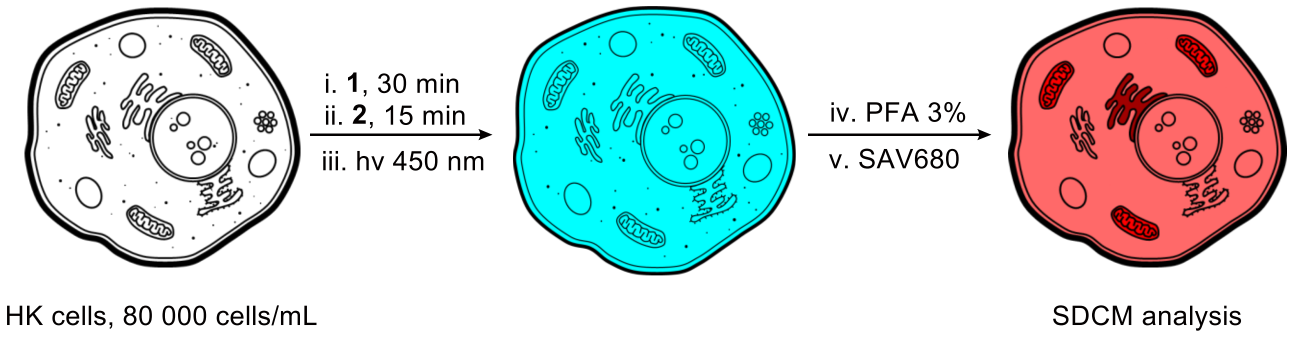

Figure S27. Labeling process in cells: HK cells were incubated with pc-AspA (**1**) for 30 min, rinsed, incubated with **2** for 15 min, and irradiated at 450 nm for 15 min. The cells were then washed with PBS, fixed with 3% PFA, and permeabilized with saponin. Finally, the cells were incubated with SAV680 for 1 hour at RT. SDCM analysis revealed in the red channel all labeled compartments in the cells.

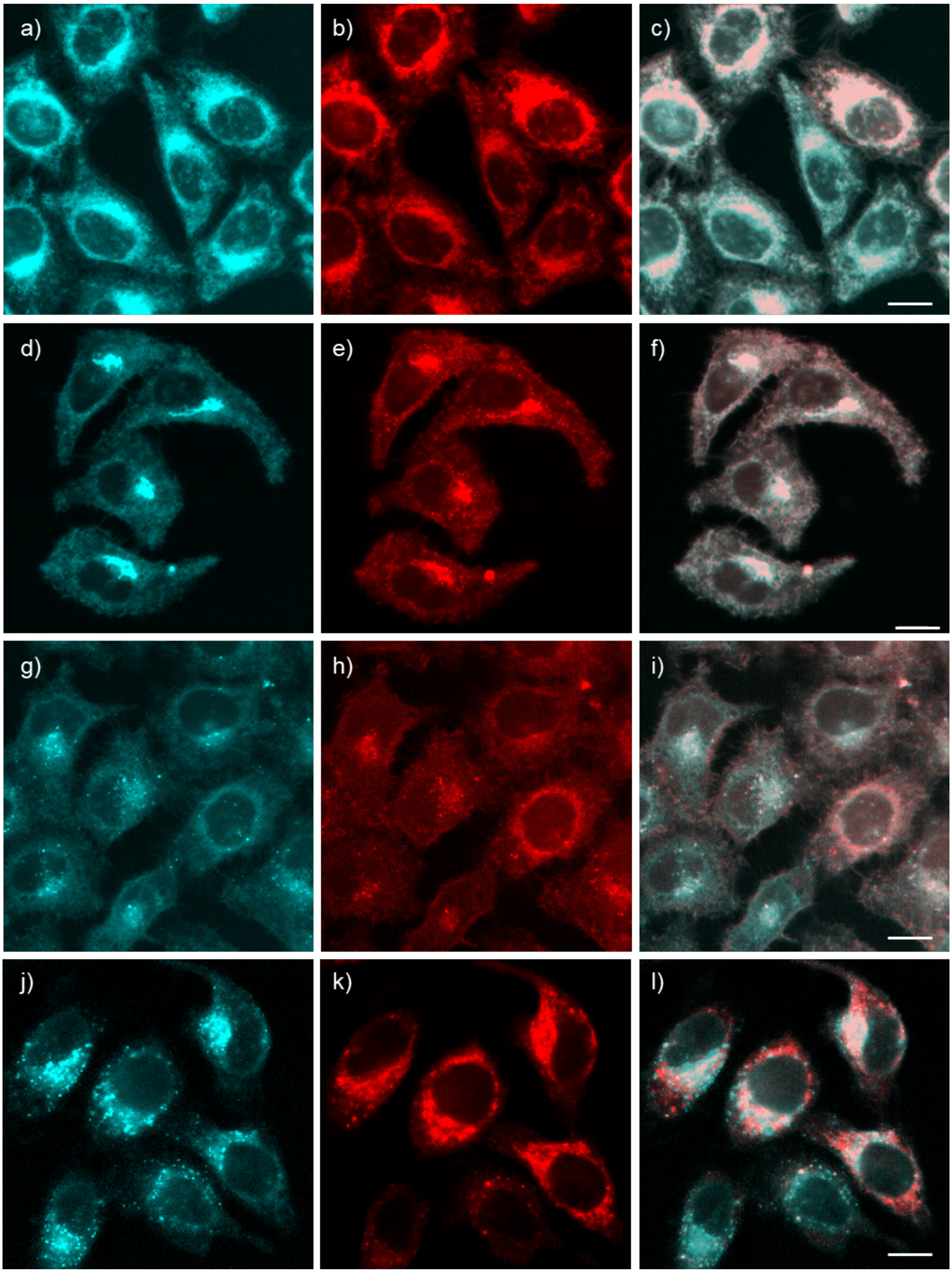

Figure S28. SDCM images (40X WI) showing luminescence of a) **3**, d) **1**, g) **4**, and j) **5** (all 5 µM, cyan), b, e, h, k) SAV680 (red) for the corresponding labeled sites, and c, f, h, l) the merge of both in HK cells (scale bar 10 µm).

13. Proteomics

13.1. General Procedure

General sample preparation. The proteome samples were prepared using 2 different methods. **Co-incubation**: Cells were seeded (10^6 cells/mL, 2 mL) in a 6-well plate (triplicate per row) in complete FDMEM and left incubating overnight at 37 °C with 5% CO_2. The next day, the cells were washed with DPBS and covered with 500 µL/well of FDMEM containing 250 µM diazirine **2**. The cells were incubated in the dark for 15 min at 37 °C with 5% CO_2. The solution of **pc-CAX** (5 µL of 1 mM stock in DMSO) in FDMEM (500 µL) was added to the cells. The cells were incubated for 20 minutes at 37 °C with 5% CO_2, protected from light. Afterward, they were exposed to continuous irradiation in a photoreactor (450 nm) for an additional 10 min at RT, with the fan turned on to prevent excessive heating. The cells were washed with DPBS (3 x 1 mL) and subjected to lysis as described in section 8. The protein lysate was adjusted to a final concentration of 1000 µg/mL. Meanwhile, magnetic streptavidin beads (Dynabeads M280, 200 µL of beads for 250 µg of total protein) were transferred to 1.5 mL Lo-Bind tubes and washed once with RIPA 1X (0.5 mL, for 5 min at 800 rpm in a plateshaker). The beads were pelleted using a magnetic rack, then resuspended in 250 µL of the protein samples per tube. The mixture was incubated in a thermoshaker at 4 °C overnight (at least 12 h) with shaking at 800 rpm. The beads were pelleted on a magnetic rack, and the supernatant was discarded. The beads were subsequently washed for 5 min at 4 °C each time with 1% SDS in DPBS (3 x 1 mL/tube), 1 M NaCl in DPBS (3 x 1 mL/tube), 10% EtOH in DPBS (3 x 1 mL/tube), DPBS (6 x 1 mL/tube) and finally 100 mM NH_4HCO_3 solution (1 mL/tube). The beads were incubated with 6 M urea and 10 mM DTT in 25 mM NH_4HCO_3 solution for 30 min at 55 °C. Following this step, iodoacetamide was added to a final concentration of 27 mM in 25 mM NH_4HCO_3, and the mixture was incubated at RT in the dark for 30 min. After removing the supernatant, the beads were washed with DPBS (3 x 1 mL/tube) and with 25 mM NH_4HCO_3 (3 x 1 mL/tube). The beads

were then covered with Lys-C/trypsin (Promega, USA) in 25 mM NH_4HCO_3 and digested for 16 h at 37 °C. Afterward, samples were centrifuged at 18,000 x g for 3 min and acidified with 0.032% (v/v) formic acid (FA). The supernatant was transferred into a fresh tube and combined with three times of wash using 300 µL of 25 mM NH_4HCO_3. The protein digest was dried using a centrifugal vacuum concentrator and dissolved in 0.1% (v/v) TFA in 3% acetonitrile (v/v). Samples were sonicated for 1 min, centrifuged at 15,000 x g for 15 min, and desalted using MonoSpin C18 columns (GL Sciences Inc., Japan). Peptides were eluted from C18 columns using 0.1% TFA in 50% acetonitrile and dried in a vacuum concentrator. Tryptic peptides were dissolved in 0.1% (v/v) formic acid in 2% (v/v) acetonitrile for MS analysis. **Pre-incubation**: Cells were seeded (10^6 cells/mL, 2 mL) in a 6-well plate (triplicate per row) in complete FDMEM and left incubating overnight at 37 °C with 5% CO_2. The next day, the cells were washed with DPBS and resuspended in 500 µL/well of FDMEM containing 5 µM photocatalyst (**1**, **3**–**6**). The cells were incubated in the dark for 20 min at 37 °C with 5% CO_2 and then washed with DPBS (3 x 1 mL/well). Diazirine **2** in FDMEM was added to the cells to a final concentration of 250 µM and final volume of 1.0 mL. The cells were incubated for 15 minutes at 37 °C with 5% CO_2, protected from light. Afterward, they were exposed to continuous irradiation in a photoreactor (450 nm) for an additional 10 min at RT, with the fan turned on. The following steps are as described in the co-incubation method.

Protein profiling after different incubation time. The general procedure for sample preparation using the co-incubation method was followed, except that the respective **pc-CAX** was incubated with HK cells for 0/10/20 min in the dark followed by 10 min of continuous irradiation in a photoreactor (450 nm) at RT.

Protein profiling with 4 in presence of TMU inhibitors. Based on the general procedure for sample preparation using the co-incubation method, cells were seeded (10^6 cells/mL, 2 mL) in a 6-well plate (triplicate per row) in complete FDMEM and left incubating overnight at 37 °C under 5% CO_2. The next day, the cells were washed with DPBS and resuspended in 500 µL/well of FDMEM

containing either **6** (20 μM), **7** (10 μM) or **8** (50 μM). The cells were incubated in the dark for 1 h at 37 °C with 5% CO_2. The photocatalyst **4** (5 μL of 1 mM in DMSO) in FDMEM (500 μL) was added to the cells to a final concentration of 5 μM and final volume of 1 mL. The cells were incubated for 20 min at 37 °C with 5% CO_2, protected from light. Diazirine **2** was added to the cells to give a final concentration of 250 μM and a final volume of 1.5 mL. The cells were incubated in the dark for 15 min, followed by exposition to continuous irradiation in a photoreactor (450 nm) for an additional 10 min at RT, with the fan turned on. The cells were washed with DPBS (3 x 1 mL) and subjected to lysis as described in section 8. The rest of the procedure was as described for the co-incubation method.

13.2. Data Acquisition

Samples were measured on an Easy Nano LC - Orbitrap Fusion System equipped with a nanospray flex™ ion source (Thermo Fisher Scientific, USA). Peptides were separated on a 1.9-µm particle, 75-µm inner diameter, 15 to 20-cm filling length homemade C18 column. A flow rate of 300 nL/min was used with a 2 h gradient (2–25% solvent B in 100 min, 25–45% solvent B in 7 min, 45–75% solvent B in 7 min). The gradient was followed with two rounds of washing steps, in each step, the gradient switched to 98% solvent B and kept for another 2 min, and switched to 2% solvent B in 1 min and kept for another 2 min. In the second round of washing, an extra 14 min of 2% solvent B was kept for system equilibration. Solvent A was 0.1% (v/v) formic acid in LC/MS grade water and solvent B was 0.1% (v/v) formic acid in 100% (v/v) acetonitrile. The ion source settings from Tune were used for the mass spectrometer ion source properties.

For data-dependent acquisition (DDA), full MS spectra were acquired from 375 to 1500 m/z at a resolution of 120,000. MS2 spectra were acquired in the ion trap with rapid scan rate. The default charge state for the MS2 was set to 2. Charge states 2-7 were included for MS2. HCD fragmentation

was set to fixed collision energy of 35%, and dynamic exclusion was set to 30 s. For both full MS and MS2, the AGC target was set to standard with a maximum injection time (IT) set to auto.

13.3. Data Analysis

Raw files from DDA measurements were searched against the UniProt human database using the Proteome Discoverer software (Thermo Fisher Scientific, USA). Digestion enzyme specificity was set to Trypsin/P. Carbamidomethylation of cysteine was set as fixed modification, oxidation of methionine, as well as acetylation, loss of methionine (met-loss), and acetylation+met loss at protein *N*-terminus were set as variable modifications. Up to two missed cleavages were allowed. Normalization was performed using total peptide amount. Protein abundances were estimated using summed abundances. Protein ratios were calculated using the pairwise ratio-based strategy and the hypothesis test was performed using t-test. A decoy database was included to estimate the false discovery rate (FDR). Search results were filtered with FDR 0.01 at both peptide and protein levels.

Protein fold changes and the significances were calculated from obtained protein abundances by the method described in reference S15. Heavily imputed data points, mainly due to the undetectability of the corresponding protein with the control probes, are marked with white dots in volcano plots. Proteins identified as significantly enriched or suppressed (*i.e.*, $|\log_2(\text{fold change})| \geq 1$ and $-\log_{10}(P) \geq 1.3$) were analyzed using DAVID functional annotation tools (https://davidbioinformatics.nih.gov/tools.jsp) and UniProt ID mapping (https://www.uniprot.org/id-mapping) to determine their subcellular localization. The cell surface proteins compiled by Backus and coworkers were also consulted.[S16]

STRING analyses (ver. 12.0, full STRING network) [S17] were conducted to identify protein networks surrounding the top-hit proteins (Figure S35). Several networked proteins, including the experimentally confirmed protein partners (TMEM30A: CDC50A, BSG), were found to be enriched in the μ-Map analyses, consistent with its capability of proximity labeling.

13.4. Results of Proteomics Analyses

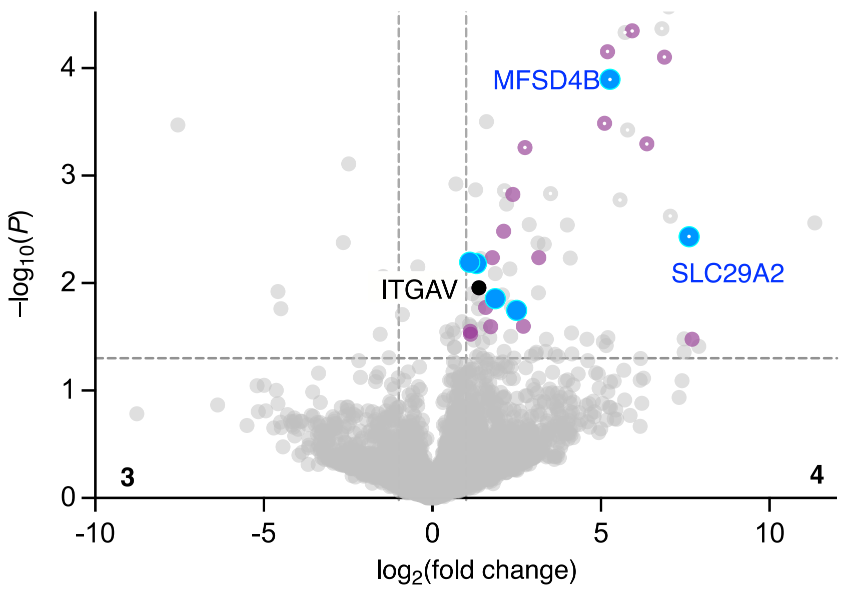

Figure S29. Volcano plot for pc-ETP **4** (right) compared to control **3** (left), obtained after pre-incubation of HK cells with **4** or **3** (20 min), followed by washing, addition of **2**, and irradiation at 450 nm for 10 min. Proteins at cell membranes (purple), cell surface SLCs and MFSDs (blue), previously identified targets (black), and heavily imputed data points (white dots).

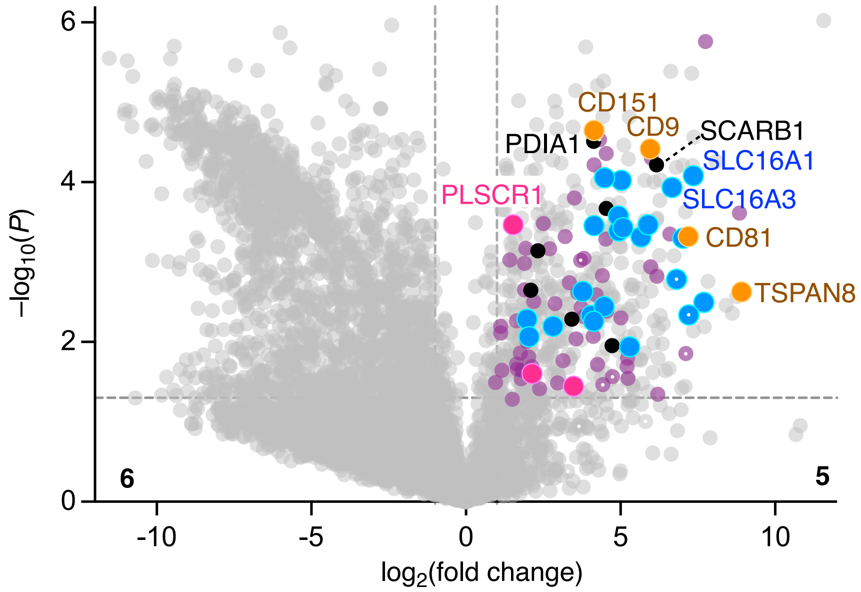

Figure S30. Volcano plot for pc-hAspA **5** (right) compared to control **6** (left), obtained after pre-incubation of HK cells with **5** or **6** (20 min), followed by washing, addition of **2**, and irradiation at 450 nm for 10 min. Proteins at cell membranes (purple), cell surface SLCs and MFSDs (blue), flip/flop/scramblases (magenta), tetraspanins (orange), previously identified targets (black), and heavily imputed data points (white dots).

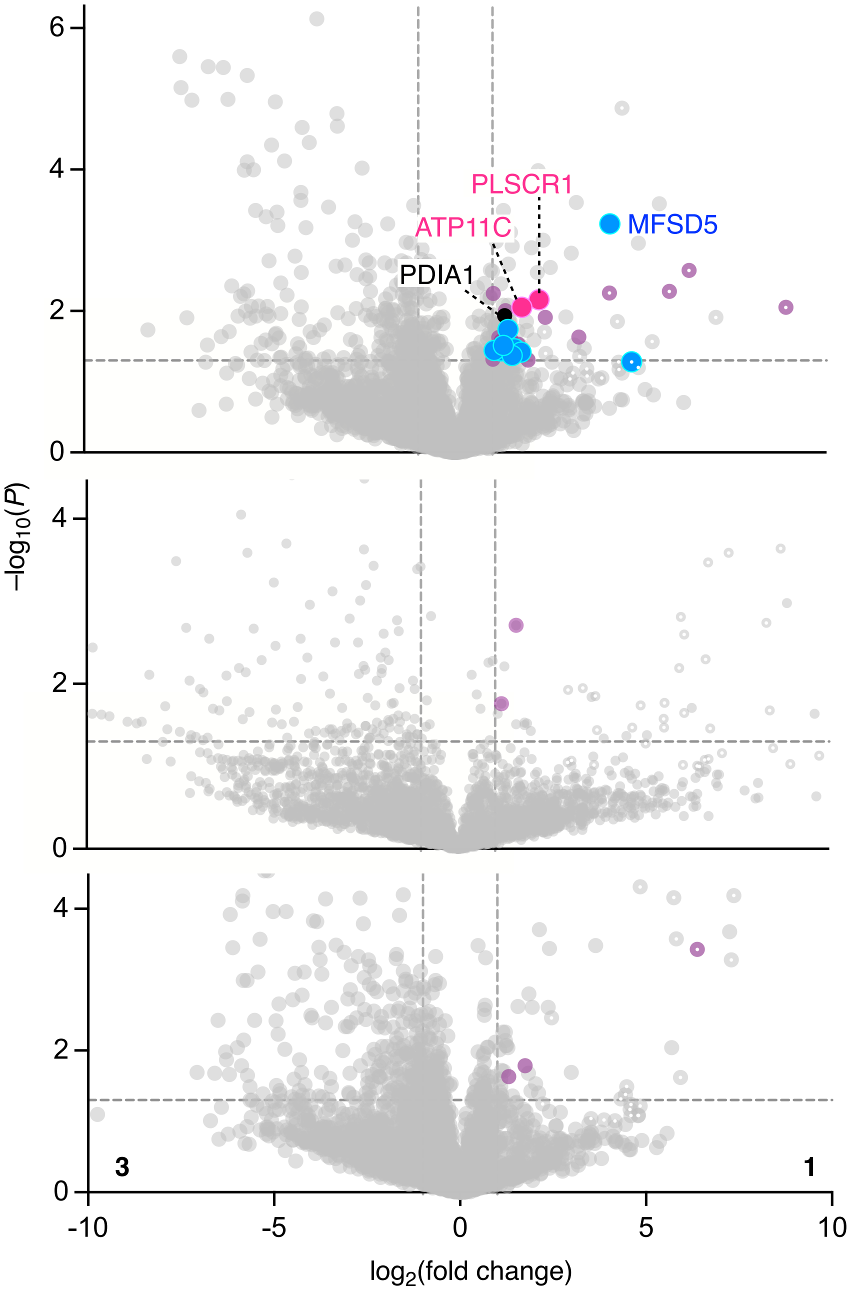

Figure S31. Volcano plots for pc-AspA **1** (right) compared to control **3** (left), obtained after incubation of HK cells with **2** for 15 min, and with photocatalyst **1** or **3** (co-incubation) for 0 (bottom), 10 (middle), or 20 min (top), followed by irradiation at 450 nm for 10 min. Proteins at cell membranes (purple), cell surface SLCs and MFSDs (blue), flip/flop/scramblases (magenta), previously identified targets (black), and heavily imputed data points (white dots). For significant enrichment of proteins, 20 min of incubation with photocatalysts was necessary.

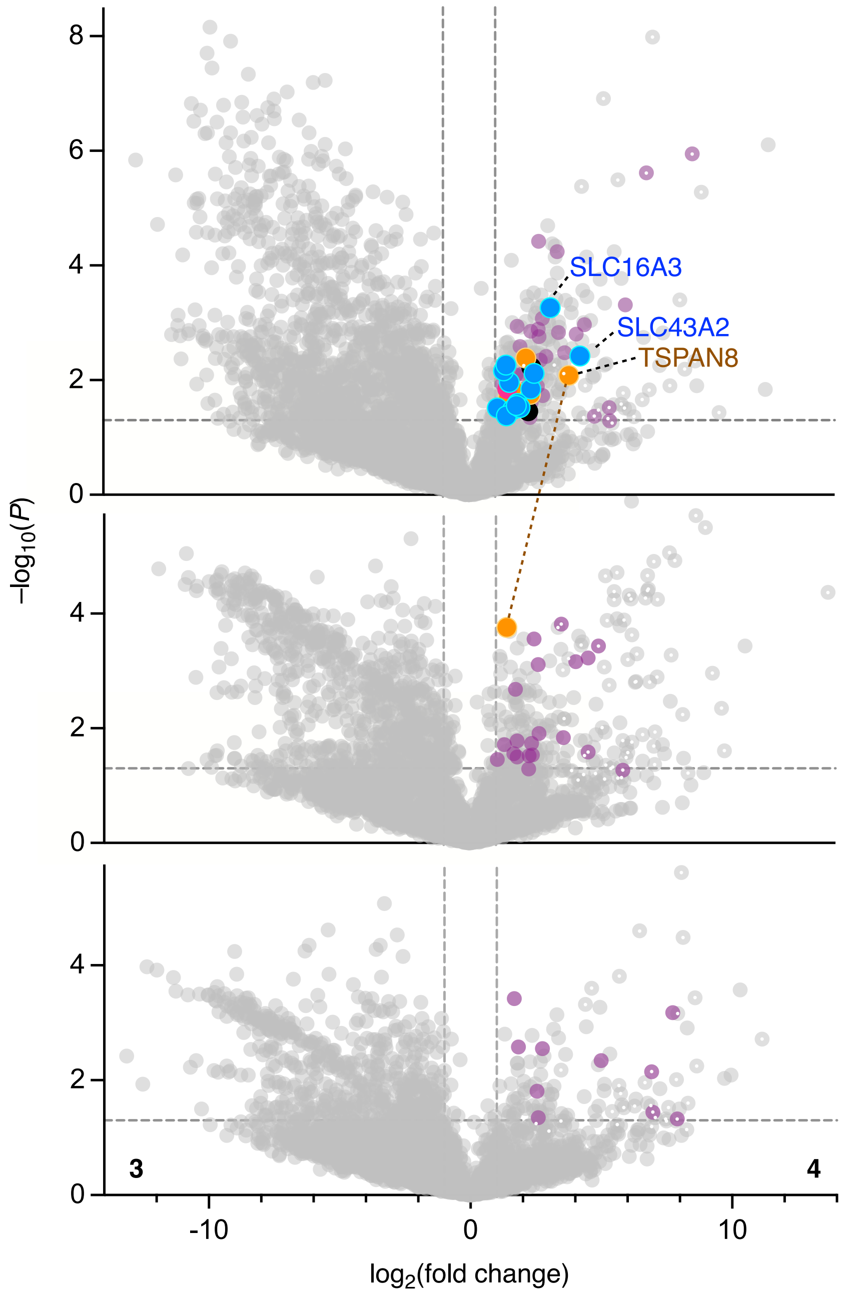

Figure S32. Volcano plots for pc-ETP **4** (right) compared to control **3** (left), obtained after incubation of HK cells with **2** for 15 min, and with additional photocatalyst **4** or **3** (co-incubation) for 0 (bottom), 10 (middle), or 20 min (top), followed by irradiation at 450 nm for 10 min. Proteins at cell membranes (purple), cell surface SLCs and MFSDs (blue), flip/flop/scramblases (magenta), tetraspanins (orange), previously identified targets (black), and heavily imputed data points (white dots). For significant enrichment of proteins, 20 min of incubation with photocatalysts was necessary.

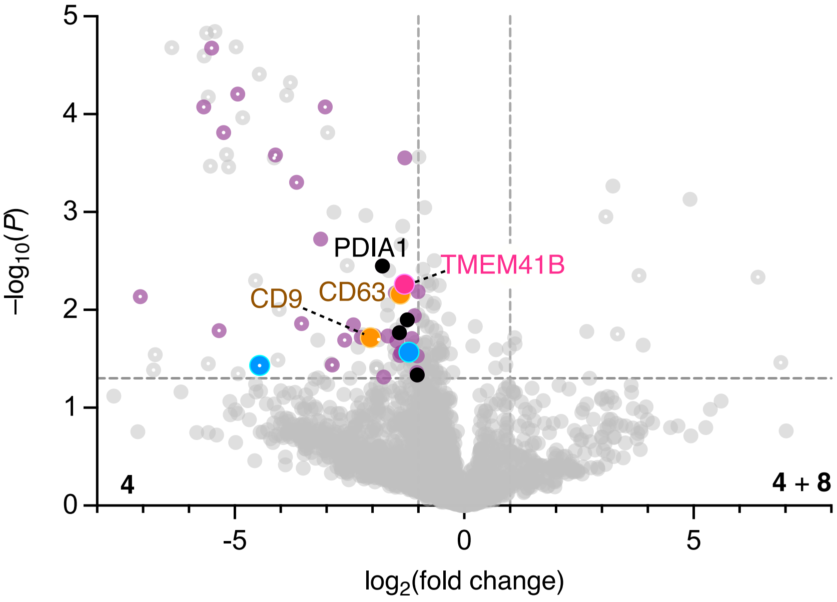

Figure S33. Volcano plots for pc-ETP **4** with (right) *vs*. without BiC **8** (left), obtained after incubation of HK cells with/without **8** (1 h), with **4** (20 min), and with diazirine **2** (15 min), followed by irradiation at 450 nm for 10 min. Proteins at cell membranes (purple), cell surface SLCs and MFSDs (blue), flip/flop/scramblases (magenta), tetraspanins (orange), previously identified targets (black), and heavily imputed data points (white dots).

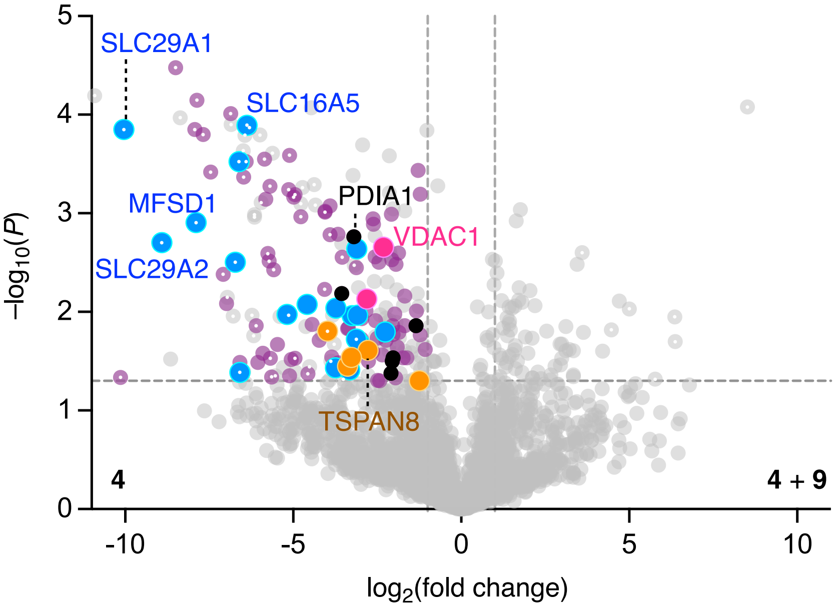

Figure S34. Volcano plots for pc-ETP **4** with (right) *vs*. without EBS **9** (left), obtained after incubation of HK cells with/without **9** (1 h), with **4** (20 min), and with diazirine **2** (15 min), followed by irradiation at 450 nm for 10 min. Proteins at cell membranes (purple), cell surface SLCs and MFSDs (blue), flip/flop/scramblases (magenta), tetraspanins (orange), previously identified targets (black), and heavily imputed data points (white dots).

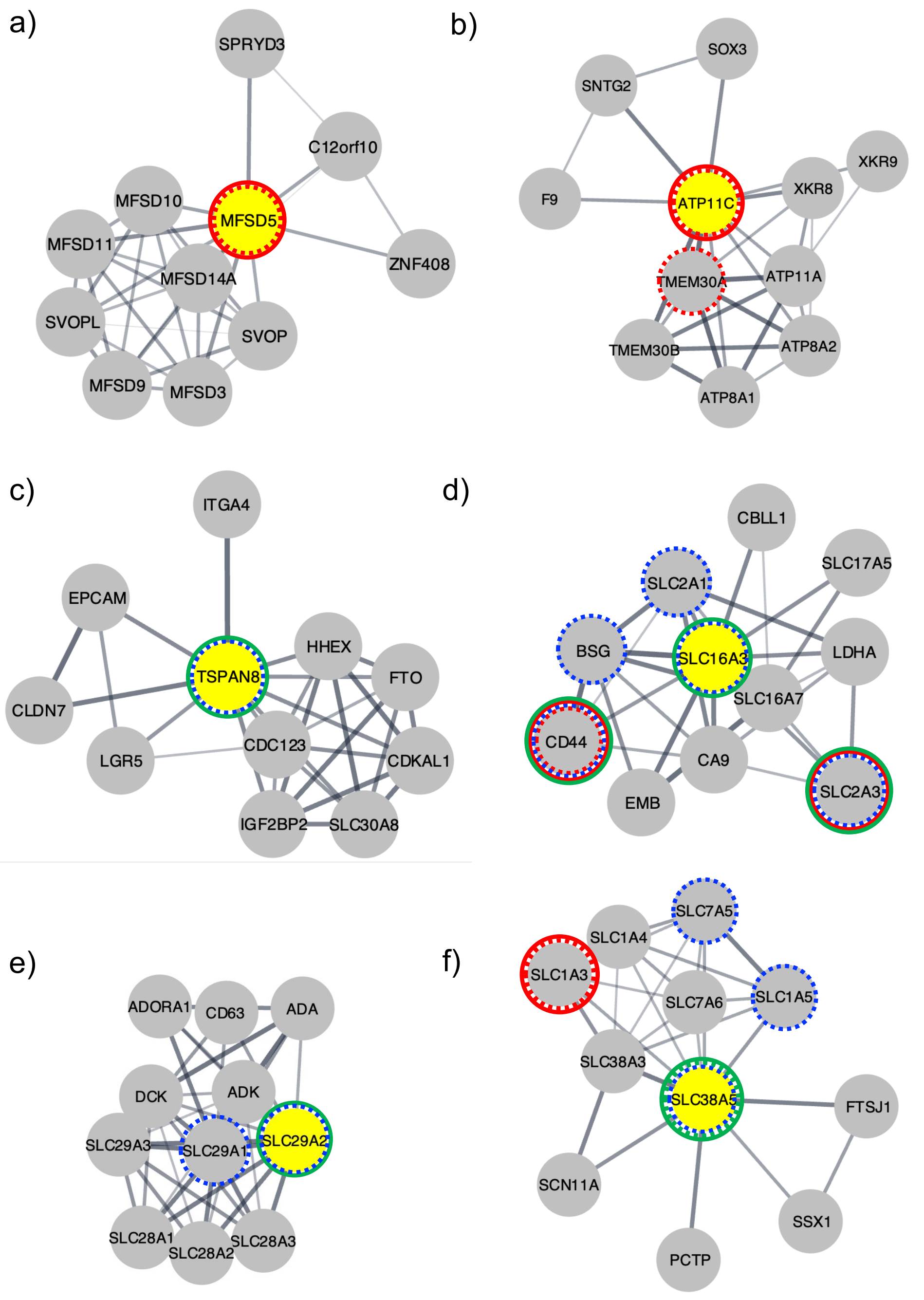

Figure S35. Full STRING networks of a) MFSD5, b) ATP11C, c) TSPAN8, d) SLC16A3, e) SLC29A2, and f) SLC38A5, visualized using Cytoscape (ver. 3.10.3, clustered). Thicker and darker lines between protein nodes indicate higher "scores", i.e., confidence. Encircled are proteins being significantly enriched with pc-AspA **1** (red), pc-ETP **4** (green), and pc-hAspA **5** (blue) under pre- (dashed) or co-incubation conditions (solid lines).

14. Protein Knockdown

14.1. Level of Expression

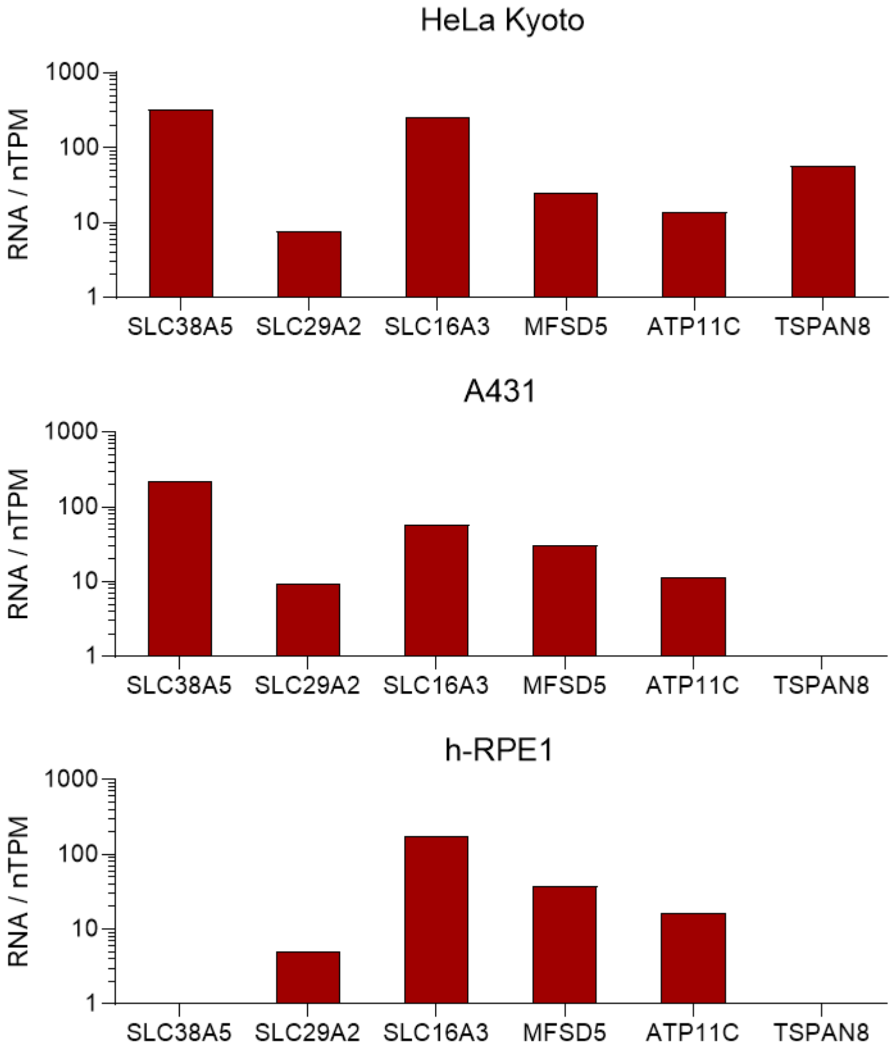

Figure S36. The level of expression of the hit proteins revealed by micromapping in selected cell lines. These data were obtained from https://www.proteinatlas.org.

14.2. Knockdown Procedure

To knockdown selected proteins, reverse transfection was performed with a final siRNA concentration of 5 nM, following the transfection procedure provided by siTOOLs Biotech. Briefly, siRNA was diluted in reduced serum Opti-MEM medium and pre-mixed with Lipofectamine RNAiMAX in Opti-MEM for 15 min at RT. The siRNA mixture was then added to a 6-well sterile plate (500 µL per well). HK, A431 or RPE-1 cells were seeded at 2×10^5 cells per well and incubated at 37 °C with 5% CO_2 for 48 h. After incubation, cells were detached using 500 µL of TrypLE Express at 37 °C for 5 min, followed by the addition of 1 mL of complete FDMEM (supplemented with FCS and PS). The cells were spun down at 1000 rpm for 5 min, resuspended in complete FDMEM, and seeded at 12×10^3 cells per well in a µ-Plate 96-well Black ibiTreat plate. The plate was then incubated overnight at 37 °C with 5% CO_2.

14.3. Quantification of Protein Knockdown by Immunofluorescence

After knocking down the relevant protein (described in section 14.2), the cells were washed with PBS (9 x 3 mL/ well) using the plate washer (Biotek EL406®). Then, they were fixed using a 5% PFA solution for 15 min at RT. The cells were washed again with PBS (9 x 3 mL/well), and treated with rabbit monoclonal primary antibody in PBS containing 1% BSA (70 µL/well, 1/100 of 0.5 mg/mL antibody solution) overnight at 4 °C. After treatment, the cells were washed with PBS (9 x 3 mL/ well) using the plate washer and then treated with a solution of the secondary antibody with a fluorescent dye in PBS (80 µL per well, 1/200 of 1 mg/mL; Alexa Fluor® 647 AffiniPure Donkey Anti-Rabbit IgG (H+L)). The cells were washed with PBS (9 x 3 mL/well), then imaged using an IXM-C automated microscope acquiring 9 images per well using a 40X WI objective lens with 2 channels, blue for Hoechst 33342 (377/50 nm excitation filter; 477/60 nm emission filter) and red for the secondary antibody (620/50 nm excitation filter; 690/50 nm emission filter). The fluorescence intensity of the red channel was extracted and analyzed as described in section 10.

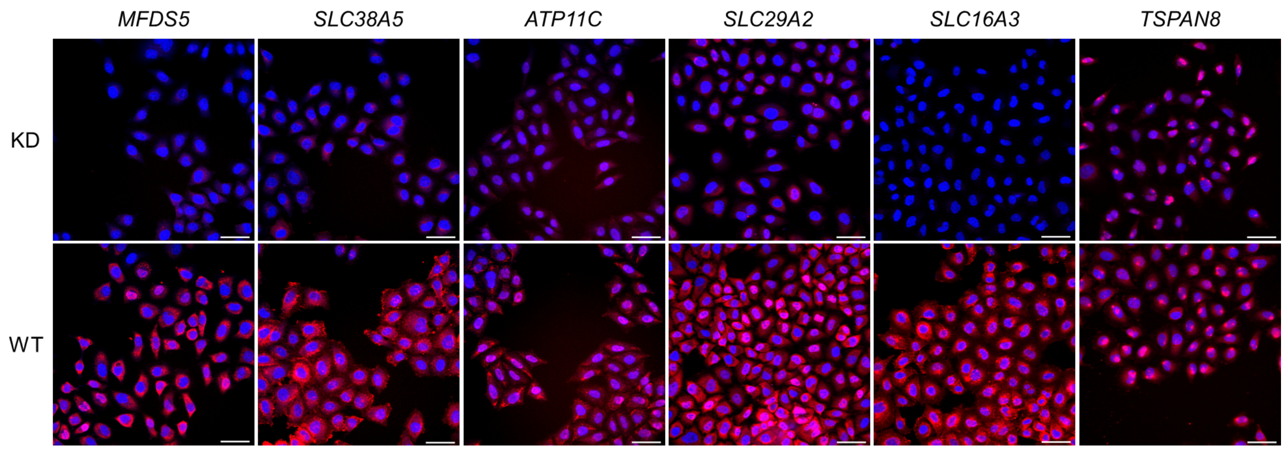

Figure S37. SDCM images (40X WI) for knockdown quantification of MFSD5, SLC38A5, ATP11C, SLC29A2, SLC16A3 and TSPAN8 in HK cells by immunofluorescence; (red: IF with AlexaFluor 647; blue: Hoechst 33342, nuclei; scale bar 50 µm).

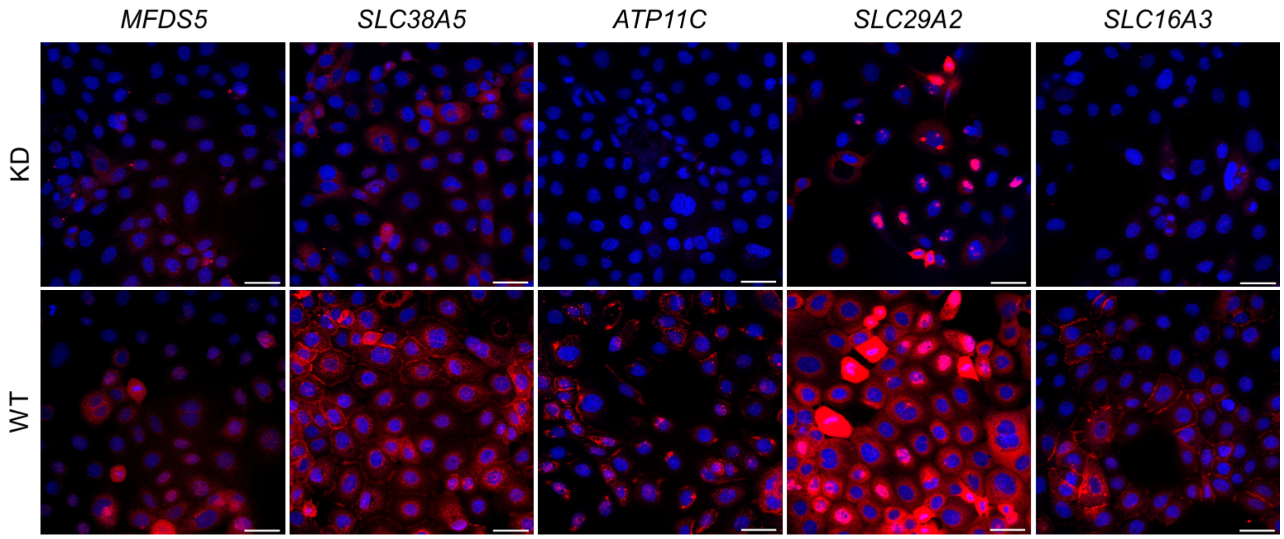

Figure S38. SDCM images (40X WI) for knockdown quantification of MFSD5, SLC38A5, ATP11C, SLC29A2, SLC16A3, SLC7A5 and SLC3A2 in A431 cells by immunofluorescence; (red: IF with AlexaFluor 647; blue: Hoechst 33342, nuclei; scale bar 50 µm).

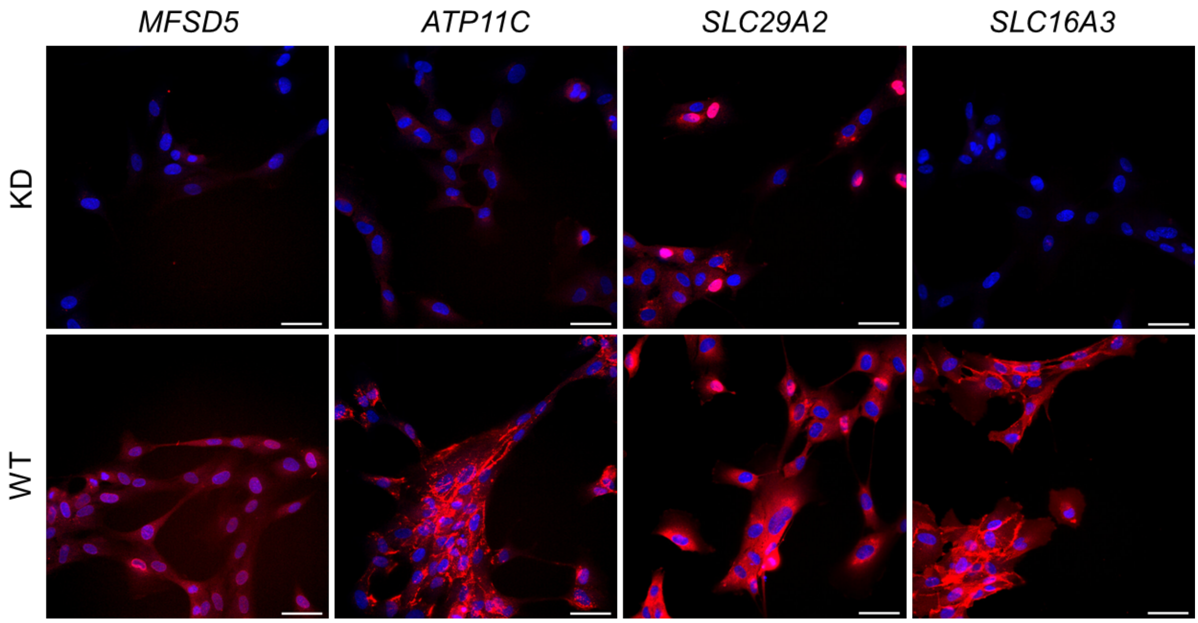

Figure S39. SDCM images (40X WI) for knockdown quantification of MFSD5, ATP11C, SLC29A2, SLC16A3, SLC7A5 and SLC3A2 in RPE-1 cells by immunofluorescence; (red: IF with AlexaFluor 647; blue: Hoechst 33342, nuclei; scale bar 50 μm).

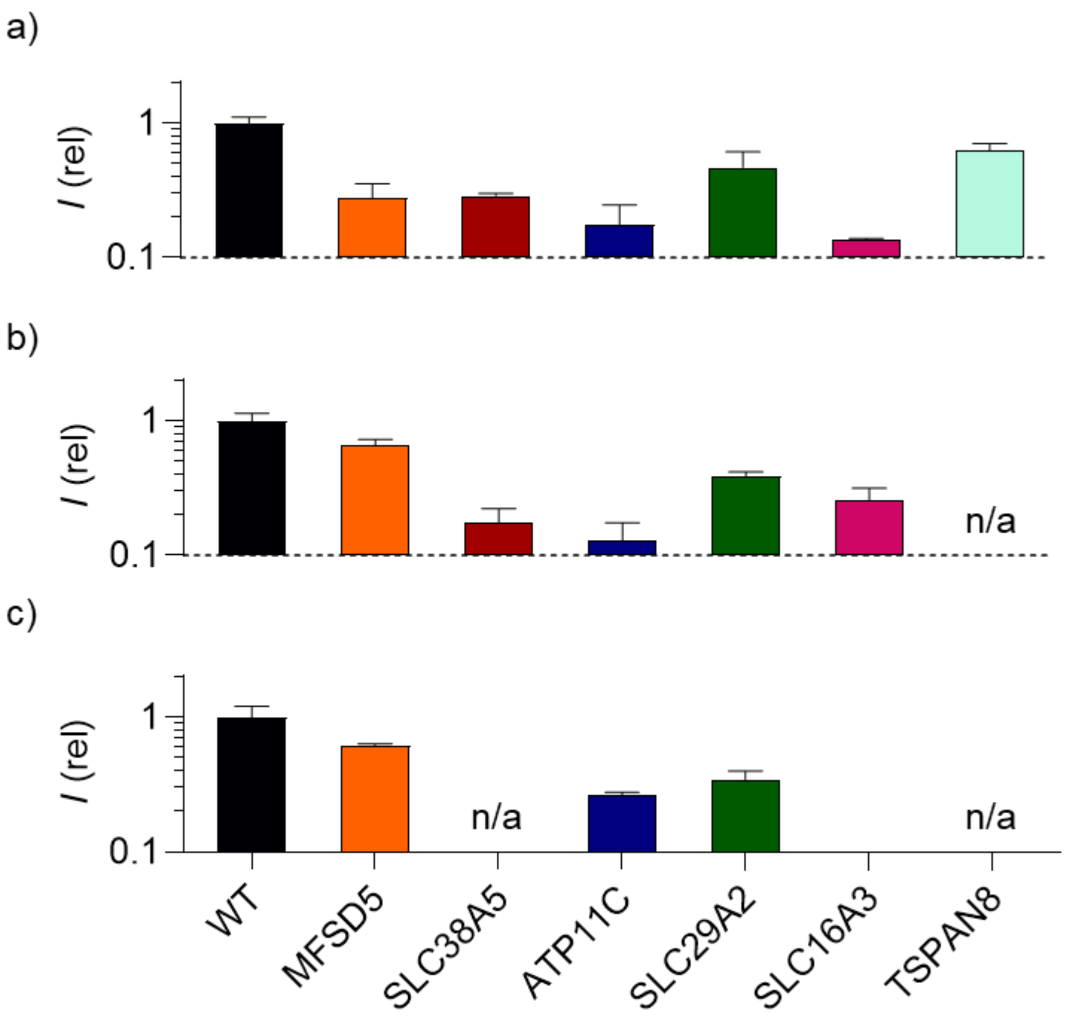

Figure S40. Relative fluorescence intensity *I* (rel) ± SEM indicating the residual expression levels of hit proteins (MFSD5, SLC38A5, ATP11C, SLC29A2, SLC16A3 and TSPAN8) in knocked-down a) HK, b) A431, and c) RPE-1 cells, as determined by IF quantification. n/a: not applicable.

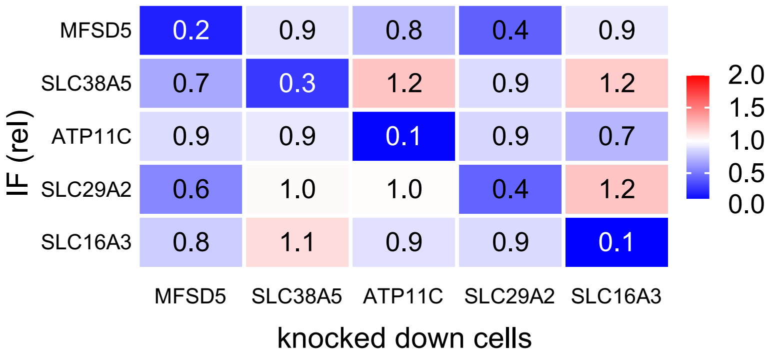

Figure S41. Heatmap of IF quantification results using knockdown cells and corresponding primary antibodies. Cross-reactivity was found between MFSD5 and SLC29A2, probably due to the cross-silencing of protein knockdown or poor specificity of the antibodies.

14.4. Cellular Uptake in Knocked-Down Cells

The cells were prepared in a 96-well plate as described in section 14.2, then medium was removed, and cells were washed with PBS (3 × 3 mL/well) followed by fresh L15 (4 × 100 µL/well) using a plate washer (Biotek EL406®), and kept in 100 µL/well of the latter medium. The KD cells were incubated with Fl-CAX **10–13** (10 µM for **10**, **12**, **13** and 5 µM for **11**) for 30 or 120 min under 5% CO_2 humidified atmosphere at 37 °C. After that, the cells were washed with PBS (3 × 3 mL/well) and L15 (4 × 100 µL/well) using the plate washer. A solution of Hoechst 33342 (100 µg/mL) and PI (10 µg/mL) from the V-bottom plate was added (50 µL/well) to the cells and left incubating for 15 min under 5% CO_2 humidified atmosphere at 37 °C. After one more step of washing with PBS (9 × 3 mL/well) followed by L15, the cells were either imaged live or fixed beforehand. For live imaging, the distribution of fluorescent signals was captured on a IXM-C automated microscope acquiring 6 images per well using a 20x WI objective lens blue for Hoechst 33342 (377/50 nm excitation filter; 477/60 nm emission filter) and green for FITC transporter (475/34 nm excitation filter; 536/40 nm emission filter). Technical replicates were performed for each condition. For fixed cells, the cells were fixed with 3% PFA for 15 min at RT. The cells were washed a final time with PBS (9 x

3 mL/well) and kept in PBS for imaging. The distribution of fluorescent signals was captured on a IXM-C automated microscope acquiring 9 images per well using a 40× WI objective lens with 2 channels: blue for Hoechst 33342 (377/50 nm excitation filter; 477/60 nm emission filter) and green for FITC transporter (475/34 nm excitation filter; 536/40 nm emission filter). Biological replicates were performed for each condition.

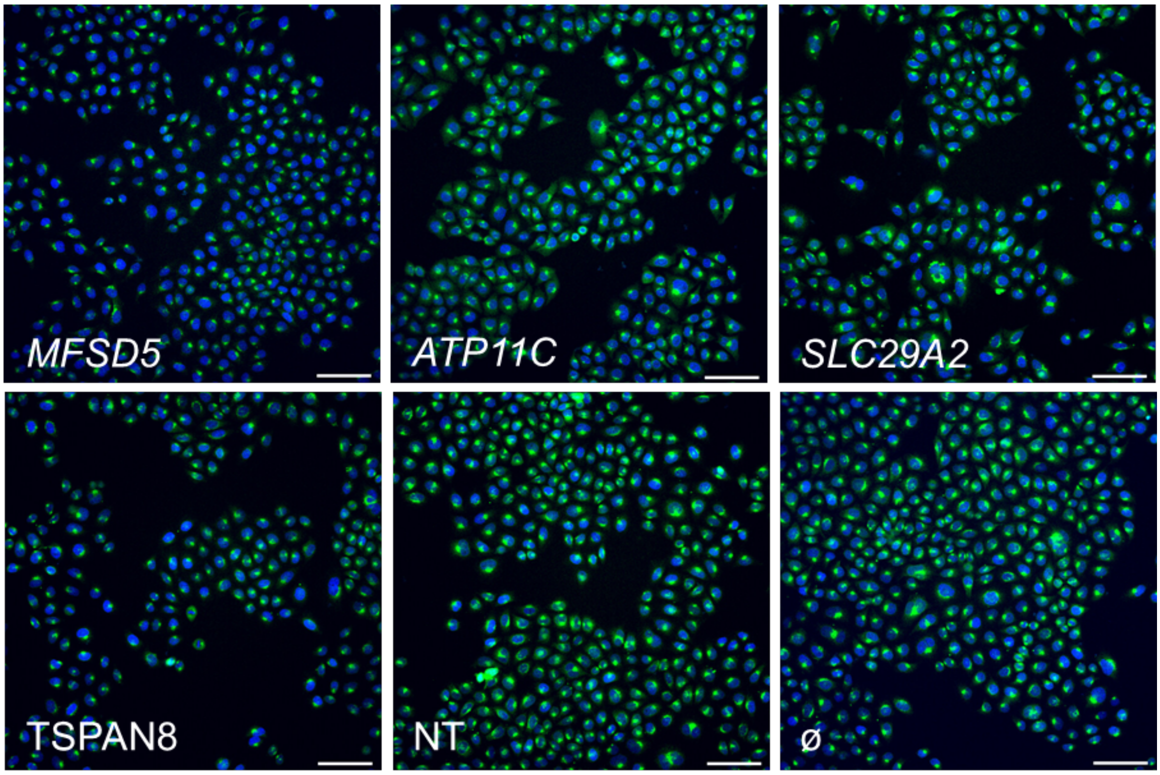

Figure S42. SDCM images (20X WI) showing fluorescence intensity of **10** (10 µM, green) in live KD HK cells after incubation for 30 min in L15 (blue: Hoechst 33342, nuclei; scale bar 100 µm; NT: non-target; ø: non-treated).

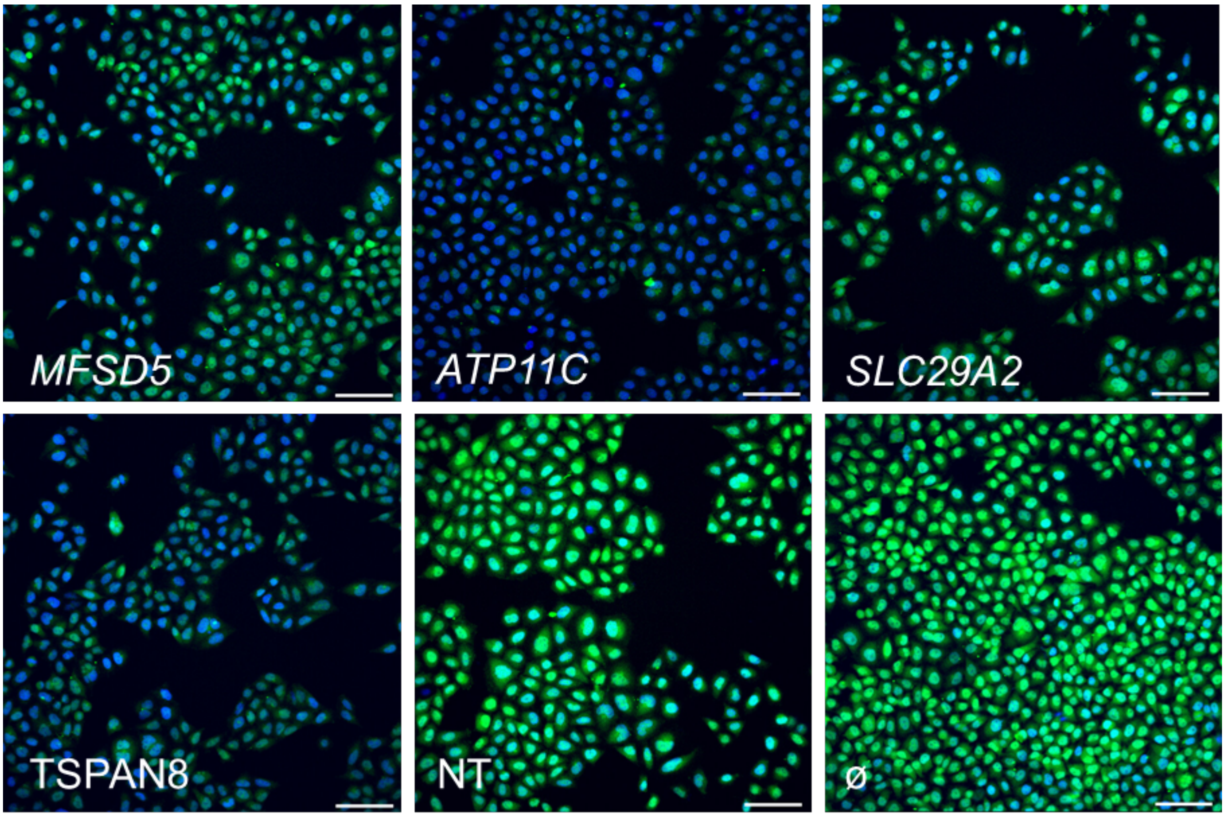

Figure S43. SDCM images (20X WI) showing fluorescence intensity of **11** (10 µM, green) in live KD HK cells after incubation for 30 min in L15 (blue: Hoechst 33342, nuclei; scale bar 100 µm; NT: non-target; ø: non-treated).

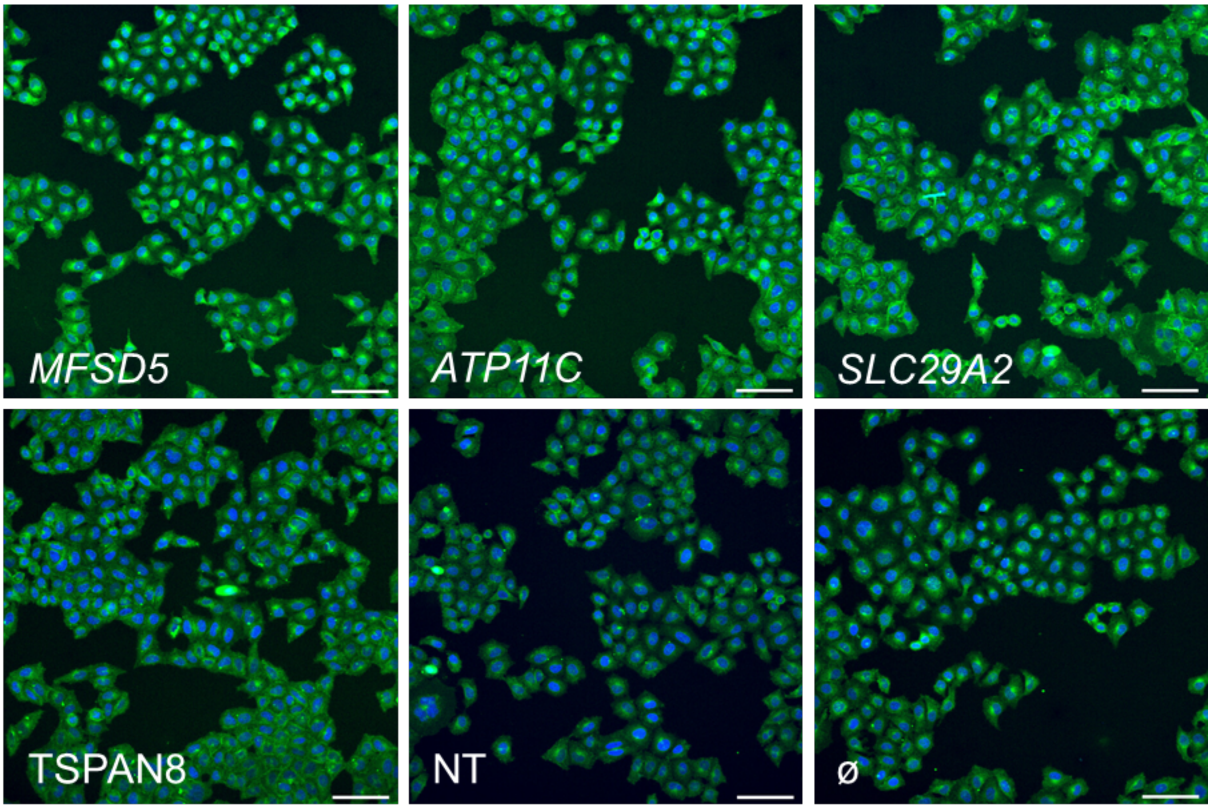

Figure S44. SDCM images (20X WI) showing fluorescence intensity of **12** (10 µM, green) in live KD HK cells after incubation for 30 min in L15 (blue: Hoechst 33342, nuclei; scale bar 100 µm; NT: non-target; ø: non-treated).

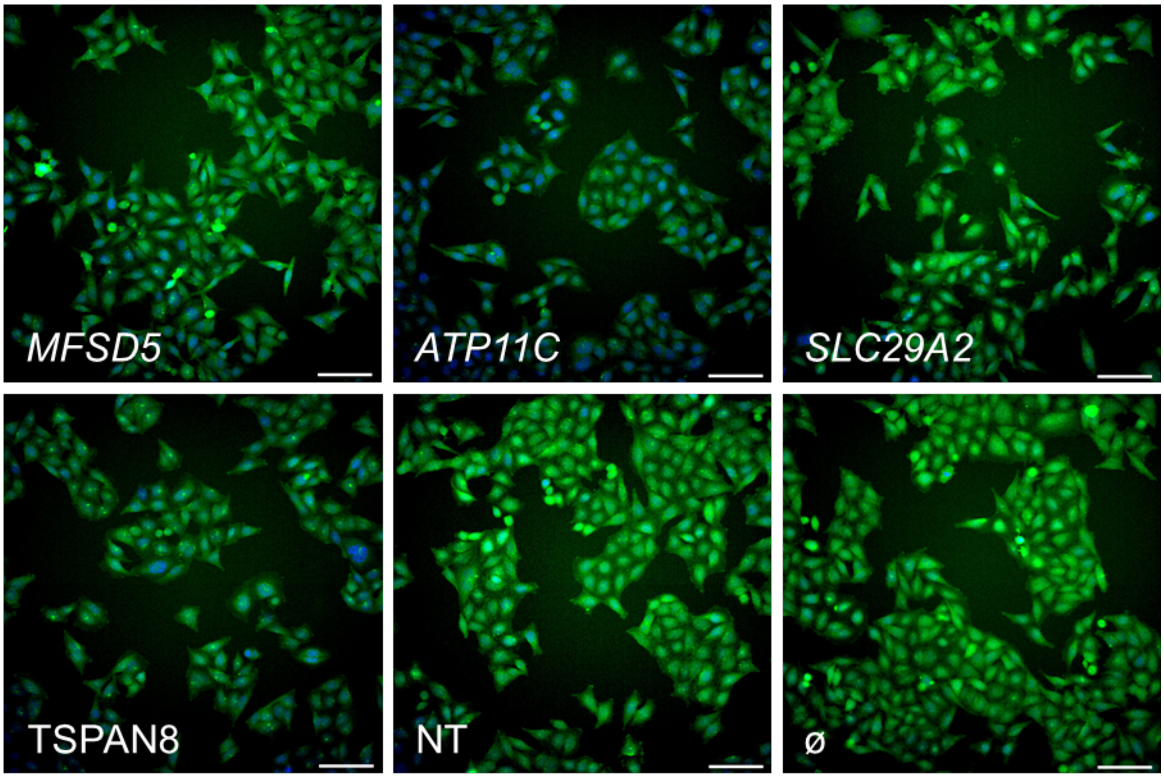

Figure S45. SDCM images (20X WI) showing fluorescence intensity of **13** (5 µM, green) in live KD HK cells after incubation for 6 h in L15 (blue: Hoechst 33342, nuclei; scale bar 100 µm; NT: non-target; ø: non-treated).

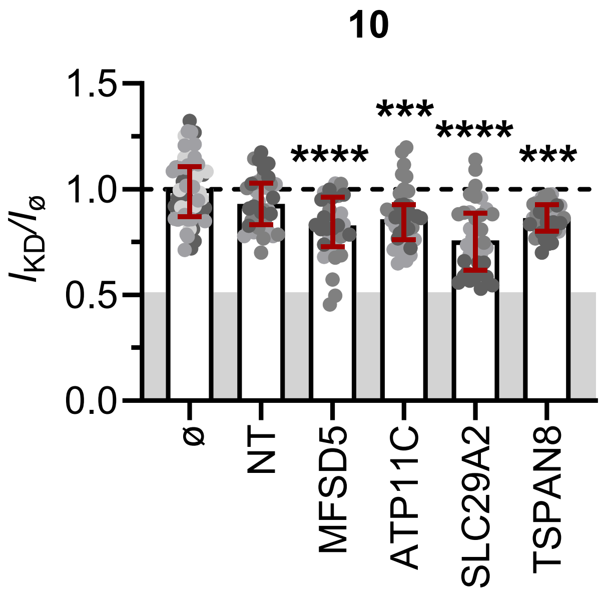

Figure S46. Resulting relative fluorescence intensity $I_{KD}/I_{ø}$ of **10** (10 µM, 30 min) in knocked down live HK cells using the Leibovitz' L15 medium. The x axis refers to knockdown of indicated protein. Each grey circle represents an average cellular fluorescence per cell of an image. Global medians (red horizontal lines) ± interquartile range (IQR) of experimental replicates (n = 3, different shades of grey) with the results of non-parametric one-way ANOVA tests compared to data in non-treated (Ø) cells (P < 0.0001: ****, 0.0002: ***). NT: non-targeted.

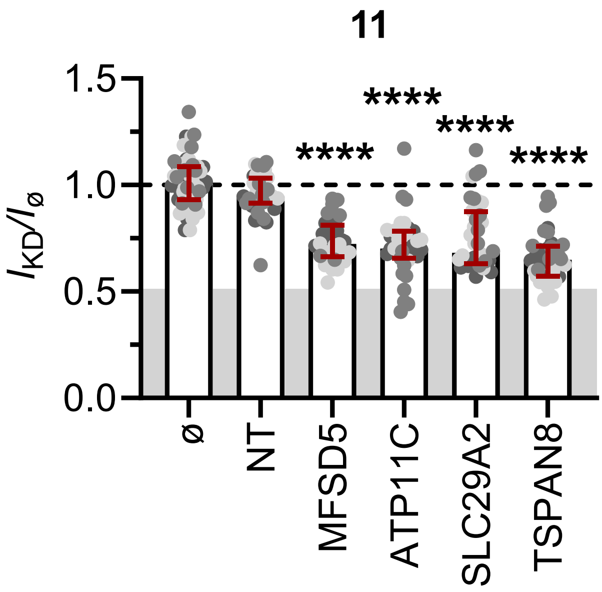

Figure S47. Resulting relative fluorescence intensity $I_{KD}/I_{ø}$ of **11** (10 µM, 30 min) in knocked down live HK cells using the Leibovitz' L15 medium. The x axis refers to knockdown of indicated protein. Each grey circle represents an average cellular fluorescence per cell of an image. Global medians (red horizontal lines) ± interquartile range (IQR) of experimental replicates (n = 3, different shades of grey) with the results of non-parametric one-way ANOVA tests compared to data in non-treated (Ø) cells (P < 0.0001: ****, 0.0002: ***). NT: non-targeted.

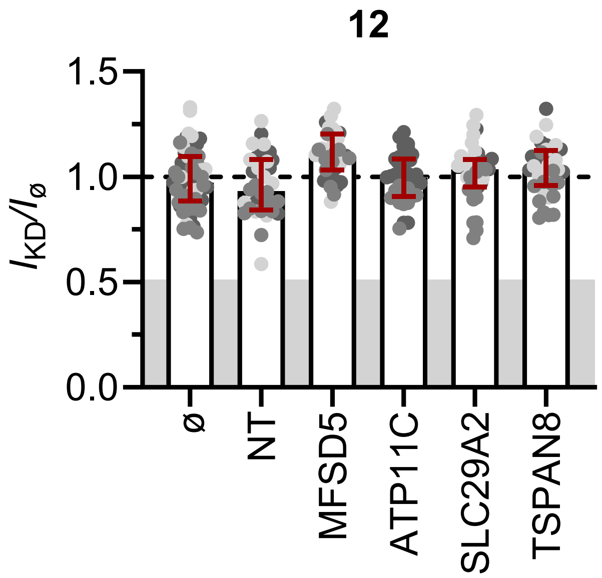

Figure S48. Resulting relative fluorescence intensity $I_{KD}/I_{ø}$ of **12** (10 µM, 30 min) in knocked down live HK cells using the Leibovitz' L15 medium. The x axis refers to knockdown of indicated protein. Each grey circle represents an average cellular fluorescence per cell of an image. Global medians (red horizontal lines) ± interquartile range (IQR) of experimental replicates (n = 3, different shades of grey) with the results of non-parametric one-way ANOVA tests compared to data in non-treated (Ø) cells ($P < 0.0001$: ****, 0.0002: ***). NT: non-targeted.

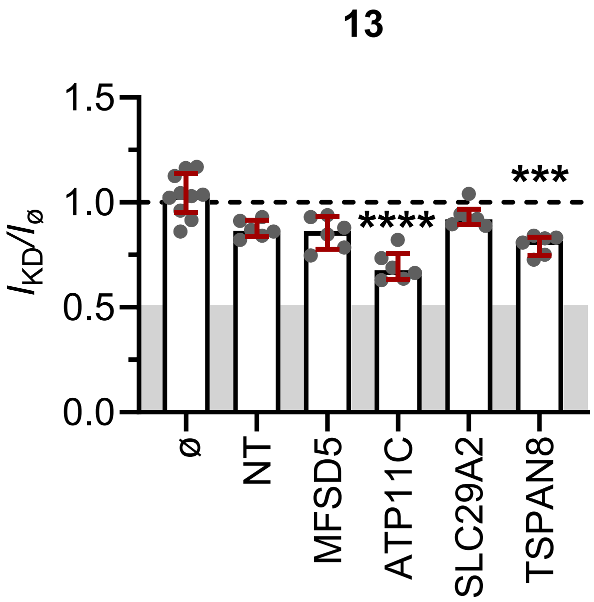

Figure S49. Resulting relative fluorescence intensity $I_{KD}/I_ø$ of **13** (5 µM, 6 h) in knocked down live HK cells using the Leibovitz' L15 medium. The x axis refers to knockdown of indicated protein. Each grey circle represents an average cellular fluorescence per cell of an image with the results of non-parametric one-way ANOVA tests compared to data in non-treated (Ø) cells (P < 0.0001: ****, 0.0002: ***). NT: non-targeted.

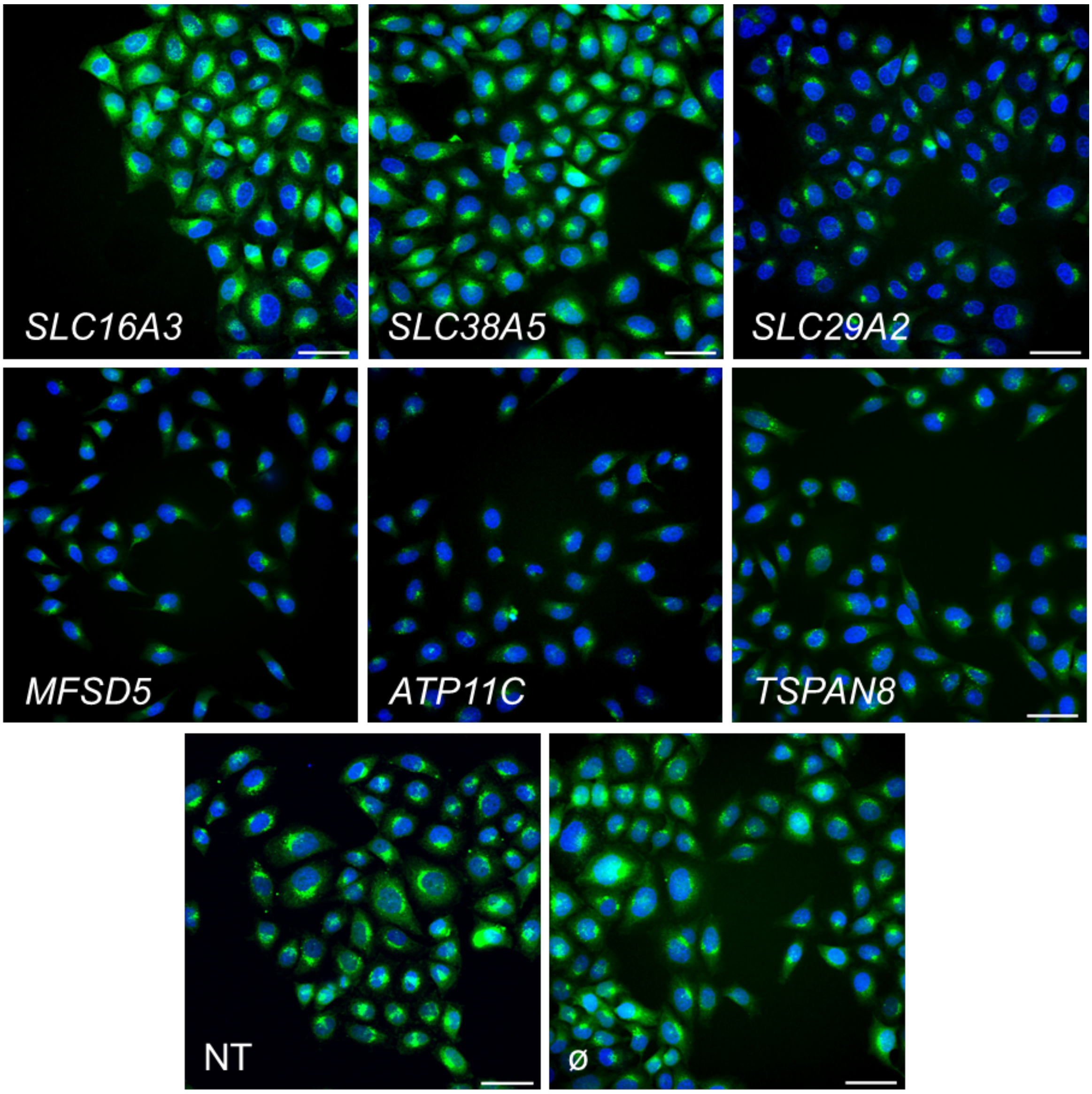

Figure S50. SDCM images (40X WI) showing fluorescence intensity of **10** (10 µM, green) in fixed KD HK cells after incubation for 30 min in L15 (blue: Hoechst 33342, nuclei; scale bar 50 µm; NT: non-target; ø: non-treated).

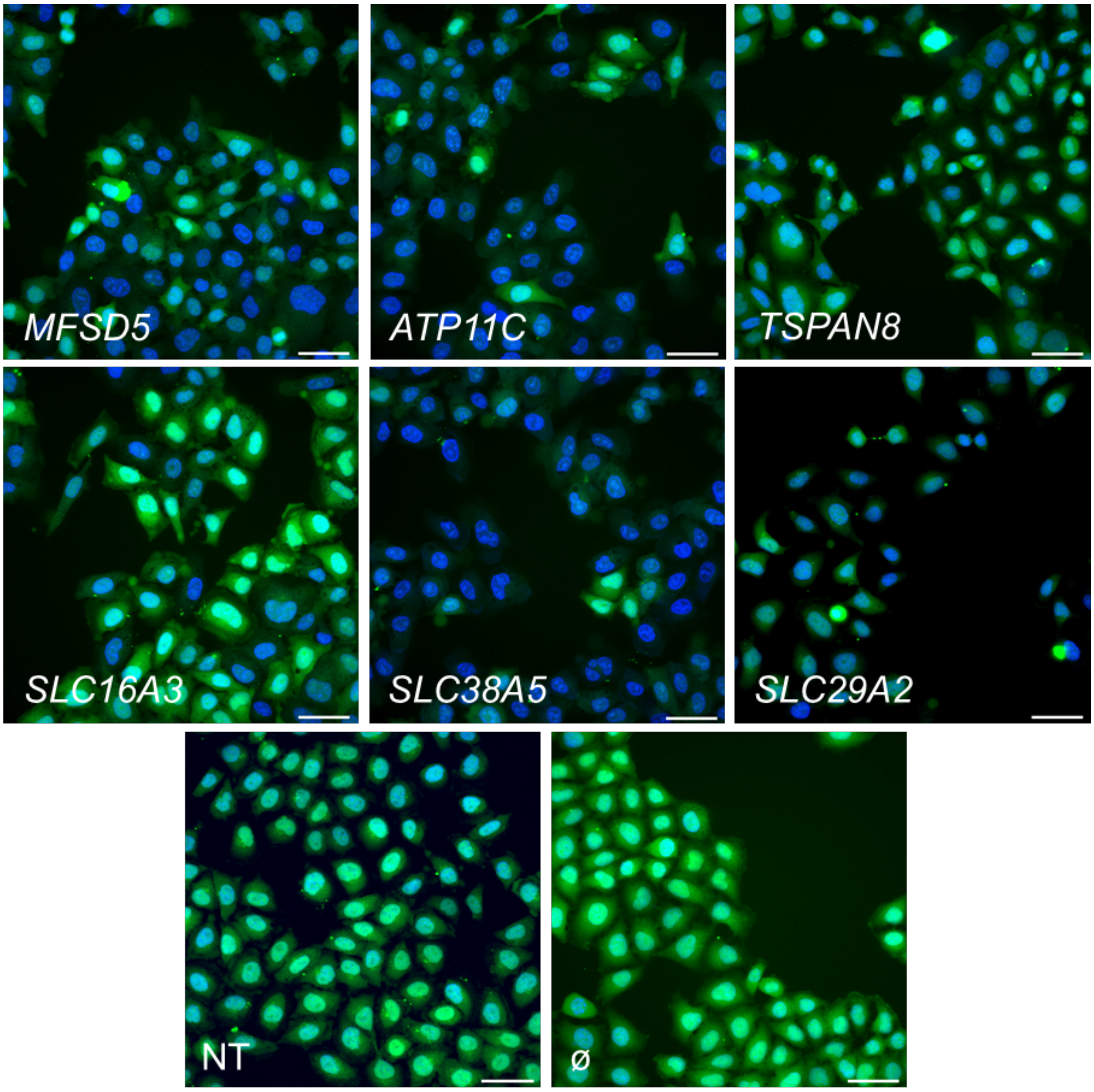

Figure S51. SDCM images (40X WI) showing fluorescence intensity of **11** (5 µM, green) in fixed KD HK cells after incubation for 30 min in L15 (blue: Hoechst 33342, nuclei; scale bar 50 µm; NT: non-target; ø: non-treated).

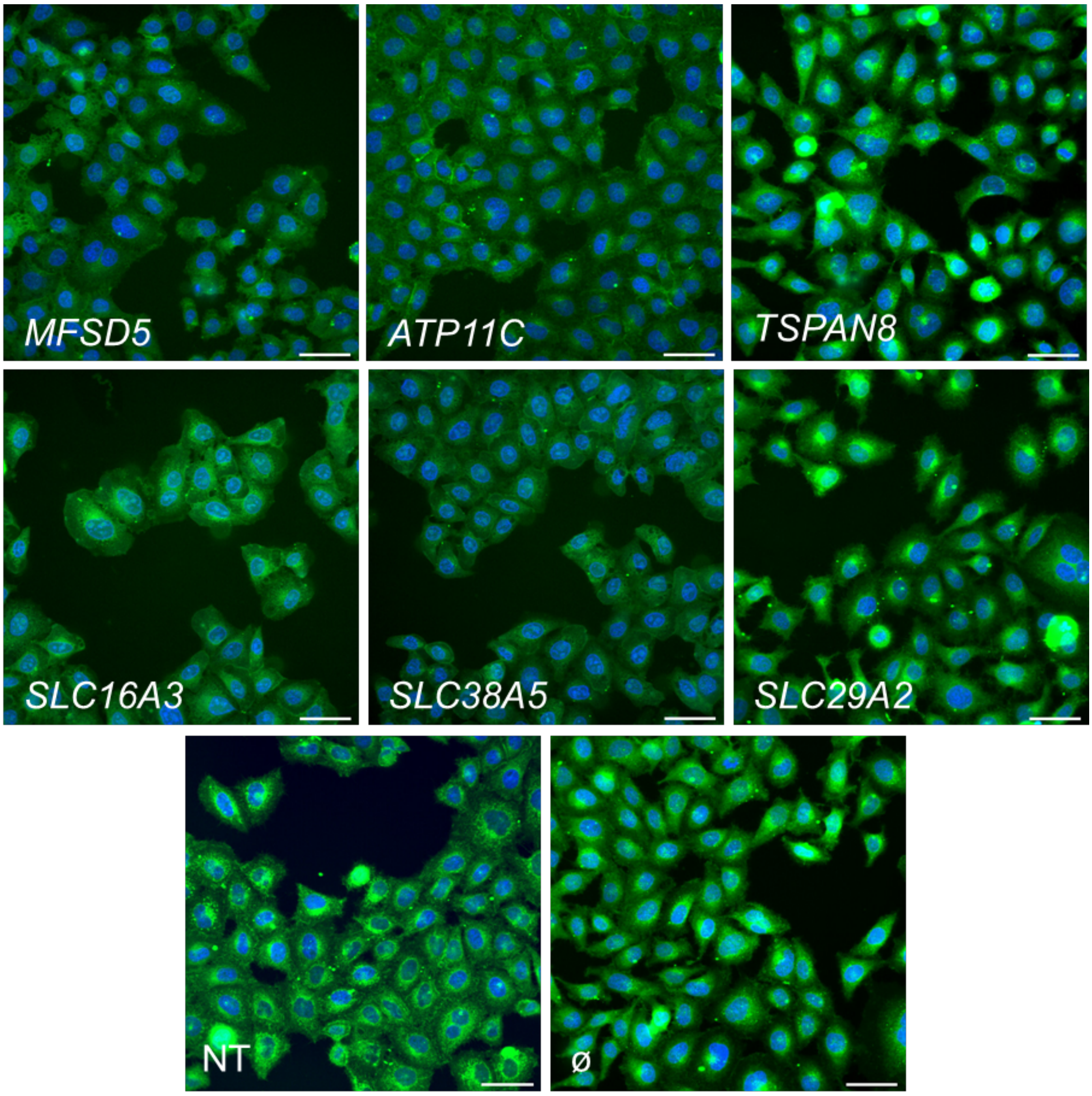

Figure S52. SDCM images (40X WI) showing fluorescence intensity of **12** (5 µM, green) in fixed KD HK cells after incubation for 30 min in L15 (blue: Hoechst 33342, nuclei; scale bar 50 µm; NT: non-target; ø: non-treated).

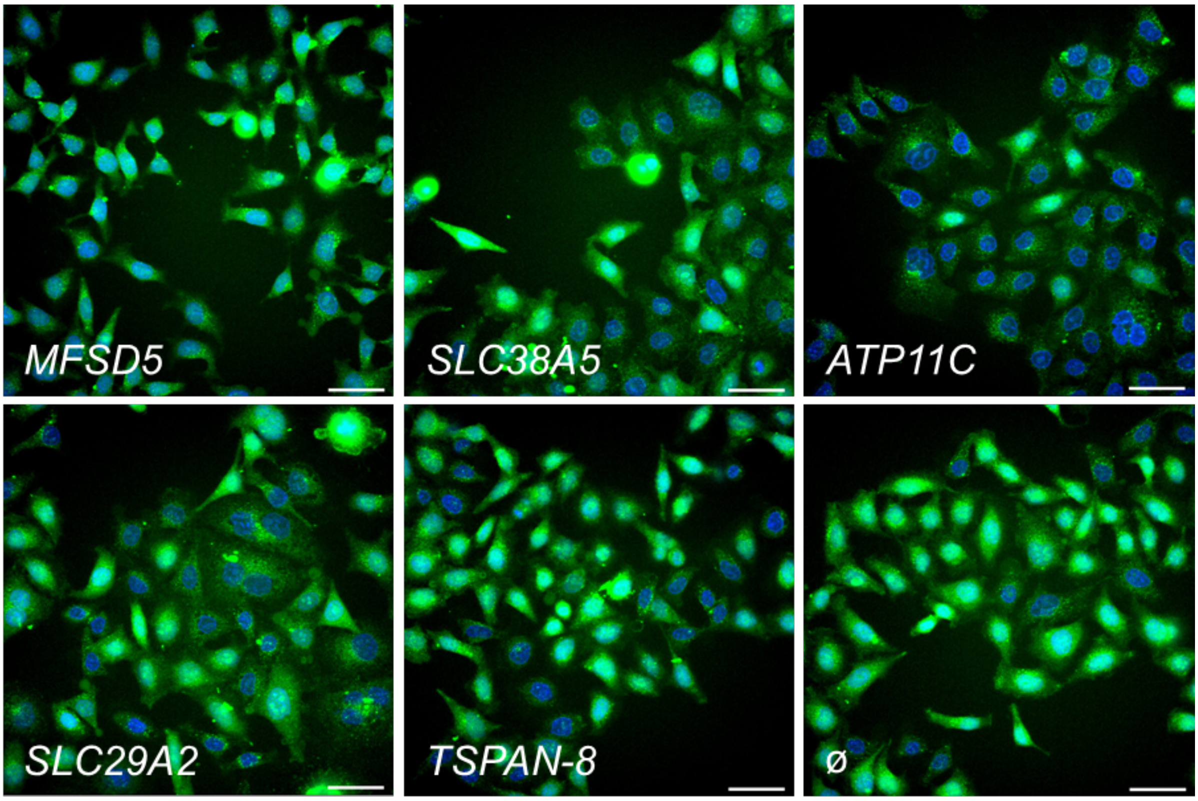

Figure S53. SDCM images (40X WI) showing fluorescence intensity of **13** (10 µM, green) in fixed KD HK cells after incubation for 120 min in L15 (blue: Hoechst 33342, nuclei; scale bar 50 µm; NT: non-target; ø: non-treated).

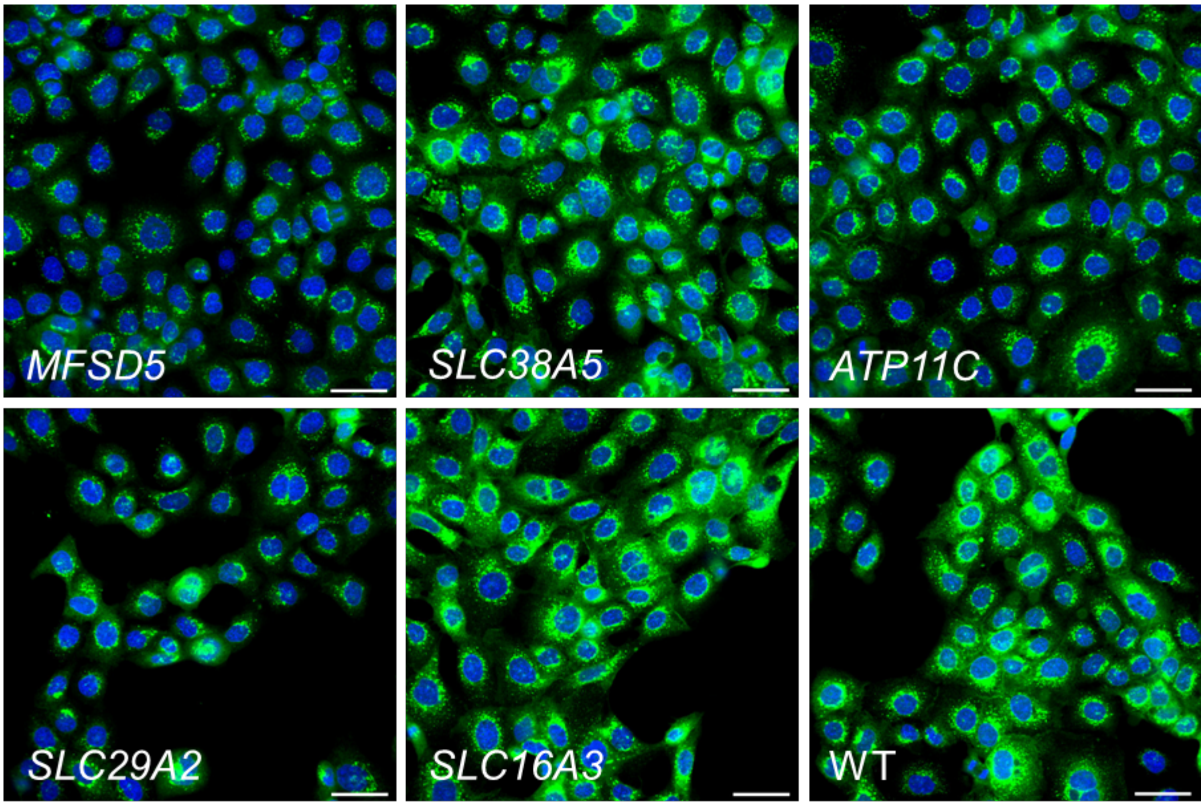

Figure S54. SDCM images (40X WI) showing fluorescence intensity of **10** (10 µM, green) in fixed KD A431 cells after incubation for 30 min in L15 (blue: Hoechst 33342, nuclei; scale bar 50 µm; NT: non-target; ø: non-treated).

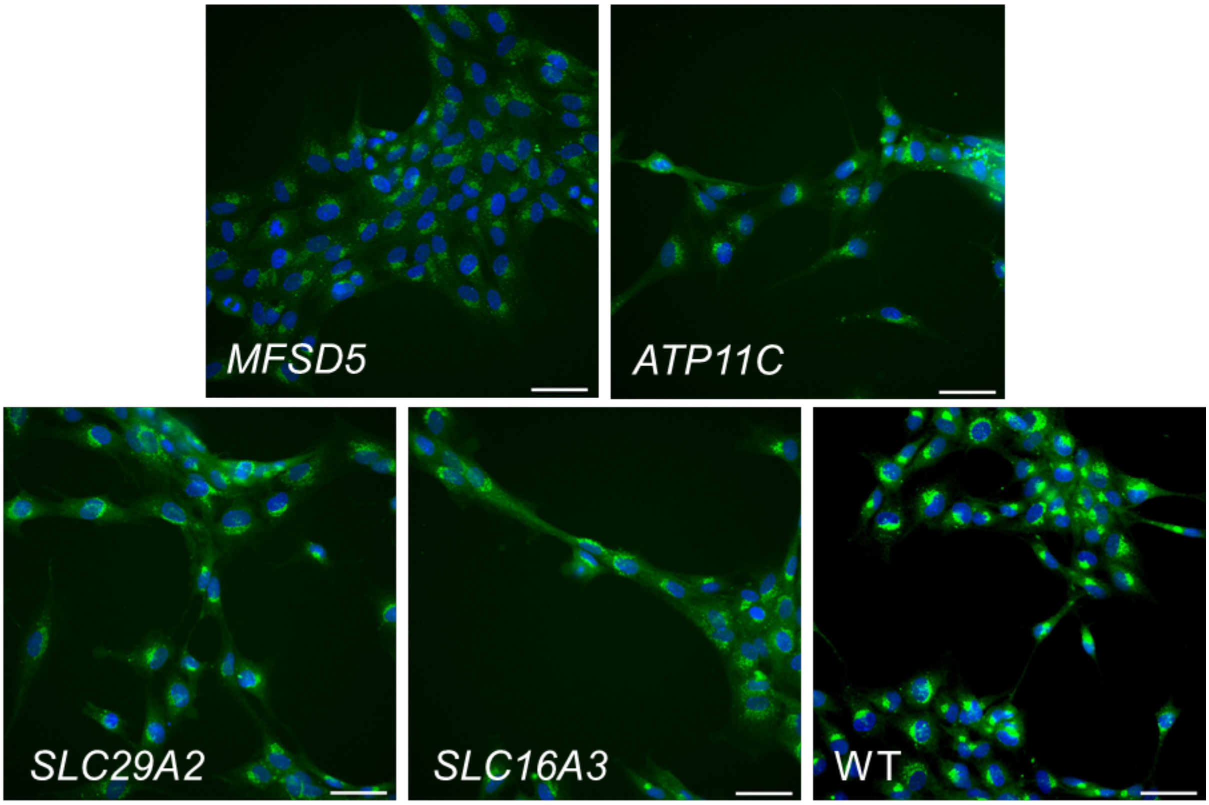

Figure S55. SDCM images (40X WI) showing fluorescence intensity of **10** (10 µM, green) in fixed KD RPE-1 cells after incubation for 30 min in L15 (blue: Hoechst 33342, nuclei; scale bar 50 µm; NT: non-target; ø: non-treated).

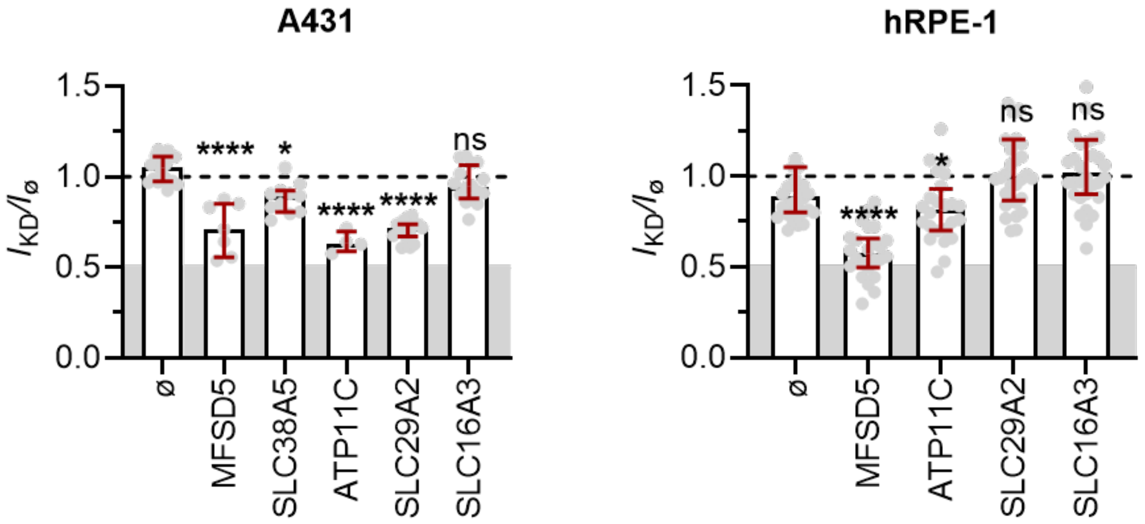

Figure S56. Resulting relative fluorescence intensity $I_{KD}/I_ø$ of **10** (10 µM, 30 min) in knocked down A431 (left) and hRPE-1 (right) cells using the Leibovitz' L15 medium. The x axis refers to knockdown of indicated protein. Each grey circle represents an average cellular fluorescence per cell of an image. On average, 16 images per knockdown. Red horizontal line: median (*P*-value < 0.0001: ****, obtained with ordinary non-parametric one-way ANOVA). ø: non-treated.

15. Supporting References

(S1) Saidjalolov, S.; Coelho, F.; Mercier, V.; Moreau, D.; Matile, S. Inclusive Pattern Generation Protocols to Decode Thiol-Mediated Uptake. *ACS Cent. Sci.* **2024**, *10*, 1033–1043.

(S2) Lampkin, P. P.; Thompson, B. J.; Gellman, S. H. Versatile Open-Source Photoreactor Architecture for Photocatalysis Across the Visible Spectrum. *Org. Lett.* **2021**, *23*, 5277–5281.

(S3) Shybeka, I.; Maynard, J. R. J.; Saidjalolov, S.; Moreau, D.; Sakai, N.; Matile, S. Dynamic Covalent Michael Acceptors to Penetrate Cells: Thiol-Mediated Uptake with Tetrel-Centered Exchange Cascades, Assisted by Halogen-Bonding Switches. *Angew. Chem. Int. Ed.* **2022**, *61*, e202213433.

(S4) Lim, B.; Kato, T.; Besnard, C.; Poblador Bahamonde, A. I.; Sakai, N.; Matile, S. Pnictogen-Centered Cascade Exchangers for Thiol-Mediated Uptake: As(III)-, Sb(III)-, and Bi(III)-Expanded Cyclic Disulfides as Inhibitors of Cytosolic Delivery and Viral Entry. *JACS Au* **2022**, *2*, 1105–1114.

(S5) Jin, W. B.; Xu, C.; Cheng, Q.; Qi, X. L.; Gao, W.; Zheng, Z.; Chan, E. W. C.; Leung, Y.-C.; Chan, T. H.; Wong, K.-Y.; Chen, S.; Chan, K.-F. Investigation of Synergistic Antimicrobial Effects of the Drug Combinations of Meropenem and 1,2-Benzisoselenazol-3(2*H*)-One Derivatives on Carbapenem-Resistant *Enterobacteriaceae* Producing NDM-1. *Eur. J. Med. Chem.* **2018**, *155*, 285–302.

(S6) Gasparini, G.; Sargsyan, G.; Bang, E.-K.; Sakai, N.; Matile, S. Ring Tension Applied to Thiol-Mediated Cellular Uptake. *Angew. Chem. Int. Ed.* **2015**, *54*, 7328–7331.

(S7) Zong, L.; Bartolami, E.; Abegg, D.; Adibekian, A.; Sakai, N.; Matile, S. Epidithiodiketopiperazines: Strain-Promoted Thiol-Mediated Cellular Uptake at the Highest Tension. *ACS Cent. Sci.* **2017**, *3*, 449–453.

(S8) Cheng, Y.; Zong, L.; López-Andarias, J.; Bartolami, E.; Okamoto, Y.; Ward, T. R.; Sakai, N.; Matile, S. Cell-Penetrating Dynamic-Covalent Benzopolysulfane Networks. *Angew. Chem. Int. Ed.* **2019**, *58*, 9522–9526.

(S9) López-Andarias, J.; Saarbach, J.; Moreau, D.; Cheng, Y.; Derivery, E.; Laurent, Q.; González-Gaitán, M.; Winssinger, N.; Sakai, N.; Matile, S. Cell-Penetrating Streptavidin: A General Tool for Bifunctional Delivery with Spatiotemporal Control, Mediated by Transport Systems Such as Adaptive Benzopolysulfane Networks. *J. Am. Chem. Soc.* **2020**, *142*, 4784–4792.

(S10) (a) Lowry, M. S.; Goldsmith, J. I.; Slinker, J. D.; Rohl, R.; Pascal, R. A.; Malliaras, G. G.; Bernhard, S. Single-Layer Electroluminescent Devices and Photoinduced Hydrogen Production from an Ionic Iridium(III) Complex. *Chem. Mater.* **2005**, *17*, 5712–5719. (b) Miller, D. C.; Ganley, J. M.; Musacchio, A. J.; Sherwood, T. C.; Ewing, W. R.; Knowles, R. R. Anti-Markovnikov Hydroamination of Unactivated Alkenes with Primary Alkyl Amines. *J. Am. Chem. Soc.* **2019**, *141*, 16590–16594.

(S11) Łomzik, M.; Mazuryk, O.; Rutkowska-Zbik, D.; Stochel, G.; Gros, P. C.; Brindell, M. New Ruthenium Compounds Bearing Semicarbazone 2-Formylopyridine Moiety: Playing with Auxiliary Ligands for Tuning the Mechanism of Biological Activity. *J. Inorg. Biochem.* **2017**, *175*, 80–91.

(S12) Fabre, B.; Pícha, J.; Vaněk, V.; Selicharová, I.; Chrudinová, M.; Collinsová, M.; Žáková, L.; Buděšínský, M.; Jiráček, J. Synthesis and Evaluation of a Library of Trifunctional Scaffold-Derived Compounds as Modulators of the Insulin Receptor. *ACS Comb. Sci.* **2016**, *18*, 710–722.

(S13) Saidjalolov, S.; Chen, X.-X.; Moreno, J.; Cognet, M.; Wong-Dilworth, L.; Bottanelli, F.; Sakai, N.; Matile, S. Asparagusic Golgi Trackers. *JACS Au* **2024**, *4*, 3759–3765.

(S14) Geri, J. B.; Oakley, J. V.; Reyes-Robles, T.; Wang, T.; McCarver, S. J.; White, C. H.; Rodriguez-Rivera, F. P.; Parker, D. L.; Hett, E. C.; Fadeyi, O. O.; Oslund, R. C.; MacMillan, D. W.

C. Microenvironment Mapping via Dexter Energy Transfer on Immune Cells. *Science* **2020**, *367*, 1091–1097.

(S15) Aguilan, J. T.; Kulej, K.; Sidoli, S. Guide for Protein Fold Change and P-Value Calculation for Non-Experts in Proteomics. *Mol. Omics* **2020**, *16*, 573–582.

(S16) Yan, T.; Boatner, L. M.; Cui, L.; Tontonoz, P. J.; Backus, K. M. Defining the Cell Surface Cysteinome Using Two-Step Enrichment Proteomics. *JACS Au* **2023**, *3*, 3506–3523.

(S17) Szklarczyk, D.; Kirsch, R.; Koutrouli, M.; Nastou, K.; Mehryary, F.; Hachilif, R.; Gable, A. L.; Fang, T.; Doncheva, N. T.; Pyysalo, S.; Bork, P.; Jensen, L. J.; von Mering, C. The STRING Database in 2023: Protein–Protein Association Networks and Functional Enrichment Analyses for Any Sequenced Genome of Interest. *Nucleic Acids Res.* **2023**, *51*, D638–D646.

16. NMR Spectra

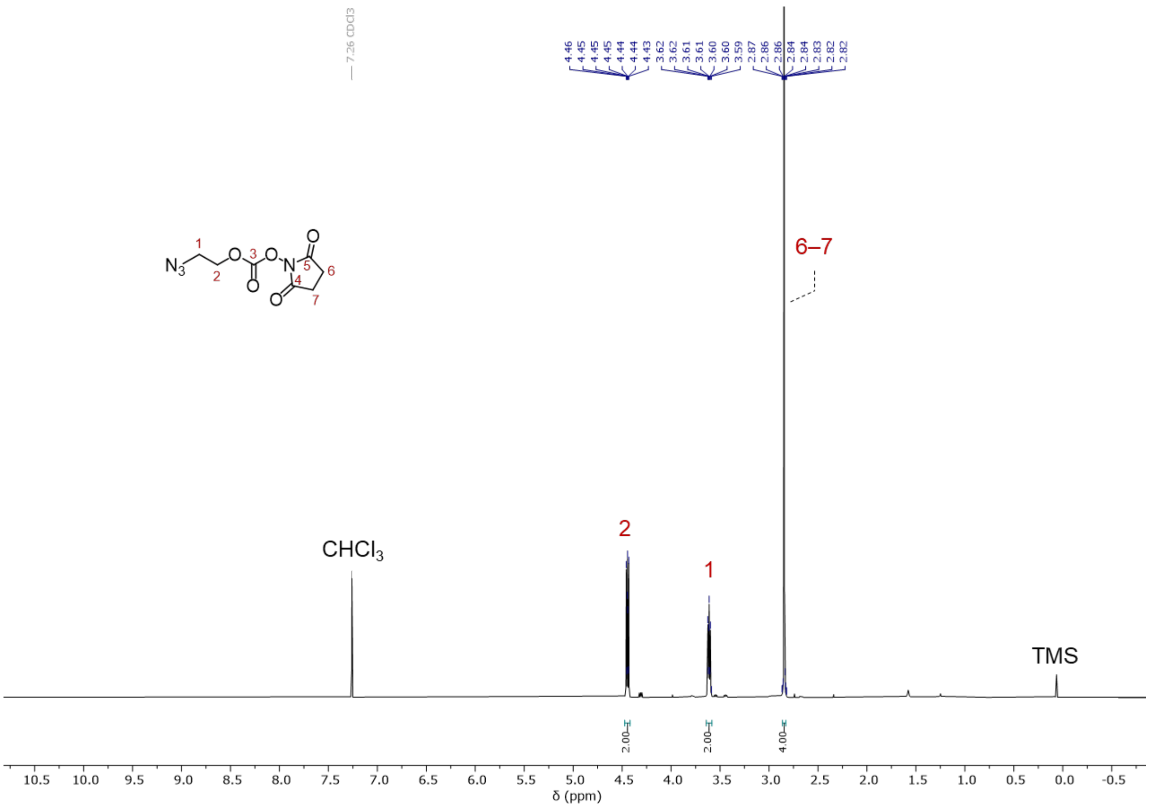

Figure S57. 400 MHz ^{1}H NMR spectrum of compound **20** in $CDCl_3$.

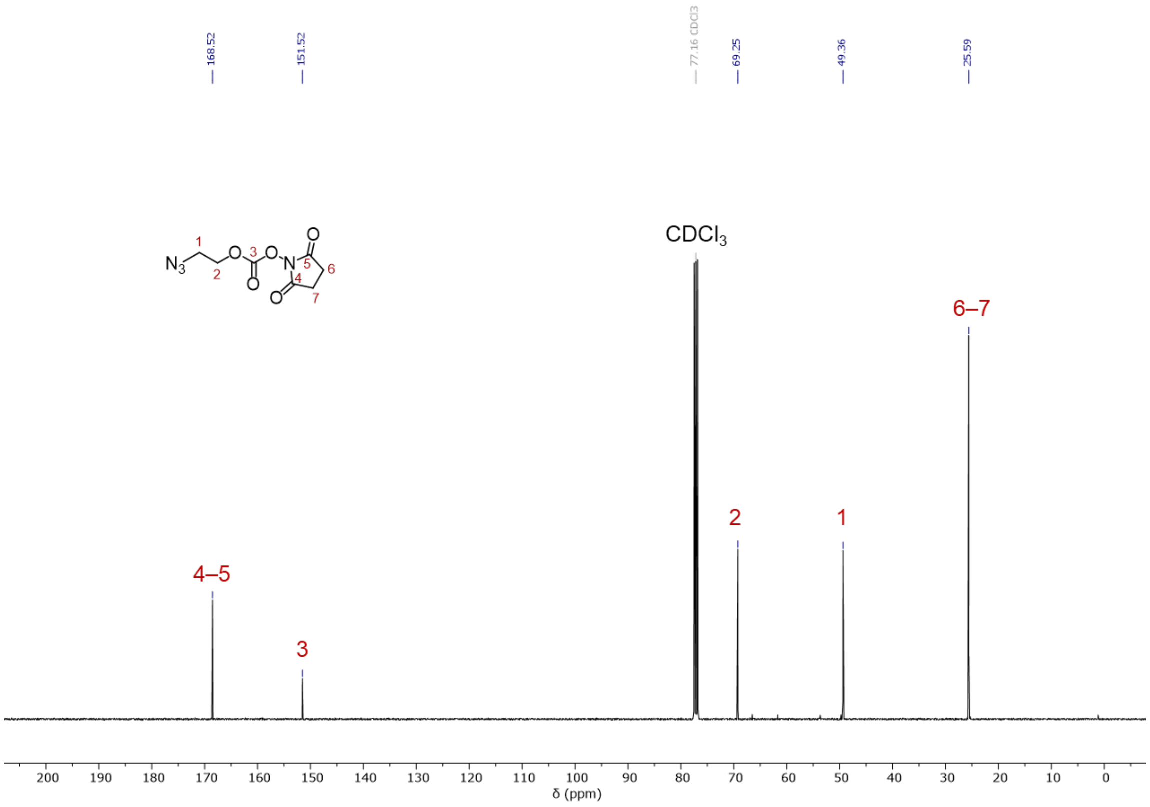

Figure 58. 101 MHz ^{13}C NMR spectrum of compound **20** in $CDCl_3$.

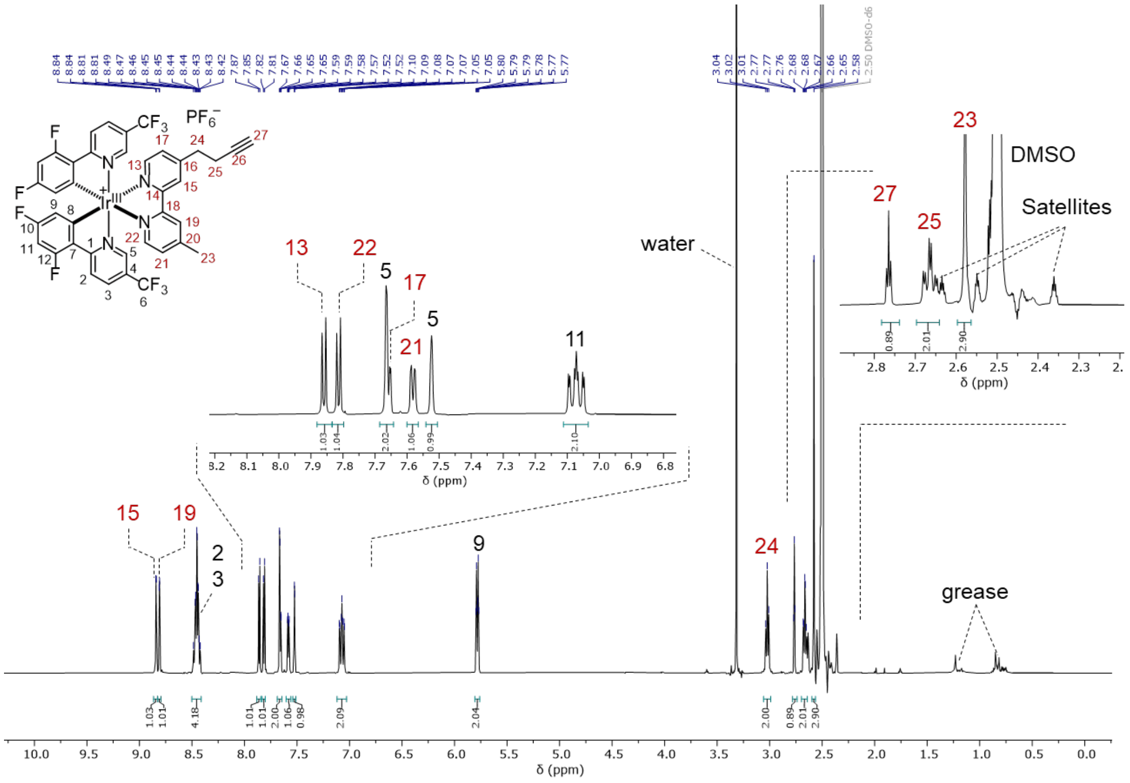

Figure S59. 500 MHz ^{1}H NMR spectrum of compound **37** in DMSO-d_6.

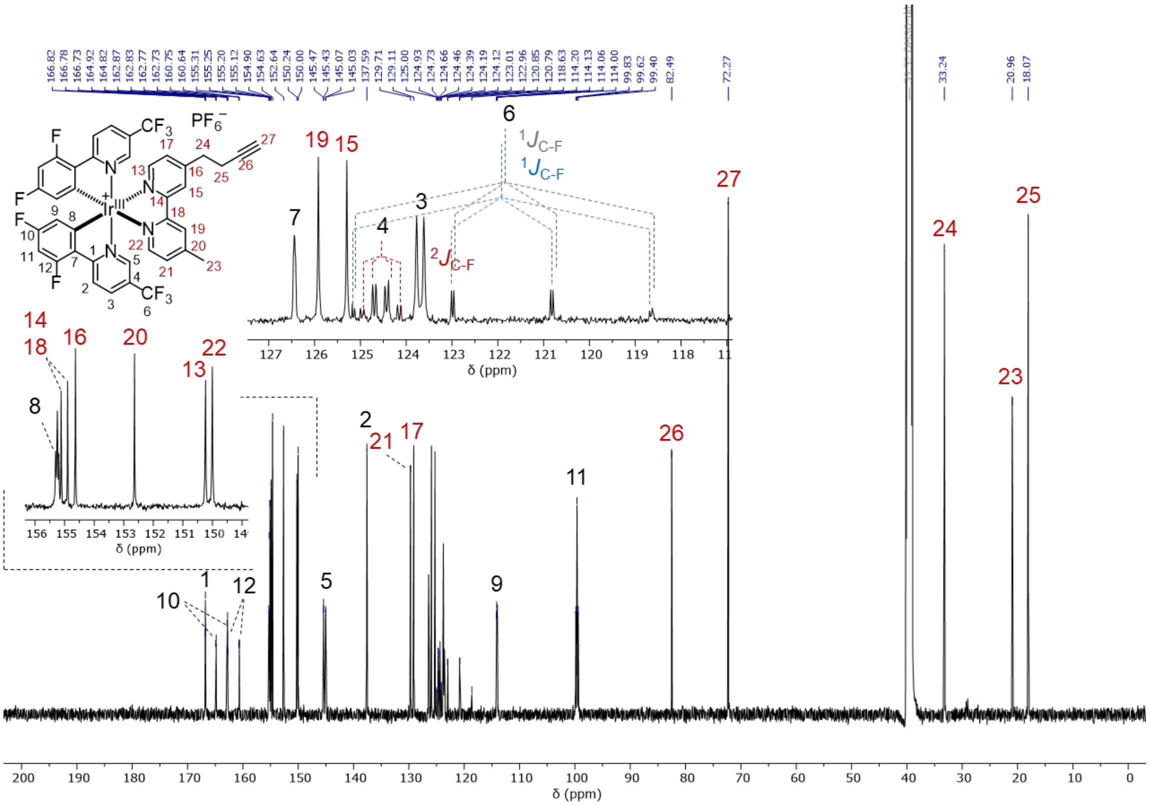

Figure S60. 126 MHz ^{13}C NMR spectrum of compound **37** in DMSO-d_6.

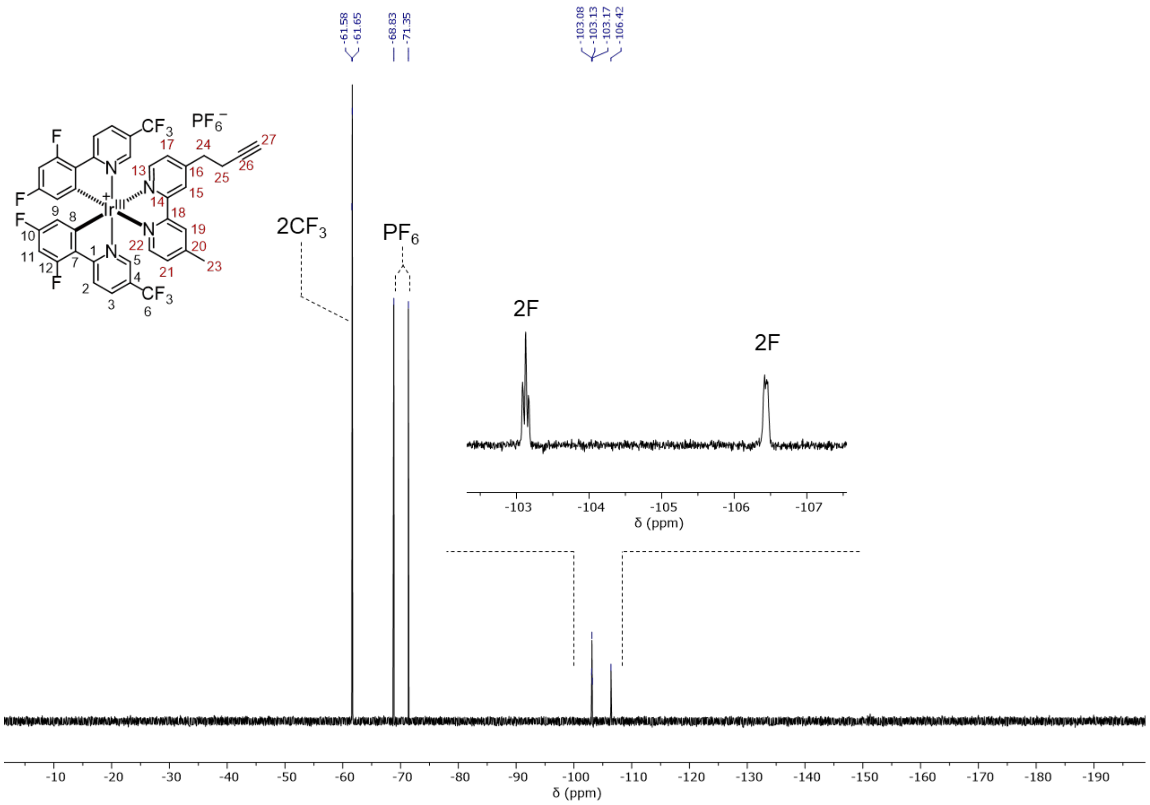

Figure S61. 282 MHz ^{19}F NMR spectrum of compound **37** in DMSO-d_6.

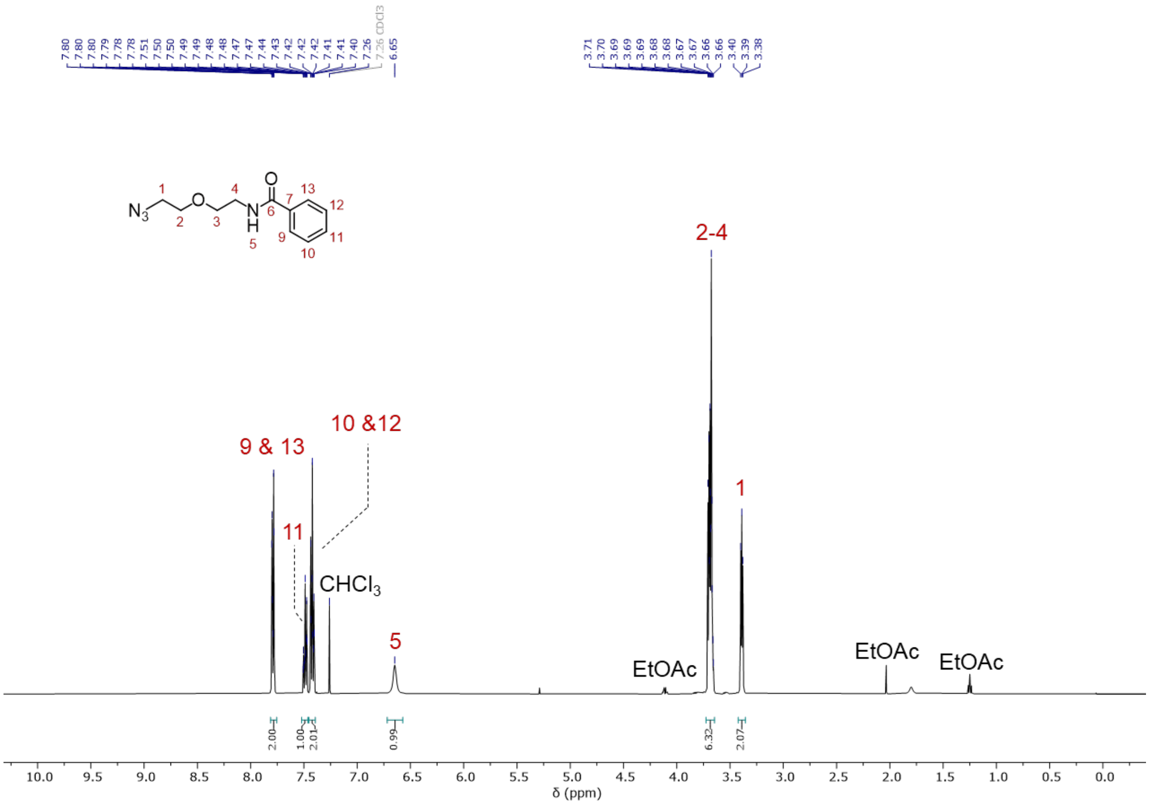

Figure S62. 500 MHz ^{1}H NMR spectrum of compound **40** in $CDCl_3$.

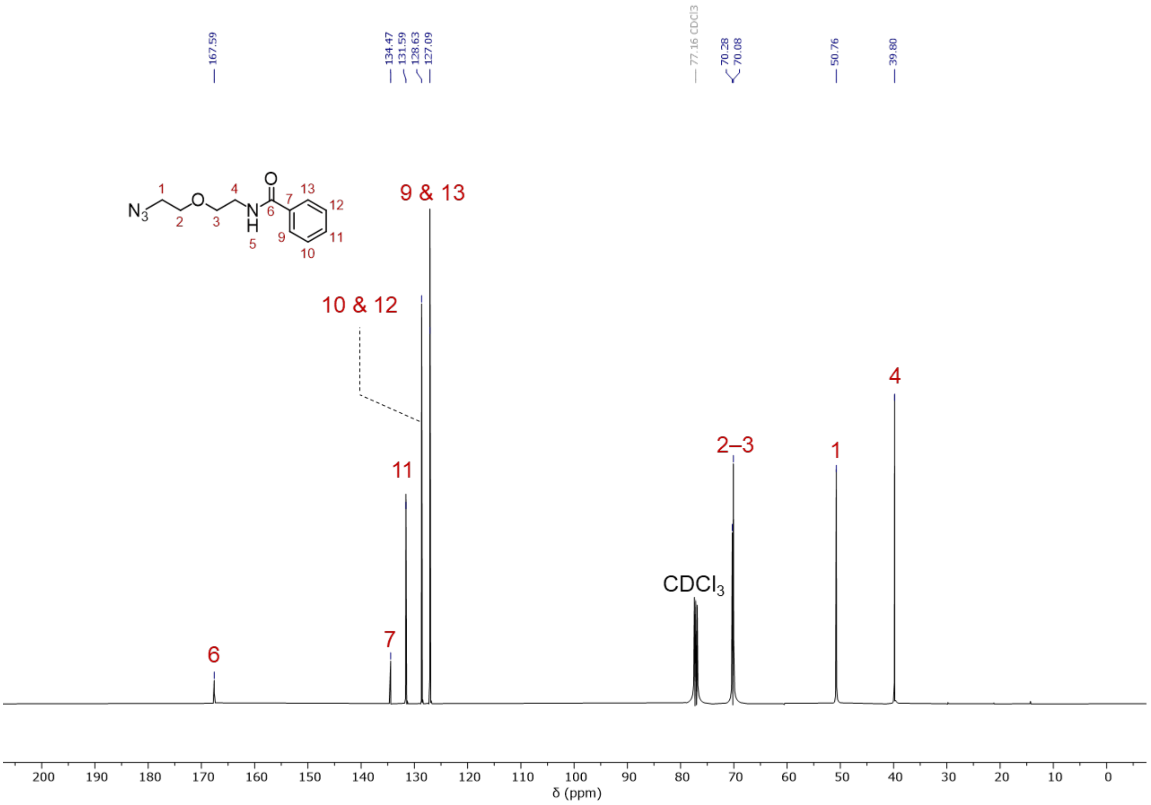

Figure S63. 126 MHz ^{13}C NMR spectrum of compound **40** in $CDCl_3$.

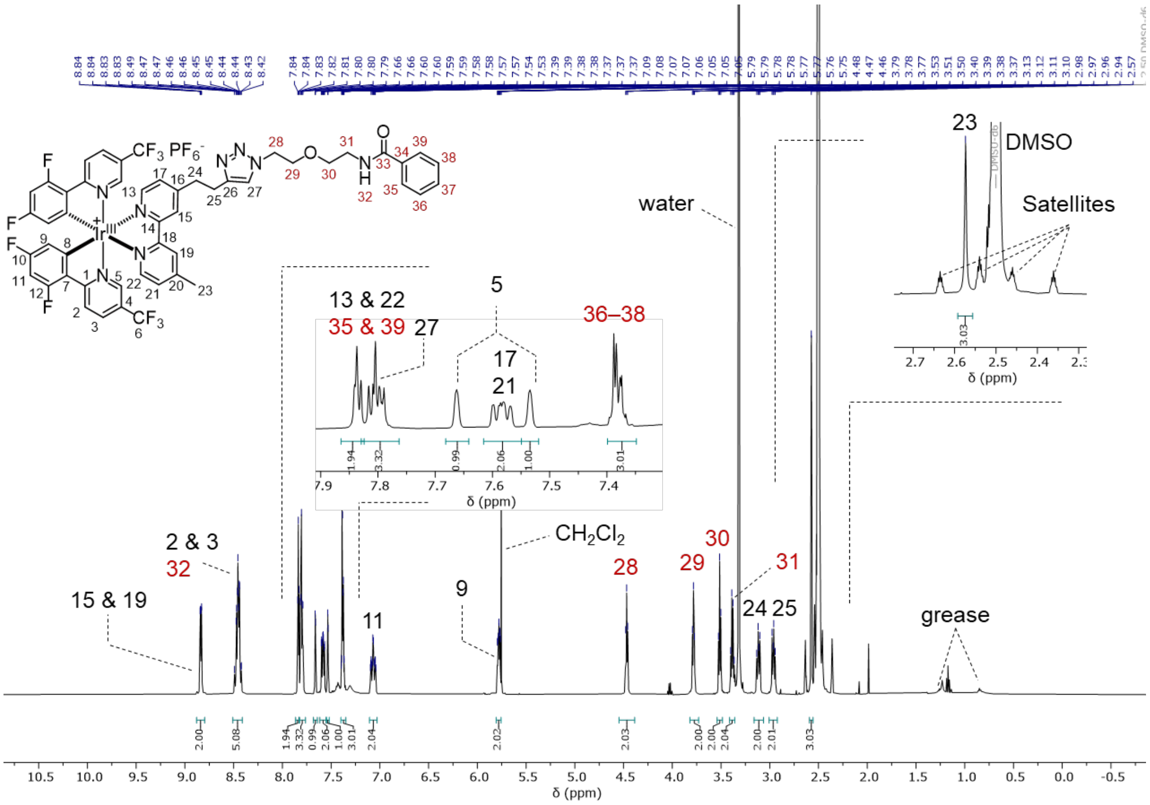

Figure S64. 500 MHz ^{1}H NMR spectrum of compound **3** in DMSO-d_6.

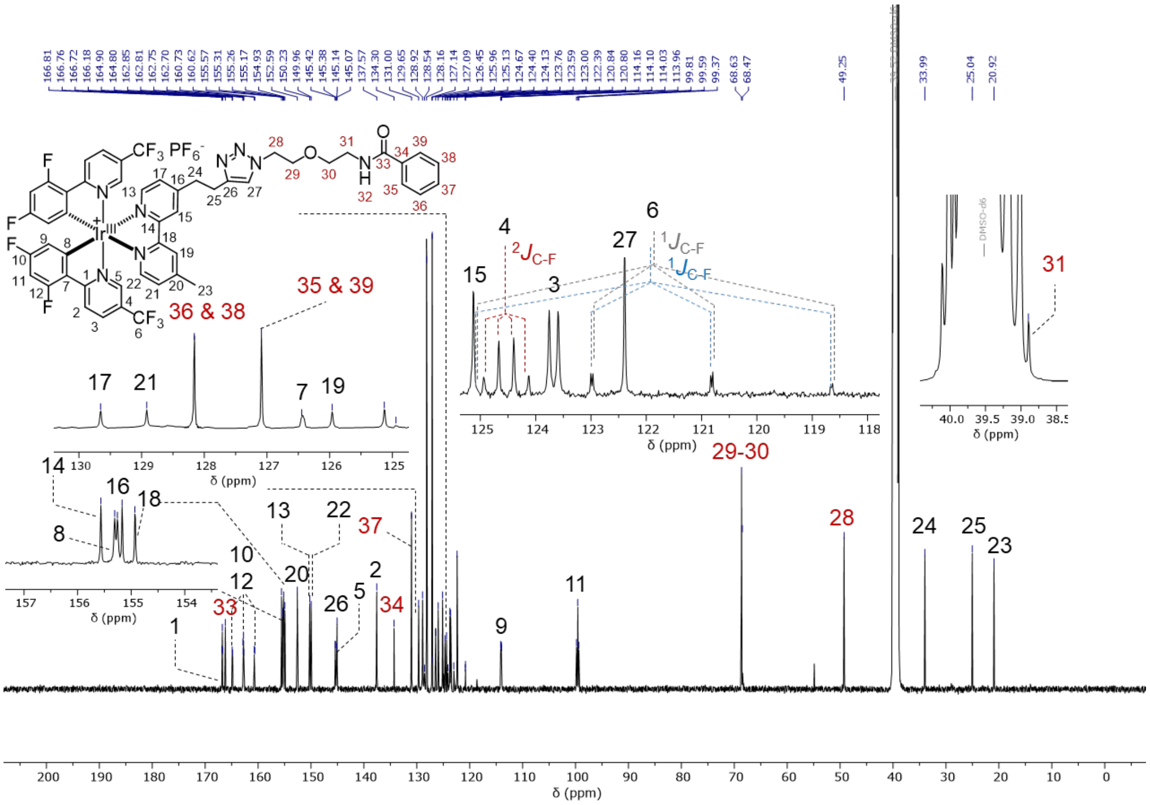

Figure S65. 126 MHz ^{13}C NMR spectrum of compound **3** in DMSO-d_6.

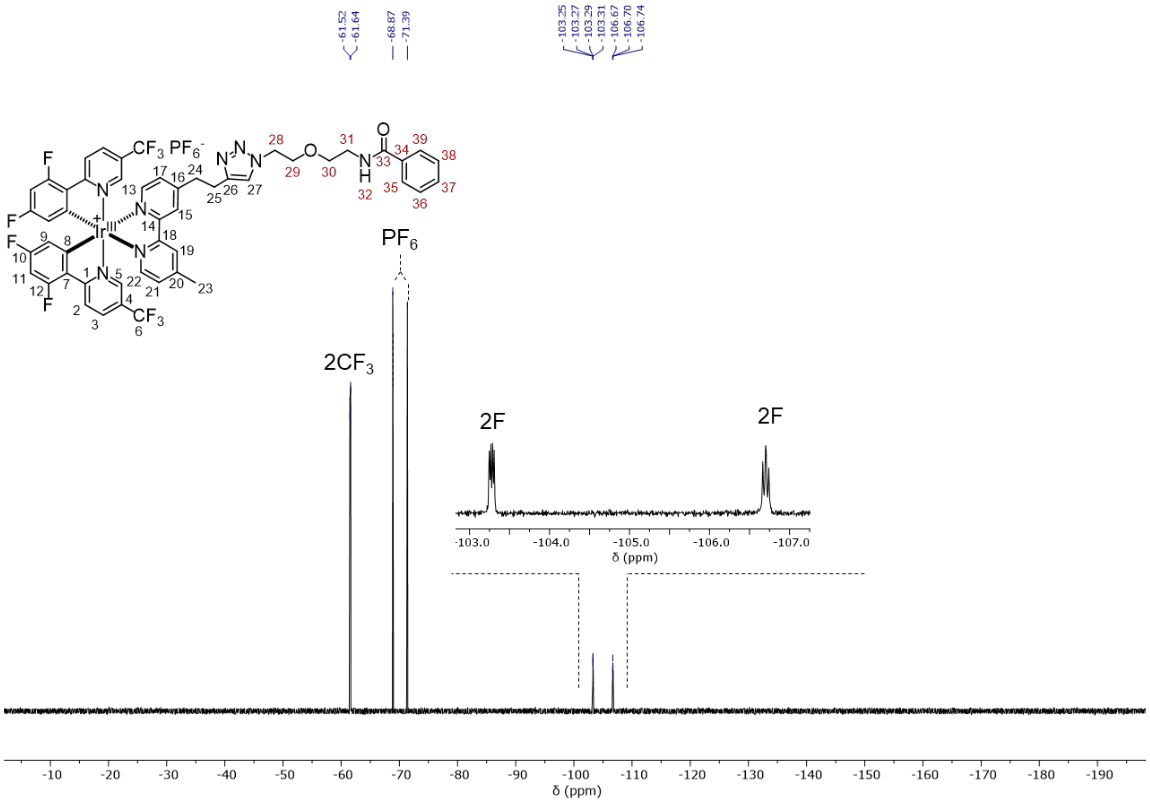

Figure S66. 282 MHz ^{19}F NMR spectrum of compound **3** in DMSO-d_6.

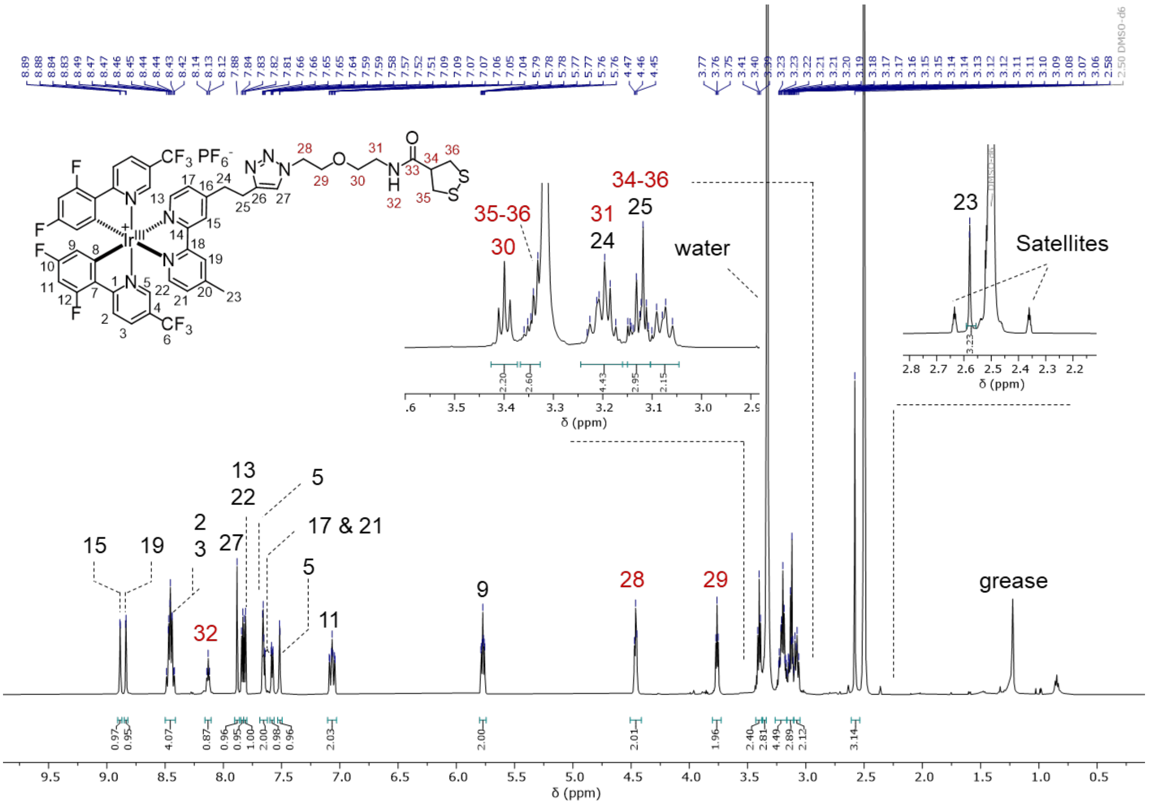

Figure S67. 500 MHz ^{1}H NMR spectrum of compound **1** in DMSO-d_6.

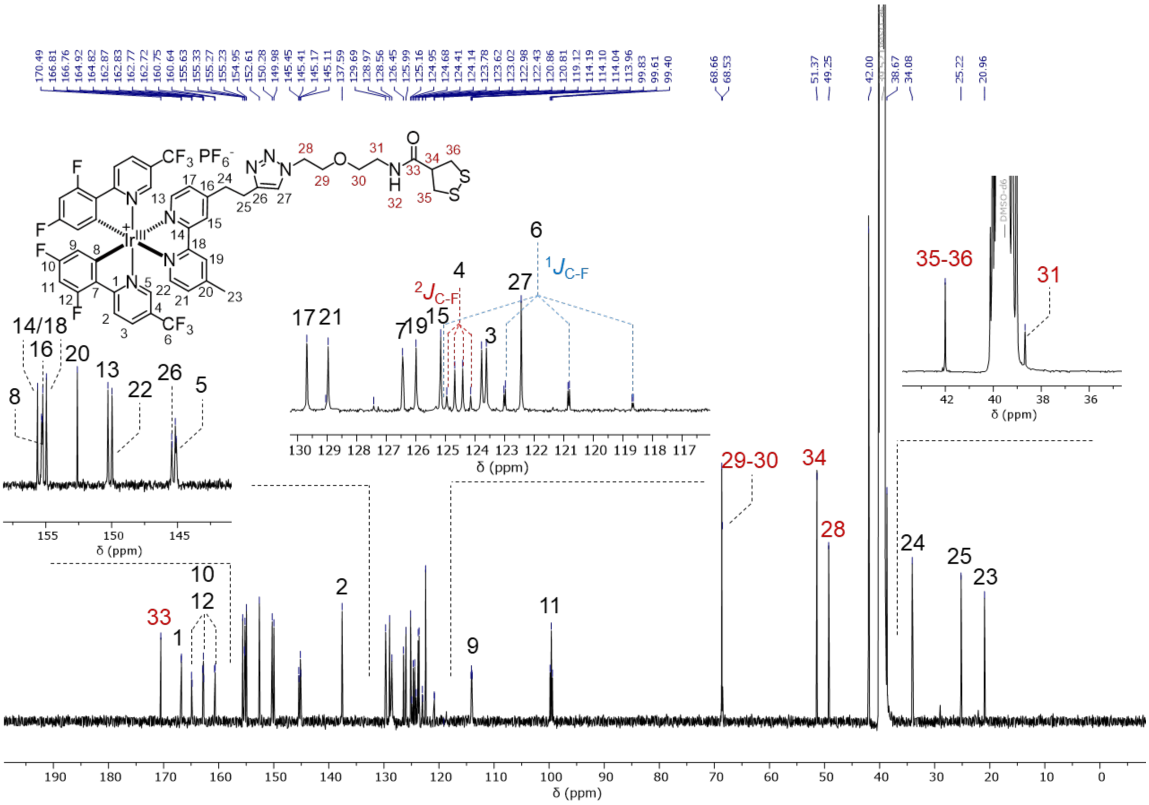

Figure S68. 126 MHz ^{13}C NMR spectrum of compound **1** in DMSO-d_6.

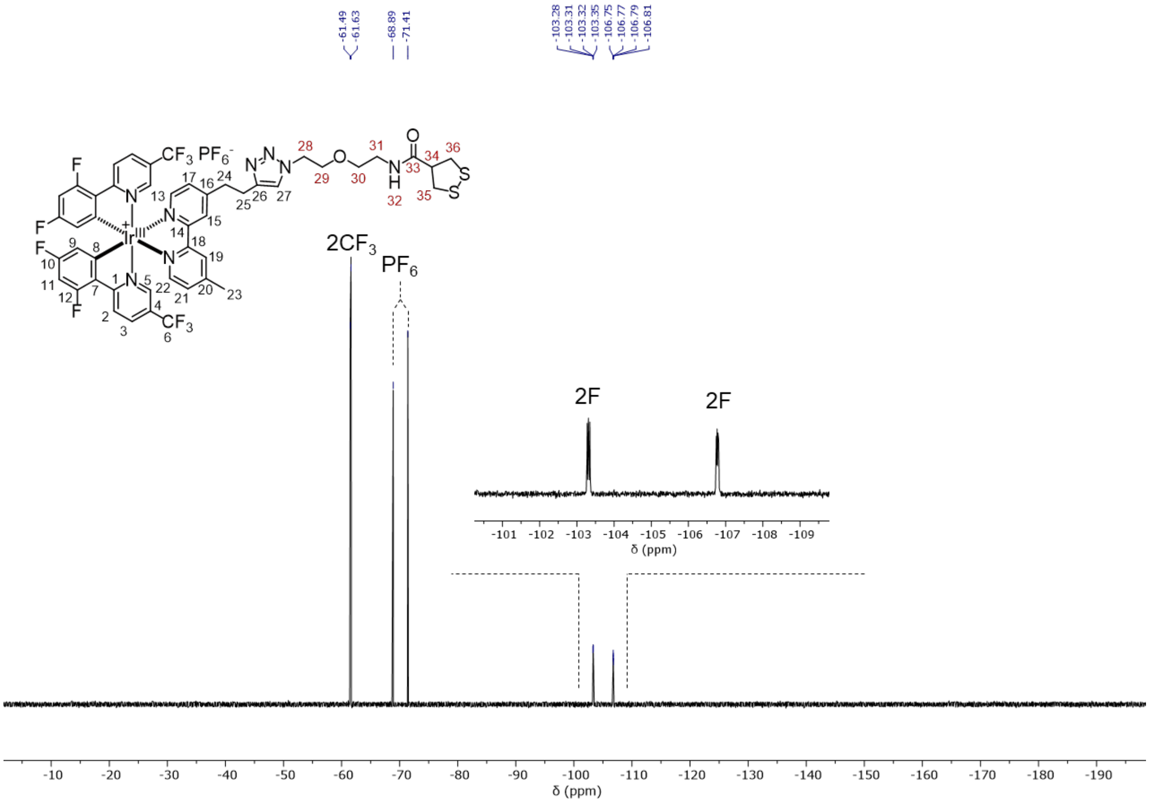

Figure S69. 282 MHz ^{19}F NMR spectrum of compound **1** in DMSO-d_6.

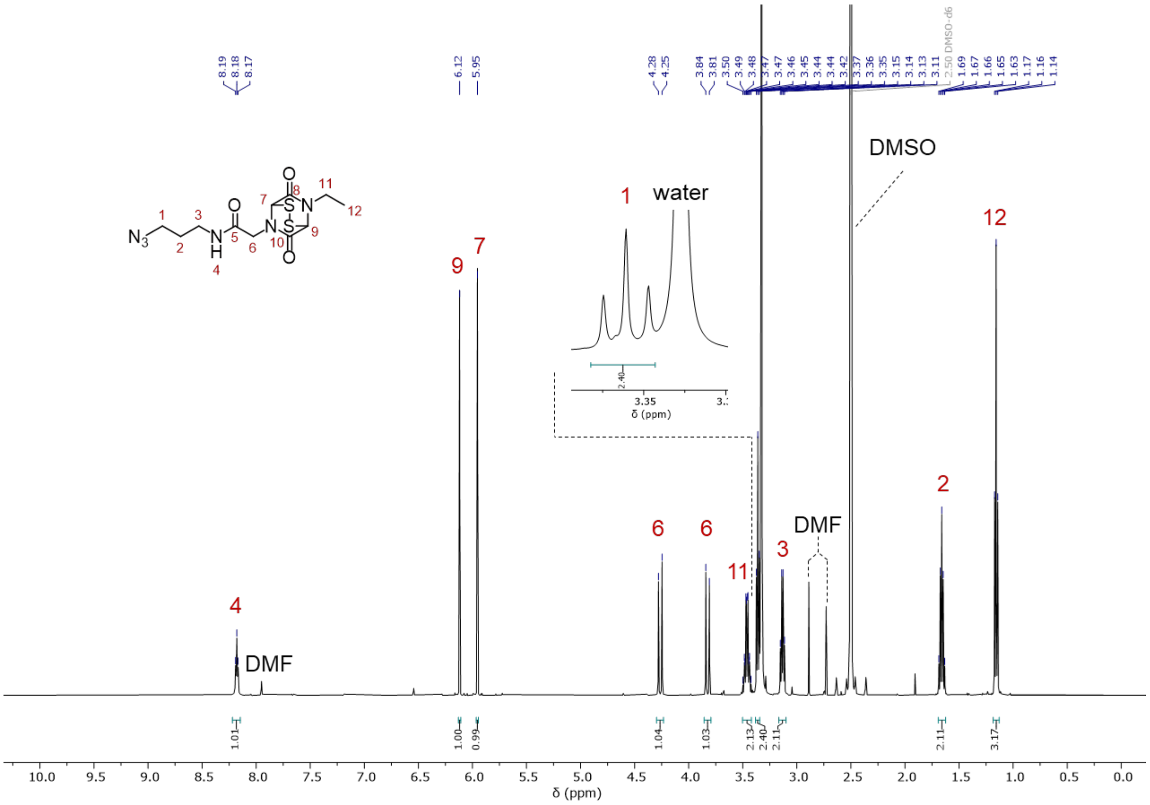

Figure S70. 500 MHz ^{1}H NMR spectrum of compound **44** in DMSO-d_6.

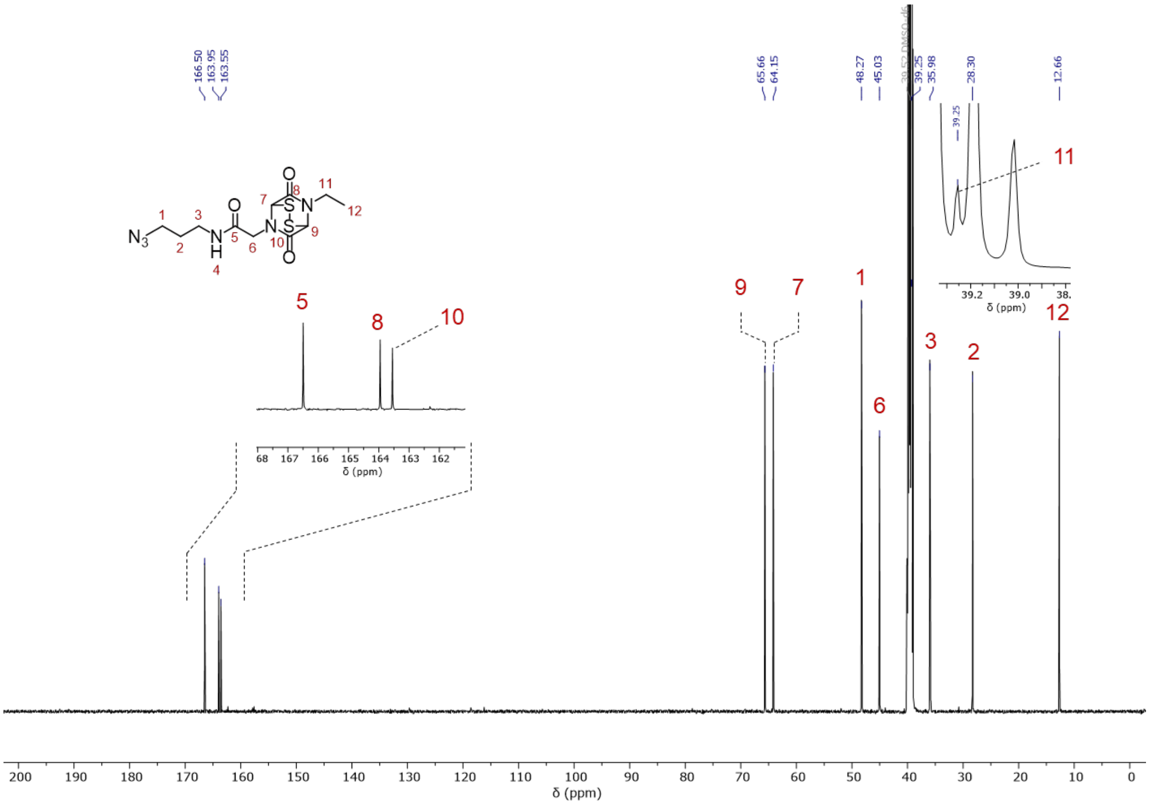

Figure S71. 126 MHz ^{13}C NMR spectrum of compound **44** in DMSO-d_6.

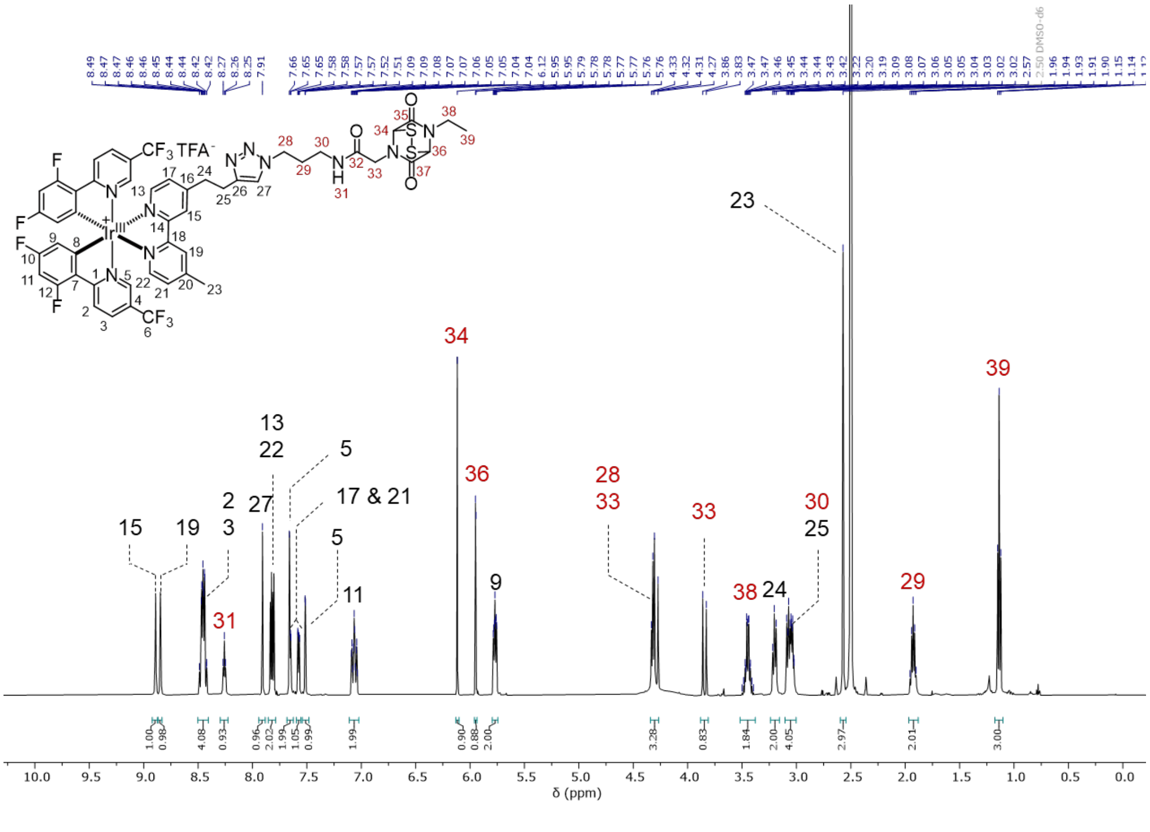

Figure S72. 500 MHz ^{1}H NMR spectrum of compound **4** in DMSO-d_6.

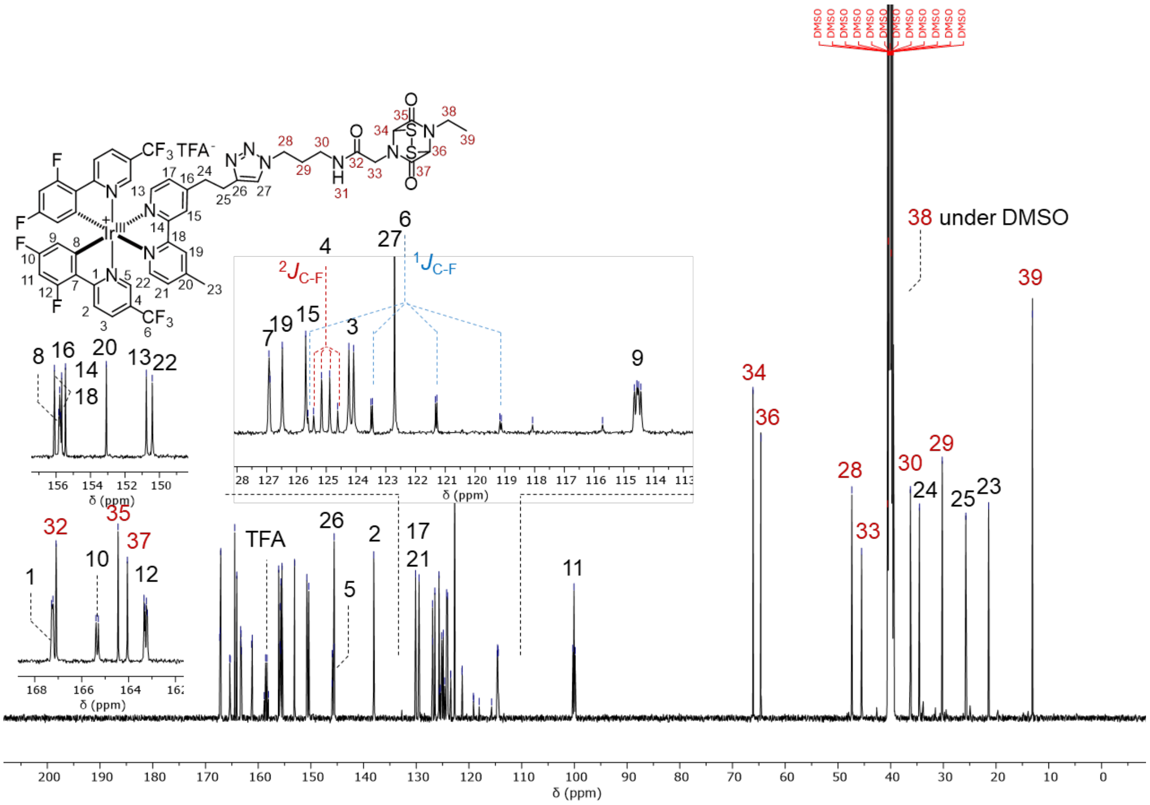

Figure S73. 126 MHz ^{13}C NMR spectrum of compound **4** in DMSO-d_6.

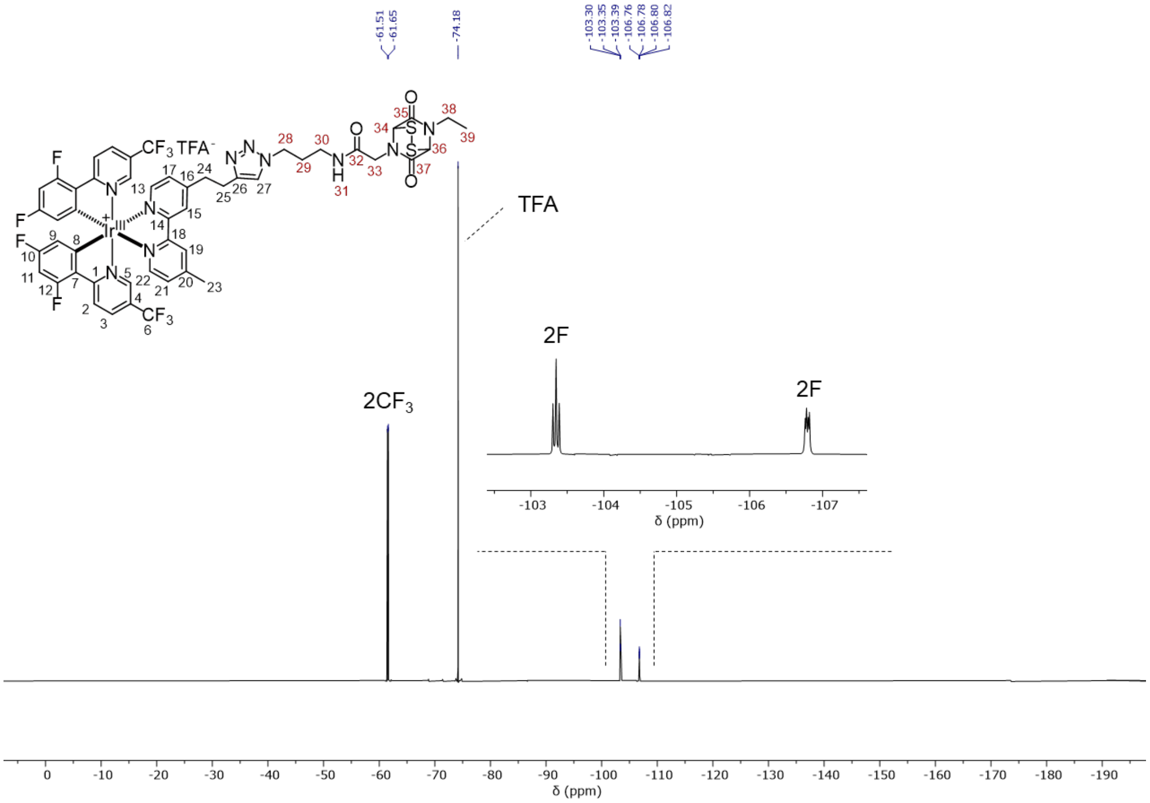

Figure S74. 282 MHz ^{19}F NMR spectrum of compound **4** in DMSO-d_6.

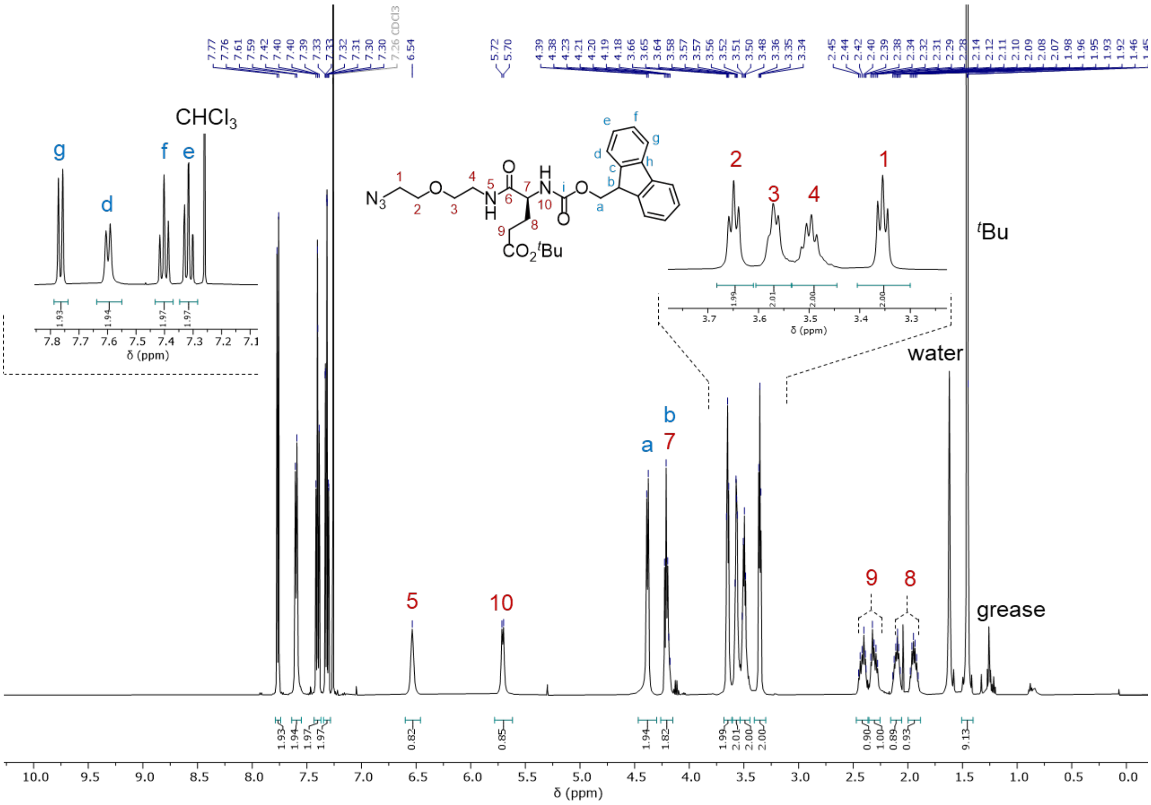

Figure S75. 500 MHz ^{1}H NMR spectrum of compound **46** in $CDCl_3$.

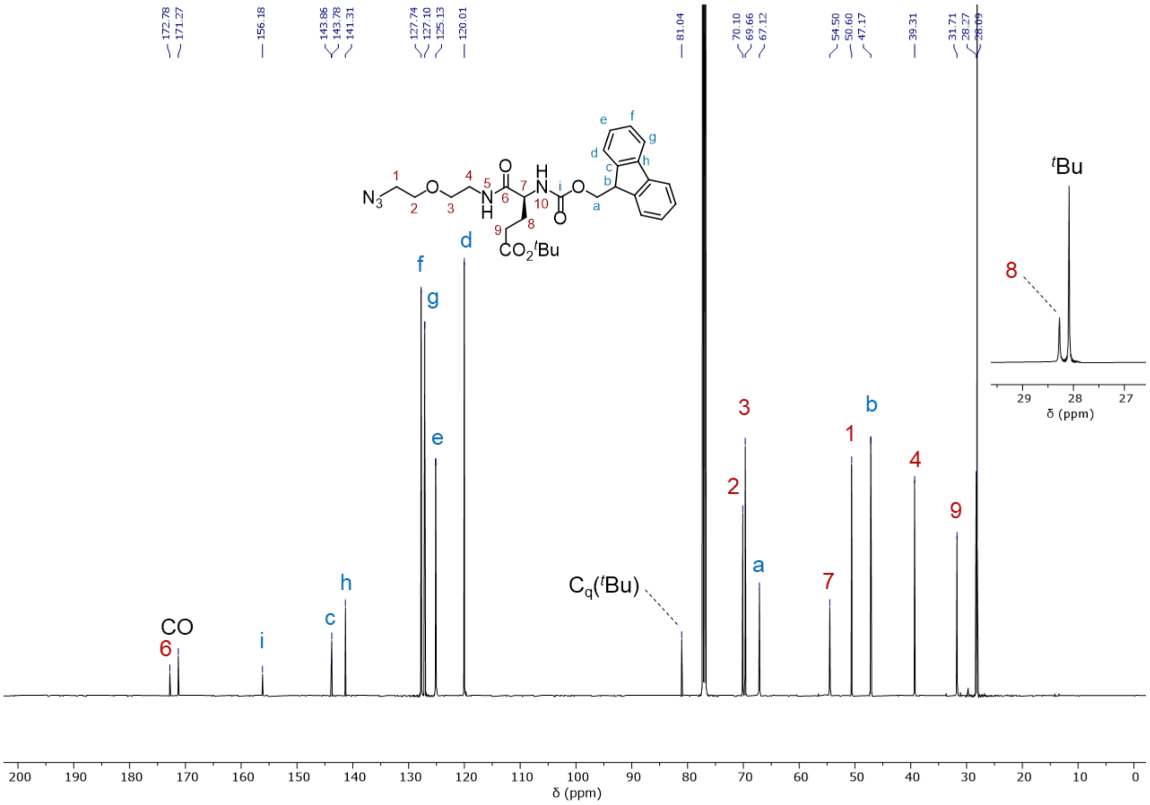

Figure S76. 126 MHz ^{13}C NMR spectrum of compound **46** in $CDCl_3$.

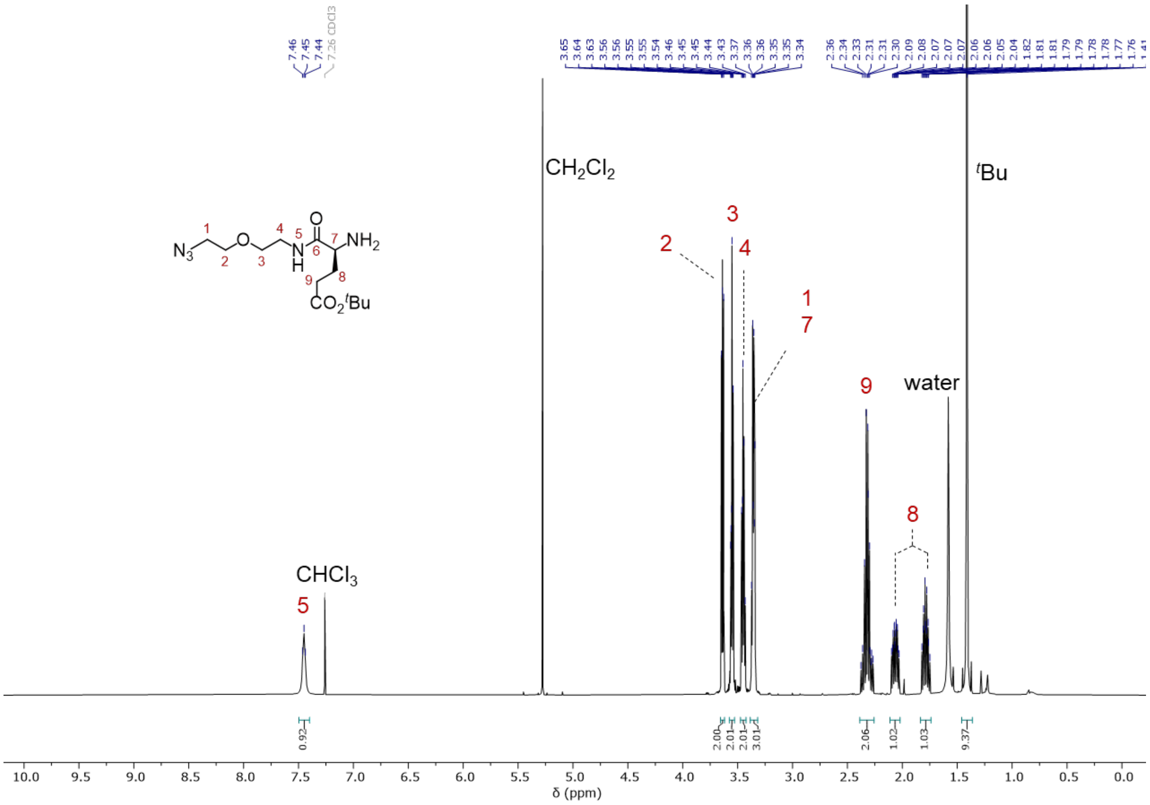

Figure S77. 500 MHz ^{1}H NMR spectrum of compound **47** in $CDCl_3$.

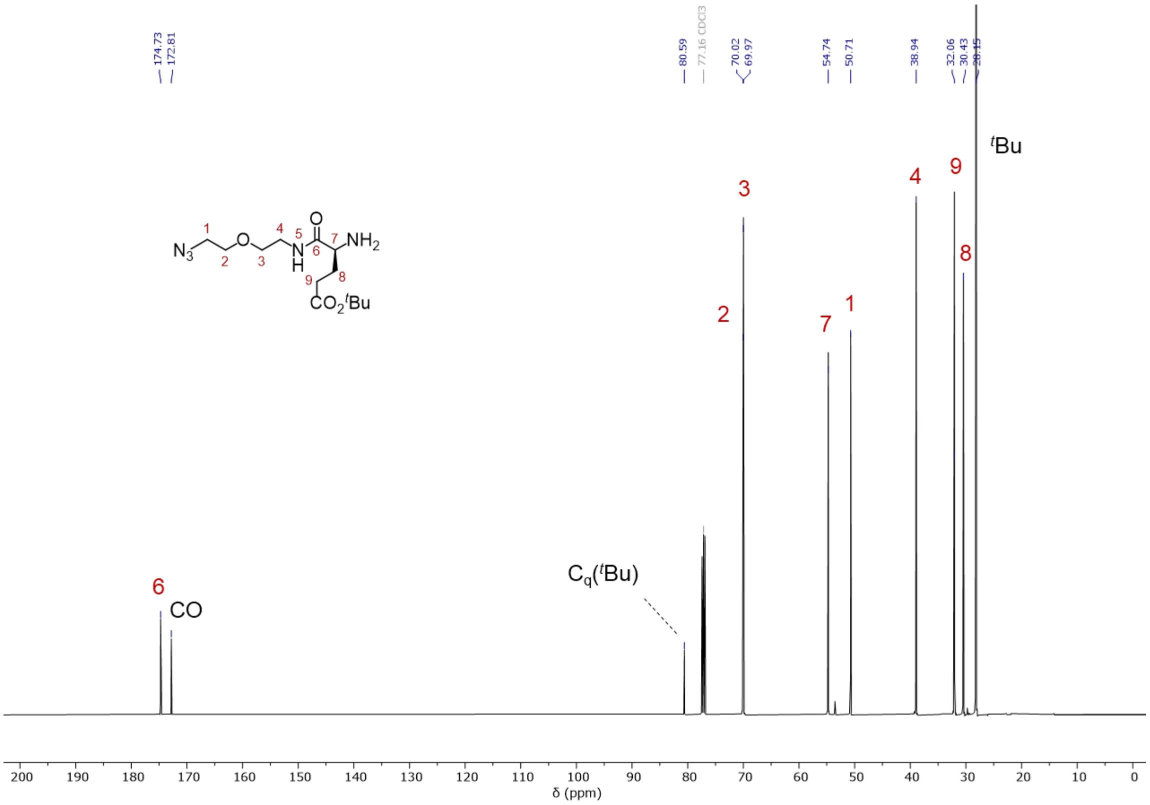

Figure S78. 126 MHz ^{13}C NMR spectrum of compound **47** in $CDCl_3$.

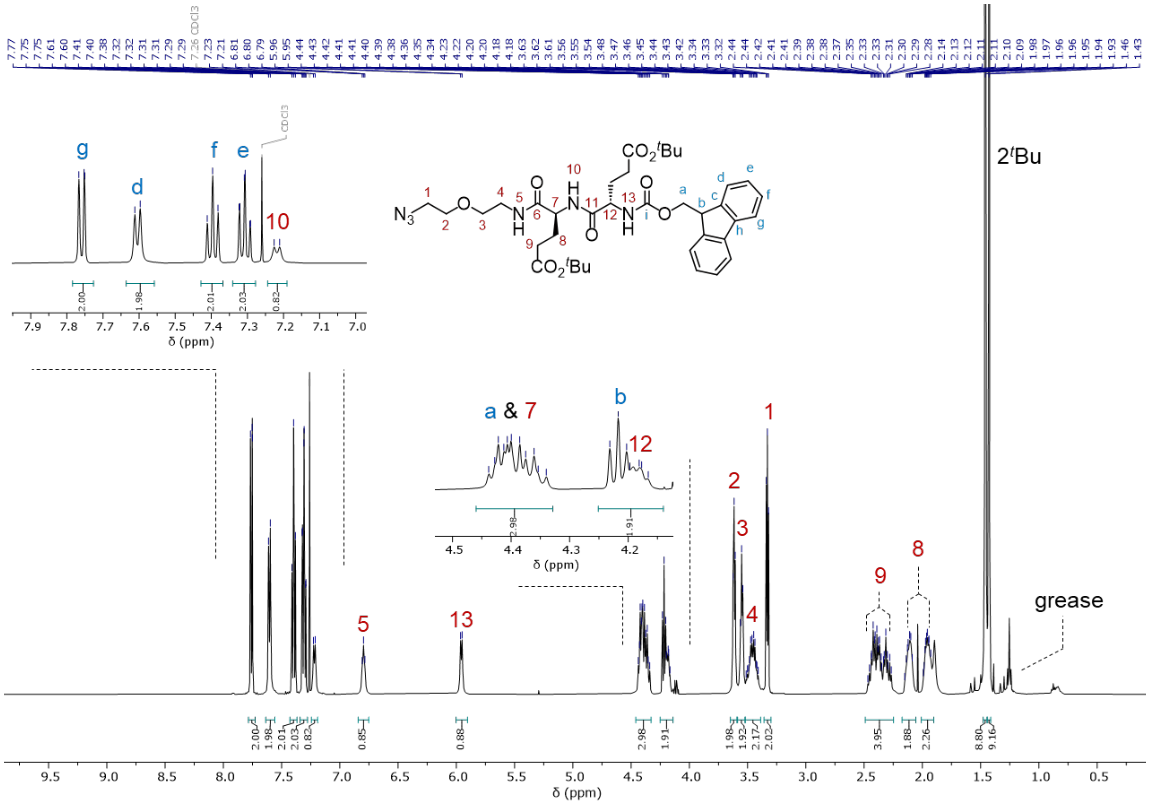

Figure S79. 500 MHz ^{1}H NMR spectrum of compound **48** in $CDCl_3$.

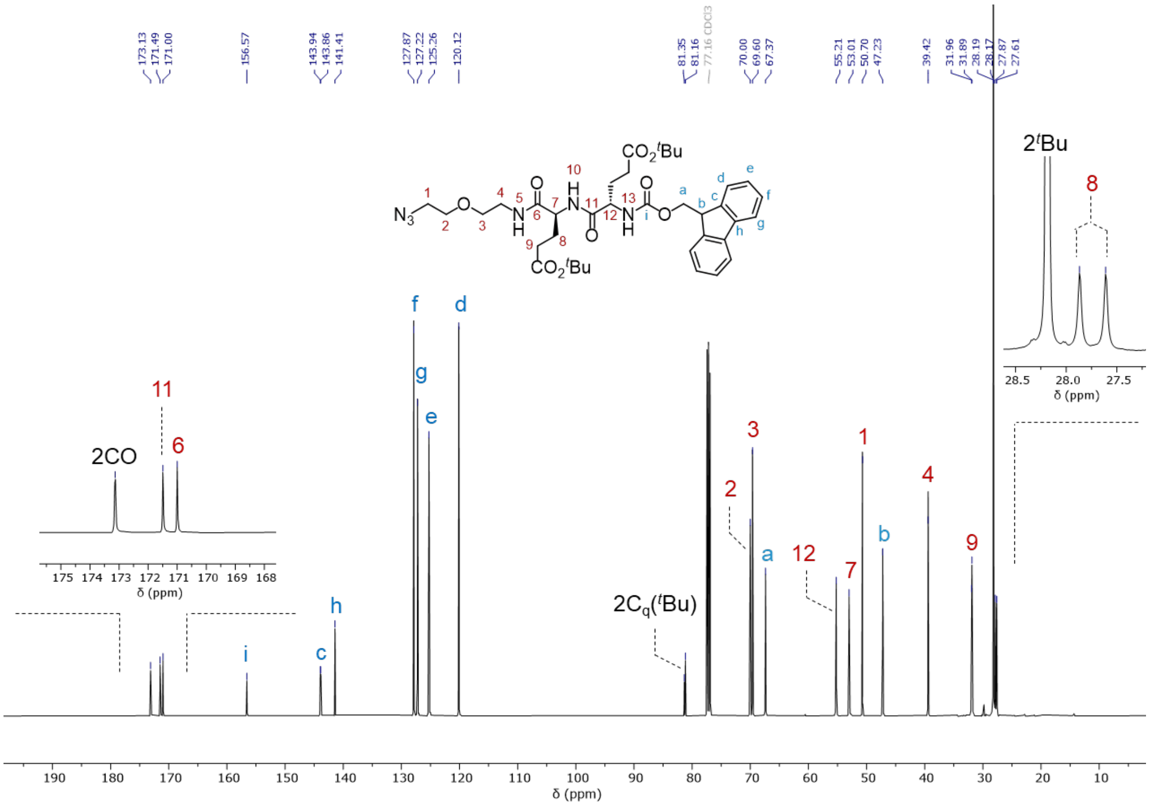

Figure S80. 126 MHz ^{13}C NMR spectrum of compound **48** in $CDCl_3$.

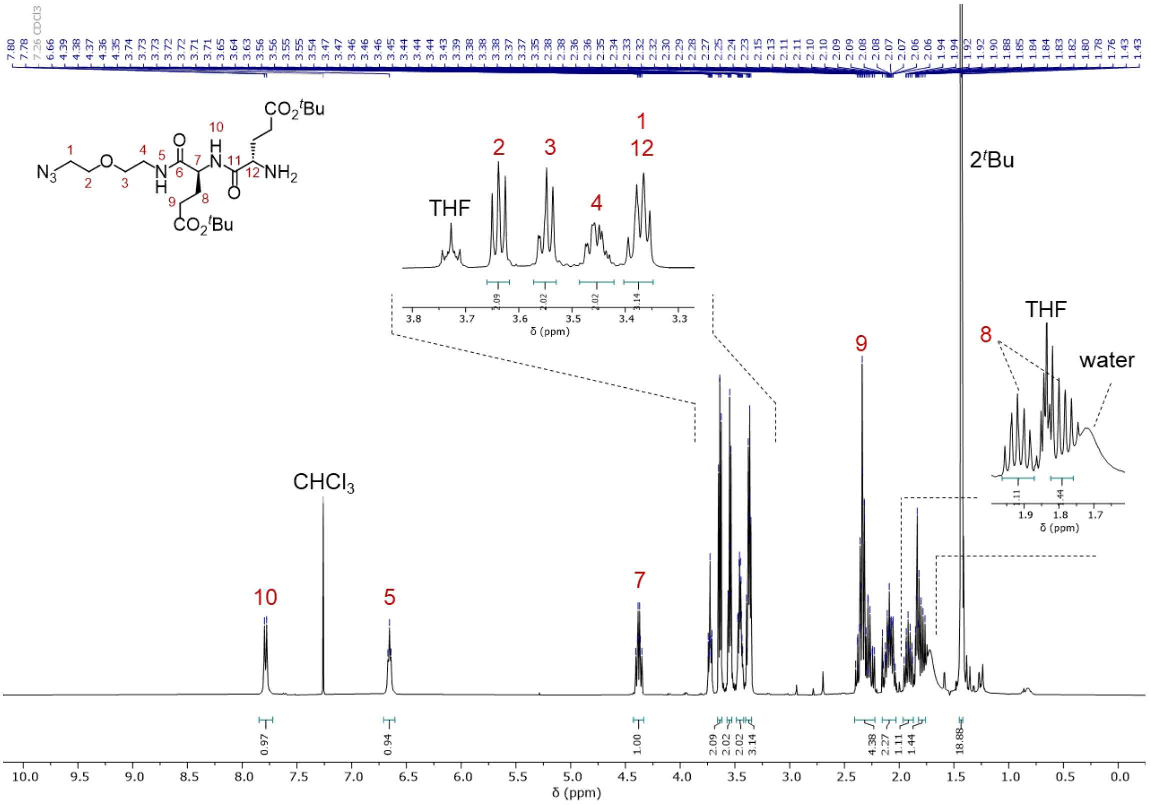

Figure S81. 500 MHz ^{1}H NMR spectrum of compound **49** in $CDCl_3$.

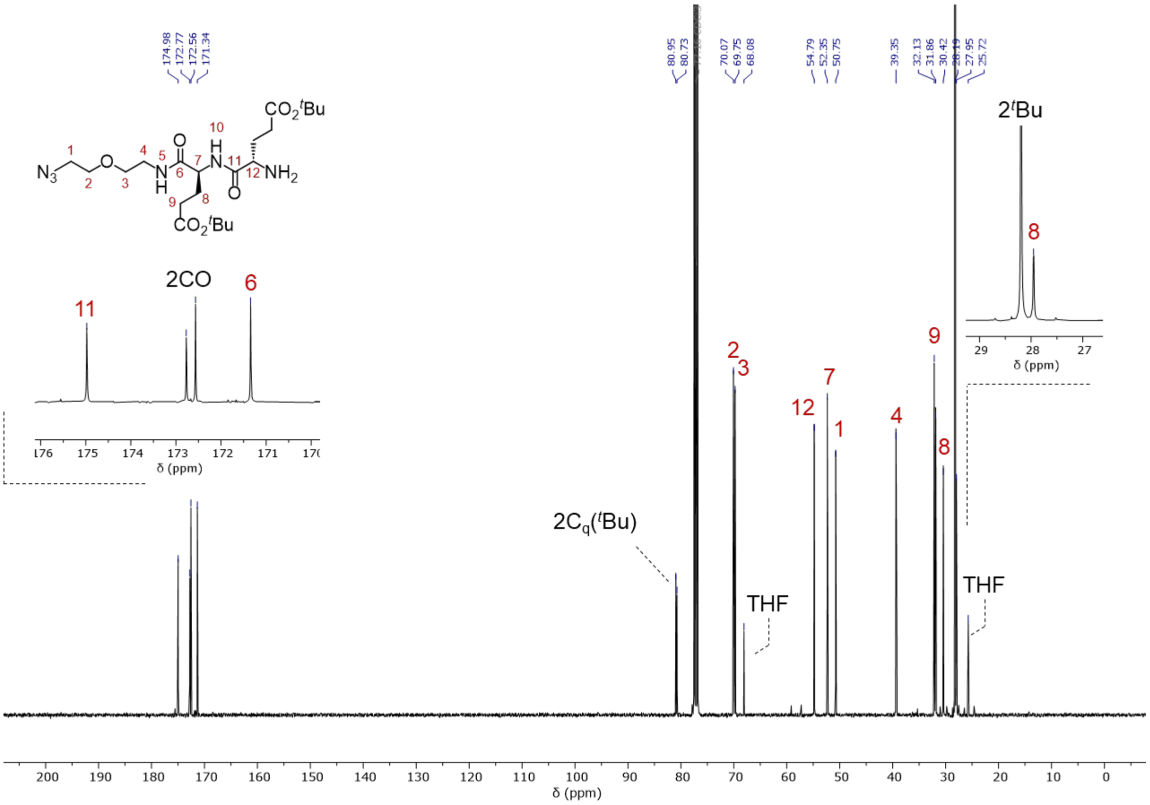

Figure S82. 126 MHz ^{13}C NMR spectrum of compound **49** in $CDCl_3$.

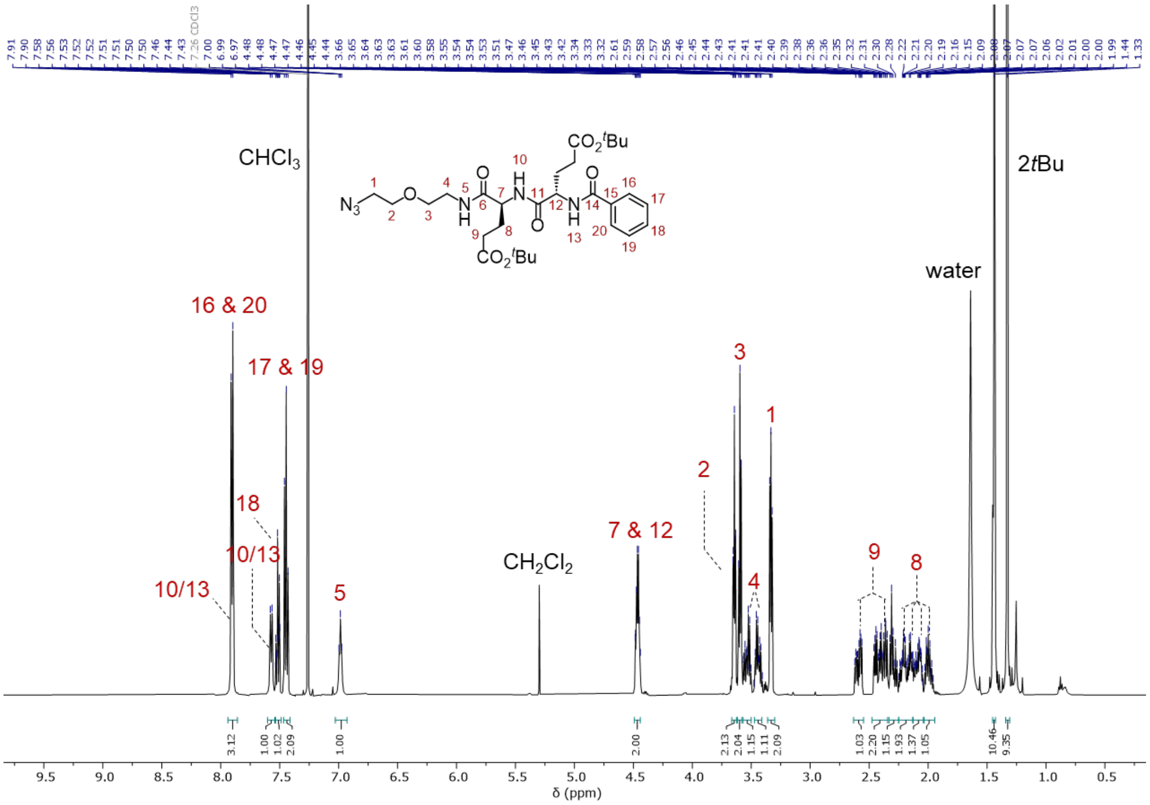

Figure S83. 500 MHz ^{1}H NMR spectrum of compound **50** in $CDCl_3$.

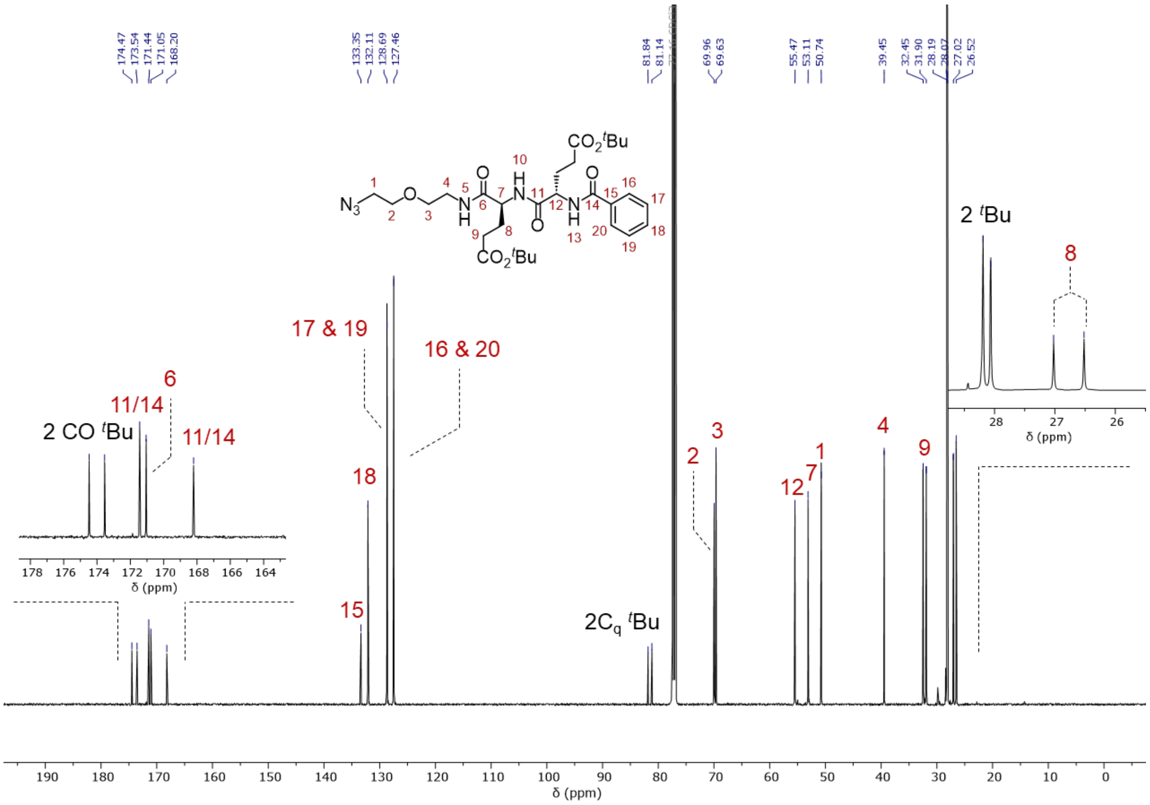

Figure S84. 126 MHz ^{13}C NMR spectrum of compound **50** in $CDCl_3$.

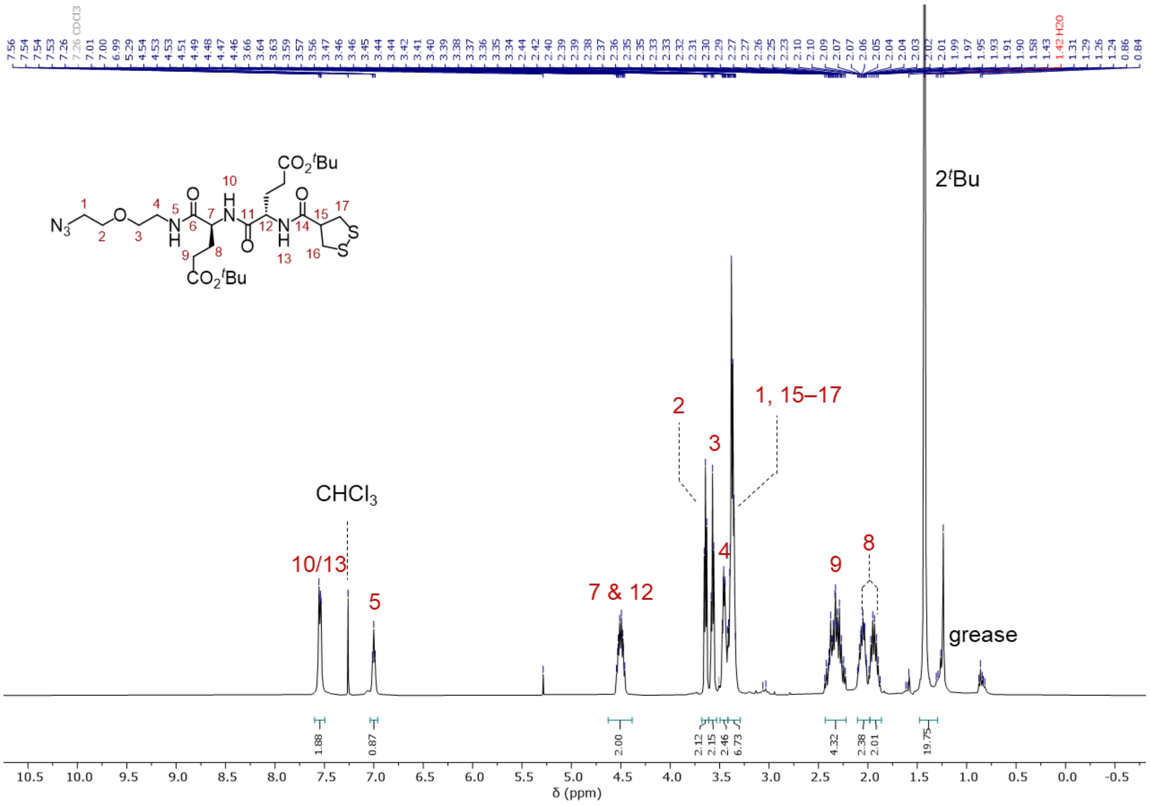

Figure S85. 400 MHz ^{1}H NMR spectrum of compound **52** in $CDCl_3$.

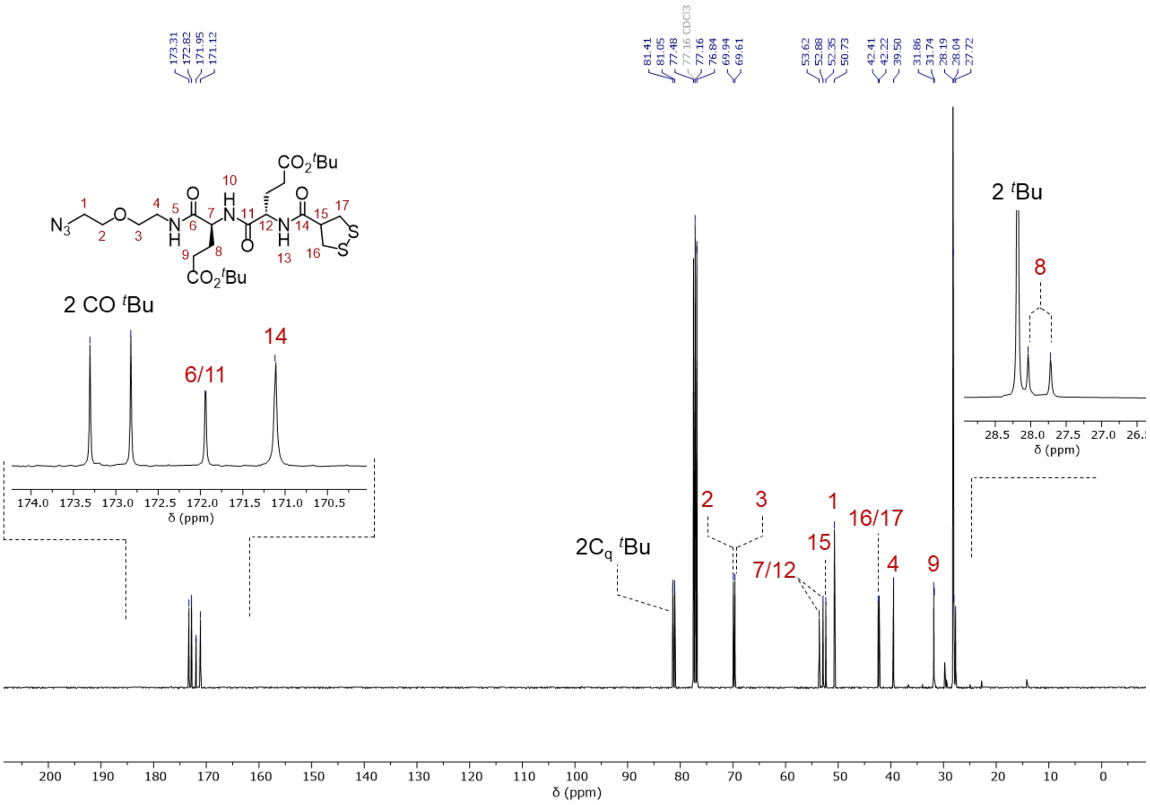

Figure S86. 101 MHz ^{13}C NMR spectrum of compound **52** in $CDCl_3$.

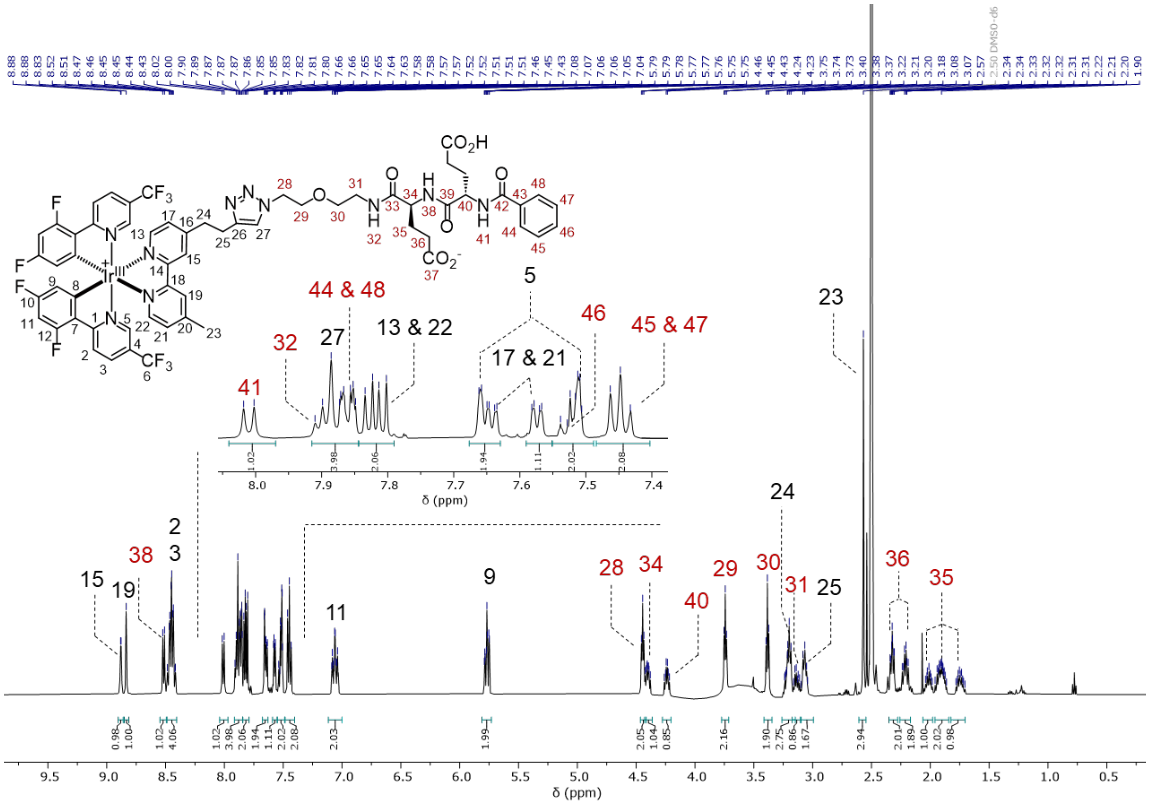

Figure S87. 500 MHz ^{1}H NMR spectrum of compound **6** in DMSO-d_6.

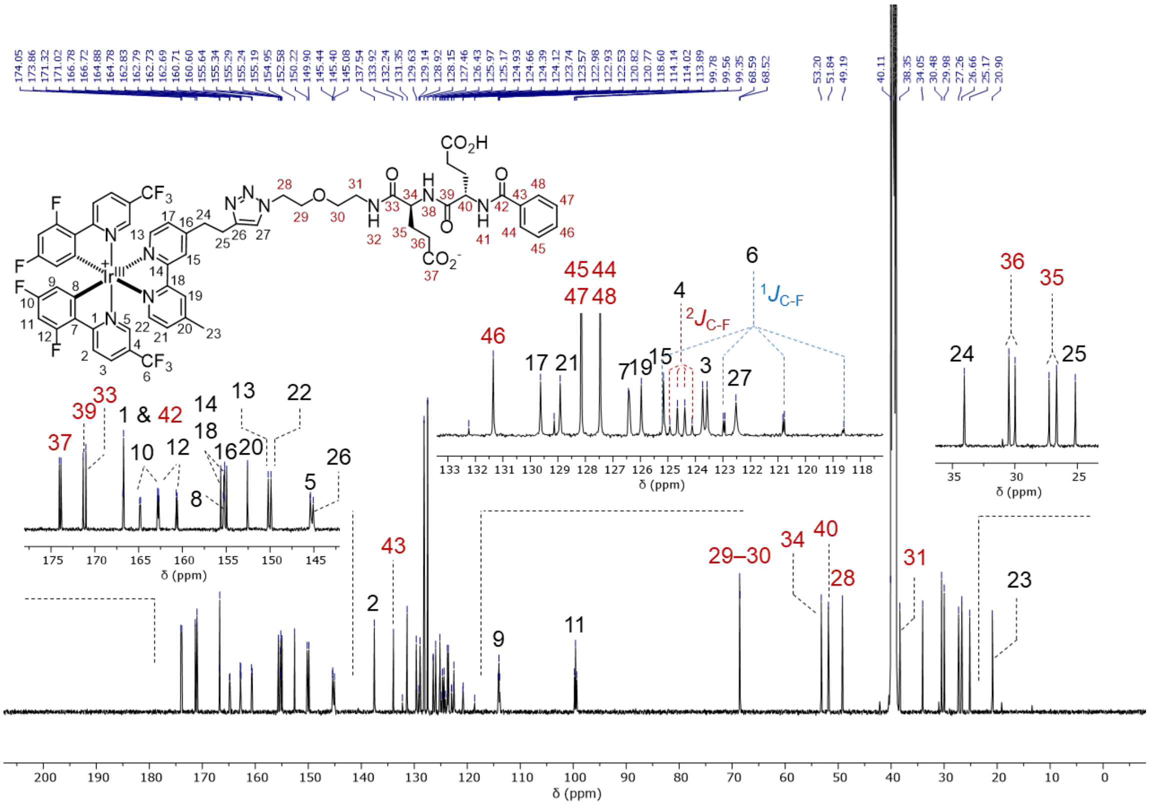

Figure S88. 126 MHz ^{13}C NMR spectrum of compound **6** in DMSO-d_6.

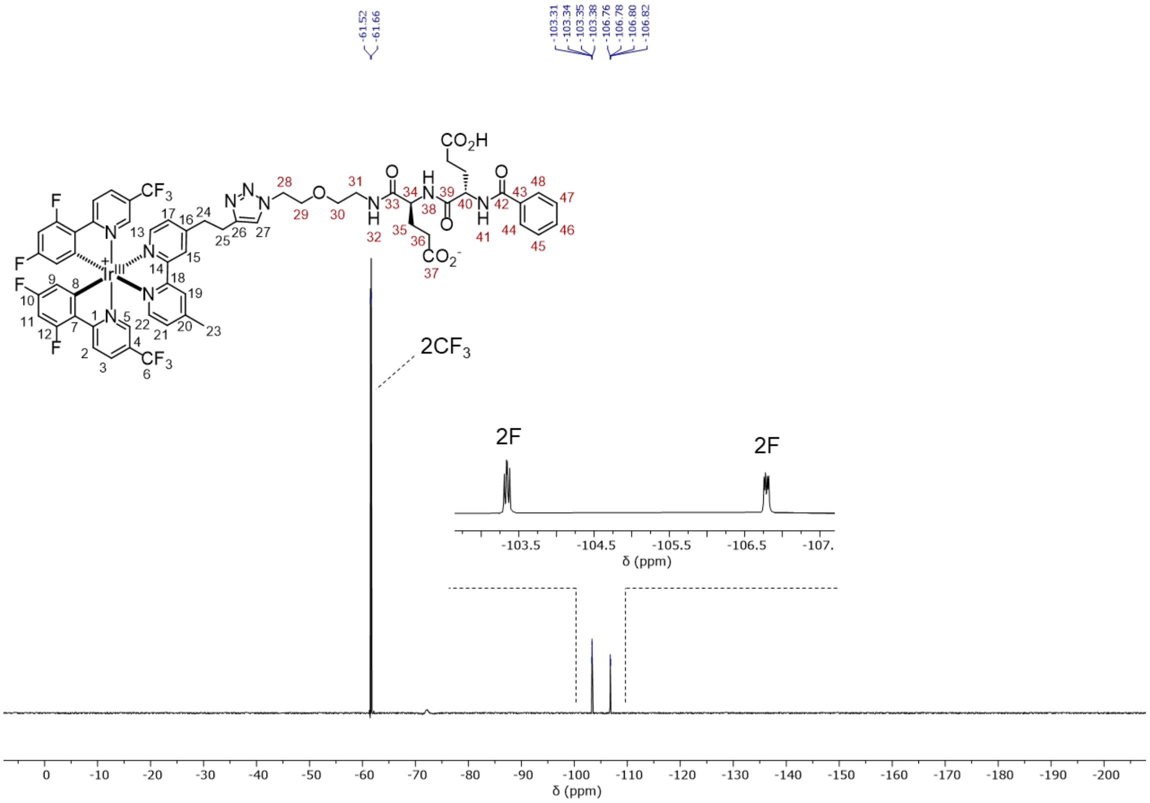

Figure S89. 282 MHz ^{19}F NMR spectrum of compound **6** in DMSO-d_6.

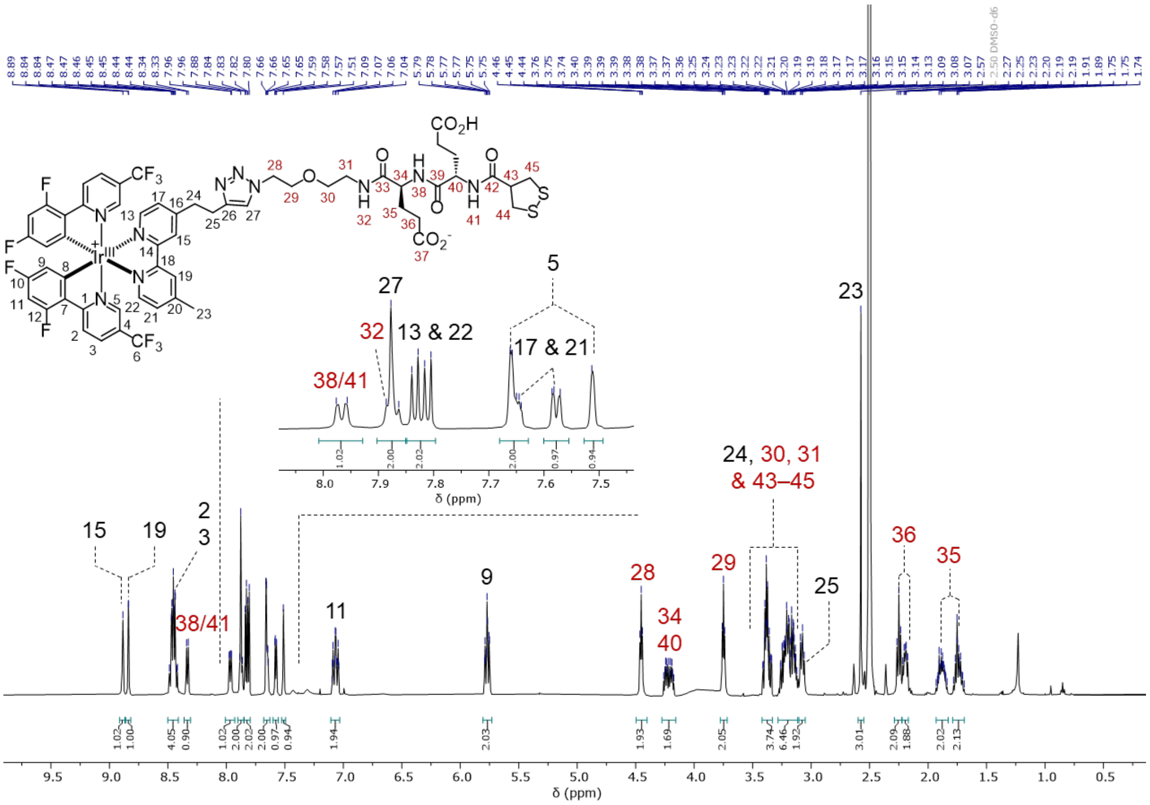

Figure S90. 500 MHz ^{1}H NMR spectrum of compound **5** in DMSO-d_6.

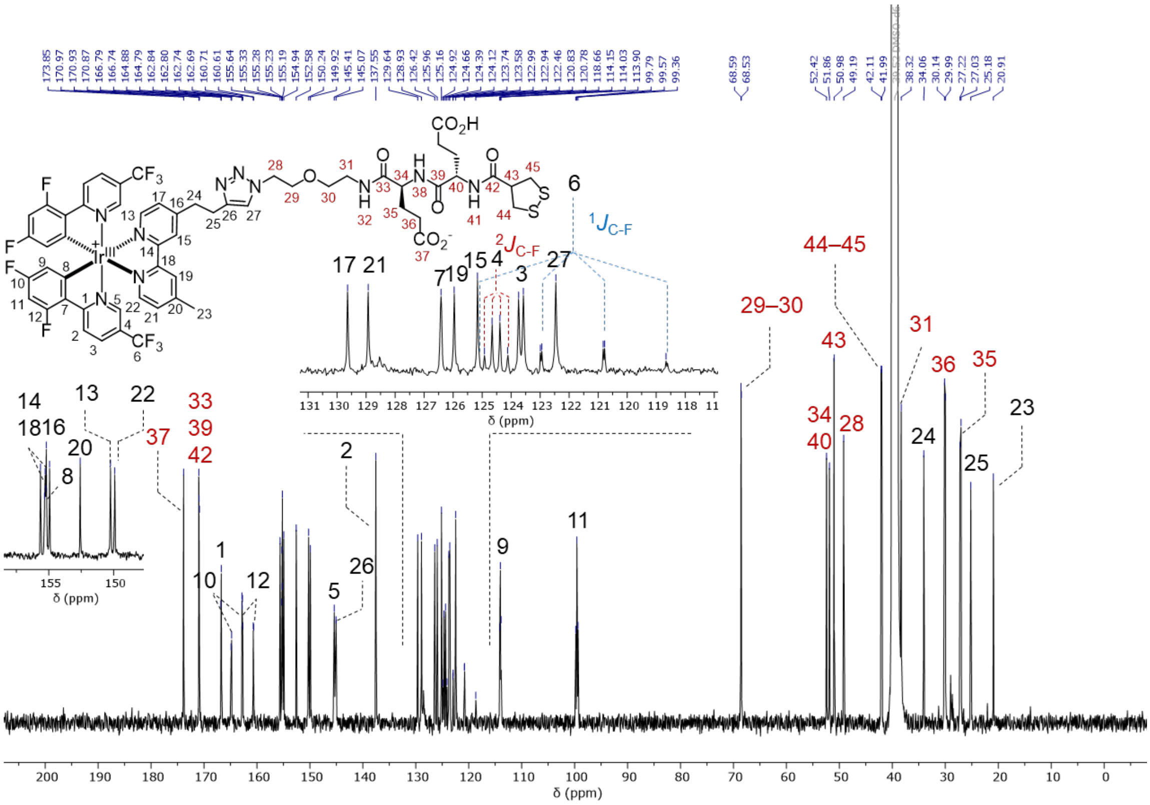

Figure S91. 126 MHz ^{13}C NMR spectrum of compound **5** in DMSO-d_6.

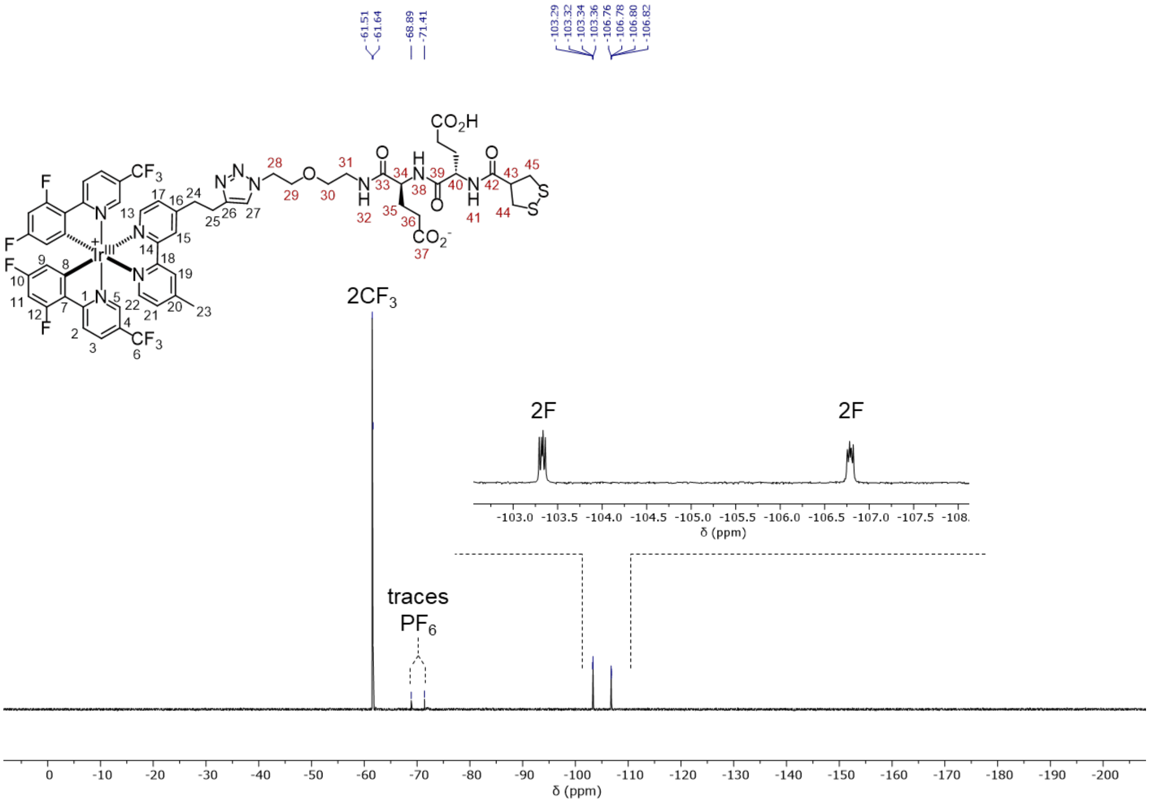

Figure S92. 282 MHz ^{19}F NMR spectrum of compound **5** in DMSO-d_6.
